# Phase 1/2a Study of the Malaria Vaccine Candidate Apical Membrane Antigen-1 (AMA-1) Administered in Adjuvant System AS01B or AS02A

**DOI:** 10.1371/journal.pone.0005254

**Published:** 2009-04-23

**Authors:** Michele D. Spring, James F. Cummings, Christian F. Ockenhouse, Sheetij Dutta, Randall Reidler, Evelina Angov, Elke Bergmann-Leitner, V. Ann Stewart, Stacey Bittner, Laure Juompan, Mark G. Kortepeter, Robin Nielsen, Urszula Krzych, Ev Tierney, Lisa A. Ware, Megan Dowler, Cornelus C. Hermsen, Robert W. Sauerwein, Sake J. de Vlas, Opokua Ofori-Anyinam, David E. Lanar, Jack L. Williams, Kent E. Kester, Kathryn Tucker, Meng Shi, Elissa Malkin, Carole Long, Carter L. Diggs, Lorraine Soisson, Marie-Claude Dubois, W. Ripley Ballou, Joe Cohen, D. Gray Heppner

**Affiliations:** 1 United States Military Malaria Vaccine Program, Walter Reed Army Institute of Research, Silver Spring, Maryland, United States of America; 2 Program for Appropriate Technology in Health (PATH) Malaria Vaccine Initiative, Bethesda, Maryland, United States of America; 3 Department of Medical Microbiology, Radboud University, Nijmegen Medical Centre, Nijmegen, The Netherlands; 4 Department of Public Health, Erasmus MC, University Medical Center Rotterdam, Rotterdam, The Netherlands; 5 GlaxoSmithKline Biologicals, Rixensart, Belgium; 6 Statistics Collaborative, Incorporated, Washington D. C., United States of America; 7 Laboratory of Malaria and Vector Research, National Institute of Allergy and Infectious Diseases, National Institutes of Health, Bethesda, Maryland, United States of America; 8 Malaria Vaccine Development Program, United States Agency for International Development, Washington D. C., United States of America; Walter and Eliza Hall Institute of Medical Research, Australia

## Abstract

**Background:**

This Phase 1/2a study evaluated the safety, immunogenicity, and efficacy of an experimental malaria vaccine comprised of the recombinant *Plasmodium falciparum* protein apical membrane antigen-1 (AMA-1) representing the 3D7 allele formulated with either the AS01B or AS02A Adjuvant Systems.

**Methodology/Principal Findings:**

After a preliminary safety evaluation of low dose AMA-1/AS01B (10 µg/0.5 mL) in 5 adults, 30 malaria-naïve adults were randomly allocated to receive full dose (50 µg/0.5 mL) of AMA-1/AS01B (n = 15) or AMA-1/AS02A (n = 15), followed by a malaria challenge. All vaccinations were administered intramuscularly on a 0-, 1-, 2-month schedule. All volunteers experienced transient injection site erythema, swelling and pain. Two weeks post-third vaccination, anti-AMA-1 Geometric Mean Antibody Concentrations (GMCs) with 95% Confidence Intervals (CIs) were high: low dose AMA-1/AS01B 196 µg/mL (103–371 µg/mL), full dose AMA-1/AS01B 279 µg/mL (210–369 µg/mL) and full dose AMA-1/AS02A 216 µg/mL (169–276 µg/mL) with no significant difference among the 3 groups. The three vaccine formulations elicited equivalent functional antibody responses, as measured by growth inhibition assay (GIA), against homologous but not against heterologous (FVO) parasites as well as demonstrable interferon-gamma (IFN-γ) responses. To assess efficacy, volunteers were challenged with *P. falciparum*-infected mosquitoes, and all became parasitemic, with no significant difference in the prepatent period by either light microscopy or quantitative polymerase chain reaction (qPCR). However, a small but significant reduction of parasitemia in the AMA-1/AS02A group was seen with a statistical model employing qPCR measurements.

**Significance:**

All three vaccine formulations were found to be safe and highly immunogenic. These immune responses did not translate into significant vaccine efficacy in malaria-naïve adults employing a primary sporozoite challenge model, but encouragingly, estimation of parasite growth rates from qPCR data may suggest a partial biological effect of the vaccine. Further evaluation of the immunogenicity and efficacy of the AMA-1/AS02A formulation is ongoing in a malaria-experienced pediatric population in Mali.

**Trial Registration:**

www.clinicaltrials.gov NCT00385047

## Introduction

Malaria, particularly due to *Plasmodium falciparum*, is responsible for an estimated 1–2 million deaths per year [1]. The populations most affected by malaria are children less than five years of age and primigravidae [2] living in malaria endemic areas, and malaria-naïve individuals traveling to endemic areas. An effective malaria vaccine, in addition to other available preventive measures, is critical to mitigating the effects of this disease in these vulnerable populations.

A malaria vaccine able to prevent infections as well as control blood stage growth will likely require pre-erythrocytic and erythrocytic stage components that are able to elicit anti-parasitic humoral and cell-mediated immune responses [3]. Apical membrane antigen-1 (AMA-1) is an 83-kDa precursor protein localized in the micronemes at the apical end of the merozoite, the erythrocyte-invading stage of the parasite [4]. This precursor is proteolytically cleaved to form a 66-kDa protein which then translocates from micronemes to the surface of the merozoite [5] and as such is thought to mediate merozoite reorientation to the erythrocyte [6]. AMA-1 has also been found on the surface of sporozoites and on hepatic merozoites [7]; thus, this “blood stage” antigen may be a target for protective immune responses against both the invading sporozoite and liver stage of the parasite.

Several studies have shown that anti-AMA-1 antibodies may play a role in protective immunity in adults living in malaria-endemic areas, and while these studies did demonstrate cross-reactivity to heterologous alleles, the degree to which these antibodies reacted varied [8], [9]. Other studies with T-cells from naturally-exposed subjects have reported proliferation in response to peptides derived from AMA-1 [10], [11]. Preclinical studies in mice have demonstrated protection (survival with reduced parasitemia) against the rodent parasite *P. chabaudi* by active immunization with homologous recombinant AMA-1 protein formulated with potent adjuvants, as well as by passive transfer of immunoglobulin from vaccinated rabbits [12]. Additionally, active immunization of rhesus monkeys with *P. knowlesi* AMA-1 adjuvanted in saponin resulted in some animals demonstrating a delayed prepatent period when challenged with *P. knowlesi* schizonts [13]. In *Aotus* monkeys immunized with *P. falciparum* recombinant AMA-1 in complete Freund's adjuvant, significant delays in parasitemia after homologous blood stage challenge were seen as compared to monkeys immunized with a similarly adjuvanted control malarial antigen [14]. T-cell responses to AMA-1 were detected in naïve adult volunteers immunized with irradiated *P. falciparum* sporozoites [15], suggesting that AMA-1 may be able to elicit cellular host immune responses to act against pre-erythrocytic stages of *P. falciparum* infection.

Two Phase 1 dose-escalation adult vaccine trials have been completed, one at WRAIR and one in Mali, evaluating FMP2.1, an AMA-1 recombinant protein vaccine based on the 3D7 allele, formulated with the GlaxoSmithKline (GSK) proprietary Adjuvant System, AS02A [16], [17]. Both studies demonstrated the vaccine to be well-tolerated and immunogenic. Recent preclinical data suggests another GSK Adjuvant System, AS01B, may be more potent than AS02A, which may translate into improved efficacy of vaccines adjuvanted with this System [18]–[20]. The current Phase 1/2a study was the first to compare the safety and immunogenicity of an AMA-1-based vaccine in both AS01B and AS02A Adjuvant Systems, and the first to assess the efficacy of such a vaccine in malaria-naïve adults using a homologous primary sporozoite challenge model [21], thus contributing key information to the development process of a multi-component malaria vaccine [22]. Further adding to such a process, the ongoing Phase 1b and 2b FMP2.1/AS02A pediatric vaccine studies in Mali, while with a single adjuvant and single AMA-1allele, will provide valuable information regarding the mechanisms and cross-reactivity of the immune response to *P. falciparum* in an endemic pediatric population.

## Methods

### Participants

The protocol for this trial and supporting CONSORT checklist are available as supporting information; see Checklist S1 and Protocol S1. This study was conducted from September 2006 through April 2007 at the Clinical Trials Center of the Walter Reed Army Institute of Research (WRAIR), Silver Spring, Maryland. Healthy malaria-naïve adults aged 18–50 years were recruited by non-coercive means in the metropolitan Washington DC area using inclusion and exclusion criteria described previously [16].

### Ethics

The protocol was reviewed and approved by the WRAIR Human Use Review Committee, the United States Army Medical Research and Materiel Command Human Subjects Review Board, as well as the Western Institutional Review Board, representing the study partner PATH Malaria Vaccine Initiative (MVI). Investigators described the protocol to potential volunteers face to face, and informed consent was obtained by the use of a written IRB-approved consent form, signed and dated by the volunteer and the investigator who conducted the informed consent discussion. This study was conducted according to the Declaration of Helsinki as well as principles of Good Clinical Practices under the United States Food and Drug Administration Investigational New Drug (IND) application BB-13089.

### Interventions

The WRAIR recombinant AMA-1 vaccine antigen termed FMP2.1 was produced in *Escherichia coli*, Tuner™ strain (Novagen, Madison, WI) under current good manufacturing practices (cGMP) at the WRAIR Pilot Bioproduction Facility and bottled as a highly purified and lyophilized recombinant protein. The protein consists of 449 amino acids representing the majority of the ectodomain (amino acids 83–531) of the 3D7 variant of AMA-1 with N- and C-terminal His-tags. The method of AMA-1 recombinant protein production and purification has been described previously [23], and FMP2.1 differs from the method described only in the *E. coli* strain used for production. FMP2.1 stability assays and potency tests in mice were carried out according to International Conference on Harmonisation (ICH) guidelines and confirmed the vaccine antigen was stable and potent from date of manufacture through preparation of this manuscript. The two proprietary Adjuvant Systems used in this study were produced by GlaxoSmithKline Biologicals (GSK), Rixensart, Belgium and have been described and evaluated in multiple vaccine products [24]. AS01B is a liposome-based Adjuvant System such that the 0.5 mL final dose contains 50 µg 3-deacylated-monophosphoryl lipid A (3-D-MPL), a TLR4 ligand derived from *Salmonella minnesota* and 50 µg of QS21, a highly purified saponin extract from the bark of the South American tree *Quillaja saponaria*, while AS02A is based on an oil-in-water emulsion with the same amounts of 3-D-MPL and QS-21. The lyophilized FMP2.1 antigen, Lot #1046, was packaged in single dose vials of approximately 60 µg. For full dose vaccine administration, the lyophilized contents were mixed with the prepackaged Adjuvant Systems AS02A or AS01B, resulting in an estimated final dose of 50 µg AMA-1 in 0.5 mL of Adjuvant System. The 10 µg dose was prepared by mixing five vials of AS01B with one vial of FMP2.1 antigen and withdrawing 0.5 mL for sterile injection. The adjuvant and antigen were stored at 2–8° Celsius. On vaccination days, the vials were placed on wet ice no longer than 4 hours, and the vaccine formulated immediately prior to injection. The vaccine constituents have undergone extensive stability testing and have been show to be stable for up to 24 hours at room temperature. For clarity, the FMP2.1 vaccine antigen herein will be referred to as AMA-1 in this publication.

This was a first-in-human study of AMA-1/AS01B; therefore, a 3-week staggered dosing schedule of this formulation was incorporated into the study design. In the open-label phase of the study, 5 volunteers received 10 µg AMA-1 in 0.5 mL of AS01B (referred to as the low dose AMA-1/AS01B group) administered intramuscularly in the non-dominant arm. A Safety Monitoring Committee (SMC) reviewed the safety data accumulated during the four days following the first immunization and recommended commencing immunizations with the higher dose of AMA-1. Thirty volunteers were assigned in a double-blind, randomized fashion to receive either 50 µg of AMA-1 in 0.5 mL of AS01B (referred to as the full dose AMA-1/AS01B group) or 50 µg AMA-1 in 0.5 mL of AS02A (referred to as the full dose AMA-1/AS02A group). A total of three immunizations were given at one-month intervals, all administered intramuscularly in alternate arms. The blinding code was broken after completion of the efficacy phase.

### Objectives

The primary objective of this trial was to assess the safety and reactogenicity of candidate malaria vaccines FMP2.1/AS01B and FMP2.1/AS02A when administered intramuscularly on a 0-, 1-, and 2-month (0-, 28- and 56-days) immunization schedule to malaria-naïve adult volunteers living in the United States. Secondary objectives were to assess the magnitude of humoral immune responses induced by the candidate malaria vaccines, the functionality of these anti-FMP2.1 antibodies to inhibit the growth of asexual parasites and to assess the efficacy of the vaccine candidates against sporozoite challenge with *P. falciparum*. The tertiary objective of the study was to assess the cell-mediated immune responses to FMP2.1/AS01B and FMP2.1/AS02A.

### Outcomes

To evaluate the primary objective of safety and reactogenicity of AMA-1/AS01B and AMA-1/AS02A, after each immunization, the occurrence of solicited symptoms over a 7-day follow-up period and unsolicited symptoms over a 30-day follow-up period, as well as any serious adverse events (SAEs) during the study period were collected. Solicited symptoms included local adverse events (pain, erythema and swelling) and systemic adverse events (fever, nausea, headache, malaise, myalgia, fatigue, and arthralgia). All symptoms were graded on a scale to indicate degree of functional impairment (Grade 0: no impairment, Grade 1: easily tolerated, Grade 2: interferes with daily activity, Grade 3: prevents daily activity) except for injection site erythema and swelling, which were graded as a physical measurement taken at the greatest diameter of involvement (Grade 0: 0 mm, Grade 1: ≥1–≤20 mm, Grade 2: >20–≤50 mm, Grade 3: ≥ 50 mm), and fever, which was graded on the following scale of oral temperature: Grade 0: ≤37.5°C, Grade 1: >37.5–≤38°C, Grade 2: >38–≤39°C, Grade 3: >39°C. Hematologic and biochemical tests for safety were collected on Days 0, 14, 28, 42, 56 and 70, and again at three months after challenge for those volunteers enrolled in the efficacy phase.

The secondary objectives were twofold: to assess vaccine immunogenicity and determine vaccine efficacy. Immunogenicity endpoints included anti-AMA-1 antibody titers as determined by Enzyme-Linked Immunoassay (ELISA), as well as functionality of anti-AMA-1 antibodies versus blood stage *P. falciparum* parasites as measured by standardized homologous (3D7 allele) and heterologous (FVO allele) growth inhibition assay (GIA).

#### Enzyme-Linked Immunoassay

Serum for anti-AMA-1 antibody determination was collected from each volunteer at Day 0, 14, 28, 42, 56, 70, 93, 114 and 156 and frozen at −20°C or below until tested as described previously [16]. The AMA-1 plate antigen was prepared from the same bulk FMP2.1 vaccine antigen lot as the test article. ELISA titers, initially defined as the serum dilution yielding an optical density of 1.0 in a standardized assay, were converted to µg/ml concentration values. As was done for a previous ELISA [25], commercially obtainable human IgG was utilized to develop an IgG-quantitation sandwich ELISA. Using this ELISA, the concentration of anti-AMA-1 antibody was determined for a single control serum sample. Each sample tested was then analyzed against this control (run on each test sample plate) to generate a normalized µg/ml value. The resulting data are summarized by vaccine group as Geometric mean Concentration (GMCs) with 95% confidence intervals (CIs).

#### Growth-Inhibition Assay

Serum samples were pre-absorbed with 5 µl of human red blood cells (RBC), at 50% hematocrit, per 100 µl of serum for 1 hour and tested at 20% for growth inhibition by measuring parasite lactate dehydrogenase (pLDH) activity [26]. Parasitized RBC (pRBC) cultures of both the 3D7 allele (homologous) and FVO allele (heterologous) at the early schizont stage were set up with pre-immune and immune sera at various concentrations at a 0.3% parasitemia and 1% hematocrit. Assay plates were sealed in bags containing 2.5% CO_2_, 2.5% O_2_, 90% N_2_ and incubated for 40 or 48 hours (cycle time of 3D7 and FVO parasites respectively). Cultures were then harvested by transferring 50 µl/well into phosphate buffered saline (PBS)-containing C-bottom plates and washed by spinning plates for 10 minutes at 10,000 g. Once completed, plates were frozen at −30°C until analysis. To measure the amount of pLDH activity, a substrate buffer containing 0.1 M Tris HCl, 50 mM Sodium-L-lactate, 0.255 Triton-X, 10 mg NBT, 10 µg/ml 3-Acetylpyridine, 10 U/ml diaphorase from *Clostridium klyiveri* (all reagents were obtained from Sigma, St. Louis, MO) was added to the plates. Colorimetric measurement at 650 nm was done after 30 minutes of reaction time using the SpectraMax Plus 384 spectrophotometer (Molecular Devices, Sunnyvale, CA). Pre-vaccination samples were run in parallel with the post vaccination samples, and calculation of growth inhibition was determined by using the formula: % inhibition = [1−[(OD immune serum−OD RBC)/(OD pre-immune serum−OD RBC)]]×100. Volunteers demonstrating ≥20% activity were considered responders, a reproducible cut-off value calculated based on mean inhibition of pre-immune sera+2 standard deviations. This cut-off value is the threshold for serum antibody-specific inhibition over the determined assay background levels (which vary between individuals) and can be caused by nutritional differences and handling artifacts of the sera [26].

GIAs were also performed in parallel at the GIA Reference Center at the National Institutes of Health by Dr. Carole Long. Performing both methods provided an opportunity to provide comparability and bridging information regarding the inhibitory antibody responses obtained by the two assays. The NIH assay similarly measures inhibition of pLDH activity of both 3D7 and FVO alleles of *P. falciparum* [26]; however, a purified immunoglobulin preparation from volunteer serum samples is used as compared to 20% diluted serum [27]. Results in this manuscript are reported for an immunoglobulin concentration of 4 mg/mL for Day 70 serum samples using the same equation for calculation of percent inhibition.

#### Efficacy

Efficacy was measured as development of parasitemia and time to parasitemia after malaria challenge as determined by light microscopy. Approximately two weeks after completion of all three immunizations (Day 70), volunteers from the high dose vaccine groups underwent homologous sporozoite challenge. Six healthy, malaria-naive adults aged 18 to 50 years were also enrolled as non-immunized infectivity controls. The sporozoite challenge was conducted as described previously [21], [28]. Volunteers were monitored closely with daily visits and blood smears starting five days after challenge. Two hundred high-powered fields were examined on each blood film to detect patent parasitemia. This number was increased to 1000 fields if the volunteer became symptomatic, and in the case of persistent symptoms, additional fresh blood smears were prepared and read at 6- to 8-hour intervals. Once a volunteer was found to be parasitemic, a directly observed oral regimen of chloroquine was administered, and three consecutive negative daily blood smears were taken to ensure parasite clearance. Thereafter, weekly blood smears were prepared over the next 4-week period. Vaccine efficacy (VE) was defined as VE = incidence in unvaccinated (I_unvac_)−I_vac_/I_unvac_).

Additional endpoints included determination of cell-mediated immune responses by IFN-γ ELISPOT assays and quantification of peripheral parasitemia by quantitative PCR (qPCR).

#### IFN-gamma Enzyme-linked Immunospot assay (IFN-γ ELISPOT)

Peripheral blood mononuclear cells (PBMCs) from peripheral blood were isolated and e*x vivo* IFN-γ ELISPOT assays performed as described in the initial Phase 1 AMA-1/AS02A study previously conducted at WRAIR [16]. Briefly, cryopreserved PBMCs were cultured for 24 hours in triplicate wells in a 96-well culture plate at 200,000 PBMCs per well in the presence of three concentrations of FMP2.1 (0.1 µg/mL, 1 µg/mL or 10 µg/mL) or medium control. At the conclusion of this step, which acts to decrease non-specific and/or background responses, the PBMCs were transferred to a multi-screen 96-well filtration ELISPOT plate (Millipore Corporation, Bedford, MA) pre-coated with mouse anti-human monoclonal antibody, 1-DIK, (MabTech, Sweden) and cultured for another 18 hours. The PBMCs were decanted and plates washed with PBS, with subsequent addition of a biotinylated anti-IFN-γ antibody (MabTech AB, Sweden). The assay was developed using alkaline phosphatase-streptavidin (MabTech AB, Sweden) with addition of substrate (Bio-Rad, Hercules, CA). The spots were counted using an IPLab analyzer (Scan-analytics, Fairfax, VA) and the results were expressed as the mean of the triplicate wells' spot forming units (sfu) per 10^6^ PBMCs.

#### Quantitative PCR

Two mL blood samples were collected in EDTA tubes on the morning of the Day of Challenge (DOC) and then daily starting five days after challenge until the volunteer became parasitemic as detected by blood film. Briefly, the blood sample was passed through a leukofilter (24-well filter plate with vacuum manifold, Whatman, Clifton, NJ) to remove human leukocytes, washed three times with PBS and the DNA extracted from a 500 µl sample using a Qiagen miniprep kit (Qiagen, Valencia, CA) [29]. The PCR reaction [30] amplified the 18S ribosomal RNA gene of *P. falciparum*. Primers and probe are as follows: Forward- 5′-GTA ATT GGA ATG ATA GGA ATT TAC AAG GT-3′, Reverse- 5′-TCA-ACT-ACG-AAC-GTT-TTA-ACT-GCA-AC-3′, Taqman probe- TGC-CAG-CAG-CCG-CGG-TAA-TTC (FAM and TAMRA labeled). Reaction contents totaled 25 µl and included 1 µl DNA template, 300 nM of each primer and 200 nM probe with 250 µM of each deoxynucleotide, 0.125 U Amplitaq Gold polymerase, 5 mM MgCl_2_, and 1× Taqman buffer. Cycling conditions were 15 minutes denaturing and activation at 95°C followed by amplification cycle of 15 seconds at 95°C, and 1 minute at 60°C for 45 cycles and conducted in the Opticon 2 machine (Biorad, Hercules, CA). Standards of known *P. falciparum* concentrations were made at dilutions from human blood at 3% parasitemia of 2×10^6^, 2×10^5^, 6×10^4^, 2×10^4^, 6×10^3^, 2×10^3^, 200, and 20 parasites per ml blood using SYBR Green dye and BD FacsCaliber (San Jose, CA) to enable construction of a standard curve. A whole blood sample containing no parasites was included as a negative control. Blood samples were filtered using the multi-well filter plates, and the DNA was extracted. A standard curve was produced based on the mean of data points from five independent experiments. The threshold cycle (Ct) was defined as the cycle at which the fluorescence of the samples crossed two standard deviations above the mean background fluorescence of the negative (uninfected human blood DNA). The standard curve R squared value was 0.994 and results were expressed as parasites per mL. A previously described statistical model [31] was used to estimate (1) reduction of growth rate (beta2), and (2) reduction of the number of infected hepatocytes (X) per volunteer. Because of the limited number of qPCR data points, growth rates (with 95% CIs) were estimated per immunization group, and the parameters of duration of parasitemia and prepatent period fixed with their respective variance (β_1_, μ_1_,_2,3_ and *σ*_1_,_2,3_ ) according to values from the previous study [31].

### Sample Size

The group sizes were designed to mirror previous Phase 1 and Phase 2a malaria vaccine challenge studies performed using the WRAIR challenge model. The logistics of the challenge model limits the total number of volunteers challenged with malaria. The study has 80% power to detect a prolongation in the prepatent period of approximately two days in immunized volunteers as compared with controls. This study was designed to assess safety, immunogenicity, and efficacy of two candidate vaccines and not for the support of vaccine intergroup comparisons.

### Randomization

The five volunteers who received the low dose of AMA-1/AS01B were not randomized nor blinded to the vaccine administered in order to conduct the appropriate safety evaluation of this vaccine formulation prior to proceeding with a higher dose formulation. For volunteers receiving the full dose of vaccine, prior to the day of first immunization, a computerized randomization list was generated by Statistics Collaborative, Inc. assigning volunteers a randomization code number to uniquely identify the group to which the volunteer belonged. The set of individual randomization code numbers was kept in code break envelopes by the medical monitor.

### Blinding

As the adjuvants were different in presentation (vials versus pre-filled syringes), specific steps were implemented to insure that vaccines were administered and evaluated in a double-blind manner. An Immunization Team was created consisting of one physician and one study coordinator and was responsible for maintaining the blind during vaccine preparation and administration. These team members were not involved in the clinical evaluation of vaccine safety and reactogenicity during the Immunization Phase. At the conclusion of the malaria challenge, the unbroken code envelope was given by the medical monitor to the PI for breaking the study blind and beginning efficacy data analysis. Safety data analysis was performed by Statistics Collaborative, Inc after the last study visit had concluded. Immunogenicity data was not unblinded to sub-investigators until after all endpoint assays were complete.

### Statistics

Study data for demographics, solicited and unsolicited adverse events, and clinical laboratory tests for all enrolled volunteers who received at least one immunization, as well as efficacy as determined by light microscopy, were entered into a database, queried, verified and locked prior to analysis. For ELISA data, all titers were log-transformed and analysis of variance (ANOVA) was used to assess the difference between vaccine groups at baseline, with a repeat measure ANOVA to evaluate the effect of the vaccine on longitudinal data from day 14 post-immunization. For GIA and ELISPOT data, statistical analysis by ANOVA was used, and for PCR results were expressed as day of patency or parasites/mL and compared by Kaplan Meier survival curve (log rank test) or repeat measures ANOVA (log transformed) and Student's t-test respectively. Tukey's Test was used for all post-hoc pairwise comparisons. Analysis for comparisons among endpoints and prepatent periods used Spearman correlation or Mann-Whitney tests and were done with GraphPad Prism software 4.0. All other analyses were implemented using SAS 9.1.3 software. All statistical tests were two-sided with an alpha of 0.05.

## Results

### Participant Flow

**Figure 1:**
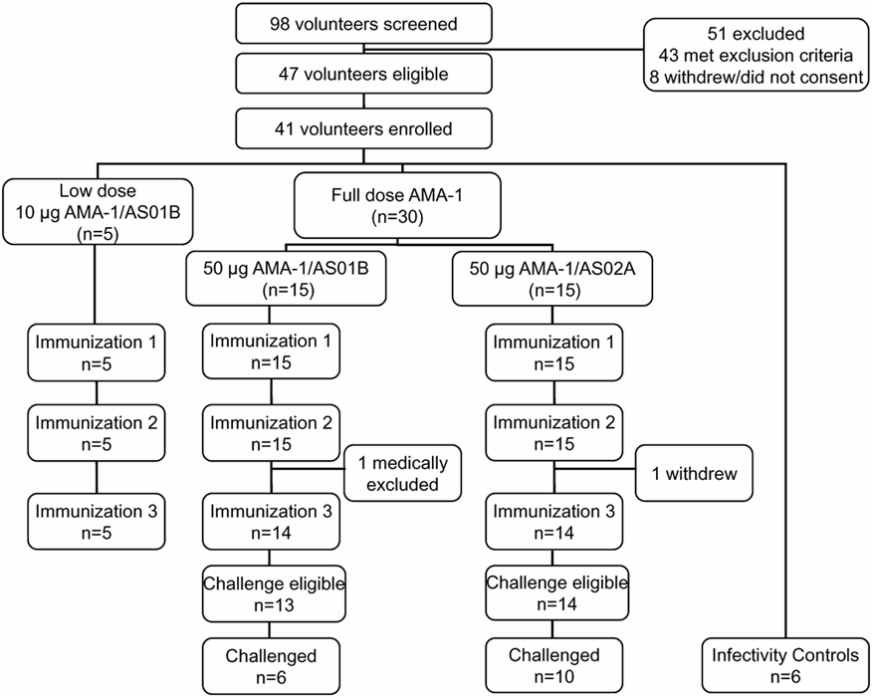
Participant flow diagram.

As shown in the study flow diagram (Figure 1), 98 adults underwent screening. Of these, 47 were eligible, 35 were enrolled as vaccinees, and 6 were enrolled as infectivity controls. The recruitment period began September 11, 2006, was completed for vaccinees at the end of October 2006, and a second brief recruitment period for infectivity controls occurred during the month of January 2007.

### Baseline data

The demographic make-up of the enrolled volunteers is shown in Table 1 and was similar in ethnicity, sex and age among the vaccine groups and the infectivity controls. The percentage of military personnel enrolled in the study was 29%. Five volunteers were enrolled in the low dose group while 15 volunteers received full dose AMA-1/AS01B and 15 volunteers received full dose AMA-1/AS02A. Thirty-three of 35 vaccinees received all three scheduled vaccinations; one volunteer (full dose AMA-1/AS02A recipient) withdrew due to a scheduling conflict, and another volunteer (full dose AMA-1/AS01B recipient) was withdrawn due to a clinical adverse event - a rash thought to be related to vaccine administration (see below). Of 27 vaccinees eligible for malaria challenge, 16 elected to undergo malaria challenge along with 6 unimmunized infectivity controls. The numbers analyzed for safety and immunogenicity included those volunteers meeting all eligibility criteria, complying with the procedures defined in the protocol, and for whom results were available after receiving at least one study vaccination. For efficacy results, only volunteers who underwent primary challenge were included.

**Table 1:** Study volunteer demographics.

	*10 µg AMA-1 in AS01B (n = 5)*	*50 µg AMA-1 in AS01B (n = 15)*	*50 µg AMA-1 in AS02A (n = 15)*	*Infectivity Controls (n = 6)*	*Overall (n = 41)*
***Sex, n (%)***					
*Male*	3 (60)	7 (47)	8 (53)	2 (33)	*20 (49)*
*Female*	2 (40)	8 (53)	7 (47)	4 (67)	*21 (51)*
***Age, years*** * Mean (SD)*	30.2 (10.5)	29.7 (6.0)	30.2 (8.7)	24.5 (7.7)	*29.2 (7.8)*
***Ethnicity/race, n (%)***					
*White*	2 (40)	10 (67)	13 (87)	5 (83)	*30 (73)*
*African-American*	2 (40)	3 (20)	0	1 (17)	*6 (15)*
*Hispanic or Latino*	1 (20)	1 (7)	1 (7)	0	*3 (7)*
*Asian*	0	1 (7)	0	0	*1 (2)*
*Middle Eastern*	0	0	1 (7)	0	*1 (2)*

### Outcomes and Estimation

#### Safety

A summary of solicited post-immunization adverse events (AEs) recorded in each 7-day follow-up period is presented in Table 2. Local injection site events were common and similar in prevalence among the three vaccine groups. Erythema and swelling increased in frequency and intensity with subsequent immunizations, with most (79–100%) volunteers in all groups experiencing Grade 3 swelling after the third vaccination. The erythema and swelling were not accompanied by significant functional impairment, and no Grade 3 injection site pain was reported.

**Table 2:** Local and systemic solicited adverse events (AEs) Day 0 through Day 7.

Vaccine	AE		Imm #1	Imm # 2	Imm #3
			% (%G3)	% (%G3)	% (%G3)
**10 µg AMA-1/AS01B**	***Local***	Pain	80 (0)	80 (0)	60 (0)
**n = 5**		Erythema	0 (0)	60 (40)	80 (60)
		Swelling	20 (0)	80 (60)	80 (80)
	***Systemic***	Fever	20 (0)	40 (0)	20 (0)
		Nausea	20 (0)	20 (0)	20 (0)
		Headache	40 (0)	40 (0)	60 (0)
		Malaise	20 (0)	80 (0)	60 (0)
		Myalgia	0 (0)	60 (0)	0 (0)
		Fatigue	20 (0)	60 (0)	80 (0)
		Joint Pain	0 (0)	20 (0)	20 (0)
**50 µg AMA-1/AS01B**	***Local***	Pain	100 (0)	93 (0)	93 (0)
n = 15*		Erythema	27 (13)	73 (27)	79 (57)
		Swelling	67 (47)	80 (47)	100 (100)
	***Systemic***	Fever	27 (0)	53 (7)	36 (0)
		Nausea	7 (0)	47 (0)	29 (7)
		Headache	33 (0)	60 (0)	57 (0)
		Malaise	33 (0)	73 (7)	64 (7)
		Myalgia	20 (0)	60 (7)	43 (0)
		Fatigue	40 (0)	67 (7)	57 (7)
		Joint Pain	20 (0)	33 (0)	14 (7)
**50 µg AMA-1/AS02A**	***Local***	Pain	93 (0)	93 (0)	86 (0)
n = 15*		Erythema	33 (0)	40 (33)	86 (57)
		Swelling	73 (33)	93 (67)	93 (79)
	***Systemic***	Fever	7 (0)	27 (0)	21 (0)
		Nausea	0 (0)	20 (0)	7 (0)
		Headache	27 (0)	47 (0)	43 (0)
		Malaise	13 (0)	40 (0)	43 (0)
		Myalgia	7 (0)	47 (0)	36 (0)
		Fatigue	13 (0)	53 (0)	43 (0)
		Joint Pain	0 (0)	0 (0)	0 (0)

Organized by AMA-1 dose and Adjuvant Group, AEs solicited post immunization with highest grade reported. Reported by percentage experiencing AE, with percent experiencing Grade 3 in parentheses. Imm = Immunization, G3 = Grade 3. * For third immunization n = 14.

The most common systemic adverse events in all three groups of immunized volunteers were headache, malaise, and fatigue. An increase in frequency and intensity of systemic adverse events in the second and third immunizations as compared to the first was also noted. Over the course of three immunizations, only 8 of 315 (3%) of solicited systemic events were rated Grade 3, and these occurred in five volunteers (14% of volunteers); all were immunized with full dose AMA-1/AS01B. Of these five volunteers, three reported systemic events after the second immunization and two after the third immunization. These few systemic Grade 3 events, as well as all other local and systemic adverse events, were short-lived, with 87% of all events occurring and resolving within the first 72 hours after immunization.

The number of volunteers experiencing any solicited AE, local or systemic, after each immunization was similar in the three groups (data not shown, Fishers exact test, p = 1.0), except after the first immunization, when the number of volunteers immunized with full dose AMA-1/AS01B experiencing at least one systemic adverse event was greater than those volunteers receiving full dose AMA-1/AS02A (11/15 volunteers versus 4/15 volunteers, respectively, Fishers exact test, p = 0.027). While the small number of volunteers limits statistical comparisons between each solicited adverse event at each immunization time point, the frequency of each solicited adverse event appeared comparable in all groups. No SAEs or clinically significant vaccine-related laboratory test abnormalities occurred during the execution of the study.

One unexpected adverse event did occur in a volunteer 18 days after receiving the second immunization with full dose AMA-1/AS01B. An erythematous, papular, slightly pruritic rash appeared intermittently over both deltoid areas, (corresponding to the two previous injection sites) during a 2-week period. There were no other associated local or systemic symptoms, and no therapeutic medications were required or administered. Hematologic and biochemical safety laboratory tests performed at the time were normal, and a biopsy of the site by a dermatologist revealed non-specific, chronic inflammation. The volunteer was withdrawn from the study and, over the next six months, the rash recurred three times without apparent provocation and eventually resolved without sequelae.

#### Immunogenicity-ELISA

**Figure 2:**
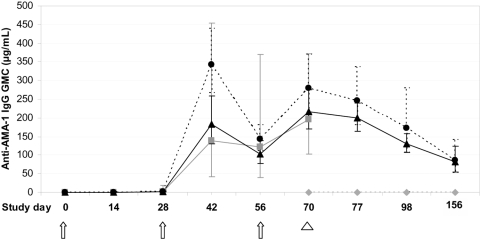
Geometric mean concentration (GMC) of anti-AMA-1 antibody by ELISA. Arrows indicate immunization time points and arrowhead indicates day of malaria challenge. Gray solid line with ▪ symbol: GMCs for low dose AMA-1/AS01B vaccinees; black solid line with ▴symbol: GMCs for high dose AMA-1/AS02A vaccinees; small black dashed line with • symbol: GMCs for high AMA-1/AS01B vaccinee;, small dashed gray line with ♦symbol: GMCs for infectivity controls. 95% CIs are shown for each time point.

Figure 2 shows the group GMCs of anti-AMA-1 antibodies as determined by antibody ELISA at specified time points during the study. Seroconversion, defined as concentrations >2 standard deviations (SD) above the group GMC at baseline, occurred in 100% of volunteers after second immunization, with peak GMC of antibody increasing at least 200-fold in all three vaccine groups. Two weeks post-third vaccination, anti-AMA-1 GMCs and 95% CIs were: low dose AMA-1/AS01B 196 µg/mL (103–371 µg/mL), full dose AMA-1/AS01B 279 µg/mL (210–369 µg/mL) and full dose AMA-1/AS02A 216 µg/mL (169–276 µg/mL). Among the three vaccine groups, there was no significant difference in antibody concentrations by longitudinal analysis (p = 0.55, repeated measures ANOVA) or by point-wise comparison with the exception of Day 42 when full dose AMA-1/AS01B induced higher concentrations than low and full dose AMA-1/AS02A (data not shown). While there was boosting with the third immunization, the maximum concentrations measured were not significantly higher than concentrations reached after second immunization for any vaccine group. Three months after the third immunization, antibody concentrations in those volunteers who underwent sporozoite challenge in both vaccine groups remained significantly greater than controls with an approximate half–life of approximately 52 days, similar to the half-lives seen over the post-vaccination period in malaria-naïve adults administered three doses of adjuvanted recombinant protein vaccine [16], [32].

#### Immunogenicity-GIA

**Figure 3:**
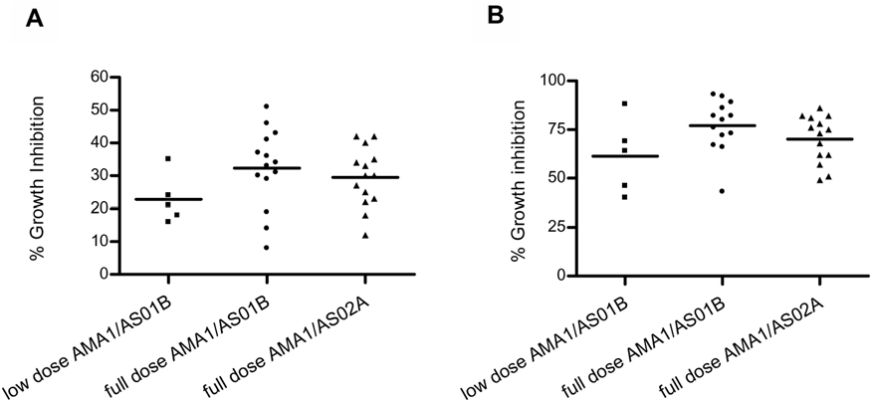
Mean percent growth inhibition activity of 3D7 parasites by GIA. (A) WRAIR 20% serum pLDH GIA (B) NIH pLDH GIA at 4 mg/mL purified immunoglobulin. Results expressed as mean percent inhibition with serum from Day 70 using GIA methods described in Outcomes section. Low dose AMA-1/AS01B (n = 5), full dose AMA-1 in AS01B or AS02A (n = 14 in each).

There was demonstrable growth inhibition by anti-AMA-1 antibodies at two weeks post-third immunization (Day 70/DOC) as measured by 20% serum GIA performed at WRAIR using homologous 3D7 parasites. In the low dose AMA-1/AS01B group, 60% (3 of 5) of volunteers were responders, while in the full dose AMA-1/AS01B group, 79% (11 of 14) and the full dose AMA-1/AS02A group, 86% (12 of 14) of volunteers were responders. As shown in Figure 3a, the mean percent growth inhibitory activity among all three groups was not significantly different: 23%, 32%, and 30% respectively (p = 0.22, one-way ANOVA). Inhibitory activity of all serum samples against heterologous FVO parasites was below the responder cut-off level of 20% (data not shown).

Assays performed at the GIA Reference Center at NIH with purified immunoglobulin also found similar mean percent inhibitions among the 3 vaccine groups (Figure 3b): low dose AMA-1/AS01B 61% inhibition, full dose AMA-1/AS01B 77% inhibition, full dose AMA-1/AS02A 70% inhibition, (p = 0.11, one-way ANOVA). The inhibitory activity was again limited to homologous 3D7 parasites, with no significant inhibition against FVO parasites demonstrated (data not shown). These two GIA methodologies, one using 20% serum and the other purified immunoglobulin, correlated well (Pearson's Correlation Coefficient r = 0.76, p<0.0001). As previously shown [16], the present study again demonstrated a good correlation of the anti-AMA-1 inhibitory activity, as measured by the WRAIR GIA, to WRAIR ELISA results (Spearman correlation coefficient r = 0.82, p<0.0001). In addition, NIH GIA results correlated well with WRAIR ELISA (Spearman correlation coefficient r = 0.77, p<0.0001) as well as NIH ELISA results (Spearman correlation coefficient r = 0.9, p<0.0001).

#### Immunogenicity-ELISPOT

**Figure 4:**
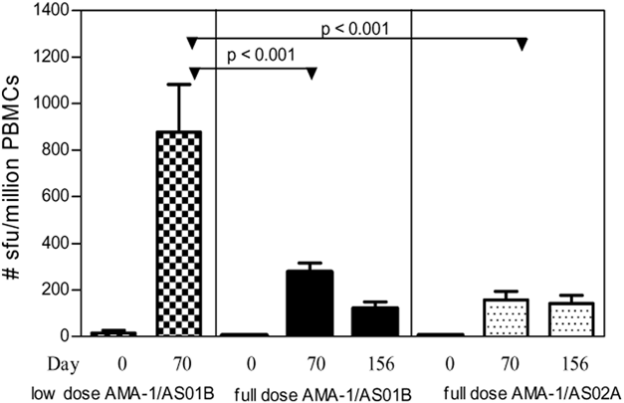
Comparison of IFN-γ ELISPOT results. Results expressed as mean sfu/million PBMCs with standard error bars. Low dose AMA-1/AS01B (n = 5), full dose AMA-1 in AS01B or AS02A (n = 14 in each). For Day 156, includes only challenged volunteers. Recombinant protein AMA-1 concentration 1.0 µg/mL. Assay also run at 0.1 µg/mL and 10 µg/mL with similar results (data not shown).

*Ex vivo* IFN-γ ELISPOT assays were performed using the PBMCs of volunteers at baseline, Day 70/DOC and three months post-challenge as shown in Figure 4 and expressed as mean sfu per million PBMCs. Baseline responses for each vaccine group were as follows: low dose AMA-1/AS01B = 14, full dose AMA-1/AS01B = 2 and full dose AMA-1/AS02A = 6. There was a demonstrable increase in IFN-γ production after the third immunization in all vaccine groups. Those volunteers immunized with low dose vaccine had greater production of IFN-γ at Day 70 as compared to the volunteers immunized with full dose AMA-1/AS01B or full dose AMA-1/AS02A (low dose AMA-1/AS01B = 877, full dose AMA-1/AS01B = 277, full dose AMA-1/AS02A = 158, p<0.001 one-way ANOVA, p<0.001 post-test Tukey's for low dose AMA-1 versus either high dose vaccine). There was no statistical difference in IFN-γ production between volunteers immunized with full dose AMA-1/AS01B or full dose AMA-1/AS02A at any time point.

#### Efficacy

**Figure 5:**
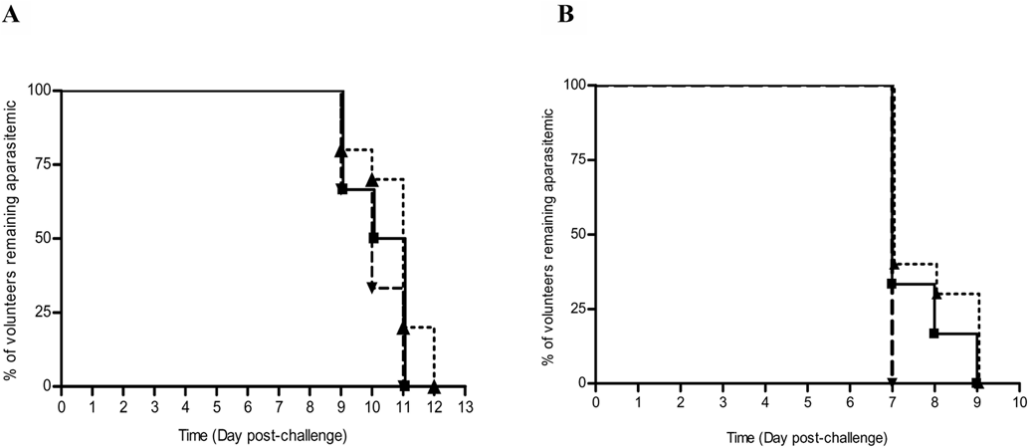
Kaplan-Meier (K-M) Survival Curve for prepatent period. Legend: — Full dose AMA-1/AS01B ^……^ Full dose AMA-1/AS02A – – – Infectivity Controls. (A) Prepatent period by thick blood film. Mean prepatent periods: full dose AMA-1/AS01B 10 days (240 hours), full dose AMA-1/AS02A 10 days 21 hours (261 hours), infectivity controls 10 days (240 hours), K-M survival curve log rank 2.94, P = 0.23. (B) Prepatent period by qPCR. Mean prepatent periods: full dose AMA-1/AS01B 7 days 12 hours (180 hours), full dose AMA-1/AS02A 7 days 17 hours (185 hours), infectivity controls 7 days (168 hours), K-M survival curve log rank 3.25, P = 0.19.

All 22 volunteers, 16 vaccinees and 6 controls, became parasitemic as determined by blood smear between 9 and 12 days after sporozoite challenge (Figure 5a), corresponding to a vaccine efficacy (VE) of 0%. There was also no difference in the mean prepatent periods as measured by light microscopy.

The prepatent period was also determined using qPCR from *P. falciparum* DNA isolated from peripheral blood drawn every morning (Figure 5b). The detection of parasitemia by PCR occurred approximately two days earlier than by blood film. On Day 7 post-challenge, all six infectivity controls became PCR positive, while four of six volunteers immunized with full dose AMA-1/AS01B and six of ten volunteers immunized with full dose AMA-1/AS02A did so; however, these differences are not statistically significant.

To further explore whether the high dose vaccines had an effect on parasite development in the liver or growth rate in the blood or both, we examined level of parasitemia from Day 7 (first parasite detection by PCR) up to and including Day 12 by qPCR. Among the three groups (two full dose vaccine groups and infectivity control group), there was a strong, statistically significant difference in longitudinal measurement of peripheral parasitemia on Days 7–9, the days in which peripheral parasitemia was detected and measured by qPCR and no treatment with chloroquine had yet been initiated (p = 0.0002, repeated measures ANOVA, data not shown). Post-test analysis showed significantly lower parasitemia in volunteers receiving full dose AMA-1/AS02A (Tukey's Test, p <0.0001), as well as a trend toward lower parasite burden in volunteers receiving full dose AMA-1/AS01B (Tukey's Test, p = 0.084) as compared to controls.

**Figure 6:**
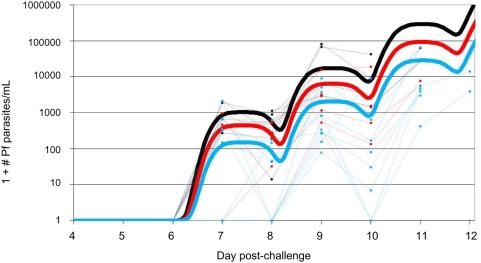
*In-vivo* growth of blood stage parasites after *P. falciparum* challenge. Observed parasite densities of individuals (dots) and predicted kinetics (as a group, thick continuous line) in the three immunization groups experimentally infected with *Plasmodium falciparum* (3D7). Dots represent observed number of parasites per milliliter of blood based on qPCR results. Individuals in the same group are represented in the same color (Infectivity Controls: black (n = 6), Full dose AMA-1/ASO1B: red (n = 6), Full dose AMA-1/ASO2A: blue (n = 10)). All data points represent pretreatment parasitemias.

Figure 6 shows the data points and the fit of parasitemia of the three groups. The estimated growth rates with 95% CIs of the AMA-1/AS01B group (14.5, 9.8–21.6) and AMA-1/AS02A group (13.9, 8.6–23.5) were slightly lower but not statistically different from the control group (16.8, 10.5–26.8). However, the number of infected hepatocytes and/or the first wave of RBC invasion of the AMA-1/AS02A group (24.6 ± 33.0) was significantly lower (p = 0.044, t-test) as compared to the control group (171 ± 135). AMA-1/AS01B also showed a lower first wave (73.6 ± 68.7), but this difference was not significant (p = 0.146, t-test).

#### Efficacy by immunological endpoint

We did examine the relationships between immunological endpoints and prepatent periods as determined by light microscopy and by qPCR for each challenged volunteer. No correlation was demonstrated between prepatent period and ELISA titer, percent inhibition by serum GIA, or cellular IFN-γ production by ELISPOT (data not shown). In further post-hoc analyses, the 4 volunteers with the longest prepatent periods by qPCR did not have significant differences in these endpoint assays as compared with the remaining volunteers. In addition, the immunogenicity results obtained by ELISA, GIA and ELISPOT were not significantly different between those volunteers that underwent challenge and those who did not (data not shown).

## Discussion

In this paper, we describe the first safety, immunogenicity and efficacy trial for a recombinant AMA-1 antigen formulated with either AS01B or AS02A.

### Interpretation

All three vaccine candidates, low dose AMA-1/AS01B, full dose AMA-1/AS01B and full dose AMA-1/AS02A, had a good tolerability profile but caused moderate reactogenicity, manifested specifically by local erythema and swelling. There was no difference in frequency or pattern of local adverse events among the three vaccine groups; although it did appear that the volunteers administered the full dose AMA-1/AS01B experienced slightly more systemic adverse events. For all three groups, there was an increase in frequency and intensity of adverse events with successive immunizations, yet the adverse events did not contribute to volunteer attrition as evidenced by completion of 103 of 105 scheduled vaccinations in 35 subjects. The frequency of adverse events described here is greater (for all three groups) than that described in recent trial of AMA-1/AS02A [16], and RTS,S in AS02A [28], [32], [33] or AS01B (personal communication, K. Kester) in malaria-naïve adults at WRAIR; however, Grade 3 swelling, accompanied by little or no pain, similar to that seen in this study, was consistently reported in Malian adults [17] and children vaccinated with AMA-1/AS02A (personal communication C. Plowe). The reason for the increased local and systemic reactogenicity in this study is unclear but may be due to an intrinsic immunologic property of adjuvanted recombinant AMA-1. It is likely not due to endotoxin or residual bacterial host contaminant given the strict cGMP standards of AMA-1 and Adjuvant Systems manufacture. A recent Phase 1a study of *Pichia pastoris*-expressed AMA1 formulated in AS02A also had elevated frequency of mild to moderate pain, erythema and systemic adverse events reported [34].

The unexpected adverse event seen in one volunteer was considered related to the vaccine given its consistent localization over both injection sites. Based on the timing of rash onset, this reaction could have represented either a Type IV hypersensitivity reaction or possibly antigen-antibody complex formation without accompanying systemic symptoms of serum sickness [35]. When evaluated, the anti-AMA-1 antibody levels and IFN-γ ELISPOT responses in this individual were not markedly elevated or depressed as compared to other volunteers in the study.

Immunization with all three vaccine formulations produced very high antibody titers in the majority of volunteers, with no statistically significant difference in GMCs among vaccine groups. Our findings are consistent with preclinical data that malarial recombinant protein antigens elicit equivalent (or greater) antibodies when formulated with AS01B versus AS02A [18]–[20] and Phase 1a vaccine studies in which antibody titers did not differ by the dose of AMA-1 formulated in AS02A [16], [34]. The third immunization did provide a boost to falling antibody titers, although the peak level measured was not significantly higher than the peak achieved post-second immunization, a finding also seen in the recent Phase 1a study of AMA-1/AS02A at WRAIR [16]. This finding raises the question of whether an effective immunization regimen can be limited to two immunizations, or alternatively, how the time interval between second and third immunization could be optimized.

The growth inhibition of homologous 3D7 *P. falciparum* parasites was the highest yet reported for any immune serum or purified antibody GIA assessed in Phase 1a trials of blood stage malaria vaccines [16], [34], [36]–[39]. Although the vaccine did induce a humoral response demonstrating *in vitro* GIA activity, the absence of protective efficacy suggests that GIA may not serve as an accurate correlate of protection. In addition, the inhibitory responses induced by this allele of AMA-1 were highly strain-specific, yielding negligible inhibitory activity against heterologous FVO parasites similar to findings observed in other studies, both preclinical [8] and clinical [16], [27].

All three vaccine candidates induced cellular responses as measured by IFN-γ production. Interestingly, the volunteers receiving the 10 µg dose of AMA-1/AS01B had significantly higher responses to recombinant protein antigen than the volunteers receiving the 50 µg dose of AMA-1 in either Adjuvant System. This phenomenon of a greater T-cell response with a reduced antigen dose (as compared to full dose) but constant amount of adjuvant was also seen in a recent trial of Liver Stage Antigen-1 (LSA-1) (*manuscript in press*, J. Cummings) as well as in the Phase 1b study of AMA-1/AS02A in Malian adults (*manuscript in revision,* K. Lyke). It has been reported in Phase 2a challenge trials of RTS,S/AS02A [32], [40], RTS,S/AS01B (personal communication, K. Kester) and heterologous prime-boost immunization regimens with antigen based on thrombospondin-related adhesion protein (TRAP) [41], [42] that elevated antigen-specific IFN-γ responses are associated with a delay in the prepatent period and protection against parasitemia. However, this association has yet to be demonstrated in a malaria-experienced population. In addition there may be a cellular versus humoral tradeoff: in a recent trial in Malian adults, there was a trend towards greater antibody responses in subjects who received 50 µg AMA-1 formulated in AS02A as compared to 25 µg AMA-1/AS02A [17].

This was the first clinical trial of any AMA-1-based vaccine using the rigorous sporozoite challenge model in order to assess efficacy. The challenge model traditionally used to assess efficacy of pre-erythrocytic vaccine candidates is robust: at WRAIR, a 100% infection rate has been obtained in the last 100 infectivity control volunteers fed upon by 5 *Anopheline* mosquitoes which were each highly infected with *P. falciparum* sporozoites. In addition, the clinical protocol under which the subjects are challenged requires malaria treatment without delay upon diagnosis of parasitemia by light microscopy, thus precluding subsequent direct observation of any parasitemia-limiting effects or abrogation of clinical manifestations that may be induced by a blood stage vaccine. Under these stringent conditions, there was no prevention of parasitemia or delay in the onset of parasitemia by light microscopy. A sensitive, quantitative PCR analysis also did not reveal any statistically significant delay in the time to parasitemia in vaccinees versus infectivity controls.

We conducted exploratory post hoc analyses to detect more subtle signs of a vaccine effect that we hypothesized could be reflected by a reduction or impairment of released hepatic merozoites and/or a reduction in parasite growth rate in the blood stage. Analysis showed that the first peak (representing the first wave of RBC invasion and/or the number of infected hepatocytes per volunteer) in the AMA-1/AS02A vaccinees was about 7 times lower compared to the control group (figure 6). AMA-1/AS01B showed a mean reduction of about 50%, but this was not significant. Unfortunately, due to the limited number of qPCR data points restricted by the once daily measurements, the power of the statistical model is reduced. The inhibitory antibodies induced by AMA-1/AS02A may act at 2 different levels: reduction of sporozoite invasion into the hepatocytes as observed *in-vitro* [7] and/or a reduction of the first wave of liver merozoites invading new red blood cells. The estimated mean reduction of 17% in growth rate is too small to effectively reduce parasite multiplication. It has been estimated that *in vivo* growth inhibition needs to be at least 70% to realize a decrease in parasitemia [31].

With the current challenge model and need to treat individuals upon first detection of parasitemia, it is not possible to ascertain the potential benefit or duration of this observed reduction in parasite burden. Additional post hoc analyses comparing vaccinees with prepatent periods revealed no correlation with antibody titer by ELISA, growth inhibition by GIA activity, or a difference in their IFN-γ production by ELISPOT.

### Generalizability

While the three vaccine candidates induced high concentrations of anti-AMA-1 antibodies which exhibited functional activity, albeit only against a homologous allele, it remains unclear if and at what level a monovalent 3D7-based AMA-1 vaccine will stimulate cross-reactive antibodies in malaria-experienced populations. The mean percent inhibition by GIA seen in Malian adults vaccinated with AMA-1/AS02A was greater against both 3D7 and FVO parasites than control vaccinees [17]. Nonetheless, a successful AMA-1-based vaccination strategy may need to include more than one AMA-1 allele to be effective in populations continually exposed to malaria infection [43]. Studies of AMA-1 diversity over three years in Bandiagara, Mali are finding extreme diversity, with no single haplotype having a prevalence of more than 4%. The 3D7 allele represents one of the most common AMA-1 haplotypes, and no parasites carry the full FVO haplotype in this setting (S. Takala, personal communication). Testing of AMA-1/AS02A in a pediatric Phase 2b, placebo-controlled trial in Bandiagara, Mali, an epidemiologic setting of substantial AMA-1 allelic diversity, is currently ongoing (www.clinicaltrials.gov NCT00460525). In addition to the primary efficacy endpoint of clinical malaria, planned ancillary analyses will explore both parasitologic and allele-specific effects, thus evaluating the potential for a 3D7 AMA-1-based vaccine to induce a cross-protective immune response in a malaria-experienced population, as well as the possibility that ongoing natural exposure will boost heterologous allele-specific responses.

Our AMA-1-based malaria vaccine development strategy requires that two sequential milestones be accomplished prior to incorporation of one or more AMA-1 antigens into a multi-stage, multi-component vaccine [22]. First, an AMA-1 vaccine must confer significant clinical benefit in either a Phase 2a malaria challenge or in an endemic population. Second, the AMA-1 vaccine must be sufficiently active against diverse AMA-1 alleles such that the risk of allelic escape is very low. In anticipation of the potential requirement for inclusion of additional AMA-1 alleles in a multi-antigen vaccine, we have produced GMP AMA-1 representing the FVO allele and anticipate potentially combining the two FVO and 3D7 antigens in a vaccine. Allelic exchange experiments have recently defined specific clusters of polymorphic amino acid residues involved in antigenic escape in vitro [43], and studies of the within-host dynamics of blood stage antigens offer another strategy for identifying specific residues associated with allele-specific clinical immunity [44]. Current research efforts at WRAIR seek to identify a consensus AMA-1 molecule/chimera that would elicit a broad immunity active against multiple *P. falciparum* AMA-1 phenotypes [43].

### Overall Evidence

Since AMA-1 is expressed on pre-erythrocytic and erythrocytic stages of the parasite, it is a promising vaccine antigen to induce malarial protection by targeting both the humoral and cell-mediated arms of the immune system. Both types of immune responses have been demonstrated in this study, in three other vaccine trials with AMA-1/AS02A [16], [17], [34] as well as an AMA-1 protein antigen formulated in alum [27] and Montanide ISA 720 [34]. This study was the first to test the efficacy of AMA-1; subjected to a stringent test of efficacy that required treatment at time of proven patency, no vaccine formulation prevented infection or delayed patency, but one formulation, AMA-1/AS02A, did suggest a significant reduction in parasite burden as determined by PCR analysis. Current field trials now underway in Bandiagara, Mali should provide additional data to potentially determine if the immunogenicity results seen will translate into significant clinical effect in a population living in a malaria endemic area with a diverse parasite population.

That AMA-1 continues to be thought of as a promising vaccine antigen is reflected in its recent testing in an adenovector-based platform (personal communication Thomas Richie) and in virosomes [45]. With the new goal of malaria eradication set forth by World Health Organization and the upcoming large scale pediatric Phase 3 study of RTS,S in subSaharan Africa, an effective second generation malaria vaccine is critical. Based on the present trial's evidence of strong immunogenicity of AMA-1/AS01B and AS02A and the encouraging suggestion of biological effect, we believe AMA-1 remains a viable vaccine candidate. Future studies will help elucidate whether modification or addition of allelic forms and/or choice of delivery platform may sufficiently enhance its immunogenicity and efficacy to protect the most vulnerable populations.

## Supporting Information

Protocol S1 Trial protocol. Phase I/IIa double-blind, randomized study of the safety, immunogenicity and preliminary efficacy after primary sporozoite challenge of FMP2.1/AS01B and FMP2.1/AS02A candidate malaria vaccines
(1.87 MB DOC)

Checklist S1 CONSORT checklist
(0.06 MB DOC)
