# Supplementary material for: Phase 1/2a Study of the Malaria Vaccine Candidate Apical Membrane Antigen-1 (AMA-1) Administered in Adjuvant System AS01B or AS02A
Source: PLoS One. 2009 Apr 23;4(4):e5254. doi: 10.1371/journal.pone.0005254 (PMC2669163; doi:10.1371/journal.pone.0005254)
Supplement: Protocol S1 — Trial protocol. Phase I/IIa double-blind, randomized study of the safety, immunogenicity and preliminary efficacy after primary sporozoite challenge of FMP2.1/AS01B and FMP2.1/AS02A candidate malaria vaccines (1.87 MB DOC) [file pone.0005254.s001.doc]

**Phase I/IIa double-blind, randomized study of the safety, immunogenicity and preliminary efficacy after primary sporozoite challenge of FMP2.1/AS01B and FMP2.1/AS02A candidate malaria vaccines administered intramuscularly at months 0, 1, and 2 in healthy malaria-naive adults living in the United States**

**TITLE:** Phase I/IIa double-blind, randomized study of the safety, immunogenicity and preliminary efficacy after primary sporozoite challenge of FMP2.1/AS01B and FMP2.1/AS02A candidate malaria vaccines administered intramuscularly at months 0, 1, and 2 in healthy malaria-naive adults living in the United States

**Trial Site:** Clinical Trials Center (CTC)

Walter Reed Army Institute of Research (WRAIR)

503 Robert Grant Avenue

Silver Spring, MD 20910

Tel: 301-319-9660

Fax: 301-319-9585

**IND No.:** FMP2.1/AS02A: BB-IND 11140

FMP2.1/AS01B: BB-IND 13089

**IND Sponsor:** US Army Surgeon General

**Sponsor’s Representative:** United States Army Medical Materiel Development Activity (USAMMDA)

**Protocol No.:** WRAIR #1280

HSRRB #A-13949

ETrack Protocol No. 104936 (MAL-045 GSK identifier)

MVI IRB #20060900

**Protocol History:** Version 1.0, March 10, 2006

Version 2.0, April 28, 2006

Version 3.0, May 22, 2006

Version 4.0, June 15, 2006

Version 5.0, August 02, 2006

Version 6.0, September 05, 2006

Version 7.0, November 01, 2006

Version 8.0, December 13, 2006

Version 9.0, March 29, 2007

**Principal Investigator:**

Michele D. Spring, M.D**.** Department of Immunology

Division of Communicable Diseases and Immunology

Walter Reed Army Institute of Research

503 Robert Grant Avenue

Silver Spring, MD 20910

Tel: 301-319-9307

Fax: 301-319-7358

Email: michele.spring@amedd.army.mil

**Principal Investigator Agreement**

I, the undersigned, have reviewed this protocol, *“Phase I/IIa double-blind, randomized study of the safety, immunogenicity and preliminary efficacy after primary sporozoite challenge of FMP2.1/AS01B and FMP2.1/AS02A candidate malaria vaccines administered intramuscularly at months 0, 1, and 2 in healthy malaria-naive adults living in the United States”*, including Appendices, and I will conduct the clinical study as described and will adhere to the Ethical and Regulatory Considerations stated. I have read and understood the contents of the Investigator Brochure.

Signature Date

Michele D. Spring, MD

**Synopsis**

**Title:**

Phase I/IIa double-blind, randomized study of the safety, immunogenicity and preliminary efficacy after primary sporozoite challenge of FMP2.1/AS01B and FMP2.1/AS02A candidate malaria vaccines administered intramuscularly at months 0, 1, and 2 in healthy malaria-naive adults living in the United States

**Participants:**

Up to 41 healthy malaria-naïve adults aged 18-50 years living in the United States: 35 vaccinees and 6 infectivity controls

**Study vaccine:**

FMP2.1, recombinant protein representing 3D7 variant of apical membrane antigen-1 (AMA-1) of *Plasmodium falciparum* formulated with GlaxoSmithKline Biologicals proprietary adjuvants AS01B or AS02A.

**Objectives**

**Primary**

- To assess the safety and reactogenicity of candidate malaria vaccines FMP2.1/AS01B and FMP2.1/AS02A when administered intramuscularly as 3 immunizations on a 0-, 1- and 2- months schedule to malaria-naïve adult volunteers living in the United States.

**Secondary**

- To assess the humoral immune responses by ELISA to candidate malaria vaccines FMP2.1/AS01B and FMP2.1/AS02A.
- To assess functionality of anti-FMP2.1 antibodies induced by both vaccine candidates FMP2.1/AS01B and FMP2.1/AS02A to inhibit the growth of asexual parasites as measured by standardized GIA.
- To assess the efficacy of FMP2.1/AS01B and FMP2.1/AS02A against sporozoite challenge with *P. falciparum*

**Tertiary**

- To assess the cell-mediated immune responses to candidate FMP2.1/AS01B and FMP2.1/AS02A malaria vaccines

**Study design**

Phase I/IIa randomized, double blind vaccine trial

Single site: Center for Clinical Trials, WRAIR

Up to 35 malaria-naive adults will be enrolled as vaccinees

Cohort 1

5 volunteers to receive 10 µg FMP2.1 in 0.5 mL AS01B intramuscularly at 0-, 1-, and 2-months

Cohort 2

- Group A: 15 volunteers to receive 50 µg FMP2.1 in 0.5 mL AS01B

intramuscularly at 0-, 1-, and 2-months

- Group B: 15 volunteers to receive 50 µg FMP2.1in 0.5 mL AS02A

intramuscularly at 0-, 1-, and 2-months

There will be at least a 2-week stagger between Cohort 1 and Cohort 2 to allow for evaluation of vaccine safety and reactogenicity of Cohort 1 prior to proceeding with immunization of Cohort 2

Investigators will enroll a minimum of 6 and a maximum of 15 volunteers in Groups A and B

A minimum of 6 and maximum of 10 of the initial 15 volunteers from each group in Cohort 2 only will go on to primary sporozoite challenge 14-30 days after third immunization

6 infectivity controls will undergo primary sporozoite challenge in parallel with vaccinees (3 alternates will be recruited to be available for challenge if needed)

**Endpoints**

**Primary endpoints**

- Occurrence of solicited symptoms over a seven day follow-up period (day of immunization and six subsequent days) after each immunization
- Occurrence of unsolicited symptoms over a 30 day follow-up period (day of immunization and 29 subsequent days) after each immunization
- Occurrence of serious adverse events during the study period

**Secondary endpoints**

- Anti-AMA-1 antibody titers at specified time points during Immunization Phase for Cohorts 1 & 2 (Days 0, 14, 28, 42, 56, 70),and Challenge Phase for Cohort 2 (Day of Challenge (Month 2), Day 7 post-challenge, approximately Day 30 post-challenge (Week I or II), Day 90 post-challenge (Month 5))
- Functionality of anti-AMA-1 antibodies as percent parasite growth inhibition as measured by standardized homologous and heterologous GIA at specified time points during the Immunization Phase for Cohorts 1 & 2 (Days 0, 14, 28, 42, 56, 70),and Challenge Phase for Cohort 2 (Day of Challenge (Month 2), Day 7 post-challenge, approximately Day 30 post-challenge (Week I or II), Day 90 post-challenge (Month 5))
- Development of parasitemia and time to parasitemia after primary challenge following administration of the FMP2.1/AS01B and FMP2.1/AS02A candidate vaccines (50 µg/0.5 mL dose formulations only)

**Tertiary endpoints**

- Cell-mediated immune responses at time points at which blood samples are taken:
- IFNγ determination by the ELISPOT assay
- Intracellular Cytokine staining (ICS) characterizing CD4/CD8 T cells expressing IFNγ and/or TNFα and/or IL-2.

Table of Contents

1 Introduction [15](#__RefHeading___Toc124070157)

1.1 Medical application and status [15](#__RefHeading___Toc124070158)

1.2 Life cycle of the malaria parasite [15](#__RefHeading___Toc124070159)

1.3 Rationale for Antigen Selection [16](#__RefHeading___Toc124070160)

1.4 Rationale for this study [18](#__RefHeading___Toc124070161)

2 Objectives [18](#__RefHeading___Toc124070162)

2.1 Primary objective [24](#__RefHeading___Toc124070163)

2.2 Secondary objectives [24](#__RefHeading___Toc124070164)

2.3 Tertiary objectives [24](#__RefHeading___Toc124070165)

3 Study Design Overview [24](#__RefHeading___Toc124070166)

4 Study Cohort [25](#__RefHeading___Toc124070167)

4.1 Number of participants / centers [25](#__RefHeading___Toc124070168)

4.2 Inclusion criteria [26](#__RefHeading___Toc124070169)

4.3 Exclusion criteria for enrollment [28](#__RefHeading___Toc124070170)

4.4 Elimination criteria during the study [30](#__RefHeading___Toc124070171)

4.5 Contraindications to immunization [31](#__RefHeading___Toc124070172)

5 Conduct of Study [31](#__RefHeading___Toc124070173)

5.1 Ethics and regulatory considerations [31](#__RefHeading___Toc124070174)

5.1.1 Institutional Review Board/Independent Ethics Committee (IRB/IEC) [32](#__RefHeading___Toc124070175)

5.1.2 Informed consent [34](#__RefHeading___Toc124070176)

5.2 General study aspects [36](#__RefHeading___Toc124070177)

5.2.1 Screening [36](#__RefHeading___Toc124070178)

5.2.2 Study Design [38](#__RefHeading___Toc124070179)

5.2.3 Safety considerations during the trial [38](#__RefHeading___Toc124070180)

5.2.4 Risks to the Participants [42](#__RefHeading___Toc124070181)

5.2.4.1 Risks associated with immunization [42](#__RefHeading___Toc124070182)

5.2.4.2 Risks associated with malaria challenge [42](#__RefHeading___Toc124070183)

5.2.5 Precautions to Minimize Risk [43](#__RefHeading___Toc124070184)

5.2.5.1 Immunization [43](#__RefHeading___Toc124070185)

5.2.6 Assessment of Vaccine Efficacy [43](#__RefHeading___Toc124070186)

5.2.6.1 Malaria Challenge [43](#__RefHeading___Toc124070187)

5.2.6.2 Parasite and mosquito strains and infection of mosquitoes [44](#__RefHeading___Toc124070188)

5.2.6.3 Infection of Human Volunteers [45](#__RefHeading___Toc124070189)

5.2.6.4 Management of Infected Human Volunteers [46](#__RefHeading___Toc124070190)

5.3 Outline of study procedures [49](#__RefHeading___Toc124070191)

5.4 Detailed description of study phases/visits [50](#__RefHeading___Toc124070192)

5.4.1 Screening and Immunization phase [50](#__RefHeading___Toc124070193)

5.4.2 Challenge phase [58](#__RefHeading___Toc124070194)

5.5 Sample handling and analysis [64](#__RefHeading___Toc124070195)

5.5.1 Treatment and storage of biological samples [65](#__RefHeading___Toc124070196)

5.5.2 Laboratory assays [65](#__RefHeading___Toc124070197)

5.5.3 Rationale for exploratory laboratory analyses [68](#__RefHeading___Toc124070198)

6 Study Vaccines and Administration [73](#__RefHeading___Toc124070199)

6.1 Study vaccines [73](#__RefHeading___Toc124070200)

6.2 Dosage and administration [73](#__RefHeading___Toc124070201)

6.3 Storage [74](#__RefHeading___Toc124070202)

6.4 Treatment allocation and randomization [75](#__RefHeading___Toc124070203)

6.5 Method of blinding and breaking the study blind [76](#__RefHeading___Toc124070204)

6.6 Replacement of unusable vaccine doses [77](#__RefHeading___Toc124070205)

6.7 Packaging [77](#__RefHeading___Toc124070206)

6.8 Vaccine accountability [77](#__RefHeading___Toc124070207)

6.9 Concomitant medication/treatment [78](#__RefHeading___Toc124070208)

7 Health Economics [78](#__RefHeading___Toc124070209)

8 Adverse Events [78](#__RefHeading___Toc124070210)

8.1 Eliciting and documenting adverse events [78](#__RefHeading___Toc124070211)

8.1.1 Solicited adverse events [82](#__RefHeading___Toc124070212)

8.1.2 Unsolicited adverse events [82](#__RefHeading___Toc124070213)

8.2 Assessment of intensity [83](#__RefHeading___Toc124070214)

8.3 Assessment of causality [86](#__RefHeading___Toc124070215)

8.4 Following-up of adverse events and assessment of outcome [87](#__RefHeading___Toc124070216)

8.5 Serious adverse events [88](#__RefHeading___Toc124070217)

8.5.1 Definition of a serious adverse event [88](#__RefHeading___Toc124070218)

8.5.2 Reporting serious adverse events [89](#__RefHeading___Toc124070219)

8.6 Pregnancy [93](#__RefHeading___Toc124070220)

8.7 Treatment of adverse events [93](#__RefHeading___Toc124070221)

9 Participant Completion and Withdrawal [94](#__RefHeading___Toc124070222)

9.1 Participant completion [94](#__RefHeading___Toc124070223)

9.2 Participant withdrawal [94](#__RefHeading___Toc124070224)

9.2.1 Participant withdrawal from the study [94](#__RefHeading___Toc124070225)

9.2.2 Participant withdrawal from investigational product [95](#__RefHeading___Toc124070226)

10 Data Evaluation: criteria for evaluation of objectives [95](#__RefHeading___Toc124070227)

10.1 Study Objectives [96](#__RefHeading___Toc124070228)

10.2 Co-Primary endpoints [96](#__RefHeading___Toc124070229)

10.3 Secondary endpoints [96](#__RefHeading___Toc124070230)

10.4 Tertiary endpoints [97](#__RefHeading___Toc124070231)

10.5 Study cohorts/data sets to be evaluated [97](#__RefHeading___Toc124070232)

10.6 Estimated sample size [98](#__RefHeading___Toc124070233)

10.6.1 Sample Size Justification [98](#__RefHeading___Toc124070234)

10.6.2 Reactogenicity [98](#__RefHeading___Toc124070235)

10.7 Final analyses [99](#__RefHeading___Toc124070236)

10.7.1 Analysis of demographics [99](#__RefHeading___Toc124070237)

10.7.2 Analysis of safety [99](#__RefHeading___Toc124070238)

10.7.3 Analysis of immunogenicity [100](#__RefHeading___Toc124070239)

10.7.4 Analysis of efficacy [101](#__RefHeading___Toc124070240)

10.7.5 Exploratory analyses [102](#__RefHeading___Toc124070241)

10.8 Final analysis [102](#__RefHeading___Toc124070242)

11 Administrative Matters [102](#__RefHeading___Toc124070243)

12 References [103](#__RefHeading___Toc124070244)

# Tables

Table 1: Procedures of immunization phase of study …………………………..56

Table 2: Procedures during challenge phase of study …...……………….…...64

Table 3: Overview of serological assays 66

Table 4: Solicited local and general adverse events 82

Table 5: Intensity grading of local solicited adverse events. 83

Table 6: Intensity grading of general solicited adverse events 84

# Appendices

Appendix A: World Medical Association Declaration of Helsinki

Appendix B: Administrative Matters

Appendix C: Overview of the recruitment plan

Appendix D: Laboratory Assays

Appendix E: Vaccine Supplies, Packaging and Accountability

Appendix F: Volunteer Agreement Affidavit (Cohort 1Vaccinees)

Appendix G: Volunteer Agreement Affidavit (Vaccinees)

Appendix H: Volunteer Agreement Affidavit (Controls)

Appendix I: CONSENT FOR HIV ANTIBODY BLOOD TEST

Appendix J: BLOOD DONATION CONSENT FORM

Appendix K : Informed Consent Comprehension Assessment (Cohort 1 Vaccinees )

Appendix L: Informed Consent Comprehension Assessment (COHORT 2 Vaccinees )

Appendix M: Informed Consent Comprehension Assessment (Controls )

Appendix N: POSITIVE MALARIA BLOOD SMEAR REPORT

Appendix O: List of investigators and their roles and responsibilities.

Appendix P: PERSONNEL

Appendix Q: Amendments/Modifications to the Study Protocol

# List of abbreviations

3-D-MPL 3-deacylated Monophosphoryl Lipid A

AE Adverse event

ALT Alanine aminotransferase

AMA-1 Apical Membrane Antigen-1

AP Alkaline phosphatase

AS01B Adjuvant system 1 containing MPL, QS21 and liposomes,

AS02A Adjuvant system 2 containing MPL, QS21 and oil/water emulsion

AST Aspartate aminotransferase

BUN Blood urea nitrogen

CBC Complete blood count

CFSE 5- (or 6-) carboxyfluorescein diacetate succinimidyl ester

CK Creatinine kinase

CMI Cell mediated immunity

CRF Case Report Form

CTL Cytotoxic T Lymphocytes

ELISA Enzyme linked immunosorbent assay

Elispot Enzyme linked immune-spot assay

ERC Ethical Review Committee

FMP Falciparum malaria protein

FMP2.1 Vaccine antigen derived from 3D7 variant of AMA-1

GIA Growth Inhibition Assay

GMT Geometric Mean Titer

GSK GlaxoSmithKline Biologicals

HBsAg Hepatitis B surface antigen

HBV Hepatitis B virus

HCV Hepatitis C virus

HIV Human immunodeficiency virus

hpf High power fields

HSRRB Human Subjects Research Review Board

HURC Human Use Review Committee

ICS Intracellular Cytokine Staining

IEC Independent Ethics Committee

IFA Immunofluorescence assay

IFNγ Interferon gamma

IgG Immunoglobulin G

IgM Immunoglobulin M

IL 2, 4, 10 Interleukin 2, 4, 10

IM Intramuscular

IRB Institutional Review Board

IU/l International units per liter

LDH Lactate dehydrogenase

MAL GSK designation for approved malaria vaccine trial

mL milliliter

mmol/l Millimole per liter

MVDU Malaria Vaccine Development Unit

MVI Malaria Vaccine Initiative

NIH National Institutes of Health

ORP Office of Research Protections

*P. chabaudi* *Plasmodium chabaudi*

*P. falciparum* *Plasmodium falciparum*

*P. yoelii* *Plasmodium yoelii*

PATH Program for Appropriate Technology in Health

PBMC Peripheral blood mononuclear cells

PCR Polymerase chain reaction

PCV Packed cell volume (hematocrit values)

PFS Prefilled syringe

PI Principal Investigator

PLDH Parasite Lactate dehydrogenase

PRBC Parasitized red blood cells

QS-21 *Quillaja saponaria* 21 (saponin derivative)

SAE Serious adverse event

SMC Safety Monitoring Committee

SOP Standard Operating Procedure

TNFα Tumor Necrosis Factor alpha

USAID United States Agency for International Development

USAMMDA US Army Medical Material Development Activity

VE Vaccine efficacy

WHO World Health Organization

WRAIR Walter Reed Army Institute of Research

WRAMC Walter Reed Army Medical Center

# Glossary of terms

| **Participant(s):** | Term used throughout the protocol to denote the enrolled individual(s). |
| --- | --- |
| **Medical Monitor:** | An individual medically qualified to assure the responsibilities of the sponsor especially as regards the ethics, clinical safety of a study and the assessment of adverse events. The medical monitor is required to review all unanticipated problems involving risk to volunteers and others, serious adverse events and all volunteer deaths associated with the protocol and provide an unbiased written report of the event. At a minimum, the medical monitor should comment on the outcomes of the event or problem and in the case of a serious adverse event or death, comment on the relationship to participation in the study. The medical monitor should also indicate whether he/she concurs with the details of the report provided by the study investigator. Reports for events determined by either the investigator or medical monitor to be possibly or definitely related to the participation and reports of events resulting in death should be promptly forwarded to the HURC, HSRRB and Western IRB. |
| **Study Monitor:** | An individual assigned by and centrally located at USAMMDA who is responsible for assuring proper conduct of a clinical study. |
| **Delay in Patency** | In this study, the measure of vaccine efficacy defined as *P. falciparum* asexual parasitemia > 0 or a delay in patency of infection >2 days versus mean prepatent period in unimmunized infectivity controls |
| **Eligible:** | Qualified for enrollment into the study based upon strict adherence to inclusion/exclusion criteria. |
|  |  |
| **Evaluable:** | Meeting all eligibility criteria, complying with the procedures defined in the protocol, and, therefore, included in analysis (see Section 10.5 for details on criteria for evaluability). |
| **Primary challenge:** | Homologous sporozoite challenge that occurs 14 to 30 days after the last immunization |
| **Protocol amendment:** | Any change in a clinical protocol that affects the safety of participants, the scope, design, assessments or scientific validity of the clinical investigation, e.g., dose change, duration of treatment, number of participants, control group(s), the assessments. |
| **Protection from challenge** | Failure to develop *P. falciparum* asexual stage parasitemia in the absence of anti-malarial chemoprophylaxis for a period of 30 days following experimental *P. falciparum* sporozoite challenge |

# Introduction

## Medical application and status

Malaria is one of the leading causes of morbidity and mortality in the developing world with an estimated 300 and 500 million clinical cases each year resulting in 1.5 to 2.7 million deaths^1,2^. Of the four protozoan species of *Plasmodium* causing human malaria, *Plasmodium falciparum* causes the majority of severe disease and death. Problems such as increasing parasite resistance to anti-malarial drugs and poor access to adequate health care contribute to overall burden of disease. The populations most affected by malaria are children less than five years of age living in endemic areas or naïve populations such as unexposed tourists or armed services personnel traveling to endemic areas who have not developed an immune response effective at controlling the infection. A malaria vaccine is critical to abrogating the effects of this disease in these vulnerable populations.

## Life cycle of the malaria parasite

The life cycle of the malaria parasite requires the bite of an infected female *Anopheles* mosquito vector to transmit the sporozoite, or exo-erythrocytic form, of *Plasmodium* *spp*. to humans. These sporozoites travel through the bloodstream and ultimately invade liver cells where they multiply asexually, maturing in five to seven days for *P. falciparum*, subsequently releasing thousands of merozoites into the bloodstream. The free merozoite invades erythrocytes and undergoes asexual maturation and multiplication, ultimately rupturing the erythrocyte, releasing new merozoites. This cycle of erythrocyte invasion and rupture continues and is responsible for some of the clinical signs and symptoms of malaria such as fever, chills, as well as (potentially severe) anemia. A small number of the invading merozoites do not multiply, but instead differentiate into sexual forms known as gametocytes. When ingested by a mosquito, male and female gametocytes can unite to form a zygote, which then matures to release sporozoites that subsequently migrate to the mosquito salivary glands. These sporozoites are transmitted to the next person bitten by the mosquito, thereby completing the life cycle.

## Rationale for Antigen Selection

The Walter Reed Army Institute of Research (WRAIR) has been focusing research efforts on a malaria vaccine that targets antigens from both the pre-erythrocytic and erythrocytic stages of *Plasmodium falciparum*. A vaccine targeting the liver stage sporozoite would theoretically prevent merozoite release from the liver thereby preventing clinical disease. An effective erythrocytic vaccine would direct the immune response against merozoite antigens in order to lessen the severity of clinical signs and symptoms of malaria caused by repeated cycles of merozoite invasion and subsequent rupture of erythrocytes. The definitive malaria vaccine will most likely need to be composed of antigens from both stages, and immunological studies support the need for both a strong humoral immune response as well as an effective cell-mediated immune response in order to induce a sufficient level of protective immunity^3^.

Apical Membrane Antigen-1 is an 83-kDa precursor protein concentrated in micronemes at the apical end of the merozoite^4^. AMA-1 has also been found on the surface of sporozoites and on hepatic merozoites^5^, thus this protein may provide both pre-erythrocytic and erythrocytic targets, engaging host humoral and cellular immune responses. The 83-kDa protein is comprised of an N-terminus prosequence, an ectodomain with three named sub-domains (I, II, II), and a carboxy-terminus transmembrane section and cytoplasmic tail^6^. The prosequence is proteolytically cleaved to form a 66-kDa protein which then translocates from micronemes to the surface of the merozoite^7^. This surface protein is thought to mediate merozoite reorientation to the erythrocyte^8^. Further proteolytic cleavage occurs at the merozoite surface producing two soluble fragments: a 44-kDa molecule and a 48-kDa molecule. Antibodies to AMA-1 that inhibit invasion have also been shown to prevent proteolytic processing and surface redistribution of the protein and suggest inhibition of red blood cell invasion^9^.

AMA-1 structure, function and role in infection have been evaluated *in vitro*, in animal studies and in human populations. Results from these studies strongly support its malaria vaccine candidacy.

*In vitro* studies

Monoclonal antibodies to AMA-1 (and their corresponding Fab fragments) have been shown to inhibit erythrocyte invasion *in* *vitro*^10^. Research at WRAIR has demonstrated antibodies that prevent erythrocyte invasion do so by inhibiting proteolytic processing of 66 kDa portion of AMA-1^9^. Recently, identification of a putative erythrocyte ligand has been published^11^. The amino acid sequence of AMA-1 is largely conserved although different variants/alleles do circulate in populations and antibody responses have been shown to be influenced by allelic-specificity. Two studies have demonstrated that immunization of rabbits with a 3D7 AMA-1 allele generates antibodies capable of inhibiting growth of homologous parasite, with reduced ability to do so for heterologous (HB3 and FVO strains) parasites^12,13^.

Preclinical studies

Two separate studies in BALB/c mice both demonstrated that immunization with recombinant AMA-1 protected against challenge with either *P. chabaudi or P. yoelii*^14,15^. Passive transfer experiments show anti-AMA IgG from rabbits immunized with AMA-1 can protect mice against *P. chabaudi* challenge^14^. Further elucidating the immune responses to AMA-1, Xu et al. immunized mice with *P. chabaudi* AMA-1 recombinant protein^16^. B-cell knockout mice were unable to control infection upon homologous challenge, reiterating the importance of humoral immunity. In addition, immunized BALB/c mice that were subsequently CD4+ depleted were also unable to control infection upon challenge, notably without a decline in antibody levels, suggesting there is also an antibody-independent cell-mediated mechanism of protection induced by AMA-1. Immunization of rabbits with the AMA-1/E protein (prior version of FMP2.1) induced strong inhibitory antibodies when tested in Growth Inhibitory Assay (GIA) at WRAIR^17^. Protection against *P. knowlesi* has been shown in rhesus monkeys immunized with affinity-purified AMA-1^18^ and, *Aotus* monkeys immunized with recombinant *P. falciparum* AMA-1 (FVO variant) were protected when challenged with homologous parasites^19^.

Human populations

Hodder et al. have shown affinity-purified AMA-1 antibodies from adults in malaria-endemic Papua New Guinea can inhibit erythrocyte invasion^12^. A study of a naturally-exposed population in coastal Kenya demonstrated that increased levels of AMA-1 antibodies correlated with protection in parasitemic adults^20^. In a holoendemic area in Western Kenya, T-cell studies have shown proliferative responses in adults to peptides derived from ectodomain of AMA-1^21^, and a longitudinal study revealed stability of antibody levels to AMA-1 over time^22^. Naïve adult volunteers immunized with irradiated *P. falciparum* sporozoites demonstrated T-cell responses to AMA-1, suggesting that AMA-1 may play a role in targeting a cellular host immune responses against malaria-infected hepatocytes, and thus play a role in the protection that the irradiated sporozoite vaccine provides^23^.

FMP2.1 antigen

For this study, AMA-1, hereby referred to as vaccine antigen FMP2.1, has been produced and manufactured under current good manufacturing practices (cGMP) at the WRAIR Pilot Bioproduction Facility from *Escherichia coli (E. coli)* Tuner^TM^ bacteria, and bottled as a highly purified and lyophilized recombinant protein. The protein consists of 449 amino acids representing the majority of the ectodomain (amino acids 83-531) of AMA-1 of the 3D7 variant with 2 attached His-tags. The protein production and purification has been described previously for FMP2.0^17^, and FMP2.1 differs only in the *E. coli* strain used for production.

## Rationale for this study

Development of a multi-stage, multi-antigen vaccine candidate requires testing of the individual antigens to assess their immunogenicity and efficacy, with the goal of combining the product with other promising vaccine candidates such as RTS,S (candidate malaria antigen, consisting of sequences of circumsporozoite protein and hepatitis B surface antigen (HBsAg)^24^. Along with immunogenicity of the antigen, using appropriate adjuvants can enhance to product’s protection. Both AS02A and AS01B are vaccine adjuvants developed and produced by GSK. AS01B is a liposome-based adjuvant with 3-D-MPL and QS21 while AS02A is an oil-in-water emulsion with the same proportions of MPL and QS-21. The bulk of studies performed using AS02A and AS01B as an adjuvant to a malaria antigen have been in studies with RTS,S; however, studies evaluating other vaccine antigens, such as FMP2.1, with these adjuvants are necessary to select the most safe, immunogenic and efficacious antigen and adjuvant combination to go forward as candidates for a future multi-antigen, multi-stage combination malaria vaccine.

Background on adjuvants AS02A and AS01B, formulated with RTS,S

RTS,S/AS02A

Extensive pre-clinical experimentation conducted in mice and in rhesus monkeys by GSK Biologicals and collaborators demonstrated that this formulation is a powerful inducer of both humoral and CMI responses, including IFN-γ-secreting T-helper 1 lymphocytes. A confirmation of the critical importance of the adequate formulation of AS02 as an adjuvant system for this pre-erythrocytic malaria vaccine was obtained in a clinical trial conducted in collaboration with GSK and WRAIR^25^. In this Phase I/IIa trial, a vaccine consisting of the RTS,S antigen formulated in AS02 protected six out of seven volunteers (86% vaccine efficacy; (95% CI 0.02 to 0.88) from infection following a sporozoite challenge delivered by the bites of laboratory-reared infectious *Anopheles* mosquitoes. In this trial, two other vaccine formulations (adjuvanted with AS04 and AS03) of the same RTS,S antigen were also evaluated but afforded only marginal protection against challenge. Subsequently, in a homologous sporozoite challenge model, the vaccine prevented approximately 40% of individuals from becoming infected and a delay in the prepatent period of non-protected individuals was observed^26^. In field efficacy studies in adult Gambian males^27^, vaccine efficacy (VE) determined as the time to first detection of infection due to *P. falciparum* was 34% (95% confidence interval [CI]: 8% to 53%). The estimated efficacy during the first 9 weeks of follow-up was 71% (95% CI: 46% to 85%), decreasing to 0% in the last 6 weeks. Vaccine efficacy against infection in the second year following a 4^th^ dose of vaccine was 47% (95% CI: 3.8 to 71 %) and a strong boosting of immune responses was induced. In Mozambican children aged 1-4 years, the 18-month point estimate of VE against clinical disease was 35.3% (95% CI 21.6% to 46.6%, p<0.0001), and higher against severe malaria; 48.6% (95% CI 12.3% to 71%, p=0.02)^28,29^.

To date, the RTS,S/AS02A candidate vaccine has been administered to both malaria-naïve adult subjects (subjects who live in countries where there is no naturally occurring malaria transmission) and naturally exposed adults and children (subjects who live in countries where malaria transmission occurs naturally). In trials completed to date, at least 247 naïve adults have received 596 doses, 266 naturally exposed adults have received 842 doses and 1292 naturally exposed children have received 3746 doses.

RTS,S/AS01B

The pre-clinical evaluation of new adjuvant formulations has led to the selection of AS01B as a potential candidate possibly capable of prolonging persistence of the immune response. Preclinical studies in mice and rhesus monkeys show that the AS01B adjuvant induces significantly superior antigen-specific cytotoxic T lymphocyte (CTL) and interferon gamma (IFNγ) responses than does AS02A, while humoral responses generated by both formulations remain equivalent. In rhesus monkey studies, AS01B also induces a cell-mediated response characterized by a more Th1-biased profile: a higher proportion of IFNγ-secreting lymphocytes than observed with AS02A. In toxicology studies in rabbits and primates the vaccine has been shown to be safe.

The RTS,S/AS01 vaccines have been developed in parallel with the RTS,S/AS02 vaccines with the aim of improving the immune response and increasing vaccine efficacy. Two studies are currently ongoing to evaluate RTS,S/AS01 in direct comparison with RTS,S/AS02A. A recent challenge study with RTS,S/AS01B in malaria-naïve adults at WRAIR (Malaria-027) has shown encouraging interim results, indicating a similar safety profile to that of RTS,S/AS02A, higher humoral immunogenicity, a favorable Th1 cell-mediated immune profile and a trend towards higher vaccine efficacy following challenge and rechallenge. To date, 51 participants have received RTS,S/AS01B and while the study has yet to be unblinded, there have been no known vaccine-related severe adverse events (personal communication, K. Kester). The preliminary data confirm that the vaccine is safe, has a similar profile to RTS,S/AS02 and shows a trend towards improved humoral and cellular responses as compared to RTS,S/AS02A. In a second proof of concept study in malaria-exposed adults in Kisumu, Kenya (Malaria-044), 255 adult volunteers were randomized to receive RTS,S/AS01B, RTS,S/AS02A or a commercial rabies vaccine. As of 12 June 2006, all immunizations have been administered, and there have been no vaccine-related SAEs (personal communication, M. Polhemus). The preliminary data confirm that the vaccine is safe, has a similar profile to RTS,S/AS02 and shows a trend towards improved humoral responses as compared to RTS,S/AS02A. Final efficacy data are not yet available for either trial.

Previous Experience with FMP2.1/AS02A and FMP2.1/AS01B

FMP2.1/AS02A

Preclinical studies of FMP2.1/AS02A in rhesus monkeys were performed at WRAIR in 2002. Monkeys were immunized intramuscularly with 50 µg FMP2.1/AS02A at 0-, 1-, 2- months and followed for 9 weeks. This vaccine regimen produced mild pain and muscle swelling after immunization but was not found to be more reactogenic than other vaccines formulated with AS02A. Monitoring of biochemical parameters revealed mild elevations in AST & ALT and creatinine kinase, although these elevations were also seen in saline injected control animals, and therefore thought to be secondary to animal sedation and handling during immunization. Antibody titers as measured by ELISA reached geometric mean titers (GMT) of 9,900 to 30,300 after third immunization and were significantly higher than saline controls (GMT 10). This study demonstrated that FMP2.1/AS02A was safe and immunogenic in rhesus monkeys and supported its use in human clinical trials (BB-IND 11140 submission serial number 0000, Section 8, pages 189-200).

MAL-033, a human Phase I dose escalation study of FMP2.1 formulated in AS02A was completed at WRAIR in 2003. Twenty-three volunteers were immunized with either 10 µg, 25 µg or 50 µg at 0, 1, 2 months, and all three doses were found to be safe and well-tolerated. Eight Grade 3 adverse events (AEs) occurred: one in the 25 µg group and seven in the 50 µg dose group. Five of seven vaccine-related AEs were self-reported from one volunteer receiving the 50 µg dose after second immunization (headache, malaise, myalgias, fatigue, and arthralgias). These and all other AEs resolved by 96 hours. One serious adverse event (SAE) was documented but was determined to be a predisposing condition and not vaccine-related (BB-IND 11140 submission serial number 0015, Section 1.0, pages 2-8, *manuscript in preparation*).

The immunogenicity of AMA-1 was also established during this trial. Volunteers in all three dosage groups were found to have mounted a strong antibody response as demonstrated by ELISA, with geometric mean antibody concentration (GMT) of 100,000 titers units (measured as dilution to give Optical Density (OD)=1) and only a 10-fold decrease in antibody concentration at 6 months after the third immunization. When GIA assays were performed, inhibition of parasite growth reached 15% in 9 out of 18 volunteers and 20% in 6 out of 18 volunteers after 3 immunizations with a range of 3-82%. The percent inhibition of invasion positively correlated with the ELISA titers with a correlation coefficient (R^2^) of 0.748, indicating that the antibodies raised against FMP2.1 were functional. Seventeen serum samples from immunized volunteers also demonstrated processing inhibitory activity greater than pre-immunization serum, further supporting functionality of antibodies generated. Researchers at WRAIR were also able to visualize a positive immunoflouresence assay (IFA) both to sporozoites and trophozoites stages of *Plasmodium falciparum.* Lymphocyte proliferation to AMA-1, as well as increased IFNγ ELISPOT responses, was also demonstrated after the third immunization (BB-IND 11140 submission serial number 0015, Section 1.0, pages 3-8, *manuscript in preparation*).

A controlled Phase I trial of FMP2.1/AS02A was conducted in Malian adults in 2005 comparing 25 µg and 50 µg dose regimens given on a 0-, 1-, 2-month schedule with a commercial rabies vaccine regimen. While the study has not yet been unblinded, no SAEs were reported during trial execution and up to study day 272 (personal communication, C. Plowe). A total of 21 Grade 3 adverse reactions were seen in the 50 µg group (versus 6 in the 25 µg group). All were local injection site swelling greater than 5 cm which did not interfere with Activities of Daily Living (ADLs). Preliminary analysis of immunogenicity revealed that all volunteers achieved antibody levels after the first immunization greater than the mean value plus two standard deviations, with boosting after both the second and third vaccine doses. Further field studies in Mali are scheduled to determine FMP2.1/AS02A vaccine efficacy in children. If FMP2.1/AS02A is deemed efficacious in the pediatric population, the antigen may be considered for integration with the WRAIR-developed RTS,S which has recently demonstrated partial efficacy in children^28,29^.

FMP2.1/AS01B

While there have not yet been any human trials with FMP2.1/AS01B, there are data to support AS01B as an adjuvant formulation and, given the safety and immunogenicity profile of FMP2.1/AS02A, advance FMP2.1/AS01B as the candidate for a combination malaria vaccine. Indications that GSK’s proprietary adjuvant AS01B may be more immunogenic than AS02A in vaccine formulations first came from preclinical studies of the candidate vaccine antigen RTS,S as explained in the previous paragraphs. Clinical human experience with the AS01B adjuvant also has been derived from several studies evaluating liver stage malarial or hepatitis antigens. A double blind Phase I/II immunogenicity and safety study in adults, in which AS01B was formulated with hepatitis B surface antigen (HBsAg) saw no SAEs or clinically significant abnormal hematological or biochemical laboratory test results. Humoral responses to hepatitis surface antigen were high and persistent with cell-mediated immunity studies showing marked lymphoproliferation and IFNγ secretion indicative of a strong Th-1 type immune response (BB-IND 12943, Section 9, pages 1330, and personal communication K. Kester.)

A comparative study of FMP2.0, the precursor vaccine candidate to FMP2.1, in one of four different adjuvants: AS02A, AS01B, AS03, and AS02V, has been done in rhesus monkeys at WRAIR in 2002. The vaccines were administered intramuscularly at 0-, 1- and 2-month intervals. FMP2.0 is a recombinant AMA-1 protein with the same primary amino acid sequence as FMP2.1, differing only in that the protein was expressed using the *E. coli* Origami strain of bacteria. Similar to the preclinical study with FMP2.1/AS02A previously described, there were mild elevations in biochemical parameters (AST, ALT, CK) in all immunized monkeys whether with antigen or saline, presumed secondary to animal manipulation. There were no marked local reactions or serious adverse events with any of the adjuvant formulations. ELISA antibody titers were significantly greater than saline for all groups with each formulation capable of generating GMT of 10,000 after the third immunization. All four vaccine formulations also produced antibodies that inhibited parasite growth as measured by GIA. FMP2.0/AS02A produced greater inhibition than FMP2.0/AS01B although this was not statistically significant. This study concluded that FMP2.0/AS01B, like FMP2.0/AS02A, is safe, immunogenic and elicits functional antibodies in rhesus monkeys (personal communication, A. Stewart).

Summary

The vaccine candidate FMP2.1, a recombinant protein based on the AMA-1 protein of *Plasmodium falciparum*, is expressed on pre-erythrocytic, hepatic and erythrocytic stages of the parasite, and as such is a promising antigen to induce malarial protection by targeting humoral and cell-mediated arms of the immune system. This will be the first human study of FMP2.1 incorporating sporozoite challenge in order to assess vaccine efficacy. In addition, this study will also allow for the comparison of FMP2.1 in two different adjuvants. While the first human vaccine trial with FMP2.1/AS02A demonstrated it to be safe and immunogenic, based on the available preclinical and clinical evidence described, GSK’s proprietary adjuvant AS01B is as safe, may be a more robust adjuvant formulation as compared to adjuvant AS02A, and thus may improve vaccine efficacy. This Phase I/IIa study of FMP2.1/AS01B versus FMP2.1/AS02A would be the first vaccine trial in humans determining the safety, efficacy and immunogenicity of a blood stage antigen in both adjuvant formulations. If either vaccine candidate shows protection, as measured by delay in patency indicating a concomitant reduction in parasite burden, this would possibly accelerate its integration with RTS,S malaria vaccine as a combined formulation. Therefore, this study is a key step in the development process of a multi-antigen malaria vaccine and will contribute information regarding the mechanisms of the human immune response to a multi-stage antigen of *P. falciparum*.

This FMP2.1 Phase I/IIa clinical study will evaluate the safety, reactogenicity, immunogenicity and efficacy profile of FMP2.1 formulated with either AS02A or AS01B after intramuscular administration in two cohorts according to a 0, 1, and 2 month schedule. The 0-, 1-, and 2- month schedule has been selected to allow for rapid immunization of non-immune populations as well as potential future implementation in field studies as it corresponds to the Expanded Program for Immunization schedule. As there are currently no laboratory tests predictive of immunity to malaria, immunized volunteers will be challenged with malaria in order to assess vaccine efficacy. Humoral and cell-mediated immune responses will also be evaluated at specific time points during the study. At completion of this vaccine trial, although direct comparisons between FMP2.1/AS01B and FMP2.1/AS02A will be limited by the small sample size of this study, data suggesting marked differences in safety, immunogenicity and efficacy profiles should be revealed.

# Objectives

## Primary objective

- To assess the safety and reactogenicity of candidate malaria vaccines FMP2.1/AS01B and FMP2.1/AS02A when administered intramuscularly as 3 immunizations on a 0-, 1-, and 2-month immunization schedule to malaria-naïve adult volunteers living in the United States.

## Secondary objectives

- To assess the humoral immune responses by ELISA to candidate malaria vaccines FMP2.1/AS01B and FMP2.1/AS02A.
- To assess functionality of anti-FMP2.1 antibodies induced by both FMP2.1/AS01B and FMP2.1/AS02A to inhibit the growth of asexual parasites as measured by standardized GIA.
- To assess the efficacy of FMP2.1/AS01B and FMP2.1/AS02A against sporozoite challenge with *P. falciparum*

## Tertiary objectives

- To assess the cell-mediated immune responses to FMP2.1/AS01B and FMP2.1/AS02A when administered intramuscularly as three immunizations on a 0-, 1-, and 2-month schedule.

# Study Design Overview

- Double-blind, randomized challenge, Phase I/IIa WRAIR study
- Healthy, malaria-naive adults aged 18 - 50 years
- Given this is a first-in-man study of FMP2.1/AS01B, a staggered dosing schedule of this formulation is incorporated into the study design.
- Two cohorts enrolled for immunization phase:
- **Cohort 1** (not blinded)
- Five participants (10µg IM dose FMP2.1/0.5 mL AS01B)
- **Cohort 2** (blinded)
- From 6 to 15 participants in Group A (50µg IM dose FMP2.1/0.5 mL AS01B)
- From 6 to 15 participants in Group B (50µg IM dose FMP2.1/ 0.5 mL AS02A)
- Goal enrollment of Cohort 2 is 15 volunteers in each group, with a minimum of 6 per group enrolled in order to proceed with study
- Immunization schedule of 0-, 1-, and 2-months for both cohorts
- Immunization of Cohort 1 at least two weeks prior to Cohort 2 will provide safety and reactogenicity data prior to proceeding on to Immunization of Cohort 2
- Sporozoite challenge 14 to 30 days after third immunization **for Cohort 2 only**
- A minimum of 6 and a maximum of 10 participants from each group in Cohort 2 will continue on to sporozoite challenge
- Controls: none for immunization phase; six infectivity controls for challenge phases will be enrolled, with three alternates to be recruited and available for challenge if needed.
- Duration of the study, per participant:
- Cohort 1: up to five months (up to three months screening &

enrollment, two months immunization phase)

- Cohort 2: up to eight months (up to three months screening &

enrollment, two months immunization phase, and three

months challenge phase and follow-up)

- Data collection will be by onsite data entry from case report forms (CRFs) and/or source documents.

# Study Cohort

## Number of participants / centers

Up to forty-one healthy, malaria-naive adults aged 18 - 50 years will be enrolled in a single center. Up to thirty-five participants will receive study vaccines: Cohort 1 will enroll five volunteers to receive 10 µg IM dose of FMP2.1/AS01B and will receive the first immunization at least 2 weeks prior to Cohort 2 to allow for evaluation of the safety and reactogenicity of the FMP2.1/AS01B formulation. Cohort 2 will have 2 groups of volunteers. A minimum of 6 and maximum of 15 participants will be in Group A to receive 50 µg IM dose of FMP2.1/AS01B and a minimum of 6 and maximum of 15 participants will be in Group B to receive 50 µg IM dose of FMP2.1/AS02A. Six healthy, malaria-naive adults aged 18 to 50 years will be enrolled just prior to challenge to act as non-immunized infectivity controls.

No volunteers from Cohort 1 will go on to primary sporozoite challenge. In Cohort 2, up to ten vaccinees from Group A and ten from Group B will continue on to primary sporozoite challenge 14 to 30 days after third immunization. Primary challenge will occur with a minimum of 6 vaccinees and a maximum of 10 from each group. This will allow for adequate assessment of efficacy while exposing the minimum number of volunteers to the risk of malaria challenge. See Section 6.4 for a detailed description of challenge volunteer selection.

Only participants for whom the investigator believes the requirements of the protocol will be complied with (e.g., completion of the diary cards, return for follow-up visits) should be enrolled in the study. See Section 10.6 for a detailed description of the criteria used in the estimation of sample size.

The study will be performed at the Clinical Trials Center, Walter Reed Army Institute of Research, 503 Robert Grant Avenue, Silver Spring, MD 20910, and is funded through the study partner Malaria Vaccine Initiative (MVI).

The following inclusion and exclusion criteria are based on current and past malaria vaccine trials referenced, as well as the investigator brochures, to assure safe conduct of this clinical trial.

## Inclusion criteria

- A male or non-pregnant female 18 to 50 years of age (inclusive) at the time of screening

J*ustification: Persons > 18 years old capable of giving informed consent are required for a Phase I trial. Persons > 50 years old may be at increased risk of poor outcomes following a severe or anaphylactic reaction, and may have decreased immunoresponsiveness.*

- Written informed consent obtained from the participant before screening procedures

*Justification: Participants must sign the Institutional Review Board (IRB)-approved consent form prior to participation in this protocol.*

- Free of clinically significant health problems as established by medical history and clinical examination before entering into the study*

*Justification: persons with significant medical problems are not eligible to participate because their underlying medical condition may make interpretation of adverse events and determination of causality difficult to distinguish from underlying health status.*

- Available to participate for duration of study (approximately five months, not including screening)

*Justification: The participant must be available for the duration of the trial to adequately assess immunogenicity and ensure safety.*

- If the participant is female, she must be of non-childbearing potential, i.e., either surgically sterilized or one year post-menopausal; or, if of childbearing potential, she must be abstinent or have used adequate contraceptive precautions (e.g., intrauterine contraceptive device; oral contraceptives; diaphragm or condom in combination with contraceptive jelly, cream or foam; Norplant® or Depo-Provera®) during this study, have a negative pregnancy test at the time of each immunization, and must agree to continue such precautions for two months after completion of the immunization series and the malaria challenge.

*Justification: It is unethical to expose an embryo to even theoretical or unknown risk in which there is no probability of benefit.*

- Prior to entry into this study, participants must score at least 80% correct on a short multiple-choice quiz that assesses their understanding of this study. If they do not score 80% on the initial quiz, the protocol information will be reviewed with them to ensure comprehension and they will have the opportunity to retest. Participants who fail the Comprehension Assessment for the second time will not be enrolled.

*Justification: An objective measure of determining if the potential volunteer is informed of the basis of this protocol and the procedures involved is highly desirable.*

** While hepatic transaminases must be normal for entry into the study, prospective participants may have a slightly elevated total bilirubin up to 1.9 mg/dL (normal values 0.1-1.0 mg/dL) and still be eligible for participation provided that they have normal transaminases.*

## Exclusion criteria for enrollment

The following criteria should be checked at the time of study entry. If any apply at the time of study entry, the participant must not be included in the study:

- Prior receipt of an investigational malaria vaccine
- Use of any investigational or non-registered drug or vaccine other than the study vaccine(s) within 30 days preceding the first study immunization or planned use during the study period, or receipt of investigational vaccine containing 3-D MPL and/or QS-21 at any time in the past (Have you received an experimental vaccine with a GSK adjuvant in the past?)
- Administration of chronic (defined as more than 14 days) immunosuppressants or other immune-modifying drugs within six months of immunization. (For corticosteroids, this will mean prednisone, or equivalent, greater than or equal to 0.5 mg/kg/day. Inhaled and topical steroids are allowed.)
- For volunteers undergoing malaria challenge, chronic use of antibiotics with anti-malarial effects (e.g., tetracyclines for dermatologic patients, sulfa for recurrent urinary tract infections, etc.)
- Planned administration of a vaccine not foreseen by the study protocol 30 days prior to or after the first immunization
- History of malaria chemoprophylaxis within 60 days prior to immunization
- Any history of malaria
- Known exposure to malaria within the previous 12 months
- Planned travel to malarious areas during the study period
- Any confirmed or suspected immunosuppressive or immunodeficient condition, including human immunodeficiency virus (HIV) infection
- History of allergic disease or reactions to any vaccine
- Chronic or active neurologic disorders including seizures, excluding a single febrile seizure as a child
- History of splenectomy
- Acute disease at the time of enrollment (Acute disease is defined as the presence of a moderate or severe illness with or without fever. All vaccines can be administered to persons with a minor illness such as diarrhea, mild upper respiratory infection with or without low-grade febrile illness, i.e., oral temperature < 37.5°C/99.5°F).
- Acute or chronic, clinically significant pulmonary, cardiovascular, hepatic or renal functional abnormality, as determined by physical examination or laboratory screening tests
- Personal medical histories including the following diagnoses: systemic lupus erythematosus, rheumatoid arthritis, mixed connective tissue disease, scleroderma, vasculitis, and multiple sclerosis
- Seropositive for hepatitis B surface antigen or hepatitis C antibody
- Hepatomegaly, right upper quadrant abdominal pain or tenderness
- Elevated serum creatinine, defined in this study as greater than or equal to 1.7 mg/dL in males and 1.4 mg/dL in females
- Significant unexplained anemia: hematocrit < 35%
- Administration of immunoglobulins and/or any blood products within the three months preceding the first study immunization or planned administration during the study period
- Pregnant or lactating female or female who intends to become pregnant during the study
- Suspected or known current alcohol abuse as defined by the American Psychiatric Association in DSM IV (Diagnostic and Statistical Manual of Mental Disorders- 4^th^ edition)
- Chronic or active illicit and/or intravenous drug use
- History of severe anaphylactic reactions to mosquito bites
- History of psoriasis (given its interaction with chloroquine)
- Any history of anaphylaxis in reaction to immunization
- History of allergy to nickel, imidazole or tetracycline group of antibiotics
- History of sickle cell disease or sickle cell trait
- Any other significant finding that in the opinion of the investigator would increase the risk of having an adverse outcome from participating in this study

## Elimination criteria during the study

The following criteria will be assessed at each visit. If any become applicable during the study, the participant may not be required to discontinue the study but may be eliminated from analysis. See Section 10.5 for definition of study cohorts/datasets to be evaluated.

- Use of any investigational or non-registered drug or vaccine other than the study vaccine(s) during the study period
- Administration of chronic (defined as more than 14 days) immunosuppressants or other immune modifying drugs within six months of immunization. (For corticosteroids, this will mean prednisone, or equivalent, ≥ 0.5 mg/kg/day. Inhaled and topical steroids are allowed.)
- Administration of a vaccine not foreseen by the study protocol during the period starting from 30 days before administration of each vaccine(s) and ending 30 days after
- Administration of immunoglobulins and/or any blood products during the study period
- Elective surgery during period starting two days before and seven days after each immunization
- Known illicit and/or intravenous drug abuse evident through patient history or on physical exam
- Travel to a malarious area during the study period
- Treatment or prophylaxis with anti-malarial medications such as mefloquine and atovaquone/proguanil during the study period
- Reliability/Keeping Appointments
- Any other significant finding that in the opinion of the investigator would increase the risk of having an adverse outcome from participating further in this study

## Contraindications to immunization

The following adverse events associated with immunization constitute absolute contraindications to further administration of vaccine. If any of these adverse events occur during the study, the participant must be withdrawn from the study and followed until resolution of the event.

- Anaphylactic reaction following the administration of vaccine
- Pregnancy

The following adverse events constitute contraindications to administration of vaccine at that point in time. If any one of these adverse events occurs at the time scheduled for immunization, the participant may be immunized at a later date, within the time window specified in the protocol, or discontinued from further immunization and challenge at the discretion of the investigator. The participant must be followed until resolution of the event as with any adverse event.

- Acute disease at the time of immunization. (Acute disease is defined as the presence of a moderate or severe illness with or without fever. All vaccines can be administered to persons with a minor illness such as diarrhea, mild upper respiratory infection with or without low-grade febrile illness, i.e., oral temperature of < 37.5°C/99.5°F).
- Oral temperature of greater than or equal to 37.5°C (99.5°F) at the time of immunization.

# Conduct of Study

## Ethics and regulatory considerations

The study will be conducted according to Good Clinical Practice as outlined in the following documents: the appended Declaration of Helsinki, ICH Guidelines, US 21 CFR Part 50 (Protection of Human Subjects) and Part 56 (Institutional Review Boards), US Army Regulations AR 40-7, AR 40-38, and AR 70-25, and in accordance with the Belmont Report (http://ohrp.osophs.dhhs.gov/humansubjects/guidance/belmont.htm). USAMMDA will have monitoring responsibilities; therefore, the term study monitor will apply to designated representatives of USAMMDA.

Both a study monitor and an independent medical monitor are assigned to this study. A study monitor from GSK has also been assigned to this study. The study monitor from USAMMDA will conduct approximately eight visits before, during and at the conclusion of the study in order to evaluate the conduct of the study. The independent medical monitor will be Dr. Peter Weina in the Department of Experimental Therapeutics, WRAIR. The medical monitor is a qualified physician not associated with this protocol who is able to monitor the volunteers during the study and ensure that medical care is provided for any events that may arise during the study. He will review all unanticipated problems involving risk to participants or others as well as any serious adverse events associated with the protocol and relationship to the study, and is required to write a written report of the event (see Section 8.5.2 for further details).

A Safety Monitoring Committee (SMC), whose members will be selected with study partner MVI (Malaria Vaccine Initiative) in consultation with the investigators, will be appointed. The SMC will consist of a group of experienced clinicians not already participating on the study team. The primary responsibilities of the SMC are to review and evaluate the accumulated study data for participant safety after first immunization of Cohort 1 and as needed and make recommendations to the collaborators concerning the continuation, modification or termination of the study.

The Walter Reed Army Institute of Research Human Use Review Committee (HURC) is the IRB of record for this study. The USAMRMC Human Subjects Research Review Board (HSRRB) will serve as the second-level review board for this study. An additional IRB review will be performed by the review board appointed by MVI, the Western Institutional Review Board, 3535 Seventh Avenue SW, Olympia, WA 98502-5010. All protocol amendments, reports, and notifications will be sent through the WRAIR HURC to the HSRRB and to MVI IRB for review and action.

### Institutional Review Board/Independent Ethics Committee (IRB/IEC)

The IRB/IEC must be constituted according to the local laws/customs of each participating country. It is recommended that it should include:

1. At least five members.
2. At least one member whose primary area of interest is in a non-scientific area.
3. At least one member who is independent of the institution/ study site.

Only those IRB/IEC members who are independent of the investigator and the sponsor of the study should vote on a study-related matter.

A list of members and their qualifications should be obtained from the IRB of record by the US Army Medical Material Development Activity (USAMMDA) Study Monitors.

This protocol and any other documents that the IRB/IEC may need to fulfill its responsibilities, including participant recruitment procedures, and information about payments and compensation available to participants, will be submitted to an appropriate Committees or Boards by the investigator, and their written unconditional approval should be in the possession of the investigator and the sponsor before commencement of the study. Relevant GSK Biologicals data will be supplied by GSK central study coordinator to the investigators or USAMMDA for transmission to the IRB/IEC for the protocol's review and approval. Verification of IRB/IEC unconditional approval of the protocol and the written informed consent statement will be transmitted by the investigator to USAMMDA, GSK and MVI using the standard notification forms, generally prior to shipment of vaccine supplies to the site. This approval must refer to the study by exact protocol title and number, and should identify the documents reviewed and state the date of review.

No deviations from, or changes to, the protocol approved by the IRB/IEC should be initiated without prior written IRB/IEC approval/ favorable opinion of an appropriate amendment or modification, except when necessary to eliminate immediate hazards to the participants or when the change(s) involves only logistical or administrative aspects of the study (e.g., change of monitor(s), telephone number(s).

The IRB/IEC must be informed by the investigator of:

- all subsequent protocol amendments, informed consent changes or revisions of other documents originally submitted for review
- serious and/or unexpected adverse events occurring during the study, where required
- unanticipated problems or risks to study participants or others
- new information that may affect adversely the safety of the participants or the conduct of the study
- an annual update and/or request for re-approval, where required
- study completion, where required.

### Informed consent

Informed consent will be obtained in accordance with 21 CFR 50.25, 32 CFR 219, 45 CFR 46, AR 70-25, OTSG 15-2 and the principles of the appended Declaration of Helsinki.

Information should be given in both oral and written form whenever possible and deemed appropriate by the IRB/IEC.

The written consent document will embody the elements of informed consent as described in the current edition of the Declaration of Helsinki, will adhere to the ICH Harmonised Tripartite Guideline for Good Clinical Practice and will also comply with local regulations.

An investigator or designate will describe the protocol to potential subjects face to face. The Subject Information and Consent Form may be read to the subjects, but, in any event, the investigator shall give the subjects ample opportunity to inquire about details of the study and ask any questions before dating and signing the Consent Form.

Subjects must be informed of the research aspect of the trial, the purpose of the trial, the aspects of the trial that are experimental, the expected benefits, the possible risks (including a statement that the particular treatment or procedure may involve risks to the subject {or to the embryo or fetus, if the subject is or may become pregnant} which are currently unforeseeable), the expected duration of the subject’s participation in the trial, the subject’s responsibilities, the approximate number of subjects involved in the trial, the procedures of the research study including all invasive procedures and the probability for random assignment to treatment groups. Subjects will be informed that they will be notified in a timely manner if information becomes available that may be relevant to their willingness to continue participation in the trial. They must also be informed of alternative procedures that may be available and the important potential benefits and risks of these available alternative procedures. Subjects must receive an explanation as to whether any compensation and any medical treatments are available if injury occurs as a result of participating in the study and, if so, what they consist of, or where further information may be obtained. Subjects must be informed of the anticipated financial expenses, if any, to the subject for participating in the trial as well as any anticipated prorated payments, if any, to the subject for participating in the trial. They must be informed whom to contact (e.g. the investigator) for answers to any questions relating to the research project. Information will also include the foreseeable circumstances and/or reasons under which the subject’s participation in the trial may be terminated. The subjects must be informed that the subject’s participation is voluntary and that they are free to withdraw from the study for any reason at any time, without penalty or loss of benefits to which the subject is otherwise entitled. Neither the investigator, nor the trial staff, should coerce or unduly influence a subject to participate or to continue to participate in the trial. The extent of the confidentiality of subject records must be defined, and subjects must be informed that applicable data protection legislation will be complied with. Subjects must be informed that the monitor(s), auditor(s), IRB/IEC, and the regulatory authority (ies) will be granted direct access to the subject's original medical records for verification of clinical trial procedures and/or data, without violating the confidentiality of the subject, to the extent permitted by the applicable laws and regulations and that, by signing a written informed consent form, the subject is authorizing such access. Subjects must be informed that records identifying the subject will be kept confidential, and to the extent permitted by the applicable laws and/or regulations, will not be made publicly available and, if the results of the trial are published, the subject’s identity will remain confidential. Measures to be taken to ensure confidentiality of volunteer information include double-locked system of file maintenance in CTC, use of volunteer identification number to protect volunteer identity, and a database entry system accessible only by selected staff in CTC.

The consent form generated by the investigator must be approved (along with the protocol and any other necessary documentation) by the IRB/IEC. Consent forms must be in a language fully comprehensible to the prospective subjects. Informed consent shall be documented by the use of a written consent form approved by the IRB/IEC and signed and dated by the subject, and by the person who conducted the informed consent discussion. The signature confirms the consent is based on information that has been understood. Each subject's signed informed consent form must be kept on file by the investigator for possible inspection by Regulatory Authorities. The subject should receive a copy of the signed and dated written informed consent form and any other written information provided to the subjects, and should receive copies of any signed and dated consent form updates and any amendments to the written information provided to subjects. Oral witnessed consent will replace written consent only in countries where the local custom is contrary or if the subject’s incapacity precludes this and provided that the local legal obligations are fulfilled.

## General study aspects

This study will enroll malaria-naïve adults living in the United States. Enrolled participants are expected to refrain from travel to malarious areas during the study period. Any such travel necessitates the intake of adequate anti-malarial chemoprophylaxis, irrespective of immunization status, and may lead to elimination of the participant from analysis. Any participant who does so will be discontinued from immunization and challenge.

### Screening

Healthy adult volunteers will be recruited as vaccinees or infectivity controls by non-coercive means through the WRAIR Clinical Trials Department and the Department of Immunology according to applicable U.S. Army regulations (i.e., AR 70-25 and AR 40-38). The first 35 eligible immunization volunteers will be recruited, immunized with one of two candidate vaccines FMP2.1/AS01B or FMP2.1/AS02A, and from 12 to 20 volunteers will complete the challenge. A minimum of 17 volunteers can be enrolled in the immunization phase: 5 in Cohort 1 and 12 in Cohort 2 (6 per group) with optimal (and maximal) enrolment numbers of 5 volunteers in Cohort 1 and 30 volunteers in Cohort 2 (15 per group). Cohort 2 volunteers will be randomly assigned to Group A or B, with randomization based from a computer-generated randomization list and assigned after volunteers are successfully screened. See Section 6.4 for details on volunteer selection.

Infectivity controls will be enrolled separately for the challenge phase. Infectivity controls are recruited just prior to challenge; six volunteers will be enrolled with up to three alternate volunteers being recruited.

Volunteers will provide a medical history (with special attention to a review of cardiac and renal systems, previous history of splenectomy, recent antibiotic usage, and prior vaccine reactions) and undergo physical examination and routine standard laboratory screening tests to include: complete blood count (CBC), serum chemistry profiles (glucose, electrolytes, blood urea nitrogen [BUN], creatinine, alanine aminotransferase [ALT], aspartate aminotransferase [AST], alkaline phosphatase, lactate dehydrogenase [LDH], and total bilirubin), urinalysis, urine beta-human chorionic gonadotrophin [β-HCG] - for females to exclude pregnancy, serological studies (HBsAg, anti-hepatitis C, and HIV-1 assessment) to characterize prospective volunteers' hepatitis B, and C serologic status, as well as to exclude HIV-1 infection. While hepatic transaminases must be normal for entry into the study, prospective participants may have a slightly elevated total bilirubin (up to 1.9 mg/dL) and still be eligible for participation provided that they have normal transaminases. All volunteers will sign an informed consent document to participate in the study (see Appendix F, Appendix G and Appendix H) and an additional informed consent statement for HIV-1 testing (see Appendix I) and for blood donation (see Appendix J).

Volunteers will be excluded from participation if they have had a splenectomy, have any significant cardiovascular, hepatic, or renal system abnormality, are immunodeficient either by history or laboratory evaluation, have a history of sickle cell disease or trait, are pregnant based on urine β-HCG assessment, or have any other finding that would increase the risk of having an adverse outcome during the conduct of the trial. Participants with a personal history of autoimmune disease will be excluded. Exclusionary personal medical histories will include the following diagnoses: systemic lupus erythematosus, rheumatoid arthritis, mixed connective tissue disease, scleroderma, vasculitis, and multiple sclerosis. Volunteers found to be seropositive for HIV-1, Hepatitis B or C will be counseled and referred to their regular health care provider for further evaluation.

All screening tests will be performed prior to entry into the study and at any other time during the course of the trial judged necessary by the investigators. Screening and follow-up diagnostic laboratory studies will be performed at Quest Diagnostics. All Quest diagnostic tests will use volunteer name and unique alphanumeric identifier allocated according to CTC SOPs for participant identification. For those volunteers who go on to enroll in the study, a different alphanumeric code will be used for participant identification on study documents. Screening history, physical examination, and laboratory findings for both vaccine recipients and infectivity controls will be recorded on a case report form (CRF) (see attached Draft CRFs).

Participants will be required to pass a short multiple-choice quiz that assesses their understanding of this study prior to enrollment (see Appendices K, L, & M).

Blood samples will be drawn from the enrolled participants prior to immunization in order to measure malaria-specific antibody titers and cell-mediated immune responses.

### Study Design

Two cohorts will be enrolled, with a minimum of 17 and maximum of 35 total immunization volunteers. Immunization will be performed according to a 0-, 1-, and 2-month schedule with an initial minimum 2-week stagger to allow for safety evaluation of FMP2.1/AS01B. Five volunteers (Cohort1) will be selected to receive 10 µg FMP2.1/AS01B. This selection will not be randomized nor blinded. These volunteers will receive the first immunization, safety and reactogenicity data will be collected and if this formulation is determined to be safe in humans, immunization of Cohort 2 will begin at least two weeks after the immunization of Cohort 1. For Cohort 2, a minimum of 6 and maximum of fifteen volunteers will be randomized to Group A (50 µg FMP2.1/AS01B) and from 6 to15 volunteers will be randomized to Group B (50 µg FMP2.1/AS02A). From 6 up to 10 of the 15 from each group will be randomly selected (see Section 6.4) and proceed to primary sporozoite challenge 14 to 30 days after the third immunization. Approximately six control volunteers per day of challenge will be recruited.

### Safety considerations during the trial

**Safety considerations for FMP2.1/AS01B:**

Given that this is a first-in-man study for FMP2.1/AS01B, immunization will be performed with a minimum 2-week stagger for this formulation. Volunteers in Cohort 1 will receive 1/5^th^ dose (10 µg) FMP2.1 and full dose adjuvant (0.5 mL of AS01B) two weeks prior to planned immunization of Cohort 2. Previous malaria vaccines trials at WRAIR have demonstrated that most adverse events related to the vaccine occur in the first 48 hours after immunization (personal communication, K. Kester, M. Polhemus, and J. Cummings). Therefore, vaccine safety and reactogenicity data following 4 days of follow-up (day of vaccination and 3 subsequent days) will be collected by the PI and reviewed by the independent Medical Monitor and Safety Monitoring Committee prior to proceeding to vaccination of subjects in Cohort 2.

The SMC will include independent experienced clinicians chosen by MVI charged with assisting the PI and the Medical Monitor with timely and thorough evaluation of the safety reports. The SMC will meet telephonically to review the safety data and adjudicate whether the trial should continue. They will be one of the parties responsible for deciding whether to progress to Cohort 2 and whether to resume immunizations if the trial has been suspended. Representatives from GSK and MVI as well as the PI will be involved in the SMC communications. It is not the role of the SMC to comment on the study design, conduct or interpretation rather to act as independent experts in evaluating adverse events. The SMC may, if deemed necessary, convene a meeting with, or request further information from the PI or the Medical Monitor. Copies of the outcome of the SMC meetings will be provided to the USAMRMC Office of Research Protections and all participating IRBs.

**Cohort 1**

Holding Rules:

A. Systemic adverse events: Vaccination of a cohort can be put on hold if more than 1 subject in a preceding cohort develops a vaccine-related Grade 3 adverse event lasting > 48 hours beginning within 2 days after vaccination if the adverse event is malaise, myalgia, fatigue, nausea, fever, headache, or there is any vaccine-related serious adverse event.

Activation of the Holding Rule requires a thorough review by the independent WRAIR Medical Monitor and SMC and discussion between the investigators, the Medical Monitors, and Sponsor. Vaccination for the subjects enrolled in the Cohort 2 will start only if all parties agree to a resumption of vaccination.

In addition to the above-stated group Holding Rules, the following Stopping Rules for individual subjects will apply (i.e., the withdrawal of an individual subject from further vaccination):

Stopping Rules:

A. Local Reactions: Investigator discretion

B. Systemic adverse events: Individual subjects will be withdrawn from further vaccination if they develop a vaccine-related Grade 3 adverse event beginning within 2 days after vaccination and persisting at Grade 3 for ≥ 48 hours if the adverse event is malaise, myalgia, fatigue, fever, headache, or joint pain or if the subject has any other Grade 3 adverse event considered to be associated with vaccination or any vaccine-related serious adverse event.

Procedure for reviewing the safety and reactogenicity data in Cohort 1:

After all 5 subjects have been followed for 4 days (day of vaccination and 3 subsequent days), the PI or his designee will summarize all solicited general adverse events related to vaccination and any SAEs related to vaccination and give this to the WRAIR independent medical monitor and the SMC. The WRAIR medical monitor and the SMC will review the data on day 8 (+/- 4 days) and allow further vaccination of the remaining 30 subjects (Cohort 2) or put further vaccination on hold.

**Cohort 2**

Volunteers for immunization will be divided into two groups. Group A will receive 50 µg FMP2.1/AS01B; Group B will receive 50µg FMP2.1/AS02A. Vaccine safety and reactogenicity will be assessed and recorded the first four days of follow-up (day of immunization and three subsequent days), and will be reviewed by the blinded independent WRAIR medical monitor.

Further immunization can be suspended by the independent medical WRAIR monitor as well as the Safety Monitoring Committee (SMC) and is subject to discussion by the investigators, medical monitors and sponsors, if suspension criteria are met.

Holding Rules: for a vaccine formulation cohort

Holding Rules:

A. Systemic adverse events: Immunization of a cohort can be put on hold if 3/15 participants develop a Grade 3 adverse event beginning within two days after immunization and persisting at Grade 3 > 48 hours if the adverse event is malaise, myalgia, fatigue, nausea, fever, headache, or joint pain, or if there are any vaccine-related Serious Adverse Events.

B. Laboratory adverse events: Immunization of a cohort can be put on hold if 3/15 participants develop a Grade 2 laboratory adverse event which last for ≥ 48 hours or 2/15 participants develop any Grade 3 laboratory adverse event considered to be associated with immunization.

Activation of the Holding Rule for Cohort 2 requires a thorough review by the independent WRAIR Medical Monitor and SMC and discussion among the investigators, the Medical Monitors, Sponsor and study partners.

In addition to the above-stated group Holding Rules, the following Stopping Rules for individual participants will apply (i.e., the withdrawal of an individual participant from further immunization):

Stopping Rules: for an individual vaccinee

Stopping Rules:

A. Local Reactions: Investigator discretion

B. Systemic adverse events: Individual participants will be withdrawn from further immunization if they develop a Grade 3 adverse event beginning within two days after immunization and persisting at Grade 3 > 48 hours if the adverse event is malaise, myalgia, fatigue, fever, headache, or joint pain or if the subject has any other Grade 3 adverse event considered to be associated with vaccination, or if they have any vaccine-related Serious Adverse Event.

C. Laboratory adverse events: Individual participants will be withdrawn from further immunization for any Grade 2 laboratory adverse event that lasts for ≥ 48 hours or any Grade 3 laboratory adverse event considered to be associated with immunization.

If the PI or sub-investigators, medical monitor or MVI has any further safety concerns, the SMC committee can be convened by MVI to review the safety data. The committee will provide verbal and hard copy written reports to the investigators, USAMRMC Office of Research Protections (ORP), MVI, GSK and the participating IRBs. Findings of a serious and immediate nature including any recommendations to modify or discontinue all or part of the study must be reported immediately in verbal and written form as indicated in Section 8.5.

Under circumstances where there are safety concerns that warrant no immunization of a cohort, no further immunizations will be performed, and USAMRMC Office of Research Protections (ORP). HURC, HSRRB and Western IRB, MVI and GSK will be promptly notified. All immunized participants will be followed for safety until resolution of their adverse events or until at least 30 days after the last immunization. A formal letter will be sent to the FDA by the sponsors explaining the reasons for cessation of the study.

### Risks to the Participants

#### Risks associated with immunization

The most frequent adverse reactions observed in previous clinical trials using AS01B and AS02A adjuvant formulations include pain, swelling, erythema, and tenderness at the site of injection, and systemic symptoms such as low-grade fever and short-term flu-like symptoms: fatigue, myalgia, headache, malaise. There is also the remote possibility of an allergic reaction to the vaccine components. There have been no reports of allergic reactions in previous malaria vaccine studies at WRAIR using the AS02A adjuvant, nor any such reactions in the MAL-033 study in which 23 volunteers received FMP2.1/AS02A. Although FMP2.1/AS01B has not been used in humans, approximately 372 adults have received AS01B with differing antigen formulations. Approximately 130 adult volunteers have received AS01B formulated with RTS,S at WRAIR with one mild, transient uticarial reaction being reported. Because FMP2.1/AS01B is an experimental vaccine being administered to humans for the first time, there may be unknown risks. By participating in the study, the study participants will undergo phlebotomy, which carries its own associated risks of infection, vascular damage, bruising and clot formation. Although every measure will be taken to assure the confidentiality of participants in this study, there is the theoretical risk associated with breach of confidentiality by participating in this study.

#### Risks associated with malaria challenge

Risks associated with malaria challenge include local inflammatory reactions to mosquito bites as well as the development of malaria infection. Additional risks include side effects from chloroquine, quinine, atovaquone/proguanil or doxycycline (e.g., nausea, vomiting, diarrhea, headaches, blurred vision, pruritus, tinnitus, or photosensitivity) and the unlikely possibility of complications of malaria, which are seen during naturally acquired malaria when diagnosis and treatment are delayed and high levels of parasitemia develop. Under the carefully controlled conditions of this study, the chance of serious illness or death from malaria infection is very small. In challenge studies involving nearly 500 study subjects over the past 15 years at the WRAIR, there has never been a case of mortality or excess morbidity requiring inpatient hospitalization. Transient abnormalities, such as fever, headache, mild anemia, leukopenia, splenomegaly, hepatic tenderness and fatigue, are expected consequences of malaria. In uncontrolled circumstances, malaria infections can lead to kidney, liver or brain damage, and death. Other remotely possible risks include a systemic allergic reaction to mosquito bites and the chance that the mosquitoes may transmit another infectious agent. No previous volunteers challenged using this procedure have developed a complication of malaria or has been unable to take the prescribed course of oral antimalarials.

The WRAIR investigative staff members who are in the insectary during the malaria challenge have a small risk of exposure to malaria. This small risk is greatly minimized by following stringent insectary SOPs regarding the handling of mosquitoes, the execution of mosquito challenge and physical movement inside the insectary.

### Precautions to Minimize Risk

#### Immunization

As outlined above, the volunteers will be monitored closely during their participation in this study. The study vaccines have been prepared according to Good Manufacturing Practices (GMP). The vaccines will be administered in the WRAIR Clinical Trials Department under the supervision of an investigator and nurses equipped with drugs and equipment to immediately treat anaphylaxis. Although an exaggerated adverse local reaction occurring at a single site after repeated immunizations is not expected with these antigen/adjuvant combinations, each immunization will be administered to alternate arms, with the first immunization in the left arm, second in the right arm and third in the left arm. Prior to each immunization, the arm will be inspected for lesions, wounds or clinically evident physical findings that might interfere with post-immunization assessment of reactogenicity. If such a finding is discovered, the unaffected arm will be used, even if the alternate arm schedule is not able to be preserved. Each vaccinee will be closely observed for at least 30 minutes following immunization. Participants will also be evaluated on Days 1, 2, 3, 7 and 14 for evaluation of any adverse events occurring after immunization by the investigating team. They will record any local or general reactions on diary cards for Days 4, 5, and 6 (in addition to Days 1-3, 7).

### Assessment of Vaccine Efficacy

#### Malaria Challenge

Volunteers in Cohort 1 will not proceed on to primary sporozoite challenge. The testing and evaluation of the 10 µg dose of FMP2.1/0.5 mL AS01B will provide crucial safety and reactogenicity information needed prior to proceeding with a higher dose formulation of FMP2.1/AS01B; however, the lower dose formulation is not a candidate for advancement in future malaria vaccine trials, and therefore the Cohort 1 volunteers will not be invited to participate in the malaria challenge. All volunteers from Cohort 2 (Groups A and B) successfully participating in and completing the Immunization Phase will be eligible to be challenged with malaria to determine whether the expected protective response has developed. Up to 10 volunteers from each group will be able to participate in the challenge phase, with a minimum of 6 required. Selecting from 6 to 10 of the 15 total volunteers per group to undergo sporozoite challenge will permit detection of vaccine efficacy while exposing the minimum number of volunteers to the risks of malaria challenge. See Section 6.2 for details regarding selection of the challenge volunteers. It is anticipated the group will be challenged within the target of 14-30 days following the final immunization. Such challenges are mandated by the absence of any laboratory tests that will unequivocally predict protection. The unimmunized infectivity control volunteers will be challenged to verify the adequacy of the challenge. We feel that the use of six unimmunized, malaria-naive controls per challenge day is adequate since the procedure described has generally successfully infected such volunteers^30^. As in past vaccine trials, one day is sufficient to complete the challenge process for less than or equal to 30 participants. Volunteers undergoing malaria challenge will receive a challenge-specific information sheet and identification card that outlines their participation in the study, as well as the appropriate investigator contact telephone numbers. Details of this information may vary, depending on the venue selected for the hotel phase of the study.

During the evaluation of protective efficacy, as soon as a malaria infection is documented in a volunteer, (s)he will be treated with a standard dose of oral chloroquine under direct observation. To date, this regimen has been 100% effective against the challenge malaria strain NF54 (clone 3D7). Such early treatment minimizes the risk of developing a complicated malaria infection.

#### Parasite and mosquito strains and infection of mosquitoes

The 3D7 clone of *P. falciparum* is a human malaria isolate that has never been passed through monkeys, is well adapted to culture, is a good producer of gametocytes that can infect mosquitoes, is susceptible to currently available, approved, anti-malarial compounds, and has previously been used in the WRAIR challenge model to successfully infect human volunteers. Master seed lots of these parasites have been developed and stored at WRAIR. No volunteer challenged with these parasites in the context of malaria vaccine studies performed at WRAIR has died or experienced unacceptable morbidity. The parasites will be thawed from the master seed lots and expanded in normal human erythrocytes using standard culture medium, which contains 10% heat inactivated (56°C, 30 minutes) normal human serum. All blood products used for malaria and mosquito culturing will be commercially tested for HIV, hepatitis B, C, and syphilis. The mosquitoes used will be laboratory-born and reared *Anopheles stephensi.* This species is relatively easy to maintain in the laboratory for up to 21 days after infection with malaria, feed readily on humans, and is able to transmit malaria to volunteers^31^. Colonies of mosquitoes used have previously been screened for the presence of infectious agents. Mosquitoes will be infected by allowing them to feed through membranes on cultures of *P. falciparum* containing a large proportion of gametocytes. One week after *P. falciparum* infection, 20 mosquitoes will be removed and dissected to estimate the malaria infection rate. If the rate is less than 50%, attempts to infect mosquitoes will be repeated using a new batch of mosquitoes and a fresh malaria culture. Beginning approximately 16 days after infection, batches of 10 mosquitoes will be removed daily and dissected to look for sporozoites in their salivary glands. If the mean sporozoite density is less than 100 per mosquito, attempts to infect mosquitoes will be repeated using a new batch of mosquitoes and a fresh malaria culture.

#### Infection of Human Volunteers

Mosquitoes infected approximately 17 to 19 days earlier that are likely to contain sporozoites in their salivary glands will be allowed to feed on the forearm of the volunteers. For each volunteer, five mosquitoes will be allowed to feed over five minutes, after which they will be dissected to confirm the five mosquitoes were infected, and then the salivary glands scored. If required, additional mosquitoes will be allowed to feed until a total of five infected mosquitoes with a minimum 2+ salivary gland score have fed^32^. The salivary gland score rates the mosquito’s salivary gland for the presence of sporozoites. The assessment is based on the following published scale^33^:

  0     no sporozoites observed

+1      1-10 sporozoites observed

+2      11-100 sporozoites observed

+3      101-1000 sporozoites observed

+4      >1000 sporozoites observed

The mosquito feedings will be performed in the secure insectary of the Department of Entomology at WRAIR. Volunteers will be observed for 30 minutes following completion of the sporozoite challenge in order to assess them for any evidence of acute allergic reactions related to mosquito exposure. To date, no severe allergic reactions related to mosquito bites have been documented in the context of the WRAIR malaria challenge model. Routinely, transient local allergic reactions (itching, rash) typical of mosquito bites occur at sites of the bites.

#### Management of Infected Human Volunteers

The prepatent period for human *P. falciparum* infection normally averages 9-12 days. In previous studies, the pre-patent period in controls range varied from 7-18 days. The shortest reported prepatent period documented in immunized volunteers is 7 days^34^, and the longest delay (excluding volunteers completely protected) is 25 days (personal communication, K. Kester). An immunized individual who does not have complete protection may have a prolongation of the parasite prepatent period.

Volunteers for challenge will return to the Department of Clinical Trials, WRAIR the day after challenge for blood draw for PCR testing and microarray analysis. Beginning on day 5 after their challenge, they will be evaluated daily by a physician member of the study, and have daily blood films performed as well as PCR and microarray analysis drawn as per schedule. The Giemsa-stained thick blood films are obtained and examined for the presence of malaria parasites. All blood smears will have at least 200 high-power (oil immersion) fields scanned for the presence of malaria parasites. Symptomatic volunteers will have at least 1000 high-power (oil immersion) fields reviewed. If fever (oral temperature above 37.5°C) develops at any time, blood films will be obtained more frequently (every 6 to 8 hours), and a study physician will evaluate the volunteer. The blood films will be read by trained microscopists. A positive reading will be confirmed by the expert microscopist. A confirmed positive result will be relayed immediately to the on-call physician by the microscopists. Volunteers who develop malaria will be treated with a standard dose of chloroquine (a total of 1500 mg chloroquine base (2500 mg salt) P.O. in divided doses: 600 mg (two 500 mg chloroquine salt tablets) initially, followed by 300 mg (500 mg chloroquine salt tablet) given approximately 6, 24, and 48 hours later) under direct observation. Chloroquine phosphate and, if required, other appropriate FDA-approved drugs, such as quinine, atovaquone/proguanil and doxycycline, currently available on the Walter Reed Army Medical Center Formulary, will be used in standard doses to treat volunteers who become infected with malaria. These drugs will be available both in the WRAIR Clinical Trials Department, as well as at the hotel used to house volunteers during the period of intense assessment for malaria following the sporozoite challenge. The infection will be treated early (i.e., as soon as parasites can be identified on thick smear). In many cases, malaria infection has often been identified and treated based upon positive blood smear results prior to the development of clinical symptoms of malaria. All blood films (positive and negative) will be archived in the WRAIR Department of Immunology for later re-examination and confirmation, if required. The slides will be archived until clinical records are eligible to be destroyed, or 75 years.

Beginning the evening of Day 9 post-challenge, a group of hotel rooms in the local area will be reserved for malaria-challenged volunteers. The volunteers will be required to spend their nights there to allow for more rapid assessment of any potential symptoms of experimental malaria during the hours that the WRAIR Clinical Trials Department is closed. There will be an investigator in the hotel each night and all weekend long. During the weekdays, it is possible that the investigator might be off-site but still be accessible via phone or pager. There will also be qualified study personnel in the hotel 24 hours per day. All challenged volunteers will be contacted by telephone each afternoon during the hotel stay until they are positive for malaria. They will be asked the same health status questions that were asked during the morning evaluation (e.g., headache, muscle aches, etc.). At any time required, the on-duty investigator will arrange for the timely production of blood smears, along with their examination and interpretation, in order to treat rapidly those volunteers in whom therapy for malaria is indicated. Once a positive smear is identified and treatment is initiated, daily blood films will continue to be obtained until three consecutive films are negative.

The maximum hotel stay for malaria-challenged volunteers should be approximately ten nights. A volunteer who develops malaria, is treated, and has 3 consecutive negative malaria smears, will not need to remain in the hotel. The investigators will be responsible for accounting for any volunteers who do not arrive in the hotel during the challenge phase. If required, the investigators will physically locate and treat any malaria-infected volunteer who is unable to maintain the follow-up dictated by this study.

If a volunteer becomes ill or injured as a direct result of participating in this research study, (s)he will be provided medical care for that illness or injury. This care will be provided at the Walter Reed Army Medical Center (WRAMC) in Washington, DC at no no-cost to the volunteers. A study-specific malaria diagnosis and treatment outline will be submitted to the staff of the WRAMC Emergency Department for their information and review at the time of sporozoite challenge.

As soon as a malaria infection is documented in a volunteer, (s)he will be treated with standard doses of oral chloroquine as described previously. This regimen has been 100% effective in previous WRAIR malaria vaccine studies using the same malaria strain as will be used in this challenge model. Such early treatment minimizes the risk of developing a complicated malaria infection.

The malaria strain used for challenge (*P. falciparum* strain NF54/clone 3D7) is sensitive to several currently available, licensed, anti-malarial drugs that are safe, effective and have a low incidence of side effects.

The investigators will have available approved antipyretics, such as acetaminophen and ibuprofen, for participants experiencing fever and myalgias. In addition, other approved medications will be available to the investigators, which may include, but are not limited to: acetaminophen with codeine (Tylenol #3™), trimethobenzamide (Tigan™), and loperamide (Imodium™) to treat other signs/symptoms as necessary. Investigators will always assure that participants do not have underlying allergies to any of these medications prior to their use. An alternative antipyretic will be provided if the participant is allergic to a prescribed drug.

It is anticipated that treatment of malaria will be curative, since relapses do not occur after adequate treatment of *P. falciparum* infections. No previous study volunteer infected and treated as part of a WRAIR malaria vaccine protocol has had a malaria relapse. Nonetheless, once weekly evaluations will be performed for at least four weeks after treatment to evaluate the unlikely possibility of recrudescent infection. In addition, participants will be advised to contact the study physician, or to advise their personal physician of their participation in this malaria study, if fever, headache, or other symptoms possibly related to malaria develop at any time within one year after completion of the study. In the unlikely event that malaria recurs, the participant will be retreated with chloroquine as outlined above, or atovaquone/proguanil (1000 mg atovaquone/400 mg proguanil e.g., 4 tablets, once a day for 3 consecutive days) or with a combination of quinine (650 mg three times a day for three days) and doxycycline (100 mg twice daily for 7 days).

If infection does not develop within the first 20 days post challenge, the volunteer will be released from staying nightly at the hotel. They may be required to present to the Clinical Trials Center for both evaluation and blood smears Monday – Friday for study days 21 – 30 (excluding weekends and holidays). Owing to the potential logistical difficulty incurred by the requirement of daily blood smears, asymptomatic volunteers with persistently negative smears may, at the discretion of the Principal Investigator, be excused from daily phlebotomy after post-challenge day 20. Assessment by an investigator with phlebotomy will still be required, however, if any symptoms develop. Telephone contact will be made if the volunteer does not keep a scheduled follow-up appointment. After post-challenge day 30, the volunteer will have blood drawn, have a blood smear made and be seen weekly times four in the Clinical Trials Center by a physician investigator or qualified study personnel.

No human viruses are known to be transmitted by colonized *Anopheles* mosquitoes. Blood purchased for malaria feedings has been commercially tested using FDA-approved test methods for antibodies to HIV, hepatitis C, and syphilis, as well as for the presence of hepatitis B surface antigen; all blood has tested negatively for these tests. No documented cases of HIV or viral hepatitis transmission from mosquitoes to humans have occurred.

The risk of accidentally transmitting malaria to a person in the community will be negligible because:

- The infected mosquitoes will be raised in the secure insectary at WRAIR,
- All malaria challenges occur in the secure insectary,
- The infected mosquitoes never leave the secured insectary area at any time.
- Additionally, malaria infections in volunteers will be treated promptly before

gametocytes can develop (generally ten days after the development of patent malaria), thus the risk of transmission to local mosquitoes is remote.

## Outline of study procedures

It is the investigator’s responsibility to ensure that the intervals between visits/contacts are strictly followed. These intervals during the active phase of the study determine a participant’s evaluability in the According To Protocol analyses (see Section 10.5 for details of criteria for evaluability). For the Immunization Phase, Cohorts 1 and 2 will follow the same study procedures. For the Challenge Phase, Groups A and B in Cohort 2 will follow the same study procedures. A list of all study investigators at WRAIR and their roles and responsibilities have been outlined in Appendix O.

## Detailed description of study phases/visits

### Screening and Immunization phase

All participants enrolled in the study will be issued an emergency notification card that details their participation in the study and specific contact phone numbers of the investigators and the WRAIR Clinical Trials Center. Table 1 outlines the procedures of the immunization phase of the study for both Cohorts 1 and 2 excepting Cohort 1 will not have blood drawn for microarray analyses.

**Visit 1: Day-90 to -3**: **Screening/enrollment of volunteers**

- Obtain written informed consent for clinical study and for HIV testing
- Informed consent/protocol comprehension assessment
- Provision of medical history by volunteer
- Physical examination
- Check of inclusion and exclusion criteria
- Blood collection: Approximately 20 mL whole venous blood sample for the determination of:
- Serology (HBsAg, anti-hepatitis C Ab, and HIV-1 assessment) to be carried out at a local laboratory
- Complete blood count (CBC)
- Urine pregnancy test where applicable
- Serum chemistry profiles (glucose, electrolytes, BUN, creatinine, ALT, AST, alkaline phosphatase, LDH, and total bilirubin)
- Urinalysis (to assess for evidence of renal dysfunction; drug testing will not be performed)

**Visit 2: Day-90 to -3**: **Blood draw for** **enrolled** **volunteers**

- Check of inclusion and exclusion criteria and contraindications/precautions
- Blood collection: Approximately 157 mL whole venous blood sample for the determination of:
- 7 mL for microarray analysis (Cohort 2 only)
- 20 mL for humoral analyses (ELISA and GIA). The research samples will be centrifuged and the serum stored at ‑20°C (±5°C) or colder until processing.
- Approximately 130 mL whole venous blood for the baseline CMI determination. Samples will be stored at 20-25°C until being transported to CMI laboratory***.***  No CMI will be drawn for infectivity controls.

**Visit 3: Day 0: Immunization 1**

- Check inclusion/exclusion criteria and contraindications/precautions.
- Randomization of enrolled volunteers to designated vaccine group
- History, directed physical examination, and recording of pre-immunization data including blood pressure (BP), pulse and oral temperature.
- Collection of urine for β-HCG pregnancy tests where applicable.
- Blood collection – approximately 7 mL whole venous blood for the determination of:
- 7 mL for a CBC, BUN, creatinine, ALT and AST. The safety samples will be appropriately packaged and sent to the appropriate lab.
- Immunization: IM administration of FMP2.1/AS01B or FMP2.1/AS02A candidate malaria vaccine in the deltoid muscle of the non-dominant arm.
- Distribution of diary cards and thermometers
  Participants will be instructed to come to the clinic on Days 1, 2, 3, 7 and 14 for evaluation of any adverse events occurring after immunization by the investigating team. They will be instructed to record oral body temperature and any local or general reactions observed after immunization on the diary cards for Days 1-7 following immunization.

Each vaccinee will be closely observed for at least 30 minutes following immunization after which blood pressure (BP), pulse, oral temperature, and solicited and unsolicited symptoms will be reviewed by the PI or his designee.

- Volunteers will be instructed to contact the investigator immediately should they manifest any signs or symptoms they perceive as serious.

**Visits 4-7: Days 1, 2, 3 & Day 7 ±3 days -Post immunization 1 follow-up visits**

- Recording of solicited and unsolicited symptoms and medication by the PI or his designee and recording of blood pressure (BP), pulse and oral temperature
- Check of elimination criteria
- Return of diary cards on Day 7 (thermometers kept for next immunization)

**Visit 8: Day 14 (±3 days) - Post immunization 1 follow-up visit**

- Check elimination criteria; record medications
- History directed physical examination including blood pressure (BP), pulse and oral temperature
- Record any unsolicited adverse events occurring after the immunization.
- Blood collection; approximately 27 mL:
- Approximately 7 mL whole venous blood will be collected, for a CBC, creatinine, BUN, ALT, AST). The safety samples will be appropriately packaged and sent to the appropriate lab.
- 20 mL venous blood will be drawn for humoral analyses (ELISA and GIA). The research samples will be centrifuged and the serum stored at ‑20°C (±5°C) or colder until processing.

**Visit 9: Day 28 (±3 days) - Immunization 2**

- Check elimination criteria, contraindications/precautions and record medications
- Record any unsolicited adverse events occurring after the first immunization.
- History, directed physical examination and recording of pre-immunization data including blood pressure (BP), pulse and oral temperature.
- Collection of urine for β-HCG pregnancy tests where applicable.
- Blood collection - approximately 27 mL whole venous blood:
- 7 mL for a CBC, BUN, creatinine, ALT and AST. The safety samples will be appropriately packaged and sent to the appropriate lab.
- 20 mL venous blood will be drawn for humoral analyses (ELISA and GIA). The research samples will be centrifuged and the serum stored at ‑20°C (±5°C) or colder until processing.
- Immunization: IM administration of malaria vaccine, in the deltoid muscle of the dominant arm.
- Distribution of new diary cards

Participants will be instructed to come to the clinic on Days 1, 2, 3, 7 and 14 post- immunizations for evaluation of any adverse events occurring after immunization by the investigating team. They will be instructed to record oral body temperature and any local or general reactions observed after immunization on the diary cards for Days 1-7 following immunization.

Each vaccinee will be closely observed for at least 30 minutes following immunization after which blood pressure (BP), pulse, oral temperature, and solicited and unsolicited symptoms will be reviewed by the PI or his designee.

- Volunteers will be instructed to contact the investigator immediately should they manifest any signs or symptoms they perceive as serious.

**Visits 10-13: Days 29, 30, 31 & Day 35 ±3 days - Post immunization 2 follow-up visits**

- Recording of solicited and unsolicited symptoms and medication by the PI or his designee and recording of blood pressure (BP), pulse and oral temperature
- Check of elimination criteria; recording of medications
- Return of diary cards on Day 35

**Visit 14: Day 42 (±5 days) – Post immunization 2 follow-up visit**

- Record any unsolicited adverse events occurring after the second immunization.
- Check elimination criteria; recording of medications
- History, directed physical examination including blood pressure (BP), pulse and oral temperature
- Blood collection; approximately 27 mL
- 7 mL whole venous blood will be collected, for a CBC, creatinine, BUN, ALT, AST). The safety samples will be appropriately packaged and sent to the appropriate lab.
- Approximately 20 mL whole venous blood for humoral analyses (ELISA and GIA). The research samples will be centrifuged and the serum stored at ‑20°C (±5°C) or colder until processing.

**Visit 15: Day 56 (±7 days) - Immunization 3**

- Check elimination criteria, contraindications/precautions, record medications
- Record any unsolicited adverse events occurring after the second immunization.
- History, directed physical examination and recording of pre-immunization data including blood pressure (BP), pulse and oral temperature
- Collection of urine for β-HCG pregnancy tests where applicable.
- Blood collection - approximately 34 mL of whole venous blood:
- 7 mL for a CBC, BUN, creatinine, ALT, and AST. The safety samples will be appropriately packaged and sent to the appropriate lab.
- 7 mL for microarray analysis (Cohort 2 only).
- Approximately 20 mL whole venous blood for humoral analyses (ELISA and GIA). Samples will be centrifuged and the serum stored at ‑20°C (±5°C) or colder until processing.
- Immunization: IM administration of malaria vaccine, in the deltoid muscle of the non-dominant arm
- Distribution of new diary cards.
  Participants will be instructed to come to the clinic on Days 1, 2, 3, 7 and 14 for evaluation of any adverse events occurring after immunization by the investigating team. They will be instructed to record oral body temperature and any local or general reactions observed after immunization on the diary cards for Days 1-7 following immunization.

Each vaccinee will be closely observed for at least 30 minutes following immunization after which blood pressure (BP), pulse, oral temperature, and solicited and unsolicited symptoms will be reviewed by the PI or his designee. Volunteers will be instructed to contact the investigator immediately should they manifest any signs or symptoms they perceive as serious.

- Volunteers will be instructed to contact the investigator immediately should they manifest any signs or symptoms they perceive as serious.

**Visits 16-19: Days 57, 58, 59 & Day 63 ±7 days - Post immunization 3 follow-up visits**

- Recording of solicited and unsolicited symptoms and medication by the PI or his designee and recording of blood pressure (BP), pulse and oral temperature.
- Check of elimination criteria; recording of medications
- Return of diary cards on Day 63
- On Days 57 and 59 only, 7 mL of whole blood for microarray analysis will be drawn for Cohort 2.

**Visit 20: Day 70 (±7 days) - Post immunization 3 follow-up visit**

- Check elimination criteria; record medications
- History, directed physical examination including blood pressure (BP), pulse and oral temperature
- Record any unsolicited adverse events occurring after the third immunization.
- If the Day 70 visit occurs at the same day as Day of Challenge, immunized volunteers will have all laboratory work due for both Day 70 and Day of Challenge drawn at the same time (See Section 5.4.2).
- Blood collection - approximately 157 mL whole venous blood:
- 7 mL for a CBC, BUN, creatinine, ALT, and AST. The safety samples will be appropriately packaged and sent to the appropriate lab.
- Approximately 20 mL whole venous blood for humoral analyses (ELISA and GIA). Samples will be centrifuged and stored at 20-25°C until transported to laboratory.
- Approximately 130 mL whole venous blood for CMI determination. Samples will be stored at 20-25°C until transported to CMI laboratory. No CMI labs for infectivity controls will be drawn.

**Table 1: Procedures of immunization** phase of study

| ***Phase*** | ***Screening*** | ***Phase Immunization Phase*** | | | | | | | | | |
| --- | --- | --- | --- | --- | --- | --- | --- | --- | --- | --- | --- |
| ***Day*** | ***-90 to -3*** | ***-90 to -3*** | ***0*** | ***1-3 &7*** | ***14*** | ***28*** | ***29-31  & 35*** | ***42*** | ***56*** | ***57-59 &63*** | ***70*** |
| ***Visit*** | ***1*** | ***2*** | ***3*** | ***4-7*** | ***8*** | ***9*** | ***10-13*** | ***14*** | ***15*** | ***16-19*** | ***20*** |
| ***Immunization*** |  |  | ***1*** |  |  | ***2*** |  |  | ***3*** |  |  |
| ***Informed consent (IC)*** | ***•*** |  |  |  |  |  |  |  |  |  |  |
| ***IC for HIV testing*** | ***O*** |  |  |  |  |  |  |  |  |  |  |
| ***Check of inclusion criteria*** | ***•*** | ***O*** | ***O*** |  |  |  |  |  |  |  |  |
| ***Check of exclusion criteria*** | ***•*** | ***O*** | ***O*** |  |  |  |  |  |  |  |  |
| ***Check of elimination criteria*** |  |  |  | ***O*** | ***O*** | ***O*** | ***O*** | ***O*** | ***O*** | ***O*** | ***•*** |
| ***Check of contraindications/ precautions*** |  |  | ***O*** |  |  | ***O*** |  |  | ***O*** |  |  |
| ***Medical history*** | ***•*** |  |  |  |  |  |  |  |  |  |  |
| ***Physical examination^6^*** | ***•*** |  | ***O*** |  | ***O*** | ***O*** |  | ***O*** | ***O*** |  | ***O*** |
| ***Pre- & post-immunization assessment incl. BP, pulse & temp measurement*** |  |  | ***•*** |  |  | ***•*** |  |  | ***•*** |  |  |
| ***Distribution of diary cards*** |  |  | ***O*** |  |  | ***O*** |  |  | ***O*** |  |  |
| ***Return of diary cards*** |  |  |  | ***O^2^*** |  |  | ***O^2^*** |  |  | ***O^2^*** |  |
| ***Post-immunization recording of solicited/unsolicited symptoms by PI*** |  |  |  | ***•*** |  |  | ***•*** |  |  | ***•*** |  |
| ***Recording medication*** | ***•*** |  | ***•*** | ***•*** | ***•*** | ***•*** | ***•*** | ***•*** | ***•*** | ***•*** | ***•*** |
| ***Recording of unsolicited AEs since previous visit, by PI*** |  |  |  | ***•*** | ***•*** | ***•*** | ***•*** | ***•*** | ***•*** | ***•*** | ***•*** |
| ***Reporting of serious AEs throughout the study by PI*** |  |  | ***•*** | ***•*** | ***•*** | ***•*** | ***•*** | ***•*** | ***•*** | ***•*** | ***•*** |
| ***Laboratory analyses*** |  |  |  |  |  |  |  |  |  |  |  |
| ***Screening (20 mL blood):***   - ***HIV 1, HBsAg, anti-HCV*** - ***Glucose, electrolytes, AP, LDH, bilirubin, BUN, creatinine, AST, ALT, CBC*** | ***•*** |  |  |  |  |  |  |  |  |  |  |
| ***Urinalysis*** | ***O*** |  |  |  |  |  |  |  |  |  |  |
| *β****-HCG –urine*** | ***●*** |  | ***•*** |  |  | ***•*** |  |  | ***•*** |  |  |
| ***Biochemical analysis  (7 mL blood):*** |  |  |  |  |  |  |  |  |  |  |  |
| ***CBC, BUN, creatinine, AST, ALT*** |  |  | ***•*** |  | ***•*** | ***•*** |  | ***•*** | ***•*** |  | ***•*** |
| ***Antibody response and cytokine assays :*** |  |  |  |  |  |  |  |  |  |  |  |
| ***- anti-AMA-1 (10 mL blood)*** |  | ***●^1^*** |  |  | ***●*** | ***•*** |  | ***●*** | ***•*** |  | ***●*** |
| ***-GIA (10 mL blood)*** |  | ***●^1^*** |  |  | **●** | ***•*** |  | **●** | ***•*** |  | ***●*** |
| ***Microarray analysis (7 mL)*** |  | ***●^1,4^*** |  |  |  |  |  |  | ***●^4^*** | ***●^3,4^*** |  |
| ***CMI (130 mL blood)**** |  | ***●^1^*** |  |  |  |  |  |  |  |  | ***•*** |
| ***Blood volume in mL^5^:*** | ***20*** | ***157*** | ***7*** | ***0*** | ***27*** | ***27*** | ***0*** | ***27*** | ***34*** | ***14*** | ***157*** |
| ***Cumulative blood volume^5^*** | ***20*** | ***177*** | ***184*** | ***184*** | ***211*** | ***238*** | ***238*** | ***265*** | ***299*** | ***313*** | ***470*** |

***• indicates a study procedure that requires documentation in the CRF and O indicates a study procedure that does not.***

***^1^initial CMI, humoral, and microarray analysis will be drawn at Visit 2 of screening phase for enrolled volunteers only***

***^2^Return of diary cards will occur on Days 7, 35, 63***

***^3^Analyses will only be performed on Day 57 and Day 59***

***^4^Cohort 1 volunteers will not have microarray analyses done, therefore blood volumes will be decreased accordingly***

***^5^All blood volumes are approximate***

***^6^While physical examination will occur at indicated visits, vital signs (blood pressure, pulse and temperature) are taken at all visits and recorded on source documents. Vital signs taken at the screening visit and 3 immunization days will be recorded on the CRF.***

| **Visit Intervals (Immunization phase)** | | **Size of interval** |
| --- | --- | --- |
| Screening → Day 0 | 3 to 90 days | |
| Visit 3 (Day 0) → Visit 8 (Day14) | 11 to 17 days | |
| Visit 3 (Day 0) → Visit 9 (Day 28) | 25 to 31 days | |
| Visit 3 (Day 0) → Visit 14 (Day 42) | 37 to 47 days | |
| Visit 3 (Day 0) → Visit 15 (Day 56) | 49 to 63 days | |
| Visit 3 (Day 0) → Visit 20 (Day 70) | 61 to 79 days | |

**All “low dose” (10 µg FMP2.1/AS01B) vaccinees as well as the “full dose” (50 µg FMP2.1/AS01B or AS02A) vaccines not undergoing sporozoite challenge will have a final study visit on Day 86 (Visit 21) to coincide with Day 30 post-third immunization. This visit will take place at the CTC, WRAIR. The procedures planned for these volunteers on Day 86 will differ from the procedures planned on Day 86 for volunteers undergoing malaria challenge.**

**Visit 21: Day 86 (±5 days) – Post immunization 3 follow-up visit and final study visit for volunteers not undergoing malaria challenge**

- Record any unsolicited adverse events occurring after the third immunization.
- Check elimination criteria; recording of medications
- History, directed physical examination including blood pressure (BP), pulse and oral temperature
- Collection of urine for β-HCG pregnancy tests where applicable.

### Challenge phase

- The primary malaria challenge is scheduled for Cohort 2 approximately 14 - 30 days after the third immunization if 6 or more volunteers from each group go forward to challenge. This may coincide with the immunized volunteers Day 70 visit. The laboratory analyses are similar for both of these visits. If the challenge occurs within the Day 70 visit window, immunized volunteers may have all laboratory work due for both Day 70 and Day of Challenge drawn at the same time.
- Infectivity control volunteers are recruited and screened in an identical manner as those volunteers recruited for the immunization phase. The exception is the study specific informed consent (Appendix H), which is unique to the control volunteer. During the hotel phase, all challenged volunteers will be assessed on a daily basis in an identical manner. Following completion of the hotel phase, any control participants who have not yet developed malaria will be assessed in a manner identical to that of the remaining challenged but protected vaccinees.
- The post challenge schedule is identical for all participants undergoing sporozoite challenge (vaccinees and infectivity controls) with the exception that there will be no blood draws for CMI analysis on the infectivity controls.
- Upon entry into the challenge phase of the study, all volunteers will be issued a study identification card. This has the volunteer’s name on it, details their exposure to malaria and has 24-hour emergency telephone contact numbers for study investigators.
- Once challenge has occurred, the volunteers will be instructed to contact the investigator immediately should they feel unwell.
- If a volunteer is found to be parasitemic, antimalarial treatment with oral chloroquine (see section 5.2.6.4) will be initiated immediately under direct observation of the physician.

The challenge will be performed according to the following procedures:

**Visit 21: Day of Challenge (DOC) - (+14 to 30 days after 3^rd^ immunization)**

- Check elimination criteria, and check contraindications to challenge
- Record medication until 30 days after last immunization
- History, directed physical examination
- Record any unsolicited adverse events occurring up to 30 days after the last immunization
- Record pre-challenge data including oral temperature, pulse, and blood pressure.
- Collection of urine for β-HCG pregnancy tests where applicable.
- Blood collection; approximately 36 mL of whole venous blood:
- 7 mL for CBC, BUN, creatinine, ALT and AST. The safety samples will be appropriately packaged and sent to the appropriate lab.
- 20 mL for humoral analyses (ELISA and GIA). The research samples will be centrifuged and the serum stored at ‑20°C (+/- 5°C) or colder until processing.
- 7 mL for microarray analysis
- 2 mL for quantitative PCR testing
- Infectivity Controls only (in addition to humoral assays and PCR)
- 4 mL for complement assays (2 mL EDTA tube and 2 mL red top)
- 20 mL for NK cell assays
- Sporozoite challenge - For each volunteer, five mosquitoes will be allowed to feed on the forearm of the volunteer over five minutes. These mosquitoes will be dissected to confirm they were infected. If all five are not adequately infected, additional mosquitoes will be allowed to feed to ensure a total of five infected mosquitoes are used.

**Each volunteer will be closely observed for at least 30 minutes following the challenge, after which blood pressure, pulse and oral temperature measurements will be taken. Any severe reactions will be reviewed by the PI and Independent Medical Monitor and noted in the volunteer case report forms*.***

**Volunteers will be instructed to contact the investigator immediately should they feel unwell.**

**Day 1 - Post-challenge follow-up**

- Participants will be required to come to the clinic on day 1 post-challenge for blood collection, approximately 9 mL of whole venous blood:
- 7 mL will be taken for microarray analysis
- 2 mL will be taken for PCR testing
- 4 mL will be taken for complement testing in infectivity controls

**Days 5-9 - Daily post-challenge clinic follow-up visits**

- History, directed physical examination including blood pressure (BP), pulse and oral temperature
- Blood collection:
- 2 mL whole venous blood for malaria smear every day
- 7 mL of blood on Day 5 post challenge for microarray analysis
- 2 mL of blood for quantitative PCR testing on days 5, 7, 9 post-challenge
- On day 7 post-challenge, 20 mL will be taken for humoral analyses (ELISA and GIA). The research samples will be centrifuged and the serum stored at ‑20°C (+/- 5°C) or colder until processing.
- 4 mL of blood for complement testing on Day 5, 7, 9 for infectivity controls only

**Days 10-20 - Post-challenge: Hotel Phase**

- Participants will be required to check into a designated hotel in the general vicinity of WRAIR every evening for approximately 10 nights. Investigators will be on call and available to provide medical care 24 hours each day. All volunteers will be contacted by telephone each afternoon until they become positive for malaria. They will be asked the same assessment questions that were asked in the morning evaluation: presence of malaria-specific symptoms such as fever, chills, sweats, mylagias, headache, nausea, anorexia, diarrhea, vomiting, arthralgias, low back pain, or any other symptoms.
- Daily history and directed physical examination will be performed.
- Daily blood collection:
- Approximately 2 mL whole venous blood for malaria smear. Approximately 2 mL whole venous blood for PCR testing until third negative blood smear
- Approximately 4 mL whole venous blood for complement testing until parasitemia detected
- Additional examinations (e.g., malaria smears, CBC) will be performed should a participant exhibit any symptoms suggestive of malaria. The range of clinical tests performed will be at the discretion of the clinical investigators.
- If a participant is found to be parasitemic:
- CBC will be performed within 12-24 hours of the time of diagnosis
- In infectivity controls, 20 mL of blood will be drawn at time of diagnosis
- Antimalarial treatment with oral chloroquine (see section 5.2.6.4) will be initiated immediately under direct observation.
- Daily malaria smears will continue until three consecutive daily blood films are negative. After the third negative smear, the investigator may excuse the volunteer from nightly check-in at the hotel.

A blood sample will be drawn for PCR on day 3 of diagnosis and thereafter daily blood collections for PCR testing will stop***.***

**Day 21-30 – Post-challenge visits for malaria-negative individuals**

Volunteers who have remained negative for malaria during the hotel stay will be seen in the Dept. of Clinical Trials during the week (Monday through Friday -weekends and holidays excluded unless a visit is determined to be necessary by PI) for evaluation and blood smear. If a volunteer does not keep a scheduled appointment, they will be contacted by telephone. Volunteers will begin weekly follow-up after Day 30.

Owing to the potential logistical difficulty incurred by the requirement of daily blood smears, asymptomatic volunteers with persistently negative smears may, be excused from daily phlebotomy after post-challenge Day 20 at the discretion of the Investigator. Additional examinations (e.g. malaria smears, CBC) will be performed should a participant exhibit any symptoms suggestive of malaria. The range of clinical tests performed will be at the discretion of the Investigator.

**Weekly post-challenge clinic follow-up visits
Post challenge Week I/Week II/Week III/Week IV (±3 days)**Note: Visit dates are based on either the third negative smear for the malaria positive volunteer, or 30 days post challenge for the malaria negative volunteer.

- History, directed physical examination
- Record BP, pulse and oral temperature
- Blood collection:
- 2 mL whole venous blood for malaria smear
- Approximately 20 mL of blood for humoral analyses (ELISA and GIA) at Week II (or 30 days post challenge). The research samples will be centrifuged and the serum stored at ‑20°C (+/- 5°C) or colder until processing.

**Post challenge clinic follow-up visit: Day 90 (±5 days)/Month 5.**

- History, directed physical examination
- Record BP, pulse and oral temperature
- Collection of urine for β-HCG pregnancy tests where applicable
- Blood collection - approximately 159 mL of whole venous blood:
- 2 mL for malaria smear
- 7 mL for a CBC, BUN, creatinine ALT, and AST. The safety labs will be appropriately packaged and sent to the appropriate lab.
- 20 mL for humoral analyses (ELISA and GIA). The research samples will be centrifuged and the serum stored at ‑20°C (± 5°C) or colder until processing.
- 130 mL for CMI determination. Samples will be stored at 20-25°C until transported to CMI laboratory. No CMI labs will be drawn for infectivity controls.

Table 2 outlines the procedures during the challenge phase of the study. Key intervals between visits are outlined below.

| **Visit Intervals (challenge phase)** | | **Size of interval** |
| --- | --- | --- |
| Visit 15 (Day 56) → Day of challenge visit (Month 2) | 14 to 28 days | |
| Day of challenge visit → 3 months Post challenge visit | 79 to 89 days | |

Table 2: Procedures during challenge phase of study

| **Phase** | **Challenge Phase** | | | | | | | | | | | | | |
| --- | --- | --- | --- | --- | --- | --- | --- | --- | --- | --- | --- | --- | --- | --- |
| Timings (Months) | M 2 |  | | | | | | | | | | | | M 5 |
| Timings (Days/Weeks) | D0 | D1 | D5 | | D6 | D7 | D8 | D9 | *D10 to D20** | Wk 1 | Wk 2 | Wk 3 | Wk 4 | D90 |
| Event | **DOC  (Day 56 + 14-28 days)** | **Post Challenge** | | | | | | | **Hotel** | **Post Hotel** | | | | |
| Check of elimination criteria &contradictions to challenge | ***O*** |  | |  |  |  |  |  |  |  |  |  |  |  |
| Reporting of serious AEs | • | • | | • | • | • | • | • | • | • | • | • | • | • |
| Physical examination | ***O*** |  | | O | O | O | O | O | O | O | O | O | O | O |
| BP, pulse, oral temperature | • |  | | O | O | O | O | O | O | O | O | O | O | O |
| Recording medication | • | • | | • | • | • | • | • | • | • | • | • | • | • |
| **Laboratory analyses ^1^** |  |  | |  |  |  |  |  |  |  |  |  |  |  |
| β-HCG-urine | • |  | |  |  |  |  |  |  |  |  |  |  | ● |
| Biochemical analyses  (7 mL) CBC, BUN, creatinine, AST, ALT | • |  | |  |  |  |  |  |  |  |  |  |  | • |
| Antibody response  - Anti-AMA-1 (10 mL)  - GIA (10 mL) | **●**  **●** |  | |  |  | **●**  ● |  |  |  |  | **●**  **●** |  |  | **●**  **●** |
| CMI (130 mL) |  |  | |  |  |  |  |  |  |  |  |  |  | •^1^ |
| Genomic analysis (7 mL) | **•** | **•** | | **•** |  |  |  |  |  |  |  |  |  |  |
| Complement assays (4 mL) | **●** | **●** | | **●** |  | **●** |  | **●** | **●** |  |  |  |  |  |
| NK cell assays (20 mL) | **●** |  | |  |  |  |  |  | **●** |  |  |  |  |  |
| PCR (2 mL) | **●** | **●** | | **●** |  | **●** |  | **●** | **●** |  |  |  |  |  |
| Malaria smear (2 mL) |  |  | | ● | ● | ● | ● | ● | ●^4^ | • | • | • | • | • |
| Blood volumes (mL) | 36 | 9 | | 11 | 2 | 24 | 2 | 4 | 11 x 4=44^2^ | 2 | 22 | 2 | 2 | 159 |
| Cumulative blood volume (mL)^3^ - controls | 60 | 73 | | 88 | 90 | 118 | 120 | 128 | 192^5^ | 194 | 216 | 218 | 220 | 247 |
| Cumulative blood volume (mL) ^3^ – immunized vols | 36 | 45 | | 56 | 58 | 82 | 84 | 88 | 132^2^ | 134 | 156 | 158 | 160 | 319 |

• indicates a study procedure that requires documentation in the CRF. O indicates a study procedure that does not require documentation in the CRF. D = Day; Wk = week: M= Month
^1^This schedule is identical for all participants undergoing sporozoite challenge (vaccinees and infectivity controls) except CMI analyses, complement and NK cell assays
^2^Blood samples on days 10-20 post challenge are estimated on 11 blood samplings during the period. For days 21 to start of Week 1 only protected participants may be required to provide blood for malaria smears
^3^Note that the cumulative blood volume does not take into account above described blood smears nor cbcs taken when participant becomes parasitemic and approximately 4 weeks after drug cure of malaria infection

^4^All smears will be recorded on CRFs.

^5^ Estimates control volunteers are parasitemic by Day 13 (smears done until D17, PCR & complement assys until D14, NK assays once)

Sample handling and analysis

### Transport of biological samples

Transport of biological samples will be done according to the WRAIR Transport SOP.

### Laboratory assays

All screening and follow-up safety laboratory testing will be performed either in the Clinical Laboratories of Walter Reed Army Medical Center, the National Institutes of Health or at an outside approved contract clinical laboratory. The WRAIR sub-investigators will maintain detailed SOPs (or the administrative equivalent) for immunological assays on site at WRAIR. The sera designated for transport to NIH for GIA will be transported according to the Transport SOP guidelines and the assay performed according to NIH GIA SOP. Urine pregnancy tests will be performed in the WRAIR Department of Clinical Trials.

As indicated in the objectives, assays will be conducted to assess safety as well as immunogenicity. The primary assay currently used to assess immunogenicity is an antibody ELISA. This study plans to run additional assays to evaluate the study participants’ immune responses, and these assays will be prioritized according to current and future needs of the study as well as volume of sample available.

***Screening*** Tests will be performed to assay for HBsAg (includes reflex testing for any positive results); antibodies against HIV-1, and HCV; complete blood count (CBC), urine pregnancy test (for females), glucose, electrolytes, BUN, creatinine, ALT, AST, LDH, alkaline phosphatase, bilirubin.

***Safety*** Tests will be performed to assay for CBC, BUN, creatinine, ALT, and AST at the following time points
-*Immunization phase(Cohorts 1 & 2)*: Days 0, 14, 28, 42, 56 & 70
-*Challenge phase (Cohort 2)*: Day of challenge (Month 2), day of first parasitemia (CBC only), and Day 90 Post day of challenge (Month 5)

***Serology***Separation of serum from the venous blood will be performed at the WRAIR, and serum frozen at -70° C. No personal identifying information will be indicated on any sample or vial other than the volunteer code, study number and date within the study. One mL of serum will be designated for testing of anti-AMA-1 antibodies via standardized AMA-1 ELISA in the WRAIR Malaria Serology Laboratory (MSL) at time points indicated below. Two mL of serum will be designated for pLDH GIA (both homologous and heterologous) at WRAIR.

For exploratory GIA assays, one ml will be designated for the WRAIR laboratory and two-three mL of serum will be sent to MVDU/NIH for GIA. For exploratory assays IFA and PIA, approximately 1.0 mL of serum will be needed and will be provided given the ELISA and GIA assays have sufficient sera.

Time points for ELISA are as follows:

*-Immunization phase (Cohorts 1 & 2)*: Days 0, 14, 28, 42, 56, 70

-*Challenge Phase (Cohort 2)*: Day of Challenge (Month 2), Day 7 post-challenge, approximately Day 30 post-challenge (Week I or II), Day 90 post-challenge (Month 5)

Time points for pLDH GIA are as follows:

-*Immunization Phase (Cohort 1 &2)*: Days 0, 14, 28, 42, 56, 70

-*Challenge Phase (Cohort 2)*: Day of Challenge (Month 2), Day 7 post-challenge, approximately Day 30 post-challenge, Day 90 post-challenge (Month 5)

However, performing testing at time points less frequent than indicated is at the discretion of the researcher conducting such tests.

Time points for exploratory assays IFA and PIA are as follows:

*Immunization Phase:* Day 70 (must be pre-challenge if Day 70 and DOC are the same)

*Challenge Phase*: Day 30 post-challenge (week I or II) Details of the serological assays can be found in Appendix D and an overview is given in Table 3.

Table 3: Overview of serological assays (anti-FMP2.1)

| **Assay type** | **Marker** | **Test Kit/ Manufacturer** | **Assay unit** | **Assay cut-off** | **Laboratory** |
| --- | --- | --- | --- | --- | --- |
| ELISA | anti-FMP2.1 Ab | In house | μg/mL | 1 μg/mL | WRAIR |
| GIA | pLDH | In house | % |  | WRAIR |
| PIA | AMA-1 processing inhibition Abs | In house | 10-kDa band / (10-kDa +20-kDa bands) |  | WRAIR |
| IFA | Anti-AMA-1 Ab | In house | +/- |  | WRAIR |

***CMI***

Whole blood samples for cell-mediated immunity assays will be collected and kept at 20-25°C until transfer to the immunogenicity laboratory for isolation of peripheral blood mononuclear cells (PBMC). Main tests to be performed are:

- IFNγ determinations by the ELISPOT assay
- Intracellular Cytokine Staining (ICS) assay characterizing Ag-induced CD4/CD8 T cells expressing IFNγ and/or IL-2 and/or TNFα

Blood for CMI will be collected:

- at baseline (prior to the start of immunization),
- two weeks after third immunization (Day 70 ±5),
- Cohort 2 only -approximately 3 months post challenge

No CMI testing will be performed on infectivity controls.

***Exploratory analyses***

Additional exploratory analyses that may be performed if feasible are:

- Merozoite immunoflouresence assay (IFA) to verify ability of antibodies to recognize AMA-1 in infected erythrocytes
- Processing inhibition assay (PIA) to assess ability of antibodies to inhibit processing of 66-kDa AMA-1 molecule
- ELISA determination of anti-AMA-1 titers (FVO variant), subdomain ELISAs for internal domains of the AMA-1 molecule, epitope mapping, specific antibody isotype analysis and antigen add-back confirmatory GIA, and 2 differing methods of GIA developed by WRAIR and NIH researchers
- Microarray analysis to assess differential levels of gene(s) expression

Level of parasitemia induced by sporozoite challenge as measured by quantitative PCR

- Whole blood assay to characterize CD4/CD8 T cells and IFNγ expression
- Particle-based assay (*Luminex)* for cytokine testing in PBMC culture supernatant
- Determination of Complement Protein and Complement Regulatory Protein Levels on Erythrocytes
- Natural Killer (NK) cell assays measuring IFN-gamma secretion
- Sporozoite immunofluoresence assay (IFA) to verify ability of antibodies to recognize AMA-1 on *P. falciparum* sporozoites

CMI and exploratory testing will be carried out at the WRAIR laboratory with the exception of one method of GIA analysis which will be conducted at the Malaria Vaccine Development Unit, NIH. Any additional laboratory assays will be performed if deemed relevant if any findings in the present study or in other studies necessitates investigation of the toxicity or immunogenicity of the vaccine.

### Rationale for exploratory laboratory analyses

**Merozoite Immunofluorescence Antibody test (IFA)**

While vaccines may generate antibodies to recombinant AMA-1, the ability of these antibodies to bind to native parasite AMA-1 cannot be evaluated by ELISA. An IFA test will be conducted to determine this. Parasitized red blood cells will be fixed to a glass slide. Appropriate dilutions of antibodies from study volunteers at different time points will be incubated on the slide, and these will be detected using an anti-human antibody conjugated with a fluorescent tag. By examining the slide under an ultraviolet microscope, the ability of the antibodies to recognize AMA-1 will be assessed by the amount of fluorescence seen and the pattern of fluorescence characterized.

**Processing Inhibition Assay (PIA)**

Researchers at WRAIR have developed a processing inhibition assay (PIA) that helped elucidate actual inhibition mechanisms utilized by the antibodies. For the AMA-1 molecule to be effective, it must be processed first from an 83-kDa molecule located in the micronemes, to a 66-kDa fragment, which then can translocate to the surface of the merozoite. Further C-terminal cleavage produces two other forms: 48-kDa and 44-kDa. The PIA will be conducted essentially as described previously^9^. This assay will measure the abilities of the antibodies generated post-third immunization and post-challenge to prevent the above-described processing of AMA-1.

**Sporozoite Immunfluorescence Assay (sporozoite IFA)**

The presence of AMA-1 on the surface of merozoites is well characterized, and immunological studies have focused on the humoral response to AMA-1 during the blood stage of infection. However, work done by Silvie et al.^5^ has demonstrated the presence of AMA-1 in sporozoites by reverse transcriptase PCR as well as with immunofluorescence assays. Immunofluorescence localization experiments determined that AMA-1 was concentrated in the apical micronemes of sporozoites, and Western blots confirmed the presence of two proteins: one of 83kDa which corresponds to the full length AMA-1 protein, and one of 66 kDa which corresponds to the protein after the first proteolytic cleavage when AMA-1 localizes to the surface of sporozoites. When *P. falciparum* was cultured with primary human hepatocytes lines, AMA-1 was not detected in the hepatocytes at early liver stage (Day 3 post-infection), but was detected on hepatic merozoites in the liver schizont stage (Day 10). In 2 selected volunteers in the Phase 1 dose escalation trial at WRAIR (MAL-033), immunoflouresence of sporozoites was demonstrated with antibodies induced by the FMP2.1/AS02A vaccine. Since AMA-1 can be demonstrated in three different stages of *P. falciparum*, immune responses generated by an AMA-1 vaccine have the potential to inhibit parasite growth at each distinct stage. Conducting a sporozoite IFA will assess whether antibodies generated by the FMP2.1/AS02A or FMP2.1/AS01B vaccine are specific for the AMA-1 localized on sporozoites, and thus have the potential to inhibit or restrict the invasion of hepatocytes and abrogate the course of infection.

**Additional exploratory serologic assays**

This study plans to assess anti-AMA-1 antibody responses to only the 3D7 variant as described in Section 5.5.2 since the vaccine is based on this variant. However in the field there is antigenic variation seen between parasite strains. Thus, assessing serologic response to the other common AMA-1 allelic variant, FVO, would be of interest, particularly as previous studies in mice and rabbits have shown that while immunization does induce high antibody titers to the homologous variant, titers to the heterologous variant are significantly less^12-13^. Similarly, some evidence is emerging in endemic populations that the isotype of the antibody generated may be as important as the overall magnitude of antibody response. Should this observation be repeated in other studies, determining antibody isotypes may become a readout of interest. In addition, if good parasite growth inhibition is demonstrated by GIA in some volunteers, it would be useful to confirm that the inhibition seen is due to a specific antibody induced by the vaccine and not non-specific toxicity. By adding excess recombinant AMA-1 to the sera while performing the assay, if the antibody is adsorbed to the free antigen, the growth inhibition should be drastically reduced by this add-back procedure. The ability of sera to inhibit parasite growth as measured by the standardized pLDH GIA assay will provide information on the immunogenicity of the two candidate vaccines. The two GIA exploratory assays developed by researchers at WRAIR and NIH have additional steps as compared to pLDH GIA, thus with possible improvements in methods. The assay developed at NIH has a preliminary immunoglobulin purification step, and the second WRAIR assay evaluates assay results when done with very small amounts of sera, and is also currently being developed to detoxify sera to improve results. Ongoing research will help indicate which among these serological exploratory assays may be most useful either as a potential correlate of protection, or as useful in assisting with future vaccine formulations, scheduling regimens or overall vaccine development strategies.

**Microarray Analysis**

Samples for microarray analysis will be taken during the immunization and challenge phase only for volunteers in Cohort 2. Blood samples will be drawn on Day 0, Day 56 (immunization #3), Day 57, Day 59, day of challenge (DOC) and post-challenge days 1 and 5. The blood samples will be used for the identification of possible patterns of gene expression associated with protection. The justification for molecular profiling of immune responses after FMP2.1 immunization using high-density oligonucleotide microarrays is as follows:
The identification of immune correlates of protection induced by immunization has not been shown to fully correlate with any single humoral or cell-mediated immunological assay to-date. The simultaneous surveillance of gene expression in thousands of human genes after an intervention (i.e. immunization) reveals patterns of expressed and repressed genes when compared to basal levels of gene expression. We hypothesize that by comparing molecular profiles from volunteers protected from *P. falciparum* malaria mosquito challenge with profiles elicited from volunteers not protected after immunization would reveal patterns of gene expression associated with protection. We plan on using expression profiling to analyze total white blood cells collected from volunteers prior to immunization, on Days 57 and 59, on the Day of Challenge and on Days 1, 5, 7, 9 post challenge in order to assess the transcriptional responses that occur and that correlate with the particular class (protected vs. non-protected; FMP2.1/AS01B vs. FMP2.1/AS02A). Pattern recognition software, which utilizes both supervised learning (artificial neural networks) and unsupervised clustering algorithms, will be employed to cluster genes according to outcome. The molecular profiling encompasses the identification of RNA transcripts present in all humans, which are induced or repressed after intervention (immunization and malaria challenge), and DOES NOT represent genetic testing of individuals or their DNA. For all participants, 7 mL of whole blood will be collected in CPT vacutainer tubes (Becton Dickinson, Inc.) by peripheral venipuncture at the time points indicated above.

**Quantitative Real-Time PCR**

New developments in PCR technology have allowed for quantification of genomic information in biological samples. Quantitative PCR can be used to assess malaria infection by amplifying the 18s ribosomal subunit RNA gene of *Plasmodium falciparum* and determining quantity of *P. falciparum* copies by detection of a fluorescent probe and comparison to a titrated standard curve of known parasite number. This method has been shown to be more sensitive in detecting copies of *P. falciparum* in venous blood samples than blood films or clinical signs or symptoms^35^. In addition, blood films can be highly microscopist-dependent. In malaria vaccine trials of malaria-naïve adults, quantitative PCR detected levels of 20 parasites/mL compared to blood film results of >1,250 parasites/mL^36^. Following parasite copy numbers as determined by this method of PCR may allow for a more accurate assessment in changes in the prepatent period after immunization with either FMP2.1/AS01B or FMP2.1/AS02A that might not be evident by blood film. It also may also provide useful information on parasite sequestration and replication dynamics. Procedures for quantitative PCR are located in Appendix D.

**Intracellular Cytokine Staining (ICS) and Whole Blood assay**

Intracellular cytokine staining and flow cytometry are state-of-the-art analyses to assess cell-mediated immune responses. To perform ICS, the first step is PBMC isolation, with subsequent steps of the assay allowing for detection of cytokines within a lymphocyte. Developing similar assays that are field-adaptable is crucial to future vaccine studies planned in endemic countries where specialized labs and equipment may not be available. The whole blood assay is a test whereby test antigen is added directly to a recently drawn venous blood sample, and so laborious and expensive PBMC separation techniques are avoided. These tests can then go on to characterize by intracellular cytokine staining and flow cytometry CD4 versus CD8 cells as well as specific cytokines. Whole blood assays are currently undergoing preliminary usage in the MAL-044 malaria vaccine trial at the WRAIR research site in Kenya. Utilizing this assay for the Phase I/IIa FMP2.1/AS01B versus FMP2.1/AS02A vaccine trial provides an additional opportunity to compare whole blood assay results to intracellular cytokine staining assays and support their utility in future field studies.

**Detection of cytokines using particle-based assays (*Luminex)* technology**

Most malaria vaccine studies have used standard ELISA techniques to detect cytokine production, often focusing on IFNγ titers. It is unlikely that a single cytokine is representative of the full cytokine pattern secreted, but to analyze multiple cytokines simultaneously is labor-intensive and expensive. *Luminex* technology enables an investigator to determine the levels of multiple cytokines with a small serum sample (approximately 50-100 µL). The proposed investigation could determine whether cytokines and chemokines such as TNFα and IFNγ, IL-1b, IL-2, IL-4, IL-7, IL-13, IL-21, MIP, RANTES and others are associated with induction of immunity and/or systemic reactogenicity. This exploratory analysis may allow an assessment of whether this simple *ex vivo* measurement can be predictive of classical CMI evaluations. One could envision using this methodology for CMI evaluations in clinical studies where there may not be possible to sample appropriate volumes of blood or where processing PBMC are problem^37^. Samples could be collected as part of the blood collected for antibody determination to assess the immune response.

**Determination of Complement Protein and Complement Regulatory Protein Levels on Erythrocytes**

Hemoglobin degradation products (heme/hematin) released as a result of the rupturing of a *Plasmodium falciparum*-infected erythrocyte can trigger the activation of the complement protein pathway. Once the complement pathway is activated, complement proteins can interact and bind to targeted pathogens or cells nearby. Complement protein C3 forms a covalent bond to an available substrate thereby labeling it for destruction through the complement cascade or promoting the interaction of an effector (phagocytic) cell. This overall process may be a key mechanism in causing malaria-associated anemia. This assay will ascertain the extent of complement deposition as a function of parasitemia and level of complement regulatory proteins.

**Natural Killer Cell Assays**

In natural infection with to *P. falciparum*, Natural Killer (NK) cells are thought to be involved in the immune response by producing IFNγ following contact with parasitized red blood cells (pRBCs)^38^. The receptor(s) involved in this recognition are not known. Immunization with *P. falciparum* AMA-1 protein generates strong cell-mediated responses with the production of IFNγ and IL-5 (personal communication, U. Krzych). The contribution of NK cells to the production of IFNγ is not well-elucidated but may play an important role in immunity. Given the possible interaction between NK cells and *P. falciparum*, the following will be investigated:

1. Ability of NK cells to produce IFN-gamma in the presence of pRBCs.
2. Identification of the NK receptor involved in pRBC recognition by performing the assays in the presence or absence of various blocking antibodies directed against different NK markers.
3. Ex-vivo measurement of the level of inflammatory cytokines by intracellular staining and flow cytometry.

# Study Vaccines and Administration

## Study vaccines

The vaccine antigen FMP2.1 has been developed and manufactured by the WRAIR, Silver Spring, MD, USA and the adjuvants AS01B and AS02A by GSK Biologicals, Rixensart, Belgium. FMP2.1/AS01B formulation has not yet been used in human studies. The Quality Control Standards and Requirements for each candidate vaccine are described in separate release protocols and the required approvals have been obtained or will be obtained prior to the start of the study.

FMP2.1, is a recombinant subunit protein produced in, and purified from *E. coli* Tuner^TM^ cells by good manufacturing practices (GMP). The antigen consists of amino acids 83-531 representing the majority of the ectodomain of the 3D7 variant of AMA-1^17^. For this study, Lot 1046 of antigen will be used. The vaccine antigen will be reconstituted in the proprietary adjuvant AS01B (a formulation based on liposomes and two immunostimulants QS21 and MPL) or AS02A (an oil-in-water emulsion and the same two immunostimulants).

**Reconstitution in Adjuvant**

The lyophilized antigen will be reconstituted in the AS01B (Lot DA1BA002A) or AS02A (Lot DA2AA002A) adjuvant.

AS01B is a liposome-based adjuvant with 50 µg 3-D-MPL and 50 µg QS-21 immunostimulants per volume of 0.5 mL. All AS01B vials should be stored at 2°C – 8°C.

AS02A is based on an oil-in-water emulsion, 50 µg 3-D-MPL and 50µg QS-21 in phosphate buffered saline per volume of 0.5 mL. All AS02A pre-filled syringes (PFS) should be stored at 2°C - 8°C.

FMP2.1/AS02A and FMP2.1/AS01B will be formulated immediately prior to use.

## Dosage and administration

*Reconstitution of vaccine with AS01B or AS02A and administration*:

For Cohort 1, the reconstitution of FMP2.1/AS01B will be followed according to the Mixing SOP. Briefly, disinfect top of a vaccine vial with the antigen pellet with alcohol swabs - let dry. Aspirate the contents of the AS01B vial in a syringe and then inject adjuvant into the vial of lyophilized antigen and remove needle from vial. Repeat this procedure with 5 vials total, all injecting in to the same vial of lyophilized antigen. Dissolve the pellet by gently shaking the vial. Wait for approximately one minute to ensure complete dissolution of vial contents before withdrawing 0.5 mL of the reconstituted vaccine solution using a fresh needle and syringe. Two additional doses of 0.5 mL each may be removed from this same vial, for a total of 3 doses/antigen vial to ensure appropriate volume of 0.5 mL each withdrawal.

For Cohort 2, disinfect top of a vaccine vial with the antigen pellet with alcohol swabs - let dry.
For AS01B, aspirate the contents of the vial in a syringe and then inject adjuvant into the vial of lyophilized antigen and remove needle from vial. For AS02A, inject directly the contents of the PFS into the vial of lyophilized antigen. Remove needle from the vial Dissolve the pellet by gently shaking the vial. Wait for approximately one minute to ensure complete dissolution of vial contents before withdrawing remaining contents of the reconstituted vaccine solution using a fresh needle and syringe (approximately 0.5 mL).

The first immunization should be administered by slow intramuscular injection in the deltoid muscle of the non-dominant arm within four hours of reconstitution. Each subsequent immunization will be given in the deltoid of the alternating arm; e.g., left, right, left. If after inspection of the arm prior to immunization, a physical finding is discovered that will interfere with evaluation of vaccine reactogenicity, the unaffected arm will be used. If this occurs, attempts will be made to preserve an alternate arm schedule if possible.

The vaccinees will be observed closely for at least 30 minutes, with appropriate medical treatment readily available in case of a rare anaphylactic reaction following the administration of vaccines.

The vaccines will be administered in the facilities of the WRAIR Clinical Trials Department under the supervision of physicians skilled in the management of anaphylactic reactions.

## Storage

- All vials containing FMP2.1 lyophilized antigen must be stored at -20° C.
- All AS01B vials and AS02A PFS should be stored in the refrigerator (+2°C to +8°C) and must not be frozen.
- Storage temperatures should be recorded daily.

Records must be maintained that document receipt, release for immunization, disposal, or return to the manufacturer of all vaccine vials, adjuvant vials and pre-filled adjuvant syringes.

## Treatment allocation and randomization

The five volunteers enrolled in Cohort 1 ( 1/5^th^ dose of FMP2.1/AS01B) will not be randomized nor blinded to the vaccine administered as this cohort is necessary for the appropriate safety evaluation of this vaccine formulation prior to proceeding with a higher dose formulation of FMP2.1/AS01B. However, in Cohort 2, participants will be randomly assigned to one of 2 groups. From 6 to fifteen volunteers will be in the immunization Group A that receives 50µg dose of FMP2.1/AS01B and from 6 to fifteen volunteers will be in the immunization Group B that receives 50µg dose of FMP2.1/AS02A.

Volunteers will be considered as ready to be assigned to one of the vaccine groups as they complete the screening process and meet entry criteria. For Cohort 2, prior to the day of first immunization (Day 0), all 30 volunteers will be randomly allocated in a 1:1 fashion to one of the investigational groups (to receive FMP2.1/AS01B or FMP2.1/AS02A) in the order of which they are enrolled. A computerized randomization list will be generated by PI or designated representative from WRAIR and will be used to number the vaccines with a randomization number to uniquely identify each vaccine dose to be administered to a participant.

An Immunization Team will be created consisting of one physician and one study coordinator. These team members shall not be involved in the evaluation of vaccine safety and reactogenicity during the Immunization Phase. Two physicians shall be named to the Team in order that one is guaranteed to be available for vaccine administration at all time points. The Team will be responsible for mixing the vaccine and adjuvant as described in Section 6.2 and having the preparation ready for injection. The Team will also fill out a Vaccine Administration Record for each volunteer and each vaccine injection, recording volunteer study number, date, vaccine, adjuvant, amount injected/dose, and injection site. The Immunization Team will apply opaque taping to the outside of the syringes after vaccine preparation to insure that the contents of the vaccine are not visible to the volunteer or person administering the vaccine. For each subsequent immunization, the Team will be responsible for assuring that each volunteer receives the same adjuvant as previously administered.

After the immunization phase of the study, a minimum of 6 up to10 of the volunteers from Group A and from 6 to 10 of the volunteers from Group B will be eligible to proceed to sporozoite challenge. (The volunteers from Cohort 1 will not proceed to challenge.) These volunteers will be selected from the volunteers who remain enrolled in the study after three immunizations, having completed all study visits and complied with all study procedures. Volunteers can refuse to go on to the Challenge Phase at any time prior. If less than ten volunteers (in each group) remain, and they number at least six, all will be invited to go on to the Challenge Phase. If the number of volunteers after three immunizations numbers more than 10, a challenge randomization list will be generated by a non-blinded physician or study personnel who is neither the Principal Investigator nor associate Investigator in order to randomly select the ten volunteers from each Group. If less than six volunteers in a group remain after three immunizations, then no sporozoite challenge will be administered for that group.

Six non-randomized infectivity controls will be recruited specifically for each challenge. Each challenge is planned to be conducted over a one day period.

## Method of blinding and breaking the study blind

As the adjuvants are different in presentation (vials versus pre-filled syringes), specific steps are required to insure that vaccines are administered and evaluated in a double-blind manner. By double-blinded, we mean that during the course of the study the vaccine recipient and those responsible for the evaluation of safety and reactogenicity study parameters, will be unaware which vaccine preparation was administered to a particular volunteer.

To do so, specific tasks of this study will be carried out by separate teams with specified functions. To insure blinding during the immunization phase and up to challenge, an individual may only serve on one team. Some personnel will be assigned to the Immunization Team, being directly in charge of the vaccine preparation as described in Section 6.4. To maintain blinding, a physician who is neither the Principal Investigator nor an Associate Investigator involved with post-immunization follow-up will administer all of the immunizations. The Principal investigator and/or the Associate Investigators who do not perform vaccine preparation or administration will subsequently evaluate for post-immunization side effects. Blinding will be maintained throughout the immunization and follow-up portions of the vaccine trial.

Before shipping the adjuvants to the site, GSK Biologicals will be responsible for insuring that individual adjuvants vials or syringes are packaged in boxes appropriately labeled and packaged for shipping from Rixensart, Belgium to WRAIR, Silver Spring, Maryland.

During the trial, a set of individual codes in code break envelopes for each study participant will be kept by WRAIR’s medical monitor and the Chief, Clinical Trial Monitoring Branch, USAMMDA. The code break envelopes will contain information associating a patient alphanumeric identification number to specific vaccine. If deemed necessary for reasons such as safety, WRAIR’s medical monitor will unblind a specific participant without revealing the study blind to the investigator. The code will be broken in the case of medical events that the investigator/physician in charge of the participant feels cannot be treated without knowing the identity of the study vaccine.

At the conclusion of the study, the PI and sub-investigators will agree upon a date and procedure for breaking the study blind and begin data analysis.

## Replacement of unusable vaccine doses

Additional doses of vaccine and adjuvant will be provided to replace those that are unusable (see Appendix E for details of supplies).

In case a vial is broken or unusable, the person in charge of the immunization should use a replacement vaccine. Although the sponsor need not be notified immediately in these cases, documentation of the use of the replacement vaccine and reason for using it must be recorded by the investigator on the vaccine administration page of the CRF and on the vaccine accountability form.

Proper disposal of syringes/needles will occur after each vaccine administration. Spent vaccine vials/ adjuvant vials and pre-filled syringes, labeled with the volunteers’ identification number, will be retained separately for confirmation of correct dosing at the end of each immunization session.

## Packaging

See Appendix E.

## Vaccine accountability

See Appendix E**.**

## Concomitant medication/treatment

At each study visit/contact, the investigator or designee should question the participant or their legal representative about any medication taken.

Any drugs with antimalarial properties (e.g. tetracyclines and sulfa drugs), immunosuppressants or other immune-modifying drugs or treatments, any vaccine other than the study vaccine(s) and any antipyretics administered at ANY time during the period starting 30 days prior to the first administration of study vaccine(s) and ending one month (minimum 30 days) after the last administration of study vaccine(s) must be recorded in the CRF with trade name and/or generic name of the medication, medical indication, total daily dose, route of administration, start and end dates of treatment.

Any other concomitant medication administered prophylactically in anticipation of reaction to the immunization must also be recorded in the appropriate CRF with trade name and/or generic name of the medication, total daily dose, route of administration, start and end dates of treatment and coded as ‘Prophylactic’.

# Health Economics

Not applicable.

# Adverse Events

The recording of adverse events is an important aspect of study documentation. It is the responsibility of the investigator or designee to document all adverse events according to the detailed guidelines set out below.

The participants will be instructed to contact the investigator immediately should they manifest any signs or symptoms they perceive as serious.

## Eliciting and documenting adverse events

*Adverse event definition*

An adverse event includes any noxious, pathological or unintended change in anatomical, physiological or metabolic functions as indicated by physical signs, symptoms and/or laboratory detected changes occurring in any phase of the clinical study whether associated with the study vaccine, active comparator or placebo and whether or not considered immunization related. This includes an exacerbation of pre-existing conditions or events, intercurrent illnesses, or vaccine or drug interaction. Anticipated day-to-day fluctuations of pre-existing conditions, including the disease under study, that do not represent a clinically significant exacerbation need not be considered adverse events. Discrete episodes of chronic conditions occurring during a study period should be reported as adverse events in order to assess changes in frequency or severity.

Adverse events should be documented in terms of a medical diagnosis(es). When this is not possible, the adverse event should be documented in terms of signs and symptoms observed by the investigator or reported by the participant at each study visit.

Pre-existing conditions or signs and/or symptoms (including any that are not recognized at study entry but are recognized during the study period) present in a participant prior to the start of the study should be recorded on the Medical History form within the participant's CRF.

Adverse events that occur after informed consent is obtained, but prior to immunization, will be documented the Medical History form within the participant's CRF as instructed by the Study Monitor.

Although not considered as an adverse event, hospitalization for either elective surgery related to a pre-existing condition that did not increase in severity or frequency following initiation of the study, or for routine clinical procedures (including hospitalization for “social” reasons) that are not the result of an adverse event, must be recorded in the CRF. If the hospitalization arises from a pre-existing condition, or was planned prior to the first immunization, it should be recorded in the Medical History form of the CRF. If the hospitalization arises from a pre-existing condition, and the hospitalization was planned after the first immunization, it should be recorded in the adverse event page of the CRF. In both cases, it should be recorded as ‘Hospitalization’, and the relationship to immunization will be checked “No”.

N.B. Adverse events to be recorded as endpoints are described in Section 8.1.1. All other adverse events will be recorded as unsolicited adverse events.

Events that are not alarming and may reasonably be regarded as caused by or probably caused by the vaccine should be batched together and reported to the Sponsor’s Representative at the conclusion of the Immunization Phase in the following manner:

Telephone (301-619-0317), facsimile (301-619-0197) or email (USAMRMCREGAFFAIRS@amedd.army.mil). Reporting to the Sponsor’s Representative does not fulfill the investigators’ duty to report all unanticipated problems involving risk to human participants or others to the IRB in accordance with 21 CFR 312.66. Follow instructions provided by the local IRB and HSRRB when reporting to these organizations.

*Surveillance period for occurrence of adverse events*

All adverse events occurring within one month (minimum 30 days) following administration of each vaccine must be recorded on the Adverse Event form in the participant's CRF, irrespective of severity or whether or not they are considered immunization-related.

Additionally, all serious adverse events (see Section 8.5) occurring during the period starting from the day of administration of the first study vaccine to each participant until study end for that participant must be recorded. See Section 8.5.2 for instructions for recording and reporting of serious adverse events.

Instances of death or congenital abnormality in offspring, if brought to the attention of the investigator AT ANY TIME after cessation of study AND suspected by the investigator to be related to study vaccine, should be reported to the USAMRMC Office of Research Protections and the appropriate contacts at GSK and MVI (see Section 8.5.2).

*Recording adverse events*

At each visit/assessment, all adverse events either observed by the investigator or one of his clinical collaborators or reported by the participant spontaneously or in response to a direct question will be evaluated by the investigator. Adverse events not previously documented in the study will be recorded in the Adverse Event form within the participant's CRF. The nature of each event, date and time (where appropriate) of onset, outcome, intensity, and relationship to immunization should be established. Details of any corrective treatment should be recorded on the appropriate page of the CRF. See Section 8.5.2 for instructions for reporting and recording of serious adverse events.

As a consistent method of soliciting adverse events, the participant or their legal representative should be asked a non-leading question such as:

"Have you felt different in any way since receiving the vaccine or since the previous visit?"

N.B. The investigator should record only those adverse events having occurred within the time frames defined above.

Adverse events already documented in the CRF, i.e. at a previous assessment, and designated as ‘ongoing’ should be reviewed at subsequent visits, as necessary. If these have resolved, the documentation in the CRF should be completed.

N.B. If an adverse event changes in frequency or intensity during a study period, the highest intensity will be recorded. If the adverse event recurs with an defined interval of symptom-free time (>24 hours) in between episodes, new record of the event will be started.

### Solicited adverse events

Table 4 lists the solicited local and general adverse events for the study.

Table 4: Solicited local and general adverse events

|  | **Adverse events** |
| --- | --- |
| **Local (injection site)** | Pain at the injection site |
|  | Swelling at the injection site |
|  | Redness at the injection site |
| **General** | Fever* (Oral Temperature) |
|  | Gastrointestinal (Nausea) |
|  | Headache |
|  | Malaise |
|  | Myalgia |
|  | Fatigue |
|  | Joint pain |

N.B. Temperature will be recorded in the evening. Should temperature measurement additionally be performed at another time of day, the highest temperature will be recorded. * Fever is defined as oral temperature ≥ 37.5°C or 99.5°F.

### Unsolicited adverse events

Unsolicited symptoms are adverse events reported by the participants that are different from those solicited, or unsolicited symptoms that begin after the seven day follow-up period for solicited symptoms. Space on the diary cards/source documents and CRF will be allocated for the recording of unsolicited symptoms. Should any systemic (general) signs/symptoms be reported, their relationship with the study vaccine will be assessed by the Investigator and transcribed into the CRF, as described in section 8.3

## Assessment of intensity

Intensity of the local adverse events should be assessed as described in Table 5.

Table 5: Intensity grading of local solicited adverse events.

| **Adverse Event** | **Intensity grade** | **Intensity definition** |
| --- | --- | --- |
| Pain at injection site | 0 | None |
|  | 1 | Pain that is easily tolerated |
|  | 2 | Pain that interferes with daily activity |
|  | 3 | Pain that prevents daily activity |
| Swelling at injection site | 0 | 0 mm |
| Record size | 1 | >0 - ≤ 20 mm |
|  | 2 | >20 - ≤ 50 mm |
|  | 3 | >50 mm |
| Erythema at injection site | 0 | 0 mm |
| Record size | 1 | >0 - ≤ 20 mm |
|  | 2 | >20 - ≤ 50 mm |
|  | 3 | >50 mm |

Intensity of the general adverse events should be assessed as described in Table 6:

Table 6: Intensity grading of general solicited adverse events

| **Adverse event** | **Intensity grade** | **Intensity** |
| --- | --- | --- |
| Fever  Record oral temperature | 0  1  2  3 | <37.5ºC  37.5 - ≤38.0ºC  >38.0 - ≤39ºC  >39ºC |
| Fatigue | 0 | Normal |
|  | 1 | Fatigue which is easily tolerated |
|  | 2 | Fatigue that interferes with normal activity |
|  | 3 | Fatigue that prevents normal activity |
| Nausea | 0 | None |
|  | 1 | Nausea that is easily tolerated |
|  | 2 | Nausea that interferes with daily activity |
|  | 3 | Nausea that prevents daily activity |
| Headache | 0 | None |
|  | 1 | Headache that is easily tolerated |
|  | 2 | Headache that interferes with daily activity |
|  | 3 | Headache that prevents daily activity |
| Malaise | 0 | None |
|  | 1 | Malaise that is easily tolerated |
|  | 2 | Malaise that interferes with daily activity |
|  | 3 | Malaise that prevents daily activity |
| Myalgia | 0 | None |
|  | 1 | Myalgia that is easily tolerated |
|  | 2 | Myalgia that interferes with daily activity |
|  | 3 | Myalgia that prevents daily activity |
| Joint pain (> 1 joint) | 0 | None |
|  | 1 | Joint pain that is easily tolerated |
|  | 2 | Joint pain that interferes with daily activity |
|  | 3 | Joint pain that prevents daily activity |

For all other adverse events, maximum intensity should be assigned to one of the following categories:

| 0 | = | No adverse event |
| --- | --- | --- |
| 1 | = | An adverse event that is easily tolerated by the participant, causing minimal discomfort and not interfering with everyday activities. |
| 2 | = | An adverse event that is sufficiently discomforting to interfere with normal everyday activities. |
| 3 | = | An adverse event that prevents normal, everyday activities, i.e. prevents attendance at work and necessitates the administration of corrective therapy. |

***Serum Biochemistry grading:***

| **Adverse event** | **Intensity grade** | **Intensity** |
| --- | --- | --- |
| BUN | Normal range | 7-25 mg/dL |
|  | 1 | ≥26 and < 30 mg/dL |
|  | 2 | ≥30 and < 35 mg/dL |
|  | 3 | ≥ 35 mg/dL |
| Creatinine (Males) | Normal range | 0.5-1.6 mg/dL |
|  | 1 | ≥1.7 and < 1.8 mg/dL |
|  | 2 | ≥ 1.8 and < 2.0 mg/dL |
|  | 3 | ≥ 2.0 mg/dL |
|  |  |  |
| Creatinine (Females) | Normal range | 0.5-1.3 mg/dL |
|  | 1 | ≥1.4 and < 1.6 mg/dL |
|  | 2 | ≥1.6 and < 1.8 mg/dL |
|  | 3 | ≥1.8 mg/dL |
|  |  |  |
| Liver Function Tests (AST/ALT) |  |  |
| AST - Males | Normal range | 3-50 |
| AST - Females | Normal range | 3-35 |
| ALT - Male | Normal range | 3-60 |
| ALT - Female | Normal range | 3-40 |
|  | 1 | > 1.0 and < 2.5 times the upper limit of normal |
|  | 2 | ≥2.5 times and < 4 times the upper limit of normal |
|  | 3 | ≥4 times the upper limit of normal |

Hematology grading

| **Adverse event** | **Intensity grade** | **Intensity** |
| --- | --- | --- |
| Hemoglobin (Males) | Normal range | 13.2-17.1 gm/dL |
|  | 1 | < 13.2 and ≥11.5 gm/dL |
|  | 2 | < 11.5 and ≥10.5 gm/dL |
|  | 3 | < 10.5 gm/dL |
| Hemoglobin (Females) | Normal range | 11.7-15.5 gm/dl |
|  | 1 | < 11.7 and ≥11 gm/dL |
|  | 2 | < 11 and ≥10L gm/dL |
|  | 3 | < 10 gm/dL |
| Increase in Leukocytes | 1 | ≥11,000 and < 15,000 |
| (WBC) | 2 | ≥15,000 and < 20,000 |
|  | 3 | ≥20,000 |
| Decrease in Leukocytes | 1 | < 3,500 and ≥2,500 |
| (WBC) | 2 | < 2,500 and ≥1,500 |
|  | 3 | < 1,500 |
| Decrease in Platelets | 1 | < 140,000 and ≥125,000 |
|  | 2 | < 125,000 and ≥100,000 |
|  | 3 | < 100,000 |

## Assessment of causality

Every effort should be made by the investigator to explain each adverse event and assess its causal relationship, if any, to administration of the study vaccine(s).

The degree of certainty with which an adverse event can be attributed to administration of the study vaccine(s) (or alternative causes, e.g. natural history of the underlying diseases, concomitant therapy, etc.) will be determined by how well the event can be understood in terms of one or more of the following:

- Reaction of similar nature having previously been observed with this type of vaccine and/or formulation.
- The event having often been reported in literature for similar types of vaccines.
- The event being temporally associated with immunization or reproduced on re-immunization.

All solicited local (injection site) reactions will be considered causally related to immunization. For all participants undergoing a malaria challenge, the period of recording unsolicited adverse events post-immunization will be as per protocol but there will be no assessment of causality for an adverse event following malaria challenge, except if the adverse event meets the criteria to be determined “serious” (see Section 8.5.1 for definition of serious adverse event).

Causality of all other adverse events should be assessed by the investigator using the following method:

In your opinion, did the vaccine(s) possibly contribute to the adverse event?

| NO | : | The adverse event is not causally related to administration of the study vaccine(s). There are other, more likely causes and administration of the study vaccine(s) is not suspected to have contributed to the adverse event. |
| --- | --- | --- |
| YES | : | There is a reasonable possibility that the vaccine contributed to the adverse event. |

Non-serious and serious adverse events will be evaluated as two distinct events given their different medical nature. If an event meets the criteria to be determined “serious” (see Section 8.5.1 for definition of serious adverse event), it will be examined by the investigator to the extent to be able to determine ALL contributing factors applicable to each serious adverse event.

Other possible contributing factors include:

- Medical history
- Other medication
- Protocol required procedure (e.g. challenge)
- Lack of efficacy of the vaccine
- Erroneous administration
- Other cause (specify)

## Following-up of adverse events and assessment of outcome

Investigators should follow up subjects with adverse events until they have returned to normal, stabilized, or the event is otherwise explained or the volunteer is lost to follow up. Clinically significant laboratory abnormalities, as well as any adverse event, will be followed up until they have returned to normal, or a satisfactory explanation has been provided. Reports relative to the subsequent course of an adverse event noted for any participant must be submitted to the PI, study monitor (USAMMDA), all IRBs, GSK and MVI.

Outcome should be assessed as:

1 = Recovered

2 = Recovered with sequelae

3 = Ongoing at study conclusion (active phase)

4 = Died

5 = Unknown

## Serious adverse events

### Definition of a serious adverse event

A serious adverse event is any untoward medical occurrence that results in death, is life threatening*, results in persistent or significant disability/incapacity†, requires in-patient hospitalization‡ or prolongation of existing hospitalization or is a congenital anomaly/birth defect in the offspring of a study subject. In addition, important medical events that may jeopardize the patient or may require intervention to prevent one of the other outcomes listed above should be considered serious. (Examples of such treatments are intensive treatment in an emergency room or at home for allergic bronchospasm; blood dyscrasias or convulsions that do not results in hospitalization; or development of drug dependency or drug abuse.)

Although not considered as ‘serious adverse events’, cancers should be reported in the same way as serious adverse events.

*Life threatening—definition: An adverse event is life threatening if the subject was at risk of death at the time of the event; it does not refer to an event that hypothetically might have caused death if it were more severe.

^†^Disabling/incapacitating—definition: An adverse event is incapacitating or disabling if the event results in a substantial disruption of the subject's ability to carry out normal life functions. This definition is not intended to include experiences of relatively minor medical significance.

‡Hospitalization: In general, hospitalization signifies that the subject has been detained (usually involving at least an overnight stay) at the hospital or emergency ward for treatment that would not have been appropriate in the physician’s office or out-patient setting.

Hospitalization for either elective surgery related to a pre-existing condition that did not increase in severity or frequency following initiation of the study or for routine clinical procedures¶ (including hospitalization for “social” reasons) that are not the result of an adverse event need not be considered as adverse events and are therefore not serious adverse events.

¶Routine Clinical Procedure—definition: One which is defined as a procedure that may take place during the study period and should not interfere with the study vaccine administration or any of the ongoing protocol specific procedures.

N.B. If anything untoward is reported during an elective procedure, that occurrence must be reported as an adverse event, either ‘serious’ or ‘non-serious’ according to the usual criteria.

When in doubt as to whether ‘hospitalization’ occurred or was necessary, the adverse event should be considered serious.

### Reporting serious adverse events

Serious Adverse Events will be reported as they occur during the period starting from the day of administration of the first study vaccine to each participant and ending with the last study visit or performance of the last study procedure. Any serious adverse events occurring during this period, whether or not considered to be related to the study vaccine, must be reported by the investigator by fax or email within 24 hours (1 calendar day) of his/her becoming aware of the event to the GlaxoSmithKline Study Contact for Serious Adverse Event Reporting, the MVI Medical Officer, the USAMRMC Office of Research Protections (301-619-2165; non-duty hours call 301-619-2165 and send information by facsimile to 301-619-7803) and to the USAMMDA Regulatory Affairs Office (telephone 301-619-0317; facsimile 301-619-0197 or via email USAMRMCREGAFFAIRS@amedd.army.mil).  In addition the IRB of record, HURC, should be notified by phone (301-319-9940) or by facsimile (310-319-9961), Office of Research Management, Walter Reed Army Institute of Research, 503 Robert Grant Avenue, Silver Spring, Maryland, 20910-7500. A written report will follow the initial contacts within 3 working days. The written report will be addressed to the US Army Medical Research and Materiel Command, ATTN: MRMC-ORP, 504 Scott Street, Fort Detrick, Maryland 21702-5012.

Unanticipated problems involving risk to volunteers or others, serious adverse events related to participation in the study and all volunteer deaths should be promptly reported by phone (301-319-9940) or by facsimile (310-319-9961) to the WRAIR HURC, and by phone (301-619-2165), by facsimile (301-619-7803) or by email (hsrrb@amedd.army.mil) to the HSRRB. A complete written report should follow the initial notification. In addition to the methods above, follow-up reports will be sent to the Director, Office of Research Management, Walter Reed Army Institute of Research, 503 Robert Grant Avenue, Silver Spring, Maryland, 20910-7500 as well as U.S. Army Medical Research and Materiel Command, ATTN: MCMR-ORP, 504 Scott Street, Fort Detrick, Maryland 21702-5012.

The investigator will document all available information regarding the serious adverse event in the Serious Adverse Event pages contained in the individual case report form and FAX or email to the GlaxoSmithKline Contact for Serious Adverse Event Reporting, the MVI Medical Officer, and the USAMRMC Office of Research Protections. The investigator should not wait to receive additional information to fully document the event before notifying MVI, GlaxoSmithKline Biologicals or the USAMRMC Office of Research Protections (ORP) and the USAMMDA Regulatory Affairs Office of a serious adverse event.

This initial notification should give, as a minimum, sufficient information to permit identification of:

- The reporter
- The participant
- The antigen and adjuvant lot
- Adverse events
- Date of last immunization

• Date of onset

In the event of a death or a serious adverse event determined by the investigator to be related to immunization, receipt of the report must be confirmed by a telephone call. The fax or email report should be followed by a full written report using either the GSK Biologicals Serious Adverse Events form or the US FDA MedWatch form, detailing relevant aspects of the adverse events in question. It should include copies of relevant hospital case records, autopsy reports, and other documents where applicable.

In the event that any serious adverse events determined by the investigator to be related to immunization are detected following any immunization in any of the vaccine groups, no further immunizations will be administered until a written report has been submitted to the USAMRMC Office of the Research Protections, the USAMMDA Regulatory Affairs Office, and the FDA, and the investigators have conferred with the MVI, GSK and WRAIR Medical Monitor. All involved IRBs will also be notified.

Instances of death or congenital abnormality in offspring, if brought to the attention of the investigator AT ANY TIME after cessation of study vaccine AND suspected by the investigator to be related to study vaccine, should be reported to the USAMRMC Office of Research Protections, USAMMDA Regulatory Affairs Office, and the appropriate contacts at GSK and MVI (see Section 8.5.2).

All information should be sent promptly to the contacts listed below:

| **Study Contacts for Reporting Serious Adverse Events** | |
| --- | --- |
| **USAMRMC Deputy for Office of Research Protections**  US Army Medical Research and Materiel Command  ATTN: MCMR-ORP-HR  504 Scott Street  Fort Detrick, MD 21702-9240  Tel: 301-619-2165  Fax: 301-619-7803 | **USAMMDA**  Regulatory Affairs Office  USAMMDA  ATTN: MCMR-UMR  1430 Veterans Drive  Ft. Detrick, MD 21702-5011  Tel: 301-619- 0317  Fax: 301-619-0197 |
| **PATH Malaria Vaccine Initiative**  Eveline Tierney, DO, MPH  7500 Old Georgetown Road, 12^th^ Floor  Bethesda, MD 20814  Tel: 240-395-2715  Fax: 240-395-2591  Email: etierney@malariavaccine.org | |
| **Independent Medical Monitor**  Peter J. Weina, LTC, MC  Department of Experimental Therapeutics, WRAIR  503 Robert Grant Avenue  Silver Spring, MD 20910  Tel: 301-319-9956  Fax: 301-319-7360  Email: peter.weina@us.army.mil | |
| **Study Contact at GlaxoSmithKline for Reporting Serious Adverse Events** | |
| **Primary Study contacts at GSK Biologicals** | |
| Central Study Coordinator  Arlette Simonon  GlaxoSmithKline Biologicals  Central Study Coordinator Rue de l'Institut, 89  1330 Rixensart  Belgium  email arlette.simonon@gskbio.com  Tel. office: 32 2 656 5507  Fax: 32-2-656 8044 | GSK Study Monitor  Ann Miller  GSK, Senior CRA  P.O. Box 65  Matthews, VA 23109    email: ann.c.miller@gsk.com  Tel. office: 1 804 725 05 27  Fax: 1 804 725 04 27 |
| **Back-up Study Contacts at GlaxoSmithKline for Reporting Serious Adverse Events** | |
| GSK Biologicals Clinical Safety Physician,  GlaxoSmithKline Biologicals,  Rue de l’Institut 89, 1330 Rixensart,  Belgium.  Tel: +32.2.656.87.98  Fax: +32.2.656.80.09  email: rix.ct-safety-vac-@gskbio.com  Mobile phone for 7/7 day availability: +32.477.40.47.13 | |

## Pregnancy

Participants who become pregnant during the study period must not receive additional injections of study vaccine and must not undergo challenge, but may continue other study procedures to include blood draws for immunogenicity and safety and review of symptoms/physical exams for reactogenicity data, at the discretion of the primary investigator.

Participants should be instructed to notify the investigator if it is determined after completion of the study that they became pregnant either during the study or within 60 days of the third immunization or malaria challenge. Although not considered an adverse event, pregnancy should be reported on the pregnancy report form and in the same way as a serious adverse event (see section 8.5.2), i.e., documenting all available information regarding the serious adverse event in the Serious Adverse Event pages and sending by FAX or email to the GlaxoSmithKline Contact for Serious Adverse Event Reporting, the MVI Medical Officer, and the USAMRMC Office of Research Protections within 24 hours (1 calendar day) of the investigator becoming aware of the event. A written report will follow within three days addressed to the contacts at GSK, MVI and USAMMDA.

A pregnancy should be followed to term, any premature terminations reported, and the health status of the mother and child including date of delivery and the child’s gender and weight should be reported to GlaxoSmithKline Biologicals, the MVI Medical Officer, and the USAMRMC Office of Research Protections after delivery.

## Treatment of adverse events

Treatment of any adverse event is at the sole discretion of the investigator and or WRAIR medical monitor and according to current Good Medical Practice. The applied measures should be recorded in the CRF of the vaccinee. If a Study volunteer requires treatment of an adverse reaction(s) arising from study research procedures administered in accordance with the Protocol, WRAIR shall provide for reasonable and necessary medical treatment at an Army hospital or clinic free of charge. This will not apply when the expenses are attributable to the negligence or willful misconduct of any person in the employ of WRAIR. No other compensation of any type will be provided by the WRAIR or MVI or GSK to the Research Subject. If full coverage for a research related adverse event has already been obtained from insurance, government programs, or other third parties, the partners shall coordinate payments under the terms of the research subject’s benefits directly with the benefit provider.

# Participant Completion and Withdrawal

## Participant completion

A participant is considered to have completed the study if he/she returns for the concluding visit or for the concluding contact foreseen in the protocol.

## Participant withdrawal

Participants who are withdrawn for AEs must be clearly distinguished from participants who are withdrawn for other reasons. Investigators will follow participants who are withdrawn as result of a SAE/AE until resolution of the event. Withdrawals will not be replaced.

### Participant withdrawal from the study

From an analysis perspective, a ‘withdrawal’ from the study is any participant who did not come back for the concluding visit.

A participant qualifies as a ‘withdrawal’ from the study when no study procedure has occurred, no follow-up has been performed and no further information has been collected for this participant from the date of withdrawal/last contact.

Investigators will make an attempt to contact those participants who do not return for scheduled visits or follow-up.

Information relative to the withdrawal will be documented on the Study Conclusion page of the CRF. The investigator will document whether the decision to withdraw from the study was made by the participant or the investigator and which of the following possible reasons was responsible for withdrawal:

- serious adverse event
- non-serious adverse event
- protocol violation (specify)
- consent withdrawal, not due to an adverse event
- moved from the study area
- lost to follow-up
- other (specify)

### Participant withdrawal from investigational product

A ‘withdrawal’ from the investigational product is any participant who does not receive the complete treatment, i.e. when no further planned immunization occurs from the date of withdrawal. A participant withdrawn from the investigational product may not necessarily be withdrawn from the study as further study procedures or follow-up may be performed (safety or immunogenicity) if planned in the study protocol.

Information relative to premature discontinuation of the investigational product will be documented on the Vaccine Administration page of the CRF. The investigator will document whether the decision to discontinue further immunization was made by the participant or the investigator and which of the following possible reasons was responsible for withdrawal:

- serious adverse event,
- non-serious adverse event,
- other (specify).
- A participant may also be withdrawn from the study as per described stopping criteria outlined in Section 5.2.3.

# Data Evaluation: criteria for evaluation of objectives

While FMP2.1/AS02A has completed a Phase 1 study at WRAIR, FMP2.1/AS01B as a formulation has not yet been tested in humans. This study design allows for safety and reactogenicity evaluation of 1/5^th^ dose (10 µg) FMP2.1/AS01B (Cohort 1) prior to proceeding on to comparison of safety and reactogenicity of full dose (50 µg) FMP2.1/AS01B and FMP2.1/AS02A (Cohort 2) with subsequent determination of efficacy of both formulations. The 1/5^th^ dose of FMP2.1/AS01B will tested according to the same study procedures as the full dose formulations i.e. 3 immunizations at 0-, 1-, 2-months with evaluation of vaccine safety and reactogenicity as well as collection of immunogenicity data. Since the 1/5^th^ dose of FMP2.1/AS01B will not be a candidate for advancement in malaria vaccine selection, volunteers receiving this formulation will not undergo sporozoite challenge for efficacy, and thus the study objectives and endpoints concerning efficacy do not apply to this cohort, only to Cohort 2. However, since this is a first-in-man study of this formulation, the collection of immunogenicity data such as humoral and cell-mediated analyses will be done during the Immunization Phase as it will provide valuable information as to the immune response to the formulation FMP2.1/AS01B.

## Study Objectives

***Primary objective:***

- To assess the safety and reactogenicity of candidate malaria vaccines FMP2.1/AS01B and FMP2.1/AS02A when administered intramuscularly as three immunizations on a 0-, 1- and 2-month schedule to malaria-naïve adult volunteers living in the United States.

***Secondary objectives:***

- To assess the humoral immune responses by ELISA to candidate malaria vaccines FMP2.1/AS01B and FMP2.1/AS02A.
- To assess functionality of anti-FMP2.1 antibodies induced by FMP2.1/AS01B and FMP2.1/AS02A to inhibit the growth of asexual parasites as measured by standardized GIA.
- To assess the efficacy of FMP2.1/AS01B and FMP2.1/AS02A against sporozoite challenge with *P. falciparum*

***Tertiary objectives:***

- To assess the cell-mediated immune responses to FMP2.1/AS01B and FMP2.1/AS02A malaria vaccine when administered intramuscularly as three immunizations on a 0-, 1- and 2-month schedule.

## Co-Primary endpoints

- Occurrence of solicited symptoms over a seven day follow-up period (day of immunization and six subsequent days) after each immunization
- Occurrence of unsolicited symptoms over a 30 day follow-up period (day of immunization and 29 subsequent days) after each immunization
- Occurrence of serious adverse events during the study period.

## Secondary endpoints

- Anti-AMA-1 antibody titers at specified time points during Immunization Phase for Cohorts 1 & 2 (Days 0, 14, 28, 42, 56, 70),and Challenge Phase for Cohort 2 (Day of Challenge (Month 2), Day 7 post-challenge, approximately Day 30 post-challenge (Week I or II), Day 90 post-challenge (Month 5))
- Functionality of anti-AMA-1 antibodies as percent parasite growth inhibition as measured by standardized homologous and heterologous GIA at specified time points during the Immunization Phase for Cohorts 1 & 2 (Days 0, 14, 28, 42, 56, 70),and Challenge Phase for Cohort 2 (Day of Challenge (Month 2), Day 7 post-challenge, approximately Day 30 post-challenge (Week I or II), Day 90 post-challenge (Month 5))
- Development of parasitemia and time to parasitemia after primary challenge following administration of the FMP2.1/AS01B and FMP2.1/AS02A candidate vaccines (50 µg/0.5 mL dose formulations only)

## Tertiary endpoints

- Cell-mediated immune responses at time points at which blood samples are taken:
- IFNγ determination by the ELISPOT assay on Day 0, 70 and 90 days post-challenge
- Intracellular Cytokine staining (ICS) characterizing CD4/CD8 T cells expressing IFNγ and/or TNFα and/or IL-2 on Day 0, 70 and 90 days post-challenge

## Study cohorts/data sets to be evaluated

**Total cohort**

The Total cohort will include all enrolled (i.e., randomized or immunized) participants for whom data are available. For the Total analysis of safety, this will include all immunized participants for whom safety data are available. For the Total analysis of efficacy, this will include immunized participants for whom data concerning efficacy endpoint measures are available.

**Protocol defined or According To Protocol (ATP) cohort for analysis of safety**

The ATP cohort for analysis of safety/reactogenicity will include all participants who meet all eligibility criteria according to their assignment; complying with all procedures defined in the protocol and will include participants for whom data are available for the analysis of safety/reactogenicity.

**Protocol defined or According To Protocol (ATP) cohort for analysis of efficacy**

The ATP cohort for analysis of efficacy will include all participants for whom differential treatment effect on efficacy is likely (i.e., those meeting all eligibility criteria, complying with the procedures defined in the protocol) and for whom data concerning efficacy endpoint measures are available.

**Protocol defined or ATP cohort for analysis of immunogenicity**

The ATP cohort for analysis of immunogenicity will include those participants meeting all eligibility criteria, complying with the procedures defined in the protocol, and will include participants for whom assay results are available for antibodies against at least one study vaccine antigen component after immunization.

## Estimated sample size for challenge model

The group sizes are designed to mirror previous malaria vaccine challenge studies performed using the WRAIR challenge model. The logistics of the challenge model (e.g., physical space for actual mosquito challenge, infection of mosquitoes, and volunteer assessment and follow-up by study physicians) limits the total number of volunteers challenged with malaria. Any malaria vaccine that appears to be efficacious in the context of the WRAIR challenge model will be targeted for transition to larger-scale field studies, where group sizes will be selected on the basis of statistical considerations. Since a major objective remains the assessment of safety and immunogenicity, the numbers chosen should provide the preliminary comparison that is needed.

### Sample Size Justification

This study is designed to assess safety, immunogenicity, and efficacy of two candidate vaccines, and not for the support of intergroup comparisons. Due to importance of conducting a safe challenge study, practical limits on the sample size within each cohort are in place; therefore, comparative statistics will be performed but will have low power to detect other than large differences between each cohort.

### Reactogenicity

The study will collect data on specific reactogenicity events. Data will be tabulated and reported. The study is not intended to determine other than very large differences in rates of adverse events.

**10.6.2.1 Protection**

This study will provide preliminary data on the efficacy (development of parasitemia and time to development of parasitemia) of both FMP2.1/AS01B and FMP2.1/AS02A vaccine formulations against sporozoite challenge. The study is powered to detect a prolongation in the prepatent period of approximately two days in immunized volunteers as compared with controls. This estimate is based upon previous challenge studies in which the pre-patent period standard deviation for a control group of six volunteers is 1.5 days. Six control group volunteers will enable us to detect a two-day mean delay in prepatent parasitemia with 80% power. A two day delay in prepatent period represents either a log reduction in the number of hepatic merozoites or a substantial reduction in erythrocytic schizogony or both.

In conclusion, as outlined previously, the demonstration of a statistically significant prolongation in the prepatent period would suggest a physiologically important antimalarial effect, and could accelerate the development of RTS,S in combination with the FMP2.1 antigen.

## Final analyses

**Interim Analysis**

There will be an interim analysis planned to provide a preliminary evaluation of vaccine efficacy. This interim report will be written after completion of the four weekly visits post-malaria challenge.

**Final report**Will include data on safety/reactogenicity, available humoral and cell-mediated immunogenicity results and vaccine efficacy.

**Annex report**Will exploratory analyses data as well as any analyses not reported at the time of the Final Report.

### Analysis of demographics

Demographic characteristics (age, sex, and race) of each study cohort will be tabulated.

The mean age (plus range and standard deviation) by sex of the enrolled participants, as a whole, and per group, will be calculated.

### Analysis of safety

***Analysis of reactogenicity***

Reactogenicity analyses will be performed primarily on an intent-to-treat basis.

- The overall percentage of participants with at least one local adverse event (solicited and unsolicited), with at least one general adverse event (solicited and unsolicited), during the seven-day follow-up period after immunization will be tabulated for each cohort and each group.
- The incidence, intensity and relationship of individual solicited symptoms over the seven day follow‑up period will be calculated per cohort and per group.
- The incidence, intensity, and relationship of individual solicited symptoms over the first four days of follow‑up (days 0-3) after immunization will also be calculated per cohort and per group.
- The number of participants with at least one report of unsolicited adverse event, classified by the Medical Dictionary for Regulatory Activities (MedDRA), and reported up to 30 days after immunization will be tabulated per cohort and per group. The intensity and relationship to immunization of the unsolicited symptoms reported will also be assessed.
- Serious adverse events are expected to be rare, but where observed will be described.

***Clinical Laboratory parameters***

Hematological (CBC) and biochemical (ALT, AST, BUN and creatinine) laboratory parameters will be measured at specific time points during the study. Clinically relevant abnormal values will be tabulated and a trend analysis performed if deemed necessary.

### Analysis of immunogenicity

Immunogenicity results will be reported for Cohort 1 as a stand-alone endpoint of interest and not be compared to immunogenicity results from Cohort 2.

***Anti-AMA-1 antibody responses***

ELISA titer data is log-transformed to normalize the distribution so that parametric analyses can be performed. For each group, anti-AMA-1 antibody titers with 95% confidence intervals will be determined by ELISA and tabulated at each time point at which blood samples are taken for serology. In the MSL at WRAIR, titer is defined as the serum dilution required to yield an optical density (OD) of 1.0. Results may be reported in either raw titer data or in µg/mL as compared to a quantitative standard currently being developed (if available at time of analysis).

*Growth Inhibition assay response*

Growth inhibition assays (GIA) will be reported in a tabular manner as percent growth inhibition seen at a given serum dilution compared with a negative control serum dilution such as preimmune serum form the same volunteer. GIA will be performed in all volunteers following standard procedures. A serum will be considered to be GIA positive if, compared with negative control serum, the growth inhibition value is 10% or more after a two-tailed student’s *t* test with a p value less than 0.05.

***Cell-mediated immune response (CMI) analysis***

Main CMI analysis to be performed will be IFNγ determinations by the ELISPOT assay as well as intracellular cytokine staining assays. Only descriptive analysis will be undertaken for the CMI endpoints.

For cytokine producing cell determinations, the results will be presented as the arithmetic mean (AM) of three wells with FMP2.1 antigen (Ag) – AM of three wells with medium alone. The data will be scored as number of ELISPOTS/10^6^ cells. Statistical comparison will be done based on AM of pre and post-immunization samples and challenge with associated 95% confidence intervals.

### Analysis of efficacy

*ATP Cohort Efficacy*

Efficacy will be assessed by comparison of malaria incidence and time to onset of parasitemia after primary challenge as compared to infectivity controls. Immunized volunteers who develop parasitemia after the infectivity control group as measured by delay in patency of infection >2 days versus mean prepatent period in unimmunized infectivity controls will be classified as “delayed prepatent period”. For the purpose of this primary efficacy analysis, those volunteers who remain smear negative through day 30 post challenge will be classified as protected. Although this study is a small, descriptive Phase I/IIa trial and differences may not reach statistical significance, where statistical analyses are appropriate, the following will be used:

- Fisher’s Exact test will be used for the comparison of malaria incidence between the two immunized groups.
- Kaplan-Meier analysis will be performed on time to onset of parasitemia, with testing between the two treatment groups using the log-rank statistic.
- Relative risk of infection and 95% confidence intervals will be calculated.

All statistical tests will be two-tailed.

### Exploratory analyses

The following exploratory tests will be performed as feasible and available results described.

- Microarray testing (gene chip analysis) will be used for the identification of possible patterns of gene expression associated with protection.
- Assess level of peripheral parasitemia after sporozoite challenge as measured by quantitative PCR
- CMI: The aim of these exploratory analyses is mainly to identify potential correlates and mechanisms of protective efficacy.
- Determinations of level of multiple cytokines and chemokines such as TNFα and IFNγ, IL-1b, IL-2, IL-4, IL-7, IL-13, IL-21, MIP, RANTES and others by particle-based assay (*Luminex*) at specified time points.
- Whole blood assay to analyze number of Ag-specific CD4 or CD8 T cells expressing IFNγ respectively per million of CD4 T or CD8 T cells.
- ELISA for titers to AMA-1 FVO strain, FMP2.1 subgroup analysis, immunoglobulin isotypes.
- GIA assays developed by WRAIR and NIH.

## Final analysis

**Final analysis**A final report will be created encompassing all safety, reactogenicity and efficacy data collected during the entire trial period. The results will be determined using cleaned data, reported by group and will not identify individuals. The personnel responsible for creating such report will be the primary investigator of the vaccine trial in conjunction with MVI.

# Administrative Matters

To comply with Good Clinical Practice important administrative obligations relating to investigator responsibilities, monitoring, archiving data, audits, confidentiality and publications must be fulfilled. See Appendix B for details.

# References

1 Nchinda TC, Malaria: A reemerging disease. *Emerging Infectious Dis*. 1998; 4(3): 398-403.

2 Sturchler D, How much malaria is there worldwide? *Parasitol Today*. 1989; 5:39.

3 Mahanty S, Saul A, Miller LH, Progress in the development of recombinant and synthetic blood-stage malaria vaccines. *J. Exp Med.* 2003; 206: 3781-3788.

4 Healer J, Crawford S, Ralph S, McFadden G, Cowman A, Independent translocation of two micronemal proteins in developing *Plasmodium falciparum* merozoites. *Infect. Immun*. 2002; 70: 5751-5758.

5 Silvie O, Franetich JF, Charrin S, Mueller MS, et al., A role for apical membrane antigen 1 during invasion of hepatocytes by *Plasmodium falciparum* sporozoites. *J Biol Chem*. 2004 ; 279(10):9490-9496.

6 Hodder AN, Crewther PE, Matthew SM, Reid GE, et al., The disulfide bond structure of *Plasmodium* apical membrane antigen-1. *J. Biol. Chem*. 1996; 271: 29446-29452.

7 Howell SA, Withers-Martinez C, Kocken CHM, Thomas AW, Blackman MJ, Proteolytic processing and primary structure of *Plasmodium falciparum* apical membrane antigen-1. *J. Biol. Chem*. 2001; 276(33): 31311-31320.

8 Mitchell GH, Thomas AW, Margos G, Dluzewski AR, Banister LH, Apical membrane antigen 1, a major malaria vaccine candidate, mediates close attachment to invasive merozoites to host red blood cells. *Infect.* *Immun.* 2004; 72:154-158

9 Dutta S, Haynes JD, Moch JK, Barbosa A, Lanar DE, Invasion-inhibitory antibodies inhibit proteolytic processing of apical membrane antigen 1 of *Plasmodium falciparum* merozoites.  *PNAS* 2003 ; 100(21): 12295-12300.

10 Thomas AW, Deans JA, Mitchell GH, Alderson T, Cohen S, The Fab fragments of monoclonal IgG to a merozoite surface antigen inhibit *Plasmodium knowlesi* invasion of erythrocytes. *Molec Biochem Parasit*. 1984 ;13 :187-199.

11 Kato, K, Ghislaine Mayer DC, Singh S, Reid M, Miller LH, Domain III of *Plasmodium falciparum* apical membrane antigen 1 binds to the erythrocyte membrane protein Kx. *PNAS* 2005; 102(15): 5552-5557.

12 Hodder AN, Crewther PE, Anders RF, Specificity of the protective antibody response to apical membrane antigen 1. *Infect Immun*. 2001; 69:3286-3294.

13 Kennedy MC, Wang J, Zhang Y, Miles AP et al., *In vitro* studies with recombinant *Plasmodium falciparum* apical membrane antigen 1 (AMA1) : Production and activity of an AMA1 vaccine and generation of a multiallelic response. *Infect Immun*. 2002; 70: 6948-6960.

14 Anders RF, Crewther PE, Edwars S, et al., Immunisation with recombinant AMA-1 protects mice against infection with *Plasmodium chabaudi*. *Vaccine* 1998;16(2-3):240-247.

15 Narum DL, Ogun SA, Thomas AW and Holder AA, Immunization with parasite-derived apical membrane antigen 1 or passive immunization with a specific monoclonal antibody protects BALB/c mice against lethal *Plasmodium yoelii yoelii* YM blood stage infection. *Infect Immun*. 2000; 68: 2899-2906.

16 Xu H, Hodder AN, Yan H, Crewther PE et al., CD4+ T cells acting independently of antibody contribute protective immunity to Plasmodium chabaudi infection after apical membrane antigen 1 immunization. *J Immuol.* 2000; 165: 389-396.

17 Dutta S, Lalitha PV, Ware LA, Barbosa A et al., Purification, characterization, and immunogenicity of the refolded ectodomain of the *Plasmodium falciparum* apical membrane antigen 1 expressed in Escherichia coli. *Infect. Imm*.2001; 70: 3101-3110.

18 Deans JA, Knight AM, Jean WC et al., Vaccination trials in rhesus monkeys with a minor, invariant, *Plasmodium knowlesi* 66 kD merozoite antigen. *Parasit Immunol*. 1988;10: 535-552.

19 Stowers AW, Kennedy MC, Keegan BP, Saul A, Long CA, Miller LH, Vaccination of monkeys with recombinant *Plasmodium falciparum* apical membrane antigen 1 confers protection against blood-stage malaria. *Infect* *Immun*. 2002 ; 70(12) : 6961-6967.

20 Polley SD, Mwangi T, Kocken CHM, Thomas AW, et al., Human antibodies to recombinant protein constructs of *Plasmodium falciparum* apical membrane antigen 1 (AMA1) and their associations with protection from malaria. *Vaccine*. 2004 ; 23 : 718-728

21 Lal AA, Hughes MA, Oliveira DA, Nelson C et al., Identification of T-cell determinants in natural immune responses to the *Plasmodium falciparum* apical membrane antigen 1 (AMA-1) in an adult population exposed to malaria. *Infect Immun*. 1996 ; 64 :1054-1059.

22 Udhayakumar V, Kariuki S, Kolczack M, Girma M, et al., Longitudinal study of natural immune responses to the *Plasmodium falciparum* membrane antigen (AMA-1) in a holoendemic region of malaria in Western Kenya : Asembo Bay Cohort Project VIII. *Am J Trop Med Hyg*. 2001 ; 65 :100-107.

23 Krzych U, Lyon JA, Jareed T, Schneider I, et al., T lymphocytes from volunteers immunized with irradiated *Plasmodium falciparum* sporozoites recognize liver and blood stage antigens. *J. Immunol*. 1995 ;155 :4072-4077.

24 Heppner DG, Kester KE, Ockenhouse CF, Tornieporth N et al., Towards an RTS,S-based, multi-stage, multi-antigen vaccine against falciparum malaria : progress at the Walter Reed Army Institute of Research. *Vaccine* 2005 ;23 :2243-2250.

25 Stoute JA, Slaoui M, Heppner DG, Momin P et al., A preliminary evaluation of a recombinant circumsporozoite protein vaccine against *Plasmodium falciparum* malaria. *NEJM* 1997 ; 336(2) : 86-91.

26 Kester KE, McKinney DA, Tornieporth N, Ockenhouse CF et al., efficacy of a recombinant circumsporozoite protein vaccine regimens against experimental *Plasmodium falciparum* malaria. *J Infect. Dis*. 2001 ; 183 : 640-7.

27 Bojang KA, Milligan PJ, Pinder M, Vigneron L et al., Efficacy of RTS,S/AS02A malaria vaccine against *Plasmodium falciparum* infection in semi-immune adult men in The Gambia: a randomized trial. *Lancet* 2001; 358: 1927-1934.

28 Alonso PL, Sacarlal J, Aponte JJ, Leach A, et al., Efficacy of the RTS,S vaccine against *Plasmodium falciparum* infection and disease in young African children: randomised controlled trial. *Lancet* 2004; 364: 1411-20.

29 Alonso PL, Sacarlal J, Aponte JJ, Leach A et al., Duration of protection with RTS,S/AS02A malaria vaccine in prevention of *Plasmodium falciparum* disease in Mozambican children: single-blind extended follow-up of a randomised controlled trial. *Lancet* 2004; 366: 2012-2018.

30 Gordon DM, McGovern TW, Krzych U, Cohen JC, Schneider I, et al., Safety, immunogenicity, and efficacy of a recombinantly produced *Plasmodium falciparum* circumsporozoite protein / HBsAg subunit vaccine. *J Infect Dis.* 1995; 171:1576-85.

31 Chulay JD, Schneider I, Cosgriff TM, Hoffman SL, Ballou WR, et al., Malaria transmitted to humans by mosquitoes infected from cultured *Plasmodium falciparum*. *Am J Trop* *Med Hyg* 1986; 35: 66-68.

32 Wirtz RA, Ballou WR, Schneider I, et al., *Plasmodium falciparum:* Immunogenicity of circumsporozoite protein constructs produced in Escherichia coli. *Exp Parasitol* 1987; 63:166-72.

33 Vanderberg JV, and Gwadz RRW, The transmission by mosquitoes of plasmodia in the laboratory. Chapter 4 in:  Kreier J. (ed.) Malaria Vol. 2. Academic Press, New York, 1980; 180-1

34 Church LWP, Le TP, Bryan JP, Gordon DM, et al., Clinical manifestations of *Plasmodium falciparum* malaria experimentally induced by mosquito challenge*. J Infect Dis*. 1997; 175: 915-20.

35 Hermsen CC, Telgt DSC, Linders EHP, van de Locht LATF et al., Detection of *Plasmodium falciparum* malaria parasites in vivo by real-time quantitative PCR. *Mol Biochem Parasit*. 2001; 118: 247-251.

36 Andrews L, Andersen RF, Webster D, Dunachie S, et al., Quantitative real-time polymerase chain reaction for malaria diagnosis and its use in malaria vaccine trials. *Am J Trop Med Hyg*. 2005; 73:191-198.

37 Morgan E, Varro R, Sepulveda H, Ember JA et al., Cytometric bead array: a multiplexed assay platform with applications in various areas of biology. Clin Immun. 2004; 110(3):252-6.

38 Korbel DS, Newman KC, Almeida CR, Davis DM, Riley EM, Heterogeneous Human NK cell responses to Plasmodium falciparum-infected erythrocytes. J Immunol. 2005;175: 7466-7473.

Appendix A: World Medical Association Declaration of Helsinki

Recommendations guiding physicians

in biomedical research involving human subjects

Adopted by the 18th World Medical Assembly

Helsinki, Finland, June 1964

and amended by the

29th World Medical Assembly

Tokyo, Japan, October 1975

35th World Medical Assembly

Venice, Italy, October 1983

41st World Medical Assembly

Hong Kong, September 1989

and the

48th General Assembly

Somerset West, Republic of South Africa, October 1996

INTRODUCTION

It is the mission of the physician to safeguard the health of the people. His or her knowledge and conscience are dedicated to the fulfilment of this mission.

The Declaration of Geneva of the World Medical Association binds the physician with the words, "The health of my patient will be my first consideration," and the International Code of Medical Ethics declares that, "A physician shall act only in the patient's interest when providing medical care which might have the effect of weakening the physical and mental condition of the patient."

The purpose of biomedical research involving human subjects must be to improve diagnostic, therapeutic and prophylactic procedures and the understanding of the aetiology and pathogenesis of disease.

In current medical practice most diagnostic, therapeutic or prophylactic procedures involve hazards. This applies especially to biomedical research.

Medical progress is based on research which ultimately must rest in part on experimentation involving human subjects.

In the field of biomedical research a fundamental distinction must be recognised between medical research in which the aim is essentially diagnostic or therapeutic for a patient, and medical research, the essential object of which is purely scientific and without implying direct diagnostic or therapeutic value to the person subjected to the research.

Special caution must be exercised in the conduct of research which may affect the environment, and the welfare of animals used for research must be respected.

Because it is essential that the results of laboratory experiments be applied to human beings to further scientific knowledge and to help suffering humanity, the World Medical Association has prepared the following recommendations as a guide to every physician in biomedical research involving human subjects. They should be kept under review in the future. It must be stressed that the standards as drafted are only a guide to physicians all over the world. Physicians are not relieved from criminal, civil and ethical responsibilities under the laws of their own countries.

I. BASIC PRINCIPLES

1. Biomedical research involving human subjects must conform to generally accepted scientific principles and should be based on adequately performed laboratory and animal experimentation and on a thorough knowledge of the scientific literature.
2. The design and performance of each experimental procedure involving human subjects should be clearly formulated in an experimental protocol which should be transmitted for consideration, comment and guidance to a specially appointed committee independent of the investigator and the sponsor provided that this independent committee is in conformity with the laws and regulations of the country in which the research experiment is performed.
3. Biomedical research involving human subjects should be conducted only by scientifically qualified persons and under the supervision of a clinically competent medical person. The responsibility for the human subject must always rest with a medically qualified person and never rest on the subject of research, even though the subject has given his or her consent.
4. Biomedical research involving human subjects cannot legitimately be carried out unless the importance of the objective is in proportion to the inherent risk to the subject.
5. Every biomedical research project involving human subjects should be preceded by careful assessment of predictable risks in comparison with foreseeable benefits to the subject or to others. Concern for the interests of the subject must always prevail over the interests of science and society.
6. The right of the research subject to safeguard his or her integrity must always be respected. Every precaution should be taken to respect the privacy of the subject and to minimise the impact of the study on the subject's physical and mental integrity and on the personality of the subject.
7. Physicians should abstain from engaging in research projects involving human subjects unless they are satisfied that the hazards involved are believed to be predictable. Physicians should cease any investigation if the hazards are found to outweigh the potential benefits.
8. In publication of the results of his or her research, the physician is obliged to preserve the accuracy of the results. Reports of experimentation not in accordance with the principles laid down in this Declaration should not be accepted for publication.
9. In any research on human beings, each potential subject must be adequately informed of the aims, methods, anticipated benefits and potential hazards of the study and the discomfort it may entail. He or she should be informed that he or she is at liberty to abstain from participation in the study and that he or she is free to withdraw his or her consent to participation at any time. The physician should then obtain the subject's freely‑given informed consent, preferably in writing.
10. When obtaining informed consent for the research project the physician should be particularly cautious if the subject is in a dependent relationship to him or her or may consent under duress. In that case the informed consent should be obtained by a physician who is not engaged in the investigation and who is completely independent of this official relationship.
11. In case of legal incompetence, informed consent should be obtained from the legal guardian in accordance with national legislation. Where physical or mental incapacity makes it impossible to obtain informed consent, or when the subject is a minor, permission from the responsible relative replaces that of the subject in accordance with national legislation.

Whenever the minor child is in fact able to give a consent, the minor's consent must be obtained in addition to the consent of the minor's legal guardian.

1. The research protocol should always contain a statement of the ethical considerations involved and should indicate that the principles enunciated in the present Declaration are complied with.

II. MEDICAL RESEARCH COMBINED WITH PROFESSIONAL CARE
(Clinical research)

1. In the treatment of the sick person, the physician must be free to use a new diagnostic and therapeutic measure, if in his or her judgement it offers hope of saving life, re-establishing health or alleviating suffering.
2. The potential benefits, hazards and discomfort of a new method should be weighed against the advantages of the best current diagnostic and therapeutic methods.
3. In any medical study, every patient - including those of a control group, if any - should be assured of the best proven diagnostic and therapeutic method. This does not exclude the use of inert placebo in studies where no proven diagnostic or therapeutic method exists.
4. The refusal of the patient to participate in a study must never interfere with the physician–patient relationship.
5. If the physician considers it essential not to obtain informed consent, the specific reasons for this proposal should be stated in the experimental protocol for transmission to the independent committee (I, 2).
6. The Physician can combine medical research with professional care, the objective being the acquisition of new medical knowledge, only to the extent that medical research is justified by its potential diagnostic or therapeutic value for the patient.

III. NON-THERAPEUTIC BIOMEDICAL RESEARCH INVOLVING HUMAN SUBJECTS
(Non-clinical biomedical research)

1. In the purely scientific application of medical research carried out on a human being, it is the duty of the physician to remain the protector of the life and health of that person on whom biomedical research is being carried out.
2. The subjects should be volunteers ‑ either healthy persons or patients for whom the experimental design is not related to the patient's illness.
3. The investigator or the investigating team should discontinue the research if in his/her or their judgement it may, if continued, be harmful to the individual.
4. In research on man, the interest of science and society should never take precedence over considerations related to the well being of the subject.

Appendix B: Administrative Matters

Additional information regarding study partner responsibilities and procedures can be found in the Addendum to CRADA W81XWH-05-0028, writ March,2006.

I. Responsibilities of the Investigator

- To ensure that he/she has sufficient time to conduct and complete the study and has adequate staff and appropriate facilities which are available for the duration of the study and to ensure that other studies do not divert essential subjects or facilities away from the study at hand.
- To submit an up-to-date curriculum vitae and other credentials (e.g. medical license number in the United States) to the sponsor and—where required—to relevant authorities.
- To acquire the normal ranges for laboratory tests performed locally and, if required by local regulations, obtain the Laboratory License or Certification.
- To retain no clinical samples (including serum samples) on site without the express written informed consent of the subject and/or the subject’s legally authorized representative.
- To perform no other biological assays at the investigator site except those described in the protocol or its amendment(s).
- To prepare and maintain adequate case histories designed to record observations and other data pertinent to the study.
- To conduct the study in compliance with the protocol and appendices.
- To co-operate with a representative of USAMMDA in the monitoring process of the study and in resolution of queries about the data.

II. Protocol Amendments and modifications

- No changes to the study protocol will be allowed unless discussed in detail with GSK Biologicals' Clinical Project Manager and MVI and filed as an amendment/modification to this protocol.
- All amendment/modification to the protocol will be adhered to by the participating centre(s) and will apply to all subjects. Written approval of protocol amendments/modifications by the Human Use Review Committee (HURC), the IRB of record, is required prior to implementation.

**All amendments/modifications will be submitted through the WRAIR Office of Research Management for review by the Human Use Review Committees (HURC). Review by the HSRRB will be required if the amendment/modification increases the risk to the study participants. In addition, the MVI IRB must receive and approve such amendments/modifications.**

III. Data Entry

Onsite data entry will be accomplished in Clinical Trials Center using CRFs and source documents, which means that source information will be entered in to a computer at the investigational site. The site will be capable of modifying the data to assure accuracy with source documentation. All new/updated information will be reviewed and verified. At the conclusion of the study, the study data will be archived in accordance with internal procedures of USAMMDA for IND studies. The computers that house the data are password protected and access is solely gained by those authorized by the PI. In addition, the investigator will be provided with a CD-ROM of the final version of the data generated at the investigational site by CTC. Investigators at WRAIR, USAMMDA, MVI and GSK will have access to this data.

IV. Monitoring by USAMMDA (i.e., the Sponsor)

Monitoring visits by a professional representative of the sponsors will be scheduled to take place as close as possible to entry of the first participant, during the study at appropriate intervals and after the last participant has completed the study. It is anticipated that monitoring visits will occur at a frequency defined before study start.

These visits are for the purpose of confirming that USAMMDA sponsored studies are being conducted in compliance with the relevant Good Clinical Practice regulations/ guidelines, verifying adherence to the protocol and the completeness and accuracy of data entered on the CRF pages/data entry screens and Vaccine Inventory Forms. The monitor will verify CRF/data entry entries by comparing them with the source data/documents that will be made available by the investigator for this purpose. Data to be recorded directly into the CRF pages/data entry screens will be specified in writing preferably in the source documentation agreement form that is contained in both the monitor’s and investigator’s study file. For data entry, the monitor will mark completed and approved screens at each visit. The investigator must ensure provision of reasonable time, space and adequate qualified personnel for monitoring visits. Reports of monitored visits will be generated by the Sponsor and provided to PI and study partners GSK and MVI.

V. Archiving of Data

The investigator/ institution should maintain all study documentation until at least 2 years after the last approval of a marketing application in an ICH region and until there are no pending or contemplated marketing applications in an ICH region or at least 2 years have elapsed since the formal discontinuation of the clinical development of the investigational product. These documents should be retained for a longer period however if required by the applicable regulatory requirements or by an agreement with the sponsor. It is the responsibility of the sponsor to inform the investigator/institution as to when these documents no longer need to be retained. The investigator/ institution should take measures to prevent accidental or premature destruction of these documents.

Similarly, the sponsor-specific study documentation should be retained until at least 2 years after the last approval of a marketing application in an ICH region and until there are no pending or contemplated marketing applications in an ICH region or at least 2 years have elapsed since the formal discontinuation of clinical development of the investigational product. These documents should be retained for a longer period however if required by the applicable regulatory requirements or if needed by the sponsor. The sponsor should inform the investigator/institution in writing of the need for record retention and should notify the investigator/institution in writing when the study-related records are no longer needed.

VI. Audits

For the purpose of compliance with Good Clinical Practice and Regulatory Agency Guidelines it may be necessary for USAMMDA (or other representatives of the USMRMC) or the FDA to conduct a site audit. This may occur at any time from start to after conclusion of the study.

When an investigator signs the protocol, he agrees to permit USAMMDA and the FDA audits, providing direct access to source data/ documents. Furthermore, if an investigator refuses an inspection, his data will not be accepted in support of a New Drug Registration and/or Application.

VII. Confidentiality and Publication

WRAIR shall have the right to publish or permit the publication of any information or material relating to or arising out of the work after prior submission to GSK and MVI provided that if GSK or MVI shall so request WRAIR will delay publication for a maximum of six months to enable GSK and/or MVI to protect its rights in such information or material. Any proposed publication or presentation (e.g. manuscript, abstract or poster) for submission to a journal or scientific meeting, should be first submitted and agreed to by any other involved author(s) from WRAIR, USAMMDA, GSK and MVI prior to publication/presentation. GSK Biologicals and MVI will undertake to comment on such proposed documents within two weeks.

**WRAIR Specific Administrative Procedures**

I. Monitoring/Quality Assurance

Monitoring of this protocol will be performed. The study monitor will review case report forms and will compare them against source documents to verify accurate data collection, to evaluate adherence to Good Clinical Practices, and to ensure completeness, accuracy, and integrity of study data.

Copies of all regulatory documents will be on file in the WRAIR Department of Clinical Trials. Source documents will be archived in the WRAIR Department of Clinical Trials unless other long-term storage options are subsequently defined. Documentation of test article storage, inventory, and accountability will also be maintained at the clinical site.

The Principal Investigator will co-ordinate the responsibilities and duties of all the sub-investigators, as well as other collaborating personnel through periodic meetings, which may occur as often as two to three times weekly during the challenge phase of the protocol and less-frequently during the preceding immunization and follow-up phases.

II. Evaluations During and Following the Project

The medical evaluations of volunteers will be recorded by one of the physician investigators on standard forms. Blood samples for antibody tests will be obtained by appropriately trained individuals. Consent forms along with a copy of the final approved protocol will be retained indefinitely. The Volunteer Registry forms will be submitted through the Office of Research Management, WRAIR to the Office of Research Protections, and USAMMDA at the conclusion of the study.

III. Withdrawal from Protocol for Individual Participants

Volunteers will be allowed to withdraw from the study at any time. Every effort will be made to complete an exit physical examination, as well as assess standard safety laboratory tests (CBC, creatinine, ALT, and AST) prior to the participant’s withdrawal from the study. If the participant withdraws after the challenge phase, they must be tested for the presence of malarial parasites and may require treatment with antimalarials.

IV. Protocol Amendments

If protocol amendments are required, they will be submitted in writing through the WRAIR Office of Research Management for transmittal to the WRAIR Human Use Review Committee (HURC) for review and approval. If an amendment to a Greater Than Minimal Risk (GTMR) protocol does not increase the risk to subjects from that posed in the original protocol, the amendment can be reviewed and approved at the local level and then implemented. However, the amended protocol and related documents to include the HURC minutes documenting the review of the amendment, must be forwarded to the HSRRB and the MVI IRB for administrative review and for placement in the protocol file.

If an amendment to a GTMR protocol increases the risk to subjects from that posed in the original protocol, the amendment must be submitted to the HSRRB and MVI IRB after review and approval at the local level, but before implementation. This amendment will receive either full HSRRB review or expedited review by the Acting Chair, as appropriate. The amendment may be implemented after HSRRB and MVI IRB approval. If necessary, volunteers will be provided with a revised informed consent document for their signature.

V. Disposition of Unused Medications

Unused investigational vaccine doses will be accounted for and will be returned to the manufacturer for safekeeping or disposed of according to the manufacturer’s policy.

VI. Use of Information and Publications Arising from this Study

It is anticipated that the results of this study will be presented to the scientific community via oral presentations at meetings and written publications in scientific journals. The official final report will be submitted through appropriate channels at WRAIR to the HSRRB and USAMMDA at Ft. Detrick, MD and to GSK and MVI. This report will contain detailed information about the volunteers, their tolerance of the vaccines, their side effects and laboratory abnormalities, as well as their overall immune responses to immunization. In addition, the report will highlight the protective efficacy of the vaccine against malaria, as well as any lessons learned from the study. This trial will also be registered in *www.clinicaltrials.gov*.

It is the policy of the U.S. Army Medical Research and Materiel Command that data sheets are to be completed on all volunteers participating in research for entry into this Command’s Volunteer Registry Data Base. The information to be entered into this confidential database includes name, address, study name, and dates. The intent of the database is two-fold: first, to readily answer questions concerning an individual’s participation in research sponsored by USAMRMC; and second, to ensure that the USAMRMC can exercise its obligation to ensure research volunteers are adequately warned of risks and to provide new information as it becomes available. The information will be stored at USAMRMC for a minimum of 75 years.

VII. Payment to volunteers:

Volunteers will be paid for his or her participation in this study. Payment will occur at each visit to compensate for time and effort provided by the volunteer.

**Compensation:**

Screening visit with blood draw is $25.00

Upon entry into the study, payment is $100.00 for each blood draw, excluding unscheduled clinical labs drawn for volunteer safety. During the hotel phase, the amount is $100.00 per day. The payments will be grouped to provide compensation after 2 – 4 completed blood draws. Active duty personnel will be paid $50 per blood draw, unless the visits occur during off duty hours or they are on leave. In this case, they will be paid the same as non-military personnel. All scheduled blood draws are planned to occur during off duty hours.

Approximate breakdown:

Screen - $25.00

Immunization only cohort (1/5^th^ dose)- $700

Immunization cohort (full dose) with challenge- $3900.00

$900 for Immunization Phase (2 additional draws versus 1/5^th^ dose group for microarray analysis)

$3000 in Challenge Phase if totally protected. (The amount will most likely be less.)

Control cohort – Most likely $1500-1900 (maximum $2200.00)

Assuming compliance with all follow-up visits, blood draws, and malaria challenge (with a hotel stay of 10 nights), a volunteer for one of the vaccine cohorts may receive as much as much as $3925 and their participation may be up to 8 months in duration, including screening. Infectivity controls may receive as much as $2225 for their participation in the study which may be up to 3 months in duration.

*Appendix C: Overview of the recruitment plan*

Approximately forty-one healthy adult volunteers from the military and civilian staff of WRAIR/WRAMC as well as non-governmental civilians will be recruited (although as many as 60 may be screened) by non-coercive means through the WRAIR Clinical Trials Department, according to existing U.S. Army regulations (AR 70-25 and AR 40-38).

Any advertisements used specifically related to this study will be submitted to the WRAIR Office of Research Management and MVI IRB for the appropriate approvals prior to their use. Recruitment will be by general advertisement and word of mouth. Upon calling the protocol volunteer recruitment phone number, a recruitment script will be used to inform potential volunteers of the details of the study. An appointment for more information and screening will be made at that time. The volunteer will then report to the CTC at WRAIR for briefing and screening. Upon full review of the exclusion/exclusion criteria, the decision for enrollment of the volunteer will be made by the PI.

Coercion will be reduced by not paying volunteers for visits, but rather, remuneration will be for blood draws. The study will be performed in compliance with GCP and adherence to the CFR Guidelines.

PROPOSED RECRUITING SCRIPT FOR VOLUNTEERS UNDERGOING VACCINATION:

This study is researching malaria vaccines.

**Background:** Malaria is a disease that can range from mild illness to death. Humans get this disease by being bitten by mosquitoes that are infected with malaria parasites. While not common in America, it is in places such as Africa, Asia and Latin America. There are 300 – 500 million clinical cases and 1.5 – 2.7 million deaths annually. Much of the malaria now seen in the world has become resistant to drugs used in the past. A vaccine against this disease would be beneficial both to people living in, or traveling to, areas where it is commonly found. The military is working in conjunction with civilian companies to produce a vaccine because it would benefit soldiers going to areas where malaria is common.

**Objectives:**

Primary, to look at a new investigational malaria vaccine for safety and immune system response.

Secondary, some volunteers will be involved in the part of the study to see if these vaccines either prevent or postpone malaria.

**Vaccine Information:**

The vaccines consist of FMP2.1, which contains a portion of malaria protein that is produced by the malaria parasite. This piece is not the malaria germ itself, and cannot cause the disease. It is combined with either the AS01B or AS02A adjuvant, a substance intended to stimulate your immune system.

The vaccine has been made under conditions acceptable to the FDA.

The vaccines are not licensed by the FDA; it is investigational and undergoing evaluation in the United States.

The FMP2.1/AS02A vaccine has been tested in human volunteers prior to this study. The FMP2.1/AS01B has not, although this formulation has been found to be safe in animals.

**Duration:**

Vaccine Group: Participation may be up to 5 months, not including screening.

Screening – up to 3 months.

Immunization phase – approximately 2 months.

Primary challenge – an additional 3 months

Control Group: Screening - up to 3 months. Study participation - up to 3 months.

**Number of people in the study:**

Vaccine Group: (we will be testing a low dose of one vaccine in a small group of volunteers first)

Low Dose Group: five volunteers to receive 1/5^th^ dose of one of the vaccines

Full Dose Group: two groups totaling up to 30 people (Group A: 15 people, Group B 15 people with a minimum of 6 in each group) to receive full dose of the vaccine.

Control Group: 6 volunteers will be recruited to act as controls for each challenge phase.

Alternate volunteers will be recruited to participate for each cohort; they will step in if a regularly scheduled volunteer is unable to participate.

**Screening Phase** includes an:

In depth explanation of the study by a study investigator,

Informed consent review and sign,

Comprehension assessment by written test, with review as needed,

Brief medical history, medical exam, urine, and blood testing.

**Immunization Phase:**

There are three immunizations, with one given at approximately 0-, 1- and 2- months. The vaccine is given through a needle into a muscle in your upper arm.

Possible side effects of the immunizations are redness, warmth, and/or soreness at the site of injection.

A study physician will see you at each visit. You will have blood drawn on several of the visits.

**Challenge Phase:**

There are no blood tests to see if the vaccines are effective. In order to test if they are effective, once the immunization phase of this study is over, we will ask some of the volunteers who received the full dose of vaccine to undergo a challenge against malaria. The volunteers receiving the low dose of the vaccine will not be able to go on to challenge. If you are not undergoing malaria challenge, either because you received the low dose or you are not selected from full dose group, your participation in the study is complete. If you are in the full dose group, you can also choose not to be in the challenge group. If you are chosen to undergo challenge, you should know:

- The challenge entails being bitten by a mosquito that is infected with malaria in a carefully controlled setting here at Walter Reed. **YOU MAY CONTRACT MALARIA FROM THE MOSQUITO BITE.**
- The strain of malaria used in this study will not recur after treatment.
- The mosquitoes are contained in a small cup with a fine mesh screen on top, which is placed against your arm for five minutes, then removed.
- This procedure doesn’t hurt, but your arm may itch at the site later, as with any mosquito bite.

**Post-Challenge / Hotel Phase:**

Approximately five days after the challenge, we will need to see you in the Clinical Trials Center during which you will have a small amount of blood drawn to check for malaria.

Malaria symptoms may include a fever, headache, fatigue, vomiting, diarrhea, muscle aches, and / or stomachache.

Then, at approximately day 9 through day 20 after challenge, all volunteers and study staff will check into a local area hotel. This is done to offer the fastest available assessment and treatment if needed.

During the hotel phase, you can come and go for work or outside activities, but we ask that you sleep at the hotel. That way, for example, if you didn’t feel well during the night, you wouldn’t have to wait for the clinic to open; you would be seen immediately.

If you do develop malaria at any time, the strain used in this study is very sensitive to a drug called chloroquine. This is an oral drug that you take for three days. This drug has few side effects, which may include an upset stomach, nausea, and/or diarrhea.

Once treated, you would then be monitored weekly for four weeks in a row and complete the study.

Again, the strain of malaria used in this study will not recur after treatment.

If you develop malaria, you will not be able to donate blood for three years.

If you do not become positive for malaria during the hotel stay, you will be checked daily at the Clinical Trials Center to day 30 post-challenge (excluding weekends / holidays). After that, we will check you weekly for four weeks in a row.

**Approximate compensation breakdown:**

Screen - $25.00

For the Immunization Phase, payment is $100.00 for each blood draw, excluding unscheduled clinical labs drawn for volunteer safety. During the hotel phase, the amount is $100.00 per day for the days you spend in the hotel. The payments will be grouped to provide compensation after 2 – 4 completed blood draws.

Immunization only group (1/5^th^ dose)- $700

Immunization group (full dose) with challenge –

$900 for Immunization Phase (2 additional blood draws versus 1/5^th^ dose group for microarray analysis)

Most likely $1500- $2200 for Challenge Phase (maximum amount possible $3000)

Challenge control cohort – most likely $1500-$1900 (maximum $2200)

**Inclusion criteria:**

Healthy adult, 18 – 50 years of age,

Male or female, military or civilian.

**Exclusion criteria:**

History of malaria or known exposure to malaria in past 12 months,

Allergy to components of vaccines or malaria drugs,

HIV, immunodeficiency diseases (cancer, transplant patient)

Severe cardiac, kidney, or liver disease (hepatitis),

Pregnancy or planned pregnancy,

Use of certain prescription medications,

Any other significant finding that in the opinion of the investigator would increase the risk of having an adverse outcome from participating in this study.

Would you like to come into the Clinical Trials Center to hear further information about this study?___________________________

Listening to the general inclusion and exclusion criteria, do you believe you meet those criteria? _______________________

Name: ____________________________________________

Contact Phone Number:_________________

Work Phone Number: __________________

Alternate Phone Number: ________________

E-mail:_______________________________

How did you learn about us? _____________________________________________________

We will enter this information into a private, not-for-release database. May we use this to contact you about future studies? ____________________________

Recruiter’s signature: ____________________________________________

Date: _________

PROPOSED RECRUITING SCRIPT FOR CHALLENGE CONTROLS

This study is researching malaria vaccines.

**Background:** Malaria is a disease that can range from mild illness to death. Humans get this disease by being bitten by mosquitoes that are infected with malaria parasites. While not common in America, it is in countries such as Africa, Asia and Latin America. There are 300 – 500 million clinical cases and 1.5 – 2.7 million deaths annually. Much of the malaria now seen in the world has become resistant to drugs used in the past. A vaccine against this disease would be beneficial both to people living in, or traveling to, areas where it is commonly found. The military is working in conjunction with civilian companies to produce a vaccine because it would benefit soldiers going to areas where malaria is common.

This vaccine study is done in two parts.

The first part, already underway, involved a group of people who volunteered to get the investigational vaccine. There isn’t a blood test to see if the vaccine works, so the second part involves exposing some of those volunteers who received the vaccine to malaria infected mosquitoes. This second part is called a ‘challenge’. If they don’t become positive for malaria, we can assume either the vaccine worked, or the mosquitoes were not able to transmit malaria. To ensure the mosquitoes can transmit malaria, we ask volunteers who haven’t been vaccinated to participate as a ‘control’ group, and only take part in the challenge, or exposure to malaria infected mosquitoes. If the control volunteers become positive, and the vaccinated volunteers don’t, then we know the vaccine worked.

Objective:

You may help validate the malaria challenge model

You are being asked to participate in the control group, and you **WILL NOT RECEIVE ANY VACCINE**. We will allow mosquitoes that are infected with malaria to bite you under controlled conditions, and **WE EXPECT YOU TO DEVELOP MALARIA FROM THE MOSQUITO BITE**. We will provide treatment to cure your infection.

**Vaccine Information:**

You will not receive any malaria vaccine.

**Duration:**

Control Group: Screening - up to 3 months. Study participation - up to 3 months.

**Number of people in the study:**

Control Group: 6 volunteers will be recruited to act as controls for each challenge phase.

Alternate volunteers will be recruited to participate for each cohort; they will step in if a regularly scheduled volunteer is unable to participate.

**Screening Phase** includes an:

In depth explanation of the study by a study investigator,

Informed consent review and sign,

Comprehension assessment by written test, with review as needed,

Brief medical history, medical exam, urine, and blood testing.

**Challenge Phase:**

There are no blood tests to see if the vaccines are effective. In order to test if they are effective, we will ask some of the immunized volunteers to undergo a challenge against malaria. You are a control group meaning you will also be challenged with malaria and he results compared to the immunized volunteers who were challenged.

- The challenge entails being bitten by a mosquito that is infected with malaria in a carefully controlled setting here at Walter Reed. **YOU MAY CONTRACT MALARIA FROM THE MOSQUITO BITE**.
- The strain of malaria used in this study will not recur after treatment.
- The mosquitoes are contained in a small cup with a fine mesh screen on top, which is placed against your arm for five minutes, then removed.
- This procedure doesn’t hurt, but your arm may itch at the site later, as with any mosquito bite.

**Post-Challenge / Hotel Phase:**

Approximately five days after the challenge, we will need to see you in the Clinical Trials Center during which you will have a small amount of blood drawn to check for malaria.

Malaria symptoms may include a fever, headache, vomiting, diarrhea, fatigue, muscle aches, and / or stomachache.

Then, at approximately the evening of Day 9 through Day 20 after challenge, all volunteers and study staff will check into a local area hotel. This is done to offer the fastest available assessment and treatment if needed.

During the hotel phase, you can come and go for work or outside activities, but we ask that you sleep at the hotel. That way, for example, if you didn’t feel well during the night, you wouldn’t have to wait for the clinic to open; you would be seen immediately.

If you do develop malaria at any time, the strain used in this study is very sensitive to a drug called chloroquine. This is an oral drug that you take for three days. This drug has few side effects, which may include an upset stomach, nausea, and/or diarrhea.

Once treated, you would then be monitored weekly for four weeks in a row and complete the study.

Again, the strain of malaria used in this study will not recur after treatment.

If you develop malaria, you will not be able to donate blood for three years.

If you do not become positive for malaria during the hotel stay, you will be checked daily at the Clinical Trials Center to day 30 post-challenge (excluding weekends / holidays). After that, we will check you weekly for four weeks in a row.

**Compensation:**

Screening visit with blood draw is $25.00

Upon entry into the study, payment is $100.00 for each blood draw, excluding unscheduled clinical labs drawn for volunteer safety. During the hotel phase, the amount is $100.00 per day for the days you spend in the hotel. The payments will be grouped to provide compensation after 2 – 4 completed blood draws.

**Approximate** breakdown:

Screen - $25.00

Challenge control cohort – Most volunteers will earn from $1500 to $1900 (with the maximum possible being $2200).

**Inclusion criteria:**

Healthy adult, 18 – 50 years of age,

Male or female, military or civilian.

**Exclusion criteria:**

History of malaria or known exposure to malaria in past 12 months,

Allergy to components of vaccines or malaria drugs,

HIV, immunodeficiency diseases (cancer, transplant patient)

Severe cardiac, kidney, or liver disease (hepatitis),

Pregnancy or planned pregnancy,

Use of certain prescription medications,

Any other significant finding that in the opinion of the investigator would increase the risk of having an adverse outcome from participating in this study.

Would you like to come into the Clinical Trials Center to hear further information about this study?___________________________

Listening to the general inclusion and exclusion criteria, do you believe you meet those criteria? _______________________

Name: ____________________________________________

Contact Phone Number:_________________

Work Phone Number: __________________

Alternate Phone Number: ________________

E-mail:_______________________________

How did you learn about us? _____________________________________________________

We will enter this information into a private, not-for-release database. May we use this to contact you about future studies? ____________________________

Recruiter’s signature: ____________________________________________

Date: _________

**TELEPHONE INTAKE FORM**

Last Name: ___________________ First Name:_________________ MI:_____

Male  Female  Mil  Civ 

Home Phone: {______}__________________ best time to contact: __________

Work Phone: {______}__________________ best time to contact: __________

Other Phone: {______}___________________ best time to contact: __________

E-mail: ________________________________________________________________

How did you hear about us? ________________________________

Have you participated in clinical research studies? Yes  No 

Comment if Yes: _________________________________________________________

TO PARTICIPATE IN THE STUDY:


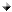
 You must be within the study age requirements, 18-50 years.


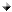
 You must be in good health.


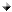
 You must not currently be under treatment for any clinically significant medical conditions.


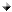
 You must not abuse drugs, tobacco or alcohol.


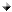
 You must not ever have tested positive with HIV or Hepatitis B or C.


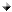
 Some medications may disqualify you from participating.


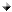
 If you are a female, you cannot be pregnant or plan on becoming pregnant during the study.

Study: _____________________________________________

Briefing Date & Time: ________________________________

*The information you just provided us will be entered into our private database. All information is considered confidential.*

*May we call you for future studies if they become available? Yes  No *

Recruiter’s Signature: __________________________Date: ________________

Appendix D: Laboratory Assays

***Hematology/biochemistry:***

A complete blood count (CBC), renal and liver function tests will be measured at regular intervals throughout the study period. Performing laboratory reference values will be maintained by the Principal Investigators and copied to the USAMMDA study monitors at start of screening.

***Procedure for the Laboratory Diagnosis of Malaria in Human Volunteers***

Beginning on day 5 post-challenge, each volunteer will undergo daily phlebotomy for approximately 2 mL of venous blood into an EDTA tube with a twist off cap. The tube will be inverted approximately ten times and sent to the Parasitology Laboratory maintained by the WRAIR Department of Immunology.

There, the tubes will be inverted approximately ten additional times and 20 μl of blood will be withdrawn for preparation two thick films (10 μl) on one glass slide. The slides will be placed horizontally on a table and allowed to dry at room temperature with the aid of a fan. Once dry, the slides will be placed upright in a slide holder. When dry, the slides will be subjected to a stream of warm air from a heat source for twenty minutes to further dry the thick blood smears.

The smears will then be inverted in a staining rack and stained for either 45 - 60 minutes in a 4% solution or for 10 minutes in a 10% solution of Giemsa in phosphate buffer (pH 7.0-7.2). The slides will then be individually washed in a beaker under running tap water until remnants of excess stain are removed.

The slides will then be returned to the slide holder and dried in a stream of room temperature air.

The stained thick smears will be read by viewing 200 oil immersion microscope fields for the presence or absence of malaria parasites. Blood from symptomatic volunteers will undergo a thorough search with at least 1000 oil immersion fields examined for malaria parasites.

The detection of malaria parasites in thick blood smears will be made by a slide reader and the diagnosis will be confirmed by an expert microscopist. Diagnosis requires identification of at least two malaria parasites, both confirmed by the expert microscopist. After confirmation of a positive blood smear, notification will be sent to the physician on call. Slide readers will not have access to the group assignments of the volunteers.

After treatment for malaria, blood smears will be processed daily until three consecutive negative blood smears are recorded.

Volunteers will then be followed by a weekly blood smear for four weeks.

Results from each blood smear will be recorded in a laboratory notebook, as well as positive results on a Blood Smear Report form (Appendix N), along with the time of notification of a study physician. All blood smears will be archived in the WRAIR Department of Immunology for future reference. Initial blood samples will be refrigerated until verification of correct staining has occurred.

**Humoral Responses**

Serological responses to malaria antigens will be performed mainly in the Serology Laboratory of the WRAIR Department of Immunology. Serum for antibody determination will be collected at indicated time points. Blood for analysis of antibody responses will be obtained from each volunteer and allowed to clot for one hour at room temperature, and serum will be separated and frozen at -20°C (+/- 5°C) or below until tested.

***Serology***

Immunogenicity (antibody levels) will be determined at the MSL at WRAIR by evaluating antibody (IgG) responses to the P. falciparum FMP2.1 antigen as measured using standard ELISA methodologies with appropriate capture antigens. ELISAs will be performed in triplicate serial dilution, and the titer defined as the serum dilution required to yield an optical density of 1.0 in our assay. Antibody levels with 95% confidence intervals will be determined. The Serology Laboratory of the WRAIR Department of Immunology maintains a quality assurance log that outlines the performance of this particular assay over time.

**pLDH Growth inhibition assay (GIA), WRAIR**

Samples are tested according to the method developed by Miura et al (*Mol Biochem Parasitol., in press*). Briefly, antisera (pre-absorbed with human RBC) were tested at 20% for growth inhibition as determined by measuring the LDH activity of the parasites. For this purpose, pRBC cultures of both the 3D7 variant (homologous) and FVO variant (heterologous) at the early schizont stage were setup with pre-immune and immune sera at various concentrations at a 0.3% parasitemia (1% Hematocrit). Assay plates were sealed in bags containing 2.5% CO2, 2.5% O2, 90% N2 and incubated for 40hrs (cycle time of 3D7 under these experimental conditions). Cultures were then harvested by transferring 50µl/well into PBS-containing C-bottom plates and washed by spinning plates for 10min at 10,000g. Then, plates were frozen at -30C until analysis. To measure the extent of LDH activity in the various plates, a substrate buffer containing 0.1M Tris HCl, 50mM Sodium-L-lactate, 0.255 Triton-X, 10mg NBT, 10µg/ml 3-Acetylpyridine, 10U/ml diaphorase from *Clostridium klyiveri* (all reagents were obtained from Sigma, St.Louis, MO) was added to the plates. Colorimetric measurement at 650nm was done after 30min reaction time using the SpectraMax Plus 384 (Molecular Devices, Sunnyvale, CA). Calculation of growth inhibition was determined by using the formula: % inhibition = 100%-(((OD immune sample- OD RBC only)/(OD pre-immune control – OD RBC only))*100).

***Growth Inhibition Assays (GIA), WRAIR***

This procedure assays the ability of malarial antigen immunized sera to inhibit the in vitro invasion of red blood cells and growth of *Plasmodium falciparum* cultures as an exploratory assay. Sera were tested in a growth inhibition assay (GIA) using homologous 3D7 *P. falciparum* parasites in a one-cycle static GIA. The sera will be heat inactivated and dialyzed against culture media made pH 7.4 with sodium hydroxide (RPMI-NaOH). Sera (20% v/v) will then be cultured 2 days with 0.2% initial parasitemia (trophozoites) 4% hematocrit in triplicate 150ul static cultures in 48-well plates. Cultures are harvested and stained with Hoechst dye 33342 and the new trophozoites counted in 40,000 erythrocytes by flow cytometry. Inhibition is calculated from final parasitemias as Inhibition = (control-test)/control, where control is the final parasitemia with pre-immune serum and expressed as a percent.

***Growth Inhibition Assays (GIA), MVDU/NIH***

Serum will be sent to Malaria Vaccine Development Branch at NIH, Rockville, Maryland. Growth Inhibition Assays will be performed there according to the site-specific protocol.

Rationale for multiple Growth Inhibition Assays

Although it is known that both humoral and cell-mediated immunity play a role in protection against malaria, there is no defined correlate of immunity identified in either naturally exposed populations or vaccinated individuals. Therefore, in current studies, both epidemiological and interventional, evaluating a variety of assays will contribute information about the immune response to malaria infection and perhaps lead to a definition of immunological correlates. Each of the three GIA assays listed in this protocol has specific methodologies and thus represents an opportunity to provide unique as well as bridging information regarding the inhibitory antibody response generated by these experimental malaria vaccines.

The pLDH GIA assay performed in the laboratory of Dr. Evelina Angov of WRAIR is the assay that will be used to determine one of the secondary endpoints of this trial: whether the anti-AMA-1 antibodies generated are functional in inhibiting growth of heterologous and homologous parasites. This pLDH assay was originally developed by Dr. Carole Long of the NIH and has been adopted at WRAIR in order to establish a standardized GIA among the two institutions. The strength of the pLDH assay performed by both the NIH and WRAIR is that it measures growth inhibitory properties of antibodies in the context of viable parasites.

The NIH GIA assay performed in the laboratory of Dr. Carole Long differs from the WRAIR assay in one respect -total immunoglobulin is purified from serum samples, thus potentially removing any toxins or non-specific inhibitors. It also permits the researcher to have more control over immunoglobulin concentrations used in the assay. One drawback with this procedure is that greater volumes of serum are required to purify the immunoglobulin. This will be particularly relevant in pediatric malaria vaccine trials where the amount of serum available is limited. Currently the NIH laboratory is preparing to become the reference center for evaluating growth inhibitory activities from malaria blood-stage vaccine clinical trials. Evaluation of clinical samples by the reference laboratory will allow direct comparability of various vaccine candidates for their potential to induce biologically relevant functional activities.

The 3^rd^ GIA assay planned is experimental and being developed at WRAIR by Dr. David Haynes. The method quantifies inhibition by parasite-DNA dye binding and flow cytometry. This assay was used in previous Phase 1 vaccine trials using FMP2.1/AS02A to quantify parasite growth inhibitory activities and in combination with the antibody levels measured by ELISA, will provide the necessary bridge to the results from the current Phase 1/2a trial.

***Merozoite Immunofluorescent Antibody Assay (IFA)***

Immunofluorescence Antibody assay (IFA) is a method to visualize AMA-1 antibodies located on the infected red blood cell. IFA titers against whole merozoites and parasitized red blood cells (PRBC) will also be measured in all volunteers pre- and post-immunization following standard procedures. A volunteer will be considered a seroconverter by IFA if the volunteer develops an antibody titer (as measured by the highest IFA titer that gives a positive reaction) on the day of challenge that is greater than four times that of the preimmune serum. IFA assessments will be performed in the WRAIR Department of Immunology.

The following method is used to detect and quantify anti-merozoite antibody in serum.

- 1. Preparing Slides: Schizonts from culture (4-6% parasitemia, 1.5% hematocrit) are centrifuged at 350g for 10 minutes. The supernatant is removed with a pipette. The cell pellet is washed 10 mL RPMI and pelleted as above. The hematocrit is adjusted to 1% with 1X PBS. The cell suspension is spotted onto multitest slides and fixed by storing in methanol at –20^o^C. (Absolute methanol, the bottle should never have previously been opened or used.)
  2. IFA Assay: After removal from methanol, the slides are dried under an air stream and blocked by adding 15 µL of PBS/1%BSA. The sera (pre-diluted) are added quickly by exchanging the blocking buffer with the sera. (In previous studies with a different blood-stage antigen (FMP1), sera obtained prior to the 21st week were diluted starting with 1:500 and up to 1: 32,000. Following week 21, sera were diluted starting from 1: 1,000 and up to 1: 64,000.) After incubation at room temperature in a humidified chamber for 1.5 hours, the spot is exchanged with 15 µL PBS/1%BSA and soaked in a Coplin jar filled with 1X PBS (2x). The second antibody (FITC-anti human IgG) is diluted 1:200 in PBS/1%BSA (600 µL for each slide). The second antibody is added and the slide is incubated for 1 hour in a humidified chamber in the dark. The slides are washed three times in a Coplin jar filled with 1X PBS and dried under air and by aspiration. The slides are then mounted with Fluoromount-G (Fisher Biotech) and the cover slip is applied. The slides are read with a UV scope at 1600X magnification.

Any serum not immediately used in antibody assays will be stored at -70°C (± 5°C) or colder and may be used in additional in vitro tests which may be useful in optimizing the follow-up of study volunteers or in evaluating the immune response to the test vaccine preparation in these volunteers.

***Processing Inhibition Assay***

This test measures the ability of anti-AMA-1 antibodies to inhibit AMA-1 processing and therefore localization and function. An appropriate volume of heat inactivated serum (dialyzed against RPMI 1640 or used directly after heat inactivation) will be mixed with 135 mL of synchronized mid-stage (~8 nuclei), >90% pure schizonts (1x10^7^ per mL) in a 48 well culture plate. The plate will be placed in a plastic bag, gassed with 5% O_2_-5% CO_2_, heat-sealed, and incubated at 37^o^C for ~6 h. Aliquots from a control culture flask will taken to monitor the percent rupture of schizonts by hemocytometer. After ~90% schizonts have ruptured, the contents of each well will be transferred to a microfuge tube and centrifuged at 10,000 ×g for 5 min. The supernatant will be aspirated and the resulting parasite pellet will be washed with 0.5 mL chilled PBS and centrifuged as before. The supernatant will be discarded and 150 mL of 1X NuPAGE sample buffer (Invitrogen®) will be added to the tubes; samples will be frozen at –30^o^C until analyzed. The AMA-1 processing will be monitored using western blotting. A positive control rabbit serum will also be used in the assay.

***Additional serologic assays***

Serologic responses to the FVO variant of AMA-1, subdomains of AMA-1, as well as immunoglobulin isotype analysis are of interest, therefore, titers may be determined by ELISA by similar methods described previously in this section.

**Cell Mediated Immunity (CMI)**

In addition to assessment of humoral immune responses, cellular assays will also be performed. These assays will include peripheral blood mononuclear cells (PBMC) and cytokine measurements by ELISPOT and intracellular cytokine staining following *in vitro* stimulation with antigen or derived-peptides. These studies will be performed in the WRAIR Department of Immunology using standard methods. All assays will be performed in a blinded manner without regard to the investigational group assignment of the volunteer participants.

***Peripheral Blood Mononuclear Cells***

Peripheral blood mononuclear cells (PBMC) will be isolated from whole blood cells by LSM (Lymphocyte Separation Medium, Organon Teknika Corp, Durham, NC) or the equivalent, Ficoll-Hypaque gradient centrifugation. 10^7^ PBMC/vial will be frozen for subsequent CMI analysis. PBMC will be re-suspended in 1 mL of 90% cold fetal calf serum and 10% DMSO. Cells are then frozen slowly to -70°C (+/- 5°C) or colder and transferred to liquid nitrogen for storage.

***Detection of AMA-1--specific γ-interferon secreting cells, ELISPOT technique:***

The ELISPOT technique is a 96-well plate method where peripheral blood mononuclear cells are incubated with test antigens or peptides. The cytokines secreted by antigen-specific T cells is captured by immobilized antibody and subsequently detected by colorimetric substrate as localized spots on the membrane coated wells.

Specific interferon-γ secreting cells will be determined following a culture period in the presence of plasmodium specific antigens. Purified PBMC will be re-suspended at 2 x 10^6^ cells/mL in culture medium containing 5% human AB serum. Triplicate cultures of 100 µl each will be stimulated with recombinant AMA-1 or medium control for 24 hours at 37°C in 96 well microtiter culture plates. Subsequently the cells will be transferred into the corresponding wells of a 96 well Elispot plate that was pre-coated with 1-DIK (anti-γ-interferon monoclonal antibody). After a second 18-hour culture at 37°C the cells will be washed out and a second γ-interferon-specific biotinylated monoclonal antibody will be to the wells. Binding will be detected using a streptavidin-alkaline phosphatase conjugate plus the appropriate substrate. Results will be expressed as the number of spots per 10^6^ cells tested.

***ICS (Intracellular cytokine staining)***

Cytokines will also be measured by intracellular staining using fluorochrome-labeled, cytokine-specific antibodies. PBMC will be cultured with recombinant AMA-1 and, 20 hours prior to cell harvest, intracellular block (e.g., Brefeldin A) will be added to inhibit cytokine secretion. Cells will subsequently be harvested, stained for surface markers (e.g., CD4 or CD8 for T cells) and then fixed and permeabilized with CytoFix and Cytoperm (Pharmingen). Cells will then be stained with either Flourochrome-labeled cytokine-specific antibody or Flourochrome-labeled isotype matched control. The antibody forms complexes with the cytokine within the cytoplasm and can be detected by cytometry. Cells will then be washed, re-suspended in PBS and analyzed by flow cytometry. Cytokines of interest include IFN-gamma, IL-2, TNF-alpha, although which cytokines will be tested will be dependent on PBMC recovery and cell left after use in the priority assay, IFN-gamma ELISPOT. For intracellular staining (ICS) assays, the data will be presented as the percentage of Ag-activated CD4+ or CD8+ T cells producing cytokines. ICS is evaluated using flow cytometry. The results will be analysed descriptively and presented as number of Ag-specific CD4 or CD8 T cells expressing IFNγ and/or TNF-α and/or IL-2 respectively per million of CD4 T cells and per million of CD8 T cells.

**Experimental Assays**

***Sporozoite Immunfluorescence Assay (sporozoite IFA)***

*Plasmodium falciparum* sporozoites are obtained from the salivary glands of infected *Anopheles stephensi* mosquitoes through the Department of Entomology at WRAIR and are then acetone-fixed. Volunteer sera diluted in 2% fetal bovine serum containing PBS is then added to the slide and incubated for 2 hours. FITC-conjugated secondary antibodies diluted 1:500 (Southern Biotech, Birmingham, AL, USA) are added and slides are mounted in SlowFade® Antifade Kit soluntion (Molecular Probes, Inc. Eugene, OR, USA). An Olympus BX50 microscope equipped with a mercury epifluorescence lamp, a 100X (oil) objective and a multi filter cube are used to observe the fluorescence. To determine IFA titers, serial dilutions of antibody with dilutions from 1:2 up to1:25600 are tested.

***High-Density Oligonucleotide Microarrays*** Blood Collection: Blood will be collected in Paxgene collection tubes for RNA stabilization and processed as described the manufacturer (Becton Dickinson & Qiagen). Six mL of blood will be collected on Day 0, Day 56 (immunization #3), Day 57, Day 59 for volunteers, and day of challenge (DOC) and post-challenge days 1 and 5 for volunteers and non-infectivity controls. Methodology: briefly, poly(A) mRNA is prepared from Paxgene blood collection tubes and extracted with Trizol reagent (Life Technologies, Gaithersburg, MD) and used to create cDNA with a T7-polyT primer and the reverse transcriptase Superscript II (GIBCO/BRL). Approximately 5 µg of cDNA is subjected to *in vitro* transcription (Ambion, Austin, TX) in the presence of biotinylated UTP and CTP (Enzo Diagnostics). Target for hybridization will be prepared by combining 40 µg of fragmented transcripts with sonicated herring sperm DNA (0.1 mg/mL) and 5 nM control oligonucleotide in a buffer containing 1.0 M NaCl, 10 mM Tris·HCl (pH 7.6), and 0.005% Triton X-100. Target will be hybridized for 16 hr at 40°C to a set of oligonucleotide arrays (Affymetrix, Santa Clara, CA) containing probes for > 12 000 human genes. Arrays will be washed at 50°C with 6_ SSPET (0.9 M NaCl/60 mM NaH_2_PO_4_/6 mM EDTA/0.005% Triton X-100, pH 7.6), then at 40°C with 0.5_ SSPET. Arrays will then be stained with streptavidin-phycoerythrein. Fluorescence intensities will be captured with a laser confocal scanner and analyzed with the GENECHIP software (Affymetrix). Expression data will be analyzed using Molecular Mining Corporation Software. Hierarchical clustering will be performed using GeneCluster and Treeview software.

***Real Time Quantitative PCR***

Samples for PCR will be taken at screening and then post-challenge days 1, 5, 7, 9 and throughout the hotel phase (Day 10 through 20). Quantitative PCR is a method by which the number copies of *Plasmodium falciparum* in venous blood samples can be quantified and thus compared to blood smear data. Two mL blood samples will be collected in EDTA tubes at each time point. The blood sample will be passed through a Plasmodipur leukofilter in order to remove any human leukocytes. The sample will then undergo DNA extraction using a Qiagen miniprep kit (Qiagen, UK). One PCR reaction will consist of extracted DNA, primers and fluorescent probe specific for *Plasmodium falciparum* 18S ribosomal subunit mixed in PCR master mix. Standards of known *Plasmodium falciparum* concentrations are made at 10-fold dilutions and run on Light Cycler PCR machine to enable construction of a standard curve. Samples are run concurrently and thus the quantity of parasites in a unknown sample is calculated in reference to the standards.

***Whole Blood Assay (FastImmune)***

This assay is a modification of ICS in that no PBMC separation is required prior to detection of intracellular cytokine production.. Whole venous blood is collected from volunteers and 0.5 mL is combined with FMP2.1 antigen as well as CD28, CD49d antibodies and Brefeldin A. After 6 hour incubation, cells are fixed and permeabilized, then stained to distinguish by flow cytometry the CD4 and CD8 cells as well as intracellular production of IFN-gamma.

***Particle-based cytokine assay (Luminex):***

Serum cytokines can also be measured by particle-based flow cytometry assays which allow multiple analyses in one small sample. PBMC are isolated and cultured with FMP2.1. The supernatant is added to a microsphere solution in a 96-well format. Each microsphere has a capture antibody to detect a specific cytokine as well as a particular internal dye to allow its recognition by the flow cytometer. Once detector antibody to the cytokine of choice is added, fluorescence is measured by flow cytometry for cytokine quantification.

*Complement Protein and Regulatory Protein Levels:*

Samples *Plasmodium falciparum*-infected blood, as well as pre-infected blood, will be probed with fluorescent monoclonal antibodies specific for complement proteins (C3/C3b/iC3b/C3dg) and complement regulatory proteins (CD35/CD55) to determine specific protein levels. Briefly, aliquots of blood will be repeatedly washed with PBS-BSA buffer and resuspended to working concentrations (~10^5^-10^6^ cells per sample); the remaining blood will be stored at 4 ^o^C for approximately 3 weeks if repeated determinations are required. Post 3 weeks blood samples will be disposed of as biohazard waste. Fluorescent monoclonal antibodies will then be mixed with blood samples, and following incubation (30 min at room temperature) the samples will be washed repeatedly to remove excess antibody then fixed in 1% paraformaldehyde. Samples will be analyzed by fluorescent microscopy and by flow cytometry.

*Natural Killer Cell Assays:*

Peripheral Blood Mononuclear cells (PBMCs) will be separated by Ficoll gradient as previously described and cryopreserved in fetal calf serum/DMSO until needed for ex vivo evaluation. NK cells account for approximately 1% of the peripheral blood; therefore, NK cells will be enriched by negative selection using a magnetic bead kit from Miltenyi Biotech (Auburn, CA). Briefly, cells will be incubated with magnetic beads coated with antibodies directed against T-cells, B-cells, macrophages and dendritic cells. Cell suspensions will then be passed through a column under a magnetic field to isolate NK cells.

To evaluate IFNγ production, NK cells will be incubated in ELISpot plates in the presence of pRBCs (ratio 1:1) or uninfected RBCs. Additional assays will be performed in the presence of blocking antibody against NK cell receptors which bind HLA Class I molecules: killer cell immunoglobulin-like receptor (KIR) and C-type lectin-like receptors of the NK gene complex (NKC).

For ex-vivo evaluation of NK cell activation, cells will be stained with fluorescent labeled antibodies against extracellular pan-NK markers (PE-conjugated antibodies) and KIR (FITC conjugated antibodies). After cell permeabilization, cells will be stained intracellularly with APC-conjugated antibody to IFNγ. Expression of NK activation markers and IFNγ production will be measured by multicolor FACS analysis as a read out of activation.

*Appendix* ***E****: Vaccine Supplies, Packaging and Accountability*

It is under no circumstances permitted to use supplies for purposes other than those specified in the protocol. The sponsor will collect unused supplies on completion of the study. Used vaccine vials can be disposed on site according to local biosafety standards for disposal of biological waste material.

**1. Vaccine supplies**

The investigator will supply the following amounts of numbered study vaccines while GSK will supply the following amounts of adjuvant. The amount will be sufficient to administer 3 immunizations to all participants as described in the present protocol:

- 105 doses of the vaccine AMA-1 (15 in Cohort 1, 90 in Cohort 2: 45 Group A, 45 Group B)
- 79 doses of the adjuvant AS01B
- 45 doses of the adjuvant AS02A

An additional 3% (3 vials) of the respective amounts of AS01B and AS02A (each) will be supplied for replacement in case of breakage or bad storage conditions.

All vials/PFS need to be accounted for on the form provided.

**2. Vaccine packaging**

The vaccines will be packed in labeled boxes. The box label will contain the following information: study number, lot number and instructions for vaccine administration.

**3. Vaccine accountability**

The investigator or pharmacist must sign a statement that he/she has received the clinical supplies for the study. At any time the figures on supplied, used and remaining vaccines should match. At the end of the study, it must be possible to reconcile delivery records with those of used and returned stocks. Account must be given of any discrepancies.

Both used and unused vaccine vials should be destroyed at the study site using locally approved biosafety procedures and documentation unless otherwise described in the protocol.

Appendix F: Volunteer Agreement Affidavit (Cohort 1Vaccinees)

**____________________________________________________________________________________**

**VOLUNTEER AGREEMENT AFFIDAVIT**

For use of this form, see AR 70-25 or AR 40-38, the proponent agency is OTSG

**____________________________________________________________________________________**

PRIVACY ACT OF 1974

___________________________________________________________________________________________________________________________________________________________

**Authority:**  10 USC 3013, 44 USC 3101, and 10 USC 1071-1087

**Principal Purpose:**  To document voluntary participation in the Clinical Investigation and Research Program, home address and phone will be used for identification and location purposes.

**Routine Uses:** Home address will be used for identification and locating purposes. Information derived from the study will be used to document the study, implementation of medical programs, adjudication of claims, and for the mandatory reporting of medical conditions as required by law. Information may be furnished to Federal, State, and local agencies.

**Disclosure:** The furnishing of your home address is mandatory for participation and necessary to provide identification and to contact you if future information indicates that your health may be adversely affected. Failure to provide the information will preclude your voluntary participation in the investigational study.

**____________________________________________________________________________________________**

**Part A - VOLUNTEER AFFIDAVIT**

**__________________________________________________________________________________________________________________**

Volunteer Subjects in Approved Department of the Army Research Studies

____________________________________________________________________________________________________________________________________________________________

Volunteers under the provisions of AR 40-38 and AR 70-25 are authorized all necessary medical care for injury or disease which is the proximate result of their participation in such studies.

I, ____________________________________, having full capacity to consent and having attained my ________birthday, do hereby volunteer/give consent to participate in the protocol entitled

**“Phase I/IIa double-blind, randomized study of safety, immunogenicity, and preliminary efficacy after primary sporozoite challenge of FMP2.1/AS01B and FMP2.1/AS02A candidate malaria vaccines administered intramuscularly at months 0, 1, and 2 in healthy malaria-naïve adults living in the United States”**

under the direction of Dr. Michele D. Spring, Department of Immunology, conducted at Walter Reed Army Institute of Research (WRAIR), Silver Spring, MD 20910. The sponsor of this study is the Department of Defense.

**Dr. Michele Spring, Department of Immunology, WRAIR, (301) 319-9307**, or one of the assisting investigators has explained the:

- implications of my voluntary participation
- duration and purpose of the research study
- methods and means by which it is to be conducted; and
- inconveniences and hazards that may reasonably be expected

**I have been given an opportunity to ask questions concerning this investigational study. Any such questions were answered to my full and complete satisfaction. Should any further questions arise concerning my rights on study-related injury, I may contact The Office of Research Protections, US Army Medical Research and Materiel Command, Fort Detrick, MD 21702-5012; phone: (301) 619-2165.**

I understand that I may at any time during the course of this study revoke my consent and withdraw from the study without further penalty or loss of benefits; however, I may be required (military volunteer) or requested (civilian volunteer) to undergo certain examinations if, in the opinion of the attending physician, such examinations are necessary for my health and well-being. My refusal to participate will involve no penalty or loss of benefits to which I am otherwise entitled.

**_________________________________________________________________________________________PART B – TO BE COMPLETED BY INVESTIGATOR__________________**

____________________________________________________________________________________________________________________________________________________________

This research study is being conducted at the Walter Reed Army Institute of Research Clinical Trials Center by the following investigators: Dr. Michele Spring, Dr. James F. Cummings, Dr. D. Gray Heppner, Dr. Kent Kester, Dr. Christian Ockenhouse, Dr. Douglas Walsh.

Your participation in this study is voluntary. Before you decide whether or not to participate, we encourage you to read the information below. Please ask questions about anything you do not understand.

**PURPOSE OF THE STUDY**:

You are being asked to participate in a research study to test the safety of a new malaria vaccine.

Malaria is a disease that affects 300-500 million people throughout the world. Humans catch this disease by being bitten by mosquitoes that are infected with malaria parasites. Mosquitoes bite humans to feed on human blood. If not diagnosed and treated promptly, malaria can be quite serious and sometimes deadly. Much of the malaria in the world has become resistant to drugs used in the past, and a vaccine against this disease would help people living in, or traveling to areas where it is commonly found.

Our overall goal in this study is to compare two different malaria vaccines called FMP2.1/AS01B and FMP2.1/AS02A. Scientists at the Walter Reed Army Institute of Research and at GlaxoSmithKline Biologicals have been working on several malaria vaccines, which are designed to prevent malaria parasites from growing in people who received the vaccine. If malaria parasites were unable to grow in the human blood stream, then infected people would not become sick with the disease called malaria.

In this part of the study, our goal is to ensure that the malaria vaccine, FMP2.1/AS01B, is safe. Since this is the first time that FMP2.1/AS01B has been in humans, if you choose to enroll in this study, you will receive a very low dose (1/5^th^) of the full dose of this vaccine. You will receive this low dose vaccine of FMP2.1/AS01B every month for three months. We examine you and do blood tests in order to make sure the vaccine is safe. If it is safe, we will then go on to test a full dose in other volunteers.

The “FMP2.1” part is a protein which looks like one of many different malaria proteins, but does not actually cause malaria infections. The “AS01B” and “AS02A” parts are called adjuvants, and each is a different mixture of oil and water that help stimulate your body to fight infections. This vaccine does not contain the actual malaria parasite and therefore cannot actually cause malaria.

**INVESTIGATIONAL NEW DRUG:**

The investigational vaccines being tested in this study are not approved by the Food and Drug Administration (FDA) for commercial use; however, the FDA has permitted their use in this research study.

Prior human experience with these vaccines:

- FMP2.1/AS02A has been successfully given to 63 human volunteers without major side effects.
- This is the first time FMP2.1/AS01B will be given to humans.
  - However, other vaccines in combination with AS01B have been given to over 150 volunteers without any major side effects.
- Both vaccines have been made under clean and sterile conditions that are acceptable to the FDA. The FDA is allowing us to test these vaccines to see if they are safe, and to see they are able to protect you from malaria infections caused by mosquito bites.

SIMILAR STUDIES

The WRAIR Clinical Trials Center has 15-20 years experience in similar malaria vaccine clinical trials.

**NUMBER OF VOLUNTEERS:**

Five volunteers will participate in this part of the study evaluating the 1/5^th^ dose of FMP2.1/AS01B. A total of 36 volunteers will be in the second part of the study. This site will be the only site where this study is done.

**DURATION OF PARTICIPATION:**

The total duration of your participation in this study will be approximately 2 months (after screening is completed). During the immunization phase you will have approximately 20 appointments at the WRAIR Clinical Trials Center. Depending on the type of appointment, visits may take from 30 minutes to 2 hours. A more detailed schedule of visits is included for you at the end of this form.

PROCEDURES:

If you volunteer to be in this study, we would ask you to do the following:

1. General Description

- In this study, approximately 5 people will receive an investigational vaccine at 3 time points, each one month apart (0-, 1-, 2- month). The vaccine is made of 10µg of FMP2.1 in 0.5 mL of AS01B.

2. Consent Phase

- If you agree to participate in this study, you will be asked to sign 3 separate consent forms
  - In your 1st consent form, you will agree to participate in the study
  - In a 2nd consent form, you will give us permission to test your blood for HIV, the virus that causes AIDS
  - A 3rd consent form is optional, with which you may give us permission to keep your blood for future testing.
- In addition, there is a short multiple-choice quiz that tests your understanding of the study. A passing score of at least 80% correct is required. If you do not score 80% on your first try, we will review the study information with you to help you more fully understand it, and you will have the opportunity to take the test a second time and score at or above 80%.

**3. Screening Phase**

After you have signed the consent forms, you will start the “Screening Phase”. A physician and other medical staff will check to make certain that you are healthy. You will undergo an interview for a medical history and then have a physical examination. In order to participate in this study you must be a healthy adult who is available for the complete duration of the study (approximately 2 months not including screening time). A time line of the study dates will be provided to you.

- Specific things that could prevent you from being in the study:
  - Planned travel to an area with malaria
  - Previous history of malaria or malaria vaccine
  - Exposure to malaria within the past year or malaria medications within a month of planned vaccination
  - Allergy to any ingredients in the vaccine
  - Certain prescription medications
  - HIV or problems fighting infections, such as cancer or organ transplants
  - Severe problems with your heart, liver, lungs or kidneys
  - History of either sickle cell disease or sickle cell trait
  - Pregnancy (now, or planned within the study period), or breast-feeding
  - Chronic or active drug abuse
  - Chronic or active neurological disorder (i.e. problems with your brain or nervous system such as seizures, migraines, etc.)
- You will undergo a screening evaluation with a medical history and physical examination. In addition to questions about your health, you will be asked about your use of illegal substances and alcohol.
- You will also give blood and urine samples for routine laboratory tests. We will need to collect approximately 2 tablespoons of blood from you at this screening visit. Blood and urine samples for routine laboratory testing may be sent to either the Walter Reed Army Medical Center clinical laboratory or another contract clinical laboratory. The routine tests will include tests for HIV and Hepatitis B and C. If your tests show that you have the HIV or hepatitis B or C infection, you will be notified in a confidential manner and be given information on where you can receive medical treatment. Also, if you have HIV or hepatitis B or C infection, you will not be allowed to participate in this study. We will give you the results of our medical history, physical exam and lab tests so that you can give them your regular doctor.
- If any of your lab tests show that you are infected with HIV or any of the hepatitis viruses, we are required by law to report these tests results to local and state governmental health agencies. If you are a military service member, this information will be reported to the military preventive medicine service. In either case, additional information and/or interviews may be required for contact tracing.
- If you are female, we will test if you are pregnant with a urine test now and within 48 hours before each vaccination or challenge with the infected mosquitoes. Female volunteers with a positive pregnancy test will not be allowed to participate in the study.
- If you are not healthy or it is not safe for you to continue to participate in the study, you will be advised to seek medical care and/or counseling. At your request, we will inform your regular physician of any lab test results.

**4. Vaccination Phase**

If you are in good health, the screening laboratory tests are normal or negative, and there are no problems that prevent you from participating in the study, we will call you to inform you that you are eligible to be in the study. You can receive the results of your tests if you wish. We will tell you when to return to CTC to begin the “Vaccination Phase”. In this phase, you will receive 3 doses of one of the investigational vaccines: FMP2.1/AS01B at 0-, 1- and 2- months.

- The vaccine will be injected into the muscle of your upper arm using a needle and syringe. You will remain in the Clinical Trials Center for observation for 30 minutes after receiving the vaccine injection.
- After each vaccination, you will be seen again at approximately 24 hours, 48 hours (2 days), 72 hours (3 days), 7 days, and 14 days after each injection. At each visit we will ask you about possible side effects or other problems that you might be experiencing.
- We will give you diary cards and thermometers to help you track and list any symptoms that you may experience after the vaccine injection.

**BLOOD DRAWS**

We will be collecting blood samples from you at some of your clinic appointments. We will collect these blood samples on the day you receive the vaccine and also on some of the follow-up days. We will collect different amounts on different days. Some days we will only collect 7 milliliters of blood (about 1 teaspoonful). Other days we will need to collect as much as 157 milliliters (11 tablespoonfuls). These blood tests will help us check the health of your bone marrow, kidney and liver and to measure how well your immune system is responding to the vaccine.. We will not collect more than 550 milliliters (2 ¼ cups) of blood from you during any 8-week period of the study. We expect that your body will be able to make enough blood to replace this loss without harming you.

- We will give you a more detailed summary of the blood-drawing schedule during this screening session.
- In a separate consent document we will ask you to allow us to collect blood that may be used in malaria research studies and might be used to develop other products. Any blood that you provide will have your name and identifying information removed. (See Appendix J, Blood Donation Form)

PRECAUTIONS:

To be observed before and during the study:

1. You will receive a copy of this form. In addition, we will provide you with a list of appointment dates and times to come to the WRAIR Clinical Trials Department.
2. If you develop any medical problems that you think might have been caused by the vaccination, please call or come to the WRAIR Clinical Trials Department.
3. If you have any elective minor surgical or dental procedures planned for the 2 days prior to the vaccination date and during the 7 days after the vaccination dose, please reschedule those procedures for another time. This will help us to better evaluate if any of your medical problems are or are not caused by the vaccination.
4. For Women: We do not know what this investigational vaccine will do to an unborn baby. Therefore, it is very important that you read, understand and following these instructions to protect your unborn baby:
   - You should not be pregnant or become pregnant during your participation in this study. A small amount of your urine will be used to do a pregnancy test. This pregnancy test must be done within 2 days before each vaccination.
   - If a female volunteer has a positive pregnancy test, they will not be vaccinated.
   - You should avoid becoming pregnant if you want to continue your participation in this study. To avoid becoming pregnant, you should either abstain from sexual relations or practice a method of birth control. Except for surgical removal of the uterus, birth control methods such as the use of condoms, a diaphragm or cervical cap, birth control pills, IUD, or sperm killing products are not totally effective in preventing pregnancy.
   - You should not become pregnant beginning 1 month prior to the first vaccination through 2 months after the third vaccination.
   - Urine pregnancy tests are not able to pick up very early pregnancies. Therefore, if the pregnancy test is negative but you still think that you might be pregnant, you should not participate in the study. The only way that you can guarantee that you are not pregnant is to not have sex with a male person.
   - You should notify the investigator if it is determined after completion of the study that you became pregnant either during the study of within 30 days of the study.
   - If you become pregnant during your participation in the study, we will ask to be allowed to follow your pregnancy through its outcome. This will include information about both your health status and that of the child. The information will be reported confidentially (without names or any way of identifying you) to WRAIR (Walter Reed Army Institute of Research), USAMMDA (The U.S. Army Medical Materiel Development Activity), USAMRMC ORP (US Army Medical Research and Materiel Command, Office of Research Protections), MVI (Malaria Vaccine Initiative), GlaxoSmithKline Biologicals and the Food and Drug Administration (FDA).

POTENTIAL RISKS and DISCOMFORTS:

In order to prevent or minimize all possible risks and all possible hazards associated with this study, our medical team will observe you very closely and offer you U.S. standard medical care and treatment if you become sick with malaria or have any other medical problem related to this investigational vaccine. Our physicians will give you frequent physical examinations and conduct multiple laboratory tests as outlined above. A study physician will always be on call at one of the phone numbers listed below. Specific risk/discomfort information is outlined below:

1. General items:

- You must not receive any other investigational drugs or vaccines within the 30 days before you receive our study vaccine.
- We ask you not to participate in other medical research studies while you are in this study.

1. From the vaccine:

- As with all experimental products that are not yet approved by the FDA for general use, there are unknown risks to your health that might be caused by our experimental vaccine.
- Based on our previous experience with volunteers vaccinated with a similar vaccine, we expect that you may have some temporary local skin symptoms like pain, redness or swelling around the injection site, a mild headache and perhaps a low-grade fever with mild flu-like symptoms like tiredness and body aches. Less common reactions include nausea, vomiting, diarrhea and allergic reactions.
- Reactions which prevent normal, everyday activities have been seen in other malaria vaccines with the adjuvant used in this study; however, the reactions were generally short-lived, lasting less than 2 days.

1. From drawing your blood:

- You will have blood drawn by an experienced phlebotomist (person whose job is to draw blood). You may experience some discomfort and possibly also a bruise where blood is taken from your vein for blood tests. Some people get dizzy or feel faint when their blood is drawn. There is a very small chance that you could get an infection at the place where your blood is drawn.

1. From loss of confidentiality:

- If you participate in this study, there are also risks that information about you may be disclosed to persons outside of this study. This is discussed in the Confidentiality section of this document (below)

1. From future malaria infection:

- In the future, if you travel to a country where malaria is common, you cannot rely on this vaccine to protect against malaria. Thus it will still be necessary to take the standard precautions to prevent malaria.

UNKNOWN RISKS:

This investigational vaccine may cause unknown risks, to either you or to your unborn baby, which we are not able to predict; this is true for both men and women volunteers. Therefore, if you are a man, you should not get a woman pregnant while you are on this study; and if you are a woman, you should not get pregnant while you are on this study.

GENERAL BENEFITS: Volunteers will not benefit directly from participation in this research study. There may be benefit to humanity in the future related to this vaccine or study.

ALTERNATIVES TO PARTICIPATION: The alternative to participation is to refuse to be in this study.

DISCLOSURE OF RESEARCH RESULTS:

Your name will not be used in any report resulting from this study. We will not be able to disclose results of this study to any of the volunteers. However, we will give you a statement of your participation in this study, with the dates and any side effects you experienced. Also, if you request, we will give you a statement of your individual study results. You can then place these statements in your personal medical record or give them to your personal physician. If you prefer, we will mail these statements directly to your personal physician.

COMPENSATION: Payment will be based upon the amount of times we collect blood from you.

- Vaccination phase:
  - We will pay you $25 for screening blood work.
  - Payment during the “Vaccination Phase” will be $100 for each blood-drawing visit ($700.00 if all visits are kept).
- Active duty personnel will be paid $50 per blood draw, unless the visits occur during off duty hours or they are on leave. In this case, they will be paid the same as non-military personnel. All scheduled blood draws are planned to occur during off duty hours.
- Other than medical care that may be provided and any other payment specifically stated in this informed consent, there is no other compensation available for your participation in this research study; however, you should also understand that this is not a waiver or release of your legal rights.

COSTS:

There is no cost to you to participate in the study. Please note that we are not able to provide extra money to pay for your transportation to and from the study site or childcare, if applicable.

MEDICAL CARE FOR RESEARCH RELATED INJURY:

If you are hurt or get sick because of this research study, you can receive medical care at an Army hospital or clinic free of charge. You will only be treated for injuries that are directly caused by the research study. The Army will not pay for your transportation to and from the hospital or clinic. If you have questions about this medical care, talk to the principal investigator for this study, Dr. Michele D. Spring, 301-319-9307.

RESEARCH RELATED INJURY

If you pay out-of-pocket for medical care elsewhere for injuries caused by this research study, contact the principal investigator, Dr. Michele D. Spring, 301-319-9307. If the issue cannot be resolved, contact the U.S. Army Medical Research and Materiel Command (USAMRMC) Office of the Staff Judge Advocate (legal office) at (301) 619-7663.

CONFIDENTIALITY:

Records relating to your participation as a research volunteer will remain confidential as far as possible. However, there are some instances where we cannot guarantee complete confidentiality.

- We are required to report information regarding certain infectious diseases (like HIV or AIDS and hepatitis) to the local health department where you live. If your blood tests show that you have one of these infections, we will report this information to the health department, and they may need to interview you to get more information.
- For volunteers who are in the military, we are required to report the same kind of information to military medical authorities. As a result, this information may end up in your military medical record and may be reported to your chain of command.
- For military service members, if we become aware of offenses under the Uniform Code of Military Justice (UCMJ), we may report them to your chain of command. Depending on the response from your commanding officer, you may be subject to disciplinary or administrative action as a result.
- Representatives from the following agencies may have access to review research records as part of their responsibility to protect humans in research and oversee the quality of the research efforts. As government agencies, they must also maintain confidentiality of your records within the limits of the law.
  - U.S. Army Medical Research and Materiel Command (USAMRMC)
  - The sponsor of this study (USAMMDA)
  - The Human Use Review Committee (HURC) of WRAIR
  - The Food and Drug Administration (FDA)

VOLUNTARY PARTICIPATION:

Participation in this study is voluntary. Refusal to participate will involve no penalty or loss of benefits to which you are otherwise entitled. You may discontinue your participation at any time without penalty or loss of benefits.

WITHDRAWAL FROM PARTICIPATION:

Withdrawal from this study before it has been completed may have an effect on your health.

- If you choose to withdraw from the study during the vaccination phase, we will not be able to monitor your health for bad effects from the investigational vaccine. Therefore, if you have been vaccinated, we will request you complete an exit physical examination, and have standard safety laboratory tests drawn (blood count, liver, and kidney function) prior to your withdrawal from the study.
- Additionally, if you withdraw from the study, you may request for us to destroy your blood samples and not test them.

TERMINATION BY INVESTIGATOR WITHOUT YOUR CONSENT:

The principal investigator, Dr. Michele D. Spring, may decide not to allow you to continue participating in this study under the following conditions:

- If you develop health conditions that would make it dangerous to you or others if you continue participating
- If other situations or conditions arise that would make participation harmful to your own health,
- If you express plans or known illegal activities that may place you or anyone in the community in danger (we reserve the right to inform the local authorities of this).
- If you don’t follow the plan and instructions in this document.

SIGNIFICANT NEW FINDINGS:

We will tell you if we discover any significant or new information during the study that may affect your health and willingness to continue participation. When we tell you, we will also ask you to confirm your willingness to continue participation by signing another consent form like this which describes the significant or new information.

VOLUNTEERS’ RIGHTS:

If you have questions about your rights as a research volunteer in this study, you may contact the Human Subjects Research Review Board, U.S. Army Medical Research and Materiel Command (USAMRMC), 301-619-7802 or the Office of the Staff Judge Advocate (legal office), also at USAMRMC; 301-619-7663.

- The Army is sponsoring this study with some financial support from the Malaria Vaccine Initiative (MVI). The MVI is a focused vaccine development program under PATH (Program for Appropriate Technology in Health) and funds projects that test promising malaria vaccines in humans. The are located at 7500 Old Georgetown Road, 12^th^ Floor, Bethesda, MD 20814, tel: (240) 395-2700
- GlaxoSmithKline Biologicals (GSK BIO) is providing the adjuvant systems (AS01B and AS02A) and expert technical and monitoring assistance. This company has some offices in the U.S., but its main headquarters are in Europe. Therefore, they are required to use special wording in their research protocols. The European Union Data Protection Directive requires them to use this special wording. This wording is designed to ensure that you are aware of your rights. This wording is also designed to ensure that you are also clearly informed about how your personal data will be used and maintained confidentially, both during and after the research study is conducted.

1. Your personal data, including data relating to your health, will be recorded and processed for the purpose of assessing the outcome of the study. Processing will be done by WRAIR/MVI/GSK or may be contracted to a third party under strict confidentiality rules. Your data may also be processed for product registration and for notification to organizations monitoring the safety and effectiveness of medicines. Your data may also be processed in order to add to scientific knowledge;

2. Your participation in the study will be treated as confidential. You will not be referred to by name in any report concerning the study, nor will your identity be disclosed to any person, other than in circumstances such as where a need arises to check the correctness or completeness of data, or where there is a need to provide such information to regulatory agencies responsible for registration and safety of medicines.)

3. Your medical data may be sent to and processed by any affiliate of GSK in any country inside or outside the European Union, always respecting the requirements of the EU Data Protection Directive (95/46/EC) and/or the equivalent applicable law.

4. You may access your personal data and have any justifiable corrections made. If you wish to do so, you should request this from the doctor conducting the study. You agree to the postponement of your access to your medical data up to the completion of the study, including analysis and reporting of data, if deemed appropriate by the doctor conducting the study in order to safeguard the aim and conduct of the study. Representatives of WRAIR, USAMMDA, USAMRMC, MVI, GSK or regulatory bodies for medicines may access your records from this study.

VOLUNTEER REGISTRY

It is the policy of the U.S. Army Medical Research and Materiel Command (USAMRMC) that data sheets are to be completed on all volunteers participating in research for entry into this Command’s Volunteer Registry Data Base. The information to be entered into this confidential data base includes your name, address, Social Security number, study name and dates. The intent of the data base is two-fold: first, to readily answer questions concerning an individual’s participation in research sponsored by USAMRMC; and second, to ensure that the USAMRMC can exercise its obligation to ensure research volunteers are adequately warned (duty to warn) of risks and to provide new information as it becomes available. The information will be stored at USAMRMC for a minimum of 75 years.

**POINTS OF CONTACT: 24 hour contact (301) 319-9331**

Please contact one of the below if you have any questions concerning the medical research study or if you have any other questions or concerns:

**Clinical Trials Department, WRAIR** (301) 319-9660

Michele D. Spring, M.D.,(Principal Investigator) (301) 319-9307

James F. Cummings, M.D. (301) 573-0781

Kent E. Kester, M.D. (301) 319-9234

D. Gray Heppner, M.D. (301) 319-9414

Christian Ockenhouse, M.D., Ph.D. (301) 319-9473

Douglas S. Walsh, M.D. (301) 319-9828

COPY OF CONSENT FORM

We will give you a copy of this consent form after you sign it.

I do______ do not______ (check one & initial) wish to have a second copy of my consent form provided to me so that I may place it in my medical record.

**IF THERE IS ANY PORTION OF THIS CONSENT EXPLANATION SHEET THAT YOU DO NOT UNDERSTAND, PLEASE ASK THE INVESTIGATOR BEFORE SIGNING.**

Satisfactory performance on comprehension test (Yes/No):________ (investigator initials)

____________________________________________________________________

SIGNATURE OF VOLUNTEER DATE

____________________________________________________________________

NAME OF VOLUNTEER PRINTED

____________________________________________________________________

PERMANENT ADDRESS OF VOLUNTEER (including city, state and zip) PRINTED

____________________________________________________________________

INVESTIGATOR’S SIGNATURE DATE

INVESTIGATOR’S NAME (PRINTED) DATE

**Volunteer Schedule – Immunization Phase for Cohort 1 (1/5^th^ dose) volunteers**

| **Visit#** | **Study Day** | **Scheduled Visit  Date** | **Time Allotment**  **(approximate)** | **Activity** | **Compensation** |
| --- | --- | --- | --- | --- | --- |
| 1 | Screen | 90 to 3 days prior to start | 2 hours | Study explanation, informed consent process, blood draw for lab work, history/physical | $25.00 |
| 2 | Screen #2 | 90 to 3 days prior to start | 30 minutes | Completion of screening ( blood draw for lab work) | $100.00 |
| **3** | **0** |  | **Immunization # 1,**  **1 to 1.5 hours** | **Brief history/physical, vital signs, blood draw. women: urine pregnancy test  *(if your pregnancy test is positive, you will not be immunized).***  **Immunization, followed by 30 min. evaluation.**  **Receive diary card, thermometer and emergency contact card.** | $100.00 |
| 4-6 | 1,2 and 3 |  | 30 minutes | See physician for interim history, including any medications taken.  Bring diary for review. | *none* |
| 7 | 7 |  | 30 minutes | See physician for interim history, including any medications taken.  Return diary. | *none* |
| 8 | 14 |  | 30 minutes | See physician for interim history, including any medications taken.  Blood draw | $100.00 |
| **9** | **28** |  | **Immunization # 2,**  **1 to 1.5 hours** | **Brief history/physical, vital signs, blood draw. Women: urine pregnancy test  *(if your pregnancy test is positive, you will not be immunized).***  **Immunization, followed by 30 min. evaluation. Receive diary card.** | $100.00 |
| 10-12 | 29, 30 and 31 |  | 30 minutes each day | See physician for interim history, including any medications taken.  Bring diary for review. | *none* |
| 13 | 35 |  | 30 minutes | See physician for interim history, including any medications taken.  Return diary. | *none* |
| 14 | 42 |  | 30 minutes | See physician for interim history, including any medications taken.  Blood draw | $100.00 |
| **15** | **56** |  | **Immunization # 3,**  **1 to 1.5 hours** | **Brief history/physical, vital signs, blood draw. women: urine pregnancy test  *(if your pregnancy test is positive, you will not be immunized).***  **Immunization, followed by 30 min. evaluation. Receive diary.** | $100.00 |
| 16-18 | 57, 58 and 59 |  | 30 minutes each day | See physician for interim history, including any medications taken.  Bring diary for review. Blood draw on D57 & 59 | *none* |
| 19 | 63 |  | 30 minutes | See physician for interim history, including any medications taken.  Return diary. | *none* |
| 20 | 70 |  | 30 minutes | See physician for interim history, including any medications taken.  Blood draw | $100.00 |

*If, after beginning the study, you are unable to keep an appointment, or have any questions, please call 301-319-9660.*

Appendix G: Volunteer Agreement Affidavit (Cohort 2 Vaccinees)

**____________________________________________________________________________________**

**VOLUNTEER AGREEMENT AFFIDAVIT**

For use of this form, see AR 70-25 or AR 40-38, the proponent agency is OTSG

**____________________________________________________________________________________**

PRIVACY ACT OF 1974

___________________________________________________________________________________________________________________________________________________________

**Authority:**  10 USC 3013, 44 USC 3101, and 10 USC 1071-1087

**Principal Purpose:**  To document voluntary participation in the Clinical Investigation and Research Program, home address and phone will be used for identification and location purposes.

**Routine Uses:** Home address will be used for identification and locating purposes. Information derived from the study will be used to document the study, implementation of medical programs, adjudication of claims, and for the mandatory reporting of medical conditions as required by law. Information may be furnished to Federal, State, and local agencies.

**Disclosure:** The furnishing of your home address is mandatory for participation and necessary to provide identification and to contact you if future information indicates that your health may be adversely affected. Failure to provide the information will preclude your voluntary participation in the investigational study.

**____________________________________________________________________________________________**

**Part A - VOLUNTEER AFFIDAVIT**

**__________________________________________________________________________________________________________________**

Volunteer Subjects in Approved Department of the Army Research Studies

____________________________________________________________________________________________________________________________________________________________

Volunteers under the provisions of AR 40-38 and AR 70-25 are authorized all necessary medical care for injury or disease which is the proximate result of their participation in such studies.

I, ____________________________________, having full capacity to consent and having attained my ________birthday, do hereby volunteer/give consent to participate in the protocol entitled

**“Phase I/IIa double-blind, randomized study of safety, immunogenicity, and preliminary efficacy after primary sporozoite challenge of FMP2.1/AS01B and FMP2.1/AS02A candidate malaria vaccines administered intramuscularly at months 0, 1, and 2 in healthy malaria-naïve adults living in the United States”**

under the direction of Dr. Michele D. Spring, Department of Immunology, conducted at Walter Reed Army Institute of Research (WRAIR), Silver Spring, MD 20910. The sponsor of this study is the Department of Defense.

**Dr. Michele Spring, Department of Immunology, WRAIR, (301) 319-9307**, or one of the assisting investigators has explained the:

- implications of my voluntary participation
- duration and purpose of the research study
- methods and means by which it is to be conducted; and
- inconveniences and hazards that may reasonably be expected

**I have been given an opportunity to ask questions concerning this investigational study. Any such questions were answered to my full and complete satisfaction. Should any further questions arise concerning my rights on study-related injury, I may contact The Office of Research Protections, US Army Medical Research and Materiel Command, Fort Detrick, MD 21702-5012; phone: (301) 619-2165.**

I understand that I may at any time during the course of this study revoke my consent and withdraw from the study without further penalty or loss of benefits; however, I may be required (military volunteer) or requested (civilian volunteer) to undergo certain examinations if, in the opinion of the attending physician, such examinations are necessary for my health and well-being. My refusal to participate will involve no penalty or loss of benefits to which I am otherwise entitled.

**_________________________________________________________________________________________PART B – TO BE COMPLETED BY INVESTIGATOR__________________**

____________________________________________________________________________________________________________________________________________________________

This research study is being conducted at the Walter Reed Army Institute of Research Clinical Trials Center by the following investigators: Dr. Michele Spring, Dr. James F. Cummings, Dr. D. Gray Heppner, Dr. Kent Kester, Dr. Christian Ockenhouse, Dr. Douglas Walsh.

Your participation in this study is voluntary. Before you decide whether or not to participate, we encourage you to read the information below. Please ask questions about anything you do not understand.

**PURPOSE OF THE STUDY**:

You are being asked to participate in a research study to test the safety of a new malaria vaccine and to see if the vaccine can stimulate your immune system to prevent malaria infection.

Malaria is a disease that affects 300-500 million people throughout the world. Humans catch this disease by being bitten by mosquitoes that are infected with malaria parasites. Mosquitoes bite humans to feed on human blood. If not diagnosed and treated promptly, malaria can be quite serious and sometimes deadly. Much of the malaria in the world has become resistant to drugs used in the past, and a vaccine against this disease would be beneficial to people living in, or traveling to areas where it is commonly found.

Our goal in this study is to compare two different malaria vaccines called FMP2.1/AS01B and FMP2.1/AS02A. Scientists at the Walter Reed Army Institute of Research and at GlaxoSmithKline Biologicals have been working on several malaria vaccines, which are designed to prevent malaria parasites from growing in people who have received the vaccine. If malaria parasites were unable to grow in the human blood stream, then infected people would not become sick with the disease called malaria.

In this study we will compare 2 different malaria vaccines: FMP2.1/AS01B and FMP2.1/AS02A. The “FMP2.1” part is a protein which looks like one of many different malaria proteins, but does not actually cause malaria infections. The “AS01B” and “AS02A” parts are called adjuvants, and each is a different mixture of oil and water that help stimulate your body to fight infections. This vaccine does not contain the actual malaria parasite and therefore cannot actually cause malaria.

In this study we will compare the different malaria vaccines in 2 groups of volunteers. One group (Group A) will receive the vaccine called “FMP2.1/AS01B” and the other group (Group B) will receive the vaccine “FMP2.1/AS02A”. Currently, there is not a blood test to see whether or not the vaccine will work or protect against malaria (such as for hepatitis). Therefore, to see if this vaccine can protect volunteers in this study against malaria, it is necessary to let them be bitten by malaria-infected mosquitoes. This is called a malaria challenge. If volunteers don’t develop malaria, then we can conclude that the vaccine worked, and we can also see if one vaccine was better at protecting than another. For this study, only a portion of the vaccinated volunteers will go on to malaria challenge.

**INVESTIGATIONAL NEW DRUG:**

The investigational vaccines being tested in this study are not approved by the Food and Drug Administration (FDA) for commercial use; however, the FDA has permitted their use in this research study.

Prior human experience with these vaccines:

- FMP2.1/AS02A has been successfully given to 63 human volunteers without major side effects.
- This is the first study in which FMP2.1/AS01B will be given to humans.
  - As part of this study, a very low dose (1/5^th^ full dose) of this vaccine will be given to 5 volunteers. We will evaluate those 5 volunteers and only schedule you to receive the full dose if we find the low dose is totally safe.
  - However, other vaccines in combination with AS01B have been given to over 150 volunteers without any major side effects.
- Both vaccines have been made under clean and sterile conditions that are acceptable to the FDA. The FDA is allowing us to test these vaccines to see if they are safe, and to see they are able to protect you from malaria infections caused by mosquito bites.

SIMILAR STUDIES

The WRAIR Clinical Trials Center has vast experience in similar malaria vaccine clinical trials. Over the past 15 – 20 years, we have let mosquito-infected mosquitoes bite approximately 500 volunteers, and all volunteers recovered from their malaria infections. No volunteers who got sick with malaria ever required hospital admission to treat their infections, and no volunteers ever died of malaria.

**NUMBER OF VOLUNTEERS:**

Five volunteers have already been selected and have begun participation in this study. They received the low dose (1/5^th^ the full dose) of the vaccine FMP2.1/AS01B. Approximately 36 new volunteers will participate in this part of the study. This site will be the only site where this study is done.

**DURATION OF PARTICIPATION:**

The total duration of your participation in this study will be approximately up to 8 months (this includes 3 months time for screening volunteers). During the immunization phase you will have approximately 20 appointments at the WRAIR Clinical Trials Center. If you are challenged with malaria-infected mosquitoes, you will spend approximately 10 nights in a nearby hotel as well as have 10 more appointments at the Clinical Trials Center over a period of 3 months. Depending on the type of appointment, visits may take from 30 minutes to 2 hours. A more detailed schedule of visits is included for you at the end of this form.

PROCEDURES:

If you volunteer to be in this study, we would ask you to do the following:

1. General Description

- In this study, approximately up to 30 people will receive an investigational vaccine at 3 time points, each one month apart (0-, 1-, 2- month).
  - Group A (up to 15 volunteers) will receive 50µg of FMP2.1 in 0.5 mL of AS01B
  - Group B (up to 15 volunteers) will receive 50µg of FMP2.1 in 0.5 mL of AS02A)
- Assignment to Group A or B will be randomly decided (like a coin toss), and which vaccine you get will not be known by you or the investigators. After receiving three immunizations, 10 of the 15 volunteers from Group A and 10 of 15 from Group B will be randomly selected to be challenged with malaria to test if the vaccine works. Only volunteers who have completed all visits and vaccinations can be eligible to go on to challenge. If more than 10 volunteers from a group are willing and eligible to go to challenge, we will have to randomly choose (from a computer generated list) who will go to challenge. You can also choose not to be involved in the malaria challenge. We will ask you if wish to do participate at the Day 3 visit after the third immunization (Day 59 of study). A minimum for 6 volunteers from each group must agree and be available to go on to challenge in order for the challenge to occur.
- If you are one of the 10 volunteers from Group A or one of the 10 volunteers from Group B that is selected to undergo malaria challenge, you will allow malaria-infected mosquitoes to bite you. YOU MAY DEVELOP MALARIA FROM THE MOSQUITO BITE.
- A new group of 6 volunteers who did NOT receive the vaccines will also be enrolled to be challenged with malaria to provide a comparison group.
- Mosquitoes that are infected with malaria will be placed on the arms of the volunteers at about 2 to 4 weeks after the third vaccination. All volunteers who undergo challenge will be closely observed to see if they become sick with malaria. If any volunteers become sick with malaria, a physician will treat them with FDA approved drugs.

2. Consent Phase

- If you agree to participate in this study, you will be asked to sign 3 separate consent forms
  - In your 1st consent form, you will agree to participate in the study
  - In a 2nd consent form, you will give us permission to test your blood for HIV, the virus that causes AIDS
  - A 3rd consent form is optional, with which you may give us permission to keep your blood for future testing.
- In addition, there is a short multiple-choice quiz that assesses your understanding of the study. A passing score of at least 80% correct is required. If you do not score 80% on the initial quiz, we will review the study information with you to help you more fully understand it, and you will have the opportunity to take the test a second time and score at or above 80%.

**3. Screening Phase**

After you have signed the consent forms, you will start the “Screening Phase”. A physician and other medical staff will check to make certain that you are healthy. You will undergo a screening evaluation and undergo a medical history and physical examination. In order to participate in this study you must be a healthy adult who is available for the complete duration of the study (approximately 5 months with vaccination and challenge, not including screening time). A time line of the study dates will be provided to you.

- Specific things that could prevent you from being in this study:
  - Planned travel to an area with malaria
  - Previous history of malaria or malaria vaccine
  - Exposure to malaria within the past year or malaria medications within a month of planned vaccination
  - Allergy to any ingredients of the vaccine
  - Certain prescription medications
  - HIV or problems fighting infections, such as cancer or transplants
  - Severe problems with your heart, liver, lungs or kidneys
  - History of either sickle cell disease or sickle cell trait
  - Pregnancy (now, or planned within the study period), or breast-feeding
  - Chronic or active drug abuse
  - History of severe allergic reaction to mosquito bites
  - Chronic or active neurological disorders (i.e. problems with your brain or nervous system such as seizures, migraines, etc.)
- You will undergo a screening evaluation with a medical history and physical examination. In addition to questions about your health, you will be asked about your use of illegal substances and alcohol.
- You will also give blood and urine samples for routine laboratory tests. We will need to collect approximately 2 tablespoons of blood from you at this screening visit. Blood and urine samples for routine laboratory testing may be sent to either the Walter Reed Army Medical Center clinical laboratory or another contract clinical laboratory. The routine tests will include tests for HIV and hepatitis B and C. If your tests show that you have the HIV or hepatitis B or C infection, you will be notified in a confidential manner and be given information on where you can receive medical treatment. You will not be allowed to participate in this study if you are found to have HIV or hepatitis B or C infection. We will give you the results of our medical history, physical exam and lab tests so that you can give them your regular doctor.
- If any of your lab tests show that you are infected with HIV or any of the hepatitis viruses, we are required by law to report these tests results to local and state governmental health agencies. If you are a military service member, this information will be reported to the military preventive medicine service. In either case, additional information and/or interviews may be required for contact tracing.
- If you are female, we will test if you are pregnant with a urine test now and within 48 hours before a vaccination or challenge with the infected mosquitoes. Female volunteers with a positive pregnancy test will not be allowed to participate in the study.
- If you are not healthy or it would not be safe for you to continue to participate in the study, you will be advised to seek medical care and/or counseling. At your request, we will inform your regular physician of any lab test results.

**4. Vaccination Phase**

If you are in good health, the screening laboratory tests are normal or negative, and there are no problems that prevent you from participating in the study, we will call you to inform you that you are eligible to be in the study. You can receive the results of your tests if you wish. We will tell you when to return to CTC to begin the “Vaccination Phase”. In this phase, you will receive 3 doses of one of the investigational vaccines: FMP2.1/AS01B or FMP2.1/AS02A at 0-, 1- and 2- months.

- The vaccine will be injected into the muscle of your upper arm using a needle and syringe. You will remain in the Clinical Trials Center for observation for 30 minutes after receiving the vaccine injection.
- After each vaccination, you will be seen again at approximately 24 hours, 48 hours (2 days), 72 hours (3 days), 7 days, and 14 days after each injection. At each visit we will ask you about possible side effects or other problems that you might be experiencing.
- We will give you diary cards and thermometers to help you track and list any symptoms that you may experience after the vaccine injection.

5. Challenge Phase:

After the “Vaccination Phase”, up to 10 volunteers from Group A and up to 10 from Group B who received all 3 doses of vaccine, and approximately 6 more volunteers who have not received any vaccine, will be invited to participate in the ”Challenge Phase”. The “Challenge Phase” will help us to determine if this vaccine works. The “Challenge Phase” will begin approximately 14 to 28 days after the “Vaccination Phase’.

- If you are female, we will perform a urine pregnancy test 48 hours before the malaria-infected mosquitoes bite you. If your pregnancy test is positive, we will not allow you to be bitten by the infected mosquitoes.
- We will let a small number of malaria-infected mosquitoes bite you. Initially, we allow 5 mosquitoes to bite you for a period of five minutes. If all 5 mosquitoes do not bite you, we will let more mosquitoes bite for you five minutes, until a total of 5 malaria-infected mosquitoes have bitten you.
- In the days after the infected mosquitoes have bitten you, we will check you for signs of malaria infection. Depending upon how well the vaccine works, YOU MAY DEVELOP MALARIA FROM THE MOSQUITO BITE.
- Malaria usually develops between 9 and 15 days after an infected mosquito bites you. Starting 5 days and for up to 30 days after the mosquitoes have bitten you, we will collect approximately 1 teaspoonful of blood from your vein once daily (or more often if you develop fever or other problems). We will look at your blood under a microscope and try to see malaria parasites in your blood. These samples will be saved for later re-examination and confirmation of disease.
- To describe the daily visits more fully:
- We will see you in the Clinical Trials Center, WRAIR on days 1 and 5 – 9, which will be a Monday through Friday schedule.
- Days 10 – 20, we will move the clinical team and volunteers into a local hotel, in order to be available at any time you may need us. We will see you every day, including weekends. The hotel phase is described more fully shortly.
- After the hotel phase, if you have not been diagnosed with malaria, you will come in to the Clinical Trials Center, WRAIR daily during the work week, but not weekends or holidays. However, we ask that you have a way to contact the study investigators at any time, should you need to.
- Symptoms of malaria may include fever, headache, joint aches, generalized body aches, shaking chills, nausea and/or diarrhea. FDA approved medications for the symptoms will be available for doctors to give to you as needed. These include, but aren’t limited to: Tylenol™ (acetaminophen) and Motrin™ (ibuprophen) for pain or fever, Tigan™ for nausea, and Imodium™ for diarrhea. The doctors will be sure to check that you aren’t allergic to the medicines before using them. An alternative medicine will be available if you are allergic to the prescribed medicine.
- Malaria symptoms may develop either before or after the parasites are seen in your blood test. Approximately 1/3 of the time, specialists are able to detect the parasites before you feel symptoms.
- If we see malaria parasites in your blood, we will treat your infection with an FDA approved drug that treats malaria. This medicine is called chloroquine and is a pill taken by mouth over three days. You will be seen every day during the treatment course, until we see no malaria parasites in your blood for 3 days in a row. From previous experience, we expect that the malaria parasites will disappear from your blood within two or three days after we treat you with chloroquine. People usually do not have any problems with this medicine, but if it causes side effects that require you to stop taking it, or if it doesn't get rid of the malaria parasites, other effective drugs are available and will be used (such as Malarone™ or doxycycline and quinine). Common side effects of chloroquine and other anti-malaria drugs may include headache, dizziness, visual disturbances, loss of appetite, nausea, vomiting, abdominal cramps, ringing in the ears, or a rash.
- After your blood has had no parasites for 3 days in a row, we will continue to see you for follow-up examinations once a week for four weeks. During these visits, you will be examined and asked about symptoms of malaria because there is a very small (less than 1%) theoretical chance that your malaria infection could back and make you ill. If you get sick again with malaria (this is called a recurrence), we will give you a different medicine to treat the infection. After this recurrence is cured, we will continue to follow you once a week for another four weeks. In our history of malaria vaccine development at the WRAIR, no volunteers have ever had a malaria recurrence.

6. Hotel Phase:

A group of hotel rooms in the local area will be reserved for malaria challenged volunteers. All volunteers who have gone through the challenge are required to spend every night at the hotel for approximately 10 nights, beginning on the night of ‘Day 9’ after the mosquitoes bite you. During the day you can leave the hotel to go to work or school or do whatever you would like to do as long as you feel up to it.

- You will have a daily study visit in the hotel. This means you must check in with the study staff in the hotel each morning. At that visit, we will ask you how you are feeling and draw your blood to check for parasites. In the afternoon, we will establish contact with you to check on how you are feeling, and tell you if you have parasites in your blood and thus need to be treated.
- Staying in the hotel at night allows the physicians and medical staff to evaluate you very quickly and easily at night. At least one physician will be on-duty and present in the hotel every night. The on-duty physician will check your blood test results and treat you if you develop malaria.
- If you do not develop malaria symptoms and we do not see malaria parasites in your blood within 30 days after the mosquitoes bite you, this means that the vaccine worked and the malaria parasites could not cause a malaria infection in you. We will, however, continue to follow you on a weekly basis for four additional weeks to make sure no other symptoms occur.
- The longest you would have to stay in the hotel is ten nights. If you develop malaria and have finished the entire treatment, you will not need to remain in the hotel at night. Only a study investigator may release you from the daily visits.
- WRAIR will pay for the hotel rooms. You will need to pay for extra expenses such as long-distance phone calls, meals and movies that you may want.

**BLOOD DRAWS**

We will be collecting blood samples from you at some of your clinic appointments and also when you are in the hotel. We will collect these blood samples on the day you receive the vaccine and also on some of the follow-up days. We will collect different amounts on different days. Some days we will only collect 2 milliliters of blood (about ½ teaspoonful). Other days we will need to collect as much as 160 milliliters (11 tablespoonfuls). These blood tests will help us check the health of your bone marrow, kidney and liver and to measure how well your body is fighting the malaria infection. We will not collect more than 550 milliliters (2 ¼ cups) of blood from you during any 8-week period of the study. We expect that your body will be able to make enough blood to replace this loss without harming you.

- We will give you a more detailed summary of the blood-drawing schedule during this screening session.
- In a separate consent document we will ask you to allow us to collect blood that may be used in malaria research studies and might be used to develop other products. Any blood that you provide will have your name and identifying information removed. (See Appendix J, Blood Donation Form)

PRECAUTIONS:

Both Groups A and B - to be observed before and during the study:

- You will receive a copy of this form. In addition, we will provide you with a list of appointment dates and times to come to the WRAIR Clinical Trials Department.
- If you develop any medical problems that you think might have been caused by the vaccination, please call or come to the WRAIR Clinical Trials Department.
- If you have any elective minor surgical or dental procedures planned for the 2 days prior to the vaccination date and during the 7 days after the vaccination dose, please reschedule those procedures for another time. This will help us to better evaluate if any of your medical problems are or are not caused by the vaccination.
- For Women: We do not know what this investigational vaccine will do to an unborn baby. Therefore, it is very important that you read, understand and following these instructions to protect your unborn baby:
  - - You should not be pregnant or become pregnant during your participation in this study. A small amount of your urine will be used to do a pregnancy test. This pregnancy test must be done within 2 days before vaccination and again 2 days before the scheduled mosquito bites. A final pregnancy test will be done at the conclusion of the study.
    - If a female volunteer has a positive pregnancy test, they will not be vaccinated and they will not be bitten with malaria-infected mosquitoes.
    - You should avoid becoming pregnant if you want to continue to participate in this study. To avoid becoming pregnant, you should either abstain from sexual relations or practice a method of birth control. Except for surgical removal of the uterus, birth control methods such as the use of condoms, a diaphragm or cervical cap, birth control pills, IUD, or sperm killing products are not totally effective in preventing pregnancy.
    - You should not become pregnant beginning 1 month prior to the first vaccination through 2 months after the third vaccination or 2 months after the malaria-infected mosquitoes bite you, whichever is later. The malaria infection within the 2-month period after the pregnancy may create a potential risk to your unborn baby.
    - Urine pregnancy tests are not able to pick up very early pregnancies. Therefore, if the pregnancy test is negative but you still think that you might be pregnant, you should not participate in the study. The only way that you can guarantee that you are not pregnant is to not have sex with a male person.
    - You should notify the investigator if you find out after the study that you became pregnant either during the study of within 30 days of the study.
    - If you become pregnant while you are participating in the study, we will ask to be allowed to follow your pregnancy through its outcome. This will include information about both your health status and that of the child. The information will be reported confidentially (without names or identifying criteria) to WRAIR (Walter Reed Army Institute of Research), USAMMDA (The U.S. Army Medical Materiel Development Activity), USAMRMC ORP (US Army Medical Research and Materiel Command, Office of Research Protections), MVI (Malaria Vaccine Initiative), GlaxoSmithKline Biologicals and the Food and Drug Administration (FDA).

Precautions for Volunteer undergoing malaria challenge:

1. Some antibiotics (medicines that fight infection) prevent the malaria parasite from growing. Therefore, you should not take any antibiotics, unless it is absolutely necessary, beginning 2 weeks before each vaccination date and for at least 2 months after the malaria-infected mosquitoes bite you. If your personal physician prescribes an antibiotic for you, you should inform one of the study physicians, preferably before you begin to take it. The study physician will then have a chance to talk with your personal physician about possibly using a different antibiotic if necessary, which would not interfere with the study. If your physician agrees to use the antibiotic that we recommended, we will provide this antibiotic to you at no cost.

2. Mosquitoes may not try to bite you and feed on your blood if you have used cologne, deodorant soap or lotion on your skin on the day of the bite. Therefore, please do not use such items on your skin on the challenge day.

POTENTIAL RISKS and DISCOMFORTS:

In order to prevent or minimize all possible risks and all possible hazards associated with this study, our medical team will observe you very closely and offer you U.S. standard medical care and treatment if you become sick with malaria or have any other medical problem related to this investigational vaccine. Our physicians will give you frequent physical examinations and conduct multiple laboratory tests as outlined above. A study physician will always be on call at one of the phone numbers listed below. Specific risk/discomfort information is outlined below:

General items:

- You must not receive any other investigational drugs or vaccines within the 30 days before you receive our study vaccine.
- We ask that you not participate in other medical research studies while you are in this study.

General items (for malaria challenged volunteers):

- It is very important for you to take all the malaria treatment medicines that we give you; otherwise you are more likely have the malaria infection recur. This is an important risk for you to understand. However, in our nearly 20 years of doing studies like this, we have never had a repeat infection of this type.
- If you plan to travel outside the Washington metropolitan area on vacation or leave, even for one day, during the year after this study, discuss your plans with one of the physician investigators listed below so that they can advise you about additional precautions. We will give you a special card that identifies you as a participant in this clinical trial. The card will have detailed instructions for contacting a study physician 24 hours a day.
- In the future, should you travel to a country where malaria is common, it will be necessary to take standard precautions to prevent malaria, regardless of whether you were, or were not, protected by the vaccine in the challenge.

From the vaccine:

- As with all experimental products that are not yet approved by the FDA for general use, there are unknown risks to your health that might be caused by our experimental vaccine.
- Based on our previous experience with volunteers vaccinated with a similar vaccine, we expect that you may have some temporary local skin symptoms like pain, redness or swelling around the injection site, a mild headache and perhaps a low-grade fever with mild flu-like symptoms like tiredness and body aches. Less common reactions include nausea, vomiting, diarrhea and allergic reactions.
- Reactions which prevent normal, everyday activities have been seen in other malaria vaccines with the adjuvant used in this study; however, the reactions were generally short-lived, lasting less than 2 days.

From malaria (malaria challenged volunteers):

- You may get sick with malaria after the malaria-infected mosquitoes bite you. However, under the very carefully controlled conditions of this study, the chance of serious illness or death from malaria infection is very small.
- If you get sick with malaria, you will commonly develop a combination of the following symptoms: fever, headache, joint pain, generalized body aches, shaking chills, nausea (become sick to your stomach), vomiting, diarrhea, or feel pretty sick all over. You might also develop mild problems with your oxygen carrying and infection fighting blood cells, tenderness over your liver and feel fatigued. If untreated,, malaria infections damage your kidney, liver or brain, and can even kill you.
- Therefore, if you get sick with malaria, we will treat you at no cost and give you FDA approved anti-malarial drugs to treat the infection.
- There is a chance if you get sick with malaria, and if we treat you and you recover, that you may get sick again with malaria, although this has not occurred in any previous malaria vaccine studies at WRAIR. You could get sick with malaria a second time at any time up to 1 year after you get malaria the 1st time. If during that 1 year after you participate in this study and you develop symptoms that might be caused by malaria, please contact one of the physicians listed below or tell your personal physician that you had participated in this study. Malaria symptoms can include fever, headache, muscle pains, nausea, or vomiting.
- The policy of the American Association of Blood Banks says that you will not be permitted to donate blood for three years after your treatment for malaria. During that period of time, no blood bank will accept you as a blood donor. You may or may not be able to donate blood for your own use ("auto-donation"), depending on the policies of the blood donor center or hospital where you are receiving care.
- Malaria is a serious infection and untreated, can lead to death. That is why it is so important to take all of the medications and follow the physician investigators instructions.

From mosquito bites (malaria challenged volunteers):

- At the sites of the mosquito bites, you might develop redness, mild swelling and itching.
- There is also a very small theoretical risk that the malaria-infected mosquitoes could give you a disease such as hepatitis or AIDS. A mosquito would have to feed on an HIV or hepatitis-infected human, and then be allowed to bite you. We do 3 specific procedures to minimize this theoretical risk:
  1. The blood for feeding the mosquitoes is obtained from an FDA-certified blood bank. People who are at risk for having infections such as HIV and hepatitis are not allowed to donate there;

b. The blood that is used to feed mosquitoes is tested by the FDA-certified blood bank to make sure that there are no Hepatitis or HIV viruses in the blood; and

c. All mosquitoes that bite you have not been allowed to feed on other human blood for at least 16 to 18 days before they bite you. This guarantees that the mosquito kills any viruses that they might have collected while feeding on human blood before the mosquito bites you.

- Specifically, there has been no reported transmission of the AIDS virus using the 3-part mosquito safeguard system described above.

From drawing your blood:

You will have blood drawn by an experienced phlebotomist (person whose job is to draw blood). You may experience some discomfort and possibly also a bruise where blood is taken from your vein for blood tests. Some people get dizzy or feel faint when their blood is drawn. There is a very small chance that you could get an infection at the place where your blood is drawn.

From loss of confidentiality:

- If you participate in this study, there are also risks that information about you may be disclosed to persons outside of this study. This is discussed in the Confidentiality section of this document (below)

UNKNOWN RISKS:

This investigational vaccine may cause unknown risks, to either you or to your unborn baby, which we are not able to predict; this is true for both men and women volunteers. Therefore, if you are a man, you should not get a woman pregnant while you are on this study; and if you are a woman, you should not get pregnant while you are on this study.

GENERAL BENEFITS: Volunteers will not benefit directly from participation in this research study. There may be benefit to humanity in the future related to this vaccine or study.

ALTERNATIVES TO PARTICIPATION: The alternative to participation is to refuse to be in this study.

DISCLOSURE OF RESEARCH RESULTS:

Your name will not be used in any report resulting from this study. We will not be able to disclose results of this study to any of the volunteers. However, we will give you a statement of your participation in this study, with the dates and any side effects you experienced. Also, if you request, we will give you a statement of your individual study results. You can then place these statements in your personal medical record or give them to your personal physician. If you prefer, we will mail these statements directly to your personal physician.

COMPENSATION: Payment will be based upon the amount of times we collect blood from you.

- Groups A and B, vaccination phase:
  - We will pay you $25 for screening blood work.
  - Payment during the “Vaccination Phase” will be $100 for each blood-drawing visit ($900.00 if all visits are kept).
- For volunteers receiving malaria challenge: In addition to the amount for vaccination phase, you will be paid $100 daily for routine blood draws associated with the “Challenge Phase” of the study.
  - Day of Challenge: $100.00
  - Days 1, 5 – 9, after challenge: $600.00
  - Days 10 – 20, during the hotel stay: up to $1000.00
  - Post hotel, negative volunteers: up to $800.00
  - Post hotel, weekly follow-up visits: $500.00
  - Total may be up to $3000.00
  - However, if you become infected with malaria during the hotel phase of the study, you will receive less money, since your blood will be drawn fewer times. An average amount would be about $1625.00.
- Active duty personnel will be paid $50 per blood draw, unless the visits occur during off duty hours or they are on leave. In this case, they will be paid the same as non-military personnel. All scheduled blood draws are planned to occur during off duty hours.
- Other than medical care that may be provided and any other payment specifically stated in this informed consent, there is no other compensation available for your participation in this research study; however, you should also understand that this is not a waiver or release of your legal rights.

COSTS:

There is no cost to you to participate in the study. However, while you are in the hotel you will need to pay for extra expenses such as long-distance phone calls, meals and movies that you may want. Please note that we are not able to provide extra money to pay for your transportation to and from the study site or childcare, if applicable.

MEDICAL CARE FOR RESEARCH RELATED INJURY:

If you are hurt or get sick because of this research study, you can receive medical care at an Army hospital or clinic free of charge. You will only be treated for injuries that are directly caused by the research study. The Army will not pay for your transportation to and from the hospital or clinic. If you have questions about this medical care, talk to the principal investigator for this study, Dr. Michele D. Spring, 301-319-9307.

RESEARCH RELATED INJURY

If you pay out-of-pocket for medical care elsewhere for injuries caused by this research study, contact the principal investigator, Dr. Michele D. Spring, 301-319-9307. If the issue cannot be resolved, contact the U.S. Army Medical Research and Materiel Command (USAMRMC) Office of the Staff Judge Advocate (legal office) at (301) 619-7663.

CONFIDENTIALITY:

Records relating to your participation as a research volunteer will remain confidential as far as possible. However, there are some instances where we cannot guarantee complete confidentiality.

- We are required to report information regarding certain infectious diseases (like HIV or AIDS and hepatitis) to the local health department where you live. If your blood tests show that you have one of these infections, we will report this information to the health department, and they may need to interview you to get more information.
- For volunteers who are in the military, we are required to report the same kind of information to military medical authorities. As a result, this information may end up in your military medical record and may be reported to your chain of command.
- For military service members, if we become aware of offenses under the Uniform Code of Military Justice (UCMJ), we may report them to your chain of command. Depending on the response from your commanding officer, you may be subject to disciplinary or administrative action as a result.
- Representatives from the following agencies may have access to review research records as part of their responsibility to protect humans in research and oversee the quality of the research efforts. As government agencies, they must also maintain confidentiality of your records within the limits of the law.
  - U.S. Army Medical Research and Materiel Command (USAMRMC)
  - The sponsor of this study (USAMMDA)
  - The Human Use Review Committee (HURC) of WRAIR
  - The Food and Drug Administration (FDA)

VOLUNTARY PARTICIPATION:

Participation in this study is voluntary. Refusal to participate will involve no penalty or loss of benefits to which you are otherwise entitled. You may discontinue your participation at any time without penalty or loss of benefits.

WITHDRAWAL FROM PARTICIPATION:

Withdrawal from this study before it has been completed may have an effect on your health.

- - If you choose to withdraw from the study during the vaccination phase, we will not be able to monitor your health for bad effects from the investigational vaccine. Therefore, if you have been vaccinated, we will request you complete an exit physical examination, and have standard safety laboratory tests drawn (blood count, liver, and kidney function) before you withdraw from the study.
  - If you withdraw after being challenged with malaria, we will ask you to undergo an exit physical exam, assess standard safety labs and take the full malaria medication treatment. We may also request additional follow-up blood tests to check for malaria, to ensure your health.
- Additionally, if you withdraw from the study, you may request for us to destroy your blood samples and not test them.

TERMINATION BY INVESTIGATOR WITHOUT YOUR CONSENT:

The principal investigator, Dr. Michele D. Spring, may decide not to allow you to continue participating in this study under the following conditions:

- If you develop health conditions that would make it dangerous to you or others if you continue participating
- If other situations or conditions arise that would make participation harmful to your own health,
- If you express plans or known illegal activities that may place you or anyone in the community in danger (we reserve the right to inform the local authorities of this).
- If you don’t follow the plan and instructions in this document.

SIGNIFICANT NEW FINDINGS:

We will tell you if we discover any significant or new information during the study that may affect your health and willingness to continue participation. When we tell you, we will also ask you to confirm your willingness to continue participation by signing another consent form like this which describes the significant or new information.

VOLUNTEERS’ RIGHTS:

If you have questions about your rights as a research volunteer in this study, you may contact the Human Subjects Research Review Board, U.S. Army Medical Research and Materiel Command (USAMRMC), 301-619-7802 or the Office of the Staff Judge Advocate (legal office), also at USAMRMC; 301-619-7663.

- The Army is sponsoring this study with some financial support from the Malaria Vaccine Initiative (MVI). The MVI is a focused vaccine development program under PATH (Program for Appropriate Technology in Health) and funds projects that test promising malaria vaccines in humans. The are located at 7500 Old Georgetown Road, 12^th^ Floor, Bethesda, MD 20814, tel: (240) 395-2700
- GlaxoSmithKline Biologicals (GSK BIO) is providing the adjuvant systems (AS01B and AS02A) and expert technical and monitoring assistance. This company has some offices in the U.S., but its main headquarters are in Europe. Therefore, they are required to use special wording in their research protocols. The European Union Data Protection Directive requires them to use this special wording. This wording is designed to ensure that you are aware of your rights. This wording is also designed to ensure that you are also clearly informed about how your personal data will be used and maintained confidentially, both during and after the research study is conducted.

1. Your personal data, including data relating to your health, will be recorded and processed for the purpose of assessing the outcome of the study. Processing will be done by WRAIR/MVI/GSK or may be contracted to a third party under strict confidentiality rules. Your data may also be processed for product registration and for notification to organizations monitoring the safety and effectiveness of medicines. Your data may also be processed in order to add to scientific knowledge;

2. Your participation in the study will be treated as confidential. You will not be referred to by name in any report concerning the study, nor will your identity be disclosed to any person, other than in circumstances such as where a need arises to check the correctness or completeness of data, or where there is a need to provide such information to regulatory agencies responsible for registration and safety of medicines.)

3. Your medical data may be sent to and processed by any affiliate of GSK in any country inside or outside the European Union, always respecting the requirements of the EU Data Protection Directive (95/46/EC) and/or the equivalent applicable law.

4. You may access your personal data and have any justifiable corrections made. If you wish to do so, you should request this from the doctor conducting the study. You agree to the postponement of your access to your medical data up to the completion of the study, including analysis and reporting of data, if deemed appropriate by the doctor conducting the study in order to safeguard the aim and conduct of the study. Representatives of WRAIR, USAMMDA, USAMRMC, MVI, GSK or regulatory bodies for medicines may access your records from this study.

VOLUNTEER REGISTRY

It is the policy of the U.S. Army Medical Research and Materiel Command (USAMRMC) that data sheets are to be completed on all volunteers participating in research for entry into this Command’s Volunteer Registry Data Base. The information to be entered into this confidential data base includes your name, address, Social Security number, study name and dates. The intent of the data base is two-fold: first, to readily answer questions concerning an individual’s participation in research sponsored by USAMRMC; and second, to ensure that the USAMRMC can exercise its obligation to ensure research volunteers are adequately warned (duty to warn) of risks and to provide new information as it becomes available. The information will be stored at USAMRMC for a minimum of 75 years.

**POINTS OF CONTACT: 24 Hour Phone (301) 319-9331**

Please contact one of the below if you have any questions concerning the medical research study or if you have any other questions or concerns:

**Clinical Trials Department, WRAIR** (301) 319-9660

Michele D. Spring, M.D.,(Principal Investigator) (301) 319-9307

James F. Cummings, M.D. (301) 573-0781

Kent E. Kester, M.D. (301) 319-9234

D. Gray Heppner, M.D. (301) 319-9414

Christian Ockenhouse, M.D., Ph.D. (301) 319-9473

Douglas S. Walsh, M.D. (301) 319-9828

COPY OF CONSENT FORM

We will give you a copy of this consent form after you sign it.

I do______ do not______ (check one & initial) wish to have a second copy of my consent form provided to me so that I may place it in my medical record.

**IF THERE IS ANY PORTION OF THIS CONSENT EXPLANATION SHEET THAT YOU DO NOT UNDERSTAND, PLEASE ASK THE INVESTIGATOR BEFORE SIGNING.**

Satisfactory performance on comprehension test (Yes/No):________ (investigator initials)

____________________________________________________________________

SIGNATURE OF VOLUNTEER DATE

____________________________________________________________________

NAME OF VOLUNTEER PRINTED

____________________________________________________________________

PERMANENT ADDRESS OF VOLUNTEER (including city, state and zip) PRINTED

____________________________________________________________________

INVESTIGATOR’S SIGNATURE DATE

INVESTIGATOR’S NAME (PRINTED) DATE

**Volunteer Schedule – Immunization Phase for Cohort 2 (Full Dose) volunteers**

*If, after beginning the study, you are unable to keep an appointment, or have any questions, please call 301-319-9660.*

| **Visit#** | **Study Day** | **Scheduled Visit  Date** | **Time Allotment**  **(approximate)** | **Activity** | **Compensation** |
| --- | --- | --- | --- | --- | --- |
| 1 | Screen | 90 to 3 days prior to start | 2 hours | Study explanation, informed consent process, blood draw for lab work, history/physical | $25.00 |
| 2 | Screen #2 | 90 to 3 days prior to start | 30 minutes | Completion of screening ( blood draw for lab work) | $100.00 |
| **3** | **0** |  | **Immunization # 1,**  **1 to 1.5 hours** | **Brief history/physical, vital signs, blood draw. women: urine pregnancy test  *(if your pregnancy test is positive, you will not be immunized).***  **Immunization, followed by 30 min. evaluation.**  **Receive diary card, thermometer and emergency contact card.** | $100.00 |
| 4-6 | 1,2 and 3 |  | 30 minutes | See physician for interim history, including any medications taken.  Bring diary for review. | *none* |
| 7 | 7 |  | 30 minutes | See physician for interim history, including any medications taken.  Return diary. | *none* |
| 8 | 14 |  | 30 minutes | See physician for interim history, including any medications taken.  Blood draw | $100.00 |
| **9** | **28** |  | **Immunization # 2,**  **1 to 1.5 hours** | **Brief history/physical, vital signs, blood draw. Women: urine pregnancy test  *(if your pregnancy test is positive, you will not be immunized).***  **Immunization, followed by 30 min. evaluation. Receive diary card.** | $100.00 |
| 10-12 | 29, 30 and 31 |  | 30 minutes each day | See physician for interim history, including any medications taken.  Bring diary for review. | *none* |
| 13 | 35 |  | 30 minutes | See physician for interim history, including any medications taken.  Return diary. | *none* |
| 14 | 42 |  | 30 minutes | See physician for interim history, including any medications taken.  Blood draw | $100.00 |
| **15** | **56** |  | **Immunization # 3,**  **1 to 1.5 hours** | **Brief history/physical, vital signs, blood draw. women: urine pregnancy test  *(if your pregnancy test is positive, you will not be immunized).***  **Immunization, followed by 30 min. evaluation. Receive diary.** | $100.00 |
| 16-18 | 57, 58 and 59 |  | 30 minutes each day | See physician for interim history, including any medications taken.  Bring diary for review. Blood draw on D57 & 59 | $200.00  *($100 per blood draw on D57 & D59)* |
| 19 | 63 |  | 30 minutes | See physician for interim history, including any medications taken.  Return diary. | *none* |
| 20 | 70 |  | 30 minutes | See physician for interim history, including any medications taken.  Blood draw | $100.00 |

**Volunteer Schedule – Challenge Phase for Cohort 2 (Full Dose) volunteers**

| **Days from "Day of Challenge”** | **Scheduled Visit Date:** | **Time Allotment**  ***(approximate)*** | **Activity** | **Compensation** |
| --- | --- | --- | --- | --- |
| 0 | **Day of Challenge** | 2 hours | Blood draw. History, directed PE. Women: urine pregnancy test. (*If positive, you will not be challenged*),  **CHALLENGE**- (3^rd^ floor WRAIR), followed30 min. evaluation. New emergency contact card issued. | $100 |
| 1 |  | 20 min | Blood draw. History, directed PE.  Call Investigator immediately if feel unwell. | $100 |
| 5-9 |  | 20 min each day | Blood draw on Days 5-9. History, directed PE.  Continue to call Investigator immediately if feel unwell. | $100/day for each blood draw |
| 10-20 | Hotel Phase *(including weekends/holidays)* | 20 min per day or more if ill | Daily morning assessment, blood draw. If you are found to be ill, you will be given medication and monitored. ***Required to sleep in hotel each evening, where a physician will be on call to provide care if necessary.***  After treatment, at the Investigator's discretion, you may be cleared from staying at the hotel. | $100/day for every day required to have blood draw |
| 21-30  *Applicable to volunteers who remain negative for malaria* | Post-Hotel-Phase  *(weekends/holidays as per PI discretion)* | 20 min | Volunteers not diagnosed with malaria will be required to come to the Clinical Trials Center  for up to 10 more 'daily assessments', including blood draws. | $ 100/day for each blood draw |
| 28 *(approximately)* | Week 1, post-hotel  *(individualized)* | 20 min | Blood draw. History, directed PE.  Continue to call Investigator immediately if feel unwell | $100 |
| 35 *(approx)* | Week 2, post-hotel  *(individualized)* | 20 min | Blood draw. History, directed PE.  Continue to call Investigator immediately if feel unwell | $100 |
| 42 *(approx)* | Week 3, post-hotel  *(individualized)* | 20 min | Blood draw. History, directed PE.  Continue to call Investigator immediately if feel unwell | $100 |
| 49 *(approx)* | Week 4, post-hotel  *(individualized)* | 20 min | Blood draw. History, directed PE.  Continue to call Investigator immediately if feel unwell | $100 |
| 84 | Study completion | 20 min | Blood draw. History, directed PE. Women: urine pregnancy test.  Continue to call Investigator immediately if feel unwell | $100 |

*If, after beginning the study, you are unable to keep an appointment, or have any questions, please call 301-319-9660.*

Appendix H: Volunteer Agreement Affidavit (Controls)

**_________________________________________________________________________________**

**VOLUNTEER AGREEMENT AFFIDAVIT**

For use of this form, see AR 70-25 or AR 40-38, the proponent agency is OTSG

**_________________________________________________________________________________**

PRIVACY ACT OF 1974

______________________________________________________________________________________________________________________________________________________

**Authority:**  10 USC 3013, 44 USC 3101, and 10 USC 1071-1087

**Principal Purpose:**  To document voluntary participation in the Clinical Investigation and Research Program, home address and phone will be used for identification and location purposes.

**Routine Uses:** Home address will be used for identification and locating purposes. Information derived from the study will be used to document the study, implementation of medical programs, adjudication of claims, and for the mandatory reporting of medical conditions as required by law. Information may be furnished to Federal, State, and local agencies.

**Disclosure:** The furnishing of your home address is mandatory for participation and necessary to provide identification and to contact you if future information indicates that your health may be adversely affected. Failure to provide the information will preclude your voluntary participation in the investigational study.

**_________________________________________________________________________________________**

**Part A - VOLUNTEER AFFIDAVIT**

**______________________________________________________________________________________________________________**

Volunteer Participants in Approved Department of the Army Research Studies

______________________________________________________________________________________________________________________________________________________

Volunteers under the provisions of AR 40-38 and AR 70-25 are authorized all necessary medical care for injury or disease which is the proximate result of their participation in such studies.

I, ___________________________________, having full capacity to consent and having attained my ________birthday, do hereby volunteer/give consent to participate in the protocol entitled

**“Phase I/IIa double-blind, randomized study of safety, immunogenicity, and preliminary efficacy after primary sporozoite challenge of FMP2.1/AS01B and FMP2.1/AS02A candidate malaria vaccines administered intramuscularly at months 0, 1, and 2 in healthy malaria-naïve adults living in the United States”**

under the direction of Dr. Michele D. Spring, Department of Immunology, conducted at Walter Reed Army Institute of Research (WRAIR), Silver Spring, MD 20910. The sponsor of this study is the Department of Defense.

**Dr. Michele D. Spring, Department of Immunology, WRAIR, phone: (301) 319-9307**, or one of the assisting investigators has explained the:

- implications of my voluntary consent and participation, and
- duration and purpose of the research study
- methods and means by which it is to be conducted, and
- inconveniences and risks that may reasonably be expected

I have been given an opportunity to ask questions concerning this investigational study. Any such questions were answered to my full and complete satisfaction. Should any further questions arise concerning my rights or study-related injury, I may contact The Office of Research Protections U.S. Army Medical Research and Materiel Command, Fort Detrick, MD 21702-5012; phone: (301) 619-2165.

I understand that I may at any time during the course of the study withdraw my consent and end participation from the study without further penalty or loss of benefits. However, I may be required (military volunteer) or requested (civilian volunteer) to undergo certain examinations if, in the opinion of the attending physician, such examinations are necessary for my health and well-being. My refusal to participate will involve no penalty or loss of benefits to which I am otherwise entitled.

**_____________________________________________________________________________________PART B – TO BE COMPLETED BY INVESTIGATOR_________________**

______________________________________________________________________________________________________________________________________________________

This research study is being conducted at the Walter Reed Army Institute of Research Clinical Trials Center by the following investigators: Dr. James F. Cummings, Dr. D. Gray Heppner, Dr. Kent Kester, Dr. Christian Ockenhouse, Dr. Douglas Walsh, and Dr. Michele Spring.

Your participation in this study is voluntary. Before you decide whether or not to participate, we encourage you to read the information below. Please ask questions about anything you do not understand.

PURPOSE OF THE STUDY

The overall goal of this study is to test the safety of two investigational malaria vaccines called FMP2.1/AS01B and FMP2.1/AS02A, and to see if these vaccines can stimulate (activate) a person’s immune system to prevent malaria infection. The investigational vaccines used in this study are not approved by the Food and Drug Administration (FDA) for commercial use, however, the FDA has permitted their use in this research study.

Malaria is a disease that affects 300-500 million people throughout the world. Humans catch this disease by being bitten by mosquitoes that are infected with malaria parasites. Mosquitoes can transmit malaria parasites to humans when they bite humans to feed on human blood. If malaria is not diagnosed and treated promptly, it can be quite serious and sometimes deadly.

The study is done in two parts. The first part, already underway, involved a group of people who volunteered to get the investigational vaccines. There isn’t a blood test to see if the vaccine works, so the second part involves exposing some of those volunteers who received the vaccines to malaria infected mosquitoes. This is called a ‘challenge’. If they don’t become positive for malaria, we can assume either the vaccines worked, or the mosquitoes were not able to transmit malaria. To ensure the mosquitoes can transmit malaria, we ask volunteers who haven’t been vaccinated to participate as a ‘control’ group, and only take part in the challenge, or exposure to malaria infected mosquitoes. If the control volunteers become positive, and the vaccinated volunteers don’t, then we know the vaccine worked.

You are being asked to participate in the control group, and you **WILL NOT RECEIVE ANY VACCINE**. We will allow mosquitoes that are infected with malaria to bite you under controlled conditions, and **WE EXPECT YOU TO DEVELOP MALARIA FROM THE MOSQUITO BITE**. We will provide treatment to cure your infection.

SIMILAR STUDIES:

The WRAIR Clinical Trials Center has vast experience in similar malaria vaccine clinical trials. Over the past 15 – 20 years, we have let malaria infected mosquitoes bite approximately 500 volunteers, and all volunteers recovered from their malaria infections. No volunteers who got sick with malaria ever required hospital admission to treat their infections, and no volunteers ever died of malaria.

NUMBER OF VOLUNTEERS: Control volunteers: 6, and volunteers who were vaccinated: up to 20. This site will be the only site where this study is done.

DURATION OF PARTICIPATION: The total duration of your participation in this study will be for approximately 3 months, not including screening time.

- During this time, you may have 14 - 21 appointments. Each of the appointments will take 15 minutes - 2 hours to complete.
- You will also sleep at a nearby hotel for up to 10 nights.
- A more complete schedule with the expected amount of time that you will need to be available for each appointment is provided at the end of this form.

PROCEDURES:

1. Consent Phase:

- If you agree to participate in this study, you will be asked to sign 3 separate consent forms:
- In your 1st consent form, you will agree to participate in the study.
- In a 2nd consent form, you will give us permission to test your blood for HIV, the virus that causes AIDS.
- A 3rd consent form is optional, with which you may give us permission to keep your blood for future testing.
- Additionally, there is a short multiple-choice quiz that assesses your understanding of this study. A passing score of 80% correct is required. If you do not score 80% on the initial quiz, we will review the study information with you to help you to more completely understand it. You will then have a 2nd chance to take the test and score at least 80%.

2. Screening Phase:

After you have signed the consent forms, you will start the “Screening Phase”. A physician and other medical staff will check to make certain that you are healthy. In order to participate in this study you must be a healthy adult who is available for the complete duration of the study (approximately 3 months, not including screening time). A time line of the study dates will be provided to you.

- Specific things that could prevent you form participating in this study:
  - Planned travel to an area with malaria
  - Previous history of malaria or malaria vaccine
  - Exposure to malaria within the past year or malaria medications within a month of planned malaria challenge
  - Certain prescription medications
  - HIV or problems fighting infections, such as cancer or transplants
  - Severe problems with your heart, liver, lungs or kidneys
  - History of either sickle cell disease or sickle cell trait
  - Pregnancy (now, or planned within the study period), or breast-feeding
  - Chronic or active drug abuse
  - History of severe allergic reaction to mosquito bites
  - Chronic or active neurological disorders (i.e. problems with your brain or nervous system such as seizures, migraines, etc.)
- You will undergo a screening evaluation with a medical history and physical examination. In addition to questions about your health, you will be asked about your use of illegal substances and alcohol.
- You will also give blood and urine samples for routine laboratory tests. We will need to collect approximately ¼ cup of blood from you at this screening visit. Blood and urine samples for routine laboratory testing may be sent to either the Walter Reed Army Medical Center clinical laboratory or another contract clinical laboratory. The routine tests will include tests for HIV, hepatitis B and hepatitis C. If your tests show that you have the HIV or hepatitis B or C infection, you will be notified in a confidential manner and be given information on where you can receive medical treatment. You will not be allowed to participate in this study if you are found to be infected with HIV or hepatitis B or C. We will give you the results of our medical history, physical exam and lab tests so that you can give them your regular doctor.
- If any of your lab tests show that you are infected with HIV or any of the hepatitis viruses, we are required by law to report these tests results to local and state governmental health agencies. If you are a military service member, this information will be reported to the military preventive medicine service. In either case, additional information and/or interviews may be required for contact tracing.
- If you are female, we will test if you are pregnant with a urine test now and within 48 hours before the challenge with the infected mosquitoes. Female volunteers with a positive pregnancy test will not be allowed to participate in the study.
- If you are not healthy or it would not be safe for you to continue to participate in the study, you will be advised to seek medical care and/or counseling. At your request, we will inform your regular physician of any lab test results.

3. Challenge Phase:

The Challenge Phase will help us to determine if the vaccine works.

- If you are female, we will perform a urine pregnancy test 48 hours before the malaria-infected mosquitoes bite you. If your pregnancy test is positive, we will not allow you to be bitten by the infected mosquitoes.
- We will let a small number of malaria-infected mosquitoes bite you. Initially, we allow 5 mosquitoes to bite you for a period of five minutes. If all 5 mosquitoes do not bite you, we will let more mosquitoes bite for you five minutes, until a total of 5 malaria-infected mosquitoes have bitten you.
- In the days after the infected mosquitoes have bitten you, we will check you for signs of malaria infection. WE EXPECT YOU TO DEVELOP MALARIA FROM THE MOSQUITO BITE.
- Malaria usually develops between 9 and 15 days after an infected mosquito bites you. Starting 5 days and for up to 30 days after the mosquitoes have bitten you, we will collect approximately 1 teaspoonful – 1 tablespoonful of blood from your vein once daily (or more often if you develop fever or other problems). We will look at your blood under a microscope and try to see malaria parasites in your blood. These samples will be saved for later re-examination and confirmation of disease.
- To describe the daily visits more fully:
- We will see you in the Clinical Trials Center, WRAIR on days 5 – 9, which will be a Monday through Friday schedule.
- Days 10 – 20, we will move the clinical team and volunteers into a local hotel, in order to be available at any time you may need us. We will see you every day, including weekends. The hotel phase is described more fully shortly.
- Although not expected, if you have not been diagnosed with malaria throughout the hotel phase, we will continue daily assessments at the Clinical Trials Center, WRAIR during the work week, but not weekends or holidays. However, we ask that you have a way to contact the study investigators at any time, should you need to.
- Symptoms of malaria may include fever, headache, joint aches, generalized body aches, shaking chills, nausea and/or diarrhea. FDA approved medications for the symptoms will be available for doctors to give to you as needed. These include, but aren’t limited to: Tylenol™ (acetaminophen) and Motrin™ (ibuprophen) for pain or fever, Tigan™ for nausea, and Imodium™ for diarrhea. The doctors will make sure to check if you are allergic to the medicines before using them. An alternative medicine will be available if you are allergic to the prescribed medicine.
- Malaria symptoms may develop either before or after the parasites are seen in your blood test. Approximately 1/3 of the time, specialists are able to detect the parasites before you feel symptoms.
- If we see malaria parasites in your blood, we will treat your infection with an FDA approved drug that treats malaria. This medicine is called chloroquine and is a pill taken by mouth over three days. You will be seen every day during the treatment course, until we see no malaria parasites in your blood for 3 days in a row. From previous experience, we expect that the malaria parasites will disappear from your blood within two or three days after we treat you with chloroquine. People usually don’t have problems with this medicine, but if it causes side effects that require you to stop taking it, or if it doesn't get rid of the malaria parasites, other effective drugs are available and will be used (such as Malarone™ or doxycycline and quinine). Common side effects of chloroquine and other anti-malaria drugs may include headache, dizziness, visual disturbances, loss of appetite, nausea, vomiting, abdominal cramps, diarrhea, ringing in the ears, or a rash.
- After your blood has had no parasites for 3 days in a row, we will continue to see you for follow-up examinations once a week for four weeks. During these visits, you will be examined and asked about symptoms of malaria because there is a very small (less than 1%) theoretical chance that your malaria infection could come back and make you ill. If you get sick again with malaria (this is called a recurrence), we will give you a different medicine to treat the infection. After the recurrence is cured, we will continue to follow you at once a week for another four weeks. In our history of malaria vaccine development at the WRAIR, no volunteers have ever had a malaria recurrence.

4. Hotel Phase:

A group of hotel rooms in the local area will be reserved for malaria challenged volunteers. All volunteers who have gone through the challenge are required to spend every night at the hotel for approximately 10 nights, beginning on the night of ‘Day 9’ after the mosquitoes bite you. During the day you can leave the hotel to go to work or school or do whatever you would like to do as long as you feel up to it.

- You will have a daily study visit in the hotel. This means you must check in with the study staff in the hotel each morning. At that visit, we will ask you how you are feeling and draw your blood to check for parasites. In the afternoon, we will establish contact with you to check on how you are feeling, and tell you if you have parasites in your blood and thus need to be treated.
- Staying in the hotel at night allows the physicians and medical staff to evaluate you very quickly and easily at night. At least one physician will be on-duty and present in the hotel every night. The on-duty physician will check your blood test results and treat you if you develop malaria.
- If you do not develop malaria symptoms and we do not see malaria parasites in your blood within 30 days after the mosquitoes bite you, this means that the vaccine worked and the malaria parasites could not cause a malaria infection in you. We will, however, continue to follow you once a week for four additional weeks to make sure you do not have any other symptoms.
- The longest you will have to stay in the hotel stay will be ten nights. If you develop malaria and are treated, you will not need to remain in the hotel at night. Only a study investigator may release you from the daily visits.
- WRAIR will pay for the hotel rooms. You will need to pay for extra expenses such as long-distance phone calls, meals and movies that you may want.

5. Blood Collections:

We will be collecting blood samples from you during your clinic appointments and also when you are in the hotel. We will collect different amounts on different days. Some days we will only collect 2 milliliters of blood (about ½ teaspoonful). Other days we will need to collect as much as 60 milliliters (about ¼ cup). These blood tests will help us check the health of your bone marrow, kidney and liver and to measure how well your body is fighting the malaria infection. We will not collect more than 247 milliliters (about 1 ¼ cup) of blood from you during any 8-week period of the study. We expect that your body will be able to make enough blood to replace this loss without harming you.

- We will give you a more detailed summary of the blood-drawing schedule during this screening session.
- In a separate consent document we will ask you to allow us to collect additional blood that may be used in other malaria research studies and might be used to develop other products. Any blood that you provide will have your name and identifying information removed. (See Appendix J, Blood Donation Form).

PRECAUTIONS:

1. You will receive a copy of this form. In addition, we will provide you with a list of appointment dates and times to come to the WRAIR Clinical Trials Department.
2. If you develop any medical problems that you think might have been caused by the vaccination, please call or come to the WRAIR Clinical Trials Department.
3. Some antibiotics (medicines that fight infection) prevent the malaria parasite from growing. Therefore, you should not take any antibiotics, unless it is absolutely necessary, beginning 2 weeks before the challenge and for at least 2 months after the malaria-infected mosquitoes bite you. If your personal physician prescribes an antibiotic for you, you should inform one of the study physicians, preferably before you begin to take it. The study physician will then have a chance to talk with your personal physician about possible using a different antibiotic which would not interfere with the study. If your physician agrees to use the antibiotic that we recommend, we will provide this antibiotic to you at no cost.
4. Mosquitoes may not try to bite you and feed on your blood if you have used cologne, deodorant soap or lotion on your skin on the day of the bite. Therefore, please do not use such items on your skin on the challenge day.
5. For Women: It is very important that you read, understand and follow these instructions :

- You should not be pregnant at the time the mosquitoes bite you. A small amount of your urine will be used to do a pregnancy test. This pregnancy test must be done during screening and again 2 days before the scheduled mosquito bites. A final pregnancy test will be done at the conclusion of the study.
- If a female volunteer has a positive pregnancy test, they will not be bitten with malaria-infected mosquitoes.
- You should avoid becoming pregnant if you want to continue your participation in this study. To avoid becoming pregnant, you should either abstain from sexual relations or practice a method of birth control. Except for surgical removal of the uterus, birth control methods such as the use of condoms, a diaphragm or cervical cap, birth control pills, IUD, or sperm killing products are not totally effective in preventing pregnancy.
- You should not become pregnant for 2 months after the malaria-infected mosquitoes bite you. The malaria infection within the 2-month period after the pregnancy may create a potential risk to your unborn baby.
- Urine pregnancy tests are not able to pick up very early pregnancies. Therefore, if the pregnancy test is negative but you still think that you might be pregnant, you should not participate in the study. The only way that you can guarantee that you are not pregnant is to not have sex with a male person.
- You should notify the investigator if you find out after the study is over that you became pregnant either during the study of within 30 days of the study.
- If you become pregnant while you are participating in the study, we will ask to be allowed to follow your pregnancy through its outcome. This will include information about both your health status and that of the child. The information will be reported confidentially (without names or identifying criteria) to WRAIR (Walter Reed Army Institute of Research), USAMMDA (The U.S. Army Medical Materiel Development Activity), USAMRMC ORP (US Army Medical Research and Materiel Command, Office of Research Protections), MVI (Malaria Vaccine Initiative), GlaxoSmithKline Biologicals and the Food and Drug Administration (FDA).

POTENTIAL RISKS and DISCOMFORTS:

In order to prevent or minimize all possible risks and all possible hazards associated with this study, our medical team will observe you very closely and offer you U.S. standard medical care and treatment for malaria. Our physicians will give you frequent physical examinations and conduct multiple laboratory tests as outlined above. A study physician will always be on call at one of the phone numbers listed below. Specific risk/discomfort information is outlined below:

General items:

- It is very important for you to take all the malaria treatment medicines that we give you; otherwise you are more likely have the malaria infection recur. This is an important risk for you to understand. However, in our nearly 20 years of doing studies like this, we have never had a repeat infection of this type.
- If you plan to travel outside the Washington metropolitan area on vacation or leave, even for one day, during the year after this study, discuss your plans with one of the physician investigators listed below so that they can advise you about additional precautions. We will give you a special card that identifies you as a participant in this clinical trial. The card will have detailed instructions for contacting a study physician 24 hours a day.
- In the future, should you travel to a place where malaria is common, it will be necessary to take standard precautions to prevent malaria.
- We ask that you not participate in other medical research studies while you are in this study.
- You must not receive any other investigational drugs or vaccines within the 30 days before the challenge.

From malaria: We expect you to get sick with malaria several days after the malaria-infected mosquitoes bite you.

- Malaria is a serious infection and untreated, can lead to death. That is why it is so important to take all of the medication and follow the physician investigators instructions. However, under the very carefully controlled conditions of this study, the chance of serious illness or death from malaria infection is very small.
- When you get sick with malaria, you will commonly develop a combination of the following symptoms: fever, headache, joint pain, generalized body aches, shaking chills, nausea (become sick to your stomach), vomiting, diarrhea, or feel pretty sick all over. You might also develop mild problems with your oxygen carrying and infection fighting blood cells, tenderness over your liver and feel fatigued. In untreated, malaria infections damage your kidney, liver or brain, and can even kill you.
- Therefore, when you get sick with malaria, we will treat you at no cost and give you FDA approved anti-malarial drugs to treat the infection.
- There is a chance if you get sick with malaria, and if we treat you and you recover, that you may get sick again with malaria, although this has not occurred in any previous malaria vaccine studies at WRAIR.. You could get sick with malaria a second time at any time up to 1 year after you get malaria the first time. If during that 1 year after you participate in this study and you develop symptoms that might be caused by malaria, please contact one of the physicians listed below or tell your personal physician that you had participated in this study. Malaria symptoms can include fever, headache, joint pain, muscle pains, nausea, or vomiting.
- The policy of the American Association of Blood Banks says that you will not be permitted to donate blood for three years after your treatment for malaria. During that period of time, no blood bank will accept you as a blood donor. You may or may not be able to donate blood for your own use ("auto-donation"), depending on the policies of the blood donor center or hospital where you are receiving care.

From mosquito bites

- At the sites of the mosquito bites, you might develop redness, mild swelling and itching.
  - There is also a very small but theoretical risk that the malaria-infected mosquitoes could give you a disease such as hepatitis or AIDS. A mosquito would have to feed on an HIV or hepatitis-infected human, and then be allowed to bite you.
- We do 3 specific procedures to minimize this theoretical risk:
  1. The blood for feeding the mosquitoes is obtained from an FDA-certified blood bank. People who are at risk for having infections such as HIV and hepatitis are not allowed to donate there;

b. The blood that is used to feed mosquitoes is tested by the FDA-certified blood bank to make sure that there are no Hepatitis or HIV viruses in the blood; and

c. All mosquitoes that bite you have not been allowed to feed on other human blood for at least 16 to 18 days before they bite you. This guarantees that the mosquito kills any viruses that they might have collected while feeding on human blood before the mosquito bites you.

- Specifically, there has been no reported transmission of the AIDS virus using the 3-part mosquito safeguard system described above.

From drawing your blood

- You will have blood drawn by an experienced phlebotomist (person whose job is to draw blood). You may experience some discomfort and possibly also a bruise where blood is taken from your vein for blood tests. Some people get dizzy or feel faint when their blood is drawn. There is a very small chance that you could get an infection at the place where your blood is drawn.

From loss of confidentiality

- If you participate in this study, there are also risks that information about you may be disclosed to persons outside of this study. This is discussed in the Confidentiality section of this document (below).

GENERAL BENEFITS: Volunteers will not benefit directly from participation in this research study. There may be benefit to humanity in the future related to the vaccine or study.

ALTERNATIVES TO PARTICIPATION: The alternative to participation is to refuse to be in this study.

DISCLOSURE OF RESEARCH RESULTS:

Your name will not be used in any report resulting from this study. We will not be able to disclose results of this study to any of the volunteers. However, we will give you a statement of your participation in this study, with the dates and any side effects you experienced. Also, if you request, we will give you a statement of your individual study results. You can then place these statements in your personal medical record or give them to your personal physician. If you prefer, we will mail these statements directly to your personal physician.

COMPENSATION: Payment will be based upon the amount of times we collect blood from you.

- Total amounts will vary, depending on when you become positive for malaria.
  - Screen: $25.00
  - Day of Challenge: $100.00
  - Days 1, 5 – 9, after challenge: $600.00
  - Days 10 – 20, during the hotel stay: up to $1000.00
  - Post hotel, weekly follow-up visits: $500.00
  - Total most likely will be approximately $1525.00 (with maximum up to $2225.00)
- Active duty personnel will be paid $50 per blood draw, unless the visits occur during off duty hours or they are on leave. In this case, they will be paid the same as non-military personnel. All scheduled blood draws are planned to occur during off duty hours.
- Other than medical care that may be provided and any other payment specifically stated in this informed consent, there is no other compensation available for your participation in this research study; however, you should also understand that this is not a waiver or release of your legal rights.

COSTS: There is no cost to you to participate in the study. However, while you are in the hotel you will need to pay for extra expenses such as long-distance phone calls, meals and movies that you may want. Please note that we are not able to provide extra money to pay for your transportation to and from the study site or childcare, if applicable.

MEDICAL CARE FOR RESEARCH RELATED INJURY:

If you are hurt or get sick because of this research study, you can receive medical care at an Army hospital or clinic free of charge. You will only be treated for injuries that are directly caused by the research study. The Army will not pay for your transportation to and from the hospital or clinic. If you have questions about this medical care, talk to the principal investigator for this study, Dr. Michele D. Spring, 301-319-9307.

RESEARCH RELATED INJURY:

If you pay out-of-pocket for medical care elsewhere for injuries caused by this research study, contact the Clinical Trials Center, WRAIR at 301-319-9660 or the principal investigator, Dr. Michele D. Spring, 301-319-9307. If the issue cannot be resolved, contact the U.S. Army Medical Research and Materiel Command (USAMRMC) Office of the Staff Judge Advocate (legal office) at (301) 619-7663.

CONFIDENTIALITY: Records relating to your participation as a research volunteer will remain confidential as far as possible. However, there are some instances where we cannot guarantee complete confidentiality.

- We are required to report information regarding certain infectious diseases (like HIV or AIDS and hepatitis) to the local health department where you live. If your blood tests show that you have one of these infections, we will report this information to the health department, and they may need to interview you to get more information.
- For volunteers who are in the military, we are required to report the same kind of information to military medical authorities. As a result, this information may end up in your military medical record and may be reported to your chain of command.
- For military service members, if we become aware of offenses under the Uniform Code of Military Justice (UCMJ), we may report them to your chain of command. Depending on the response from your commanding officer, you may be subject to disciplinary or administrative action as a result.
- Representatives from the following agencies may have access to review research records as part of their responsibility to protect humans in research and oversee the quality of the research efforts. As government agencies, they must also maintain confidentiality of your records within the limits of the law.
  - U.S. Army Medical Research and Materiel Command (USAMRMC)
  - The sponsor of this study (USAMMDA)
  - The Human Use Review Committee (HURC) of WRAIR
  - The Food and Drug Administration (FDA)

VOLUNTARY PARTICIPATION: Participation in this study is voluntary. Refusal to participate will involve no penalty or loss of benefits to which you are otherwise entitled. You may discontinue your participation at any time without penalty or loss of benefits.

WITHDRAWAL FROM PARTICIPATION:

- Withdrawl from this study before it has been completed may have bad effects on your health. This is because we will not be able to closely monitor you to see if you get sick with malaria, to give you anti-malarial drugs, and make sure you recover from malaria.

If you withdraw after being challenged with malaria, we will ask you to undergo an exit physical exam, assess standard safety labs and take the full malaria medication treatment. We may also request additional follow-up blood tests to check for malaria, to ensure your health.

- Additionally, if you withdraw from the study, you may request for us to destroy your blood samples and not test them.

TERMINATION BY INVESTIGATOR WITHOUT YOUR CONSENT:

The principal investigator, Dr. Michele D. Spring, may decide not to allow you to continue participating in this study under the following conditions:

- If you develop health conditions that would make it dangerous to you or others if you continue participating
- If other situations or conditions arise that would make participation harmful to your own health,
- If you express plans or known illegal activities that may place you or anyone in the community in danger (we reserve the right to inform the local authorities of this).
- If you fail to comply with the procedures as outlined in this volunteer agreement affidavit.

SIGNIFICANT NEW FINDINGS: We will tell you if we discover any significant or new information during the study that may affect your health and willingness to continue participation. When we tell you, we will also ask you to confirm your willingness to continue participation by signing another consent form like this which describes the significant or new information.

VOLUNTEERS’ RIGHTS:

If you have questions about your rights as a research volunteer in this study, you may contact the Human Subjects Research Review Board, U.S. Army Medical Research and Materiel Command (USAMRMC), 301-619-7802 or the Office of the Staff Judge Advocate (legal office), also at USAMRMC; 301-619-7663.

- The Army is sponsoring this study with some financial support from the Malaria Vaccine Initiative (MVI). The MVI is a focused vaccine development program created under PATH (Program for Appropriate Technology in Health) and funds projects that test promising malaria vaccines in humans. The are located at 7500 Old Georgetown Road, 12^th^ Floor, Bethesda, MD 20814, tel: (240) 395-2700
- GlaxoSmithKline Biologicals (GSK BIO) is providing the adjuvant systems (AS01B and AS02A) and expert technical and monitoring assistance. This company has some offices in the U.S., but its main headquarters are in Europe. Therefore, they are required to use special wording in their research protocols. The European Union Data Protection Directive requires them to use this special wording. This wording is designed to ensure that you are aware of your rights. This wording is also designed to ensure that you are also clearly informed about how your personal data will be used and maintained confidentially, both during and after the research study is conducted.

1. Your personal data, including data relating to your health, will be recorded and processed for the purpose of assessing the outcome of the study. Processing will be done by WRAIR/MVI/GSK or may be contracted to a third party under strict confidentiality rules. Your data may also be processed for product registration and

for notification to organizations monitoring the safety and effectiveness of medicines. Your data may also be processed in order to add to scientific knowledge;

2. Your participation in the study will be treated as confidential. You will not be referred to by name in any report concerning the study, nor will your identity be disclosed to any person, other than in circumstances such as where a need arises to check the correctness or completeness of data, or where there is a need to provide such information to regulatory agencies responsible for registration and safety of medicines.)
3. Your medical data may be sent to and processed by any affiliate of GSK in any country inside or outside the European Union, always respecting the requirements of the EU Data Protection Directive (95/46/EC) and/or the equivalent applicable law.
4. You may access your personal data and have any justifiable corrections made. If you wish to do so, you should request this from the doctor conducting the study. You agree to the postponement of your access to your medical data up to the completion of the study, including analysis and reporting of data, if deemed appropriate by the doctor conducting the study in order to safeguard the aim and conduct of the study. Representatives of WRAIR, USAMMDA, USAMRMC, MVI, GSK or regulatory bodies for medicines may access your records from this study.

VOLUNTEER REGISTRY

It is the policy of the U.S. Army Medical Research and Materiel Command (USAMRMC) that data sheets are to be completed on all volunteers participating in research for entry into this Command’s Volunteer Registry Data Base. The information to be entered into this confidential data base includes your name, address, Social Security number, study name and dates. The intent of the data base is two-fold: first, to readily answer questions concerning an individual’s participation in research sponsored by USAMRMC; and second, to ensure that the USAMRMC can exercise its obligation to ensure research volunteers are adequately warned (duty to warn) of risks and to provide new information as it becomes available. The information will be stored at USAMRMC for a minimum of 75 years.

**POINTS OF CONTACT:	24 Hour Phone	(301) 319-9331**

Please contact one of the below if you have any questions concerning the medical research study or if you have any other questions or concerns:

Clinical Trials Department, WRAIR	(301) 319-9660

Michele D. Spring, M.D.	(301) 319-9307

James F. Cummings, M.D.	(301) 319-9312

Kent E. Kester, M.D.	(301) 319-9234

D. Gray Heppner, M.D.	(301) 319-9414

Christian Ockenhouse, M.D., Ph.D.	(301) 319-9473

Douglas S. Walsh, M.D.	(301) 319-9828

COPY OF CONSENT FORM

We will give you a copy of this consent form after you sign it.

I do______ do not______ (check one & initial) wish to have a second copy of my consent form provided to me so that I may place it in my medical record.

**IF THERE IS ANY PORTION OF THIS CONSENT EXPLANATION SHEET THAT YOU DO NOT UNDERSTAND, PLEASE ASK THE INVESTIGATOR BEFORE SIGNING.**

Satisfactory performance on comprehension test (Yes/No):________ (investigator initials)

____________________________________________________________________

SIGNATURE OF VOLUNTEER	DATE

____________________________________________________________________

NAME OF VOLUNTEER	PRINTED

____________________________________________________________________

PERMANENT ADDRESS OF VOLUNTEER (including city, state and zip)	PRINTED

____________________________________________________________________

INVESTIGATOR’S SIGNATURE	DATE

INVESTIGATOR’S NAME (PRINTED)	DATE

**Volunteer Schedule – Challenge Phase for infectivity controls**

| **Days from**  **"Day of Challenge”** | **Scheduled Visit Date:** | **Time Allotment**  ***(approximate)*** | **Activity** | **Compensation** |
| --- | --- | --- | --- | --- |
| 90 to 3 days prior to challenge | Screen | 2 hours | Study explanation, informed consent process, lab work, history/physical | $25 |
| 0 |  | 2 hours | Blood draw. History, directed PE. Women: urine pregnancy test. (*If positive, you will not be challenged*),  CHALLENGE- (3^rd^ floor WRAIR), followed30 min. evaluation. New emergency contact card issued. | $100 |
| 1 |  | 20 min | Blood draw. History, directed PE. Call Investigator immediately if feel unwell. | $100 |
| 5-9 |  | 20 min each day | Blood draw on Days 5-9. History, directed PE.  Continue to call Investigator immediately if feel unwell. | $100/day |
| 10-20 | Hotel Phase *(including weekends/holidays)* | 20 min per day or more if ill | Daily morning assessment, blood draw. If you are found to be ill, you will be given medication and monitored. ***Required to sleep in hotel each evening, where a physician will be on call to provide care if necessary.***  After treatment, at the Investigator's discretion, you may be cleared from staying at the hotel. | $100/day for every day required to have blood draw |
| 21-30  *Applicable to volunteers who remain negative for malaria* | Post-Hotel-Phase  *(no weekends/holidays)* | 20 min | Volunteers not diagnosed with malaria will be required to come to the Clinical Trials Center  for up to 10 more 'daily assessments', including blood draws. | $ 100/day for each blood draw |
| 28 *(approximately)* | Week 1, post-hotel  *(individualized)* | 20 min | Blood draw. History, directed PE.  Continue to call Investigator immediately if feel unwell | $100 |
| 35 *(approx)* | Week 2, post-hotel  *(individualized)* | 20 min | Blood draw. History, directed PE.  Continue to call Investigator immediately if feel unwell | $100 |
| 42 *(approx)* | Week 3, post-hotel  *(individualized)* | 20 min | Blood draw. History, directed PE.  Continue to call Investigator immediately if feel unwell | $100 |
| 49 *(approx)* | Week 4, post-hotel  *(individualized)* | 20 min | Blood draw. History, directed PE.  Continue to call Investigator immediately if feel unwell | $100 |
| 90 | Study completion | 20 min | Blood draw. History, directed PE. Women: urine pregnancy test.  Continue to call Investigator immediately if feel unwell | $100 |

*If, after beginning the study, you are unable to keep an appointment, or have any questions, please call 301-319-9660.*

Appendix I: CONSENT FOR HIV ANTIBODY BLOOD TEST

1. I, consent to have a blood test for the presence of antibodies to the Human Immunodeficiency Virus (HIV), the cause of AIDS, as part of the screening tests necessary to participate in the malaria vaccine protocol, **“Phase I/IIa double-blind, randomized study of safety, immunogenicity, and preliminary efficacy after primary sporozoite challenge of FMP2.1/AS01B and FMP2.1/AS02A candidate malaria vaccines administered intramuscularly at months 0, 1, 2 in healthy malaria-naïve adults living in the United States”**

2. It has been explained to me that a positive test result would need to be confirmed through a second test.

3. It has been explained to me that the presence of antibodies to HIV means that a person has been infected with the virus and that an infected person can spread the HIV virus to others through blood, sexual contact, and childbirth.

4. It has been explained to me that most persons who are infected with the HIV virus are not aware that they are infected and that infection with HIV does not necessarily mean that the person has AIDS.

5. It has been explained to me that the three most important reasons for infected persons to be made aware of their infection are:

A) Infected persons will know that they can pass the HIV virus to other persons through their blood, through unsafe sex, or through childbirth (from an infected mother to her newborn infant). That knowledge will help the individual to avoid putting others at risk of disease due to HIV.

B) Infected persons will understand the need to seek medical care for their infection, permitting them to delay the complications of HIV infection through early treatment.

C) Infected persons will be better able to make decisions about matters such as changing jobs, applying for or canceling insurance, marriage, and plans for children.

6. It has been explained to me that negative result is not absolutely conclusive of not having HIV, since the development of antibodies to HIV after exposure can take up to three to six months (“window period”).

7. The results of this test will be provided to me by one of the study investigators.

8. I have had a chance to have my questions about this test answered.

9. It has been explained to me that being HIV positive can lead to forms of discrimination by others beyond the control of the investigators.

10. It has been explained to me that HIV infection is a lifetime infection. At present there is no cure and medical treatment and follow-up will be necessary should I test positive.

11. Active-duty military personnel who test positive for antibodies to HIV will be referred to military Preventive Medicine authorities for further evaluation and testing. Positive HIV tests for non-military volunteers may also need to be reported to local public health authorities. Civilian participants who test positive for HIV will be referred to their private physician for further evaluation.

_________________________________________________________________

SIGNATURE OF VOLUNTEER	DATE

_________________________________________________________________

PRINTED NAME OF VOLUNTEER

_________________________________________________________________

SIGNATURE OF INVESTIGATOR	DATE

_________________________________________________________________

PRINTED NAME OF INVESTIGATOR

Appendix J: BLOOD DONATION CONSENT FORM

During this study, you will be asked to provide blood. These samples will be used for standard medical tests, as well as tests to see how well your immune system has been protected against malaria. These samples may also be used in research for malaria vaccine development. Should your donated samples lead to the development of a commercial product, the United States Army will own it and may take action to patent and license the product. Other than the specific payments for blood samples outlined in the Informed Consent documents, no additional compensation is intended for your participation or for any future value that the samples you have given may be found to have. You will not receive any notice of future uses of your samples.

You do not have to agree to allow your samples to be used for future malaria research.

You can still participate in the study regardless of your decision about allowing your samples to be used for future malaria research.

Please **initial below** if you do or do not wish to have your samples stored for use in future malaria research studies.

_________Samples can be stored for future malaria research use.

_________Samples to be used in the current study only. Samples cannot be stored for malaria vaccine development.

SIGNATURE OF VOLUNTEER	DATE

PRINTED NAME OF VOLUNTEER

SIGNATURE OF INVESTIGATOR	DATE

PRINTED NAME OF INVESTIGATOR

Appendix K: Informed Consent Comprehension Assessment (Cohort 1 Vaccinees)

1. If I participate in this study, I will receive an investigational new vaccine against which of the following diseases?

A. Malaria

B. Chicken Pox

C. Leprosy

2. By receiving the study vaccinations, I may develop malaria.

A. True

B. False

3. Which of the following are true regarding pregnancy and participation in this study?

A. Pregnant women may participate in this study

B. Women should not get pregnant for 12 months after immunization.

C. An effective method of birth control is required for women while participating in this study.

4. Is it possible to get malaria from the vaccines used in this study?

A. Yes

B. No

5. Medical screening for this study will include which of the following?

A. Laboratory tests (including an HIV test)

B. Physical examination

C. Review of medical history

D. All of the above

6. If you participate in this study, you may:

A. Withdraw voluntarily at any time.

B. Never withdraw from the study.

7. FMP2.1 malaria vaccine is licensed by the FDA.

A. True

B. False

8. If you enroll in this study, you will receive a full dose of one of the malaria vaccines we are testing.

A. True

B. False

9. After each immunization in this study, you will be:

A. Observed for approximately 30 minutes.

B. Permitted to go outside to smoke cigarettes.

_____________________________________________________________

Volunteer signature / date

Score: _________

_____________________________________________________________

Reviewer signature / date

Appendix L: Informed Consent Comprehension Assessment (Cohort 2 Vaccinees)

1. If I participate in this study, I will receive an investigational new vaccine against which of the following diseases?

A. Malaria

B. Chicken Pox

C. Leprosy

2. The Challenge Phase of the study involves being bitten by which of the following?

A. Spiders infected with malaria

B. Mosquitoes infected with malaria

C. Ants infected with malaria

3. By receiving the study vaccinations, I may develop malaria.

A. True

B. False

4. Which of the following are true regarding pregnancy and participation in this study?

A. Pregnant women may participate in this study

B. Women should not get pregnant for 12 months after immunization.

C. An effective method of birth control is required for women while participating in this study.

5. Is it possible to get malaria from the vaccines used in this study?

A. Yes

B. No

6. Medical screening for this study will include which of the following?

A. Laboratory tests (including an HIV test)

B. Physical examination

C. Review of medical history

D. All of the above

7. If you participate in the Challenge Phase and develop malaria, for how long will you be unable to donate blood?

A. 3 years

B. 25 years

C. Never able to donate blood again

8. What are common findings associated with malaria infection?

A. Fever

B. Chills

C. Headache

D. All of the above

E. None of the above

9. If you participate in this study, you may:

A. Withdraw voluntarily at any time.

B. Never withdraw from the study.

10. FMP2.1 malaria vaccine is licensed by the FDA.

A. True

B. False

11. Untreated malaria can result in death

A. True

B. False

12. Which of the following are components of this study?

A. Staying in a hotel each evening for up to 10 nights during the challenge phase

B. Staying overnight in a hospital for up to 10 nights during the challenge phase

C. Coming and going as I please throughout the study

13. If our specific medical instructions are not followed in this study, malaria infection can cause:

A. Death

B. Serious illness

C. All of the above

14. We diagnose malaria by which method?

A. Looking at a sample of your blood under a microscope

B. Chest X-ray

C. Having you walk on a treadmill

15. After each immunization in this study, you will be:

A. Observed for approximately 30 minutes.

B. Permitted to go outside to smoke cigarettes.

_____________________________________________________________

Volunteer signature / date

Score: _________

_____________________________________________________________

Reviewer signature / date

Appendix M: Informed Consent Comprehension Assessment (Controls)

1. This protocol asks you to help test new vaccines against which of the following diseases?

A. Malaria

B. Chicken Pox

C. Leprosy

2. I expect to get malaria from my participation in this study.

A. True

B. False

3. The challenge phase of the study involves volunteers being bitten by which of the following?

A. Spiders infected with malaria

B. Mosquitoes infected with malaria

C. Ants infected with malaria

4. By participating in this study, you can expect to develop which of the following?

A. Tuberculosis

B. Kidney stones

C. Malaria

5. Is it likely that a single treatment course will be effective to treat malaria induced by this study?

A. Yes

B. No

6. Medical screening for this study will include which of the following?

A. Laboratory tests (including an HIV test)

B. Physical examination

C. Review of medical history

D. All of the above

7. If you develop malaria, for how long will you be unable to donate blood?

A. 3 years

B. 25 years

C. Never able to donate blood again

8. What are common findings associated with malaria infection?

A. Fever

B. Chills

C. Headache

D. All of the above

E. None of the above

9. If you participate in this study, you may:

A. Withdraw voluntarily at any time.

B. Never withdraw from the study.

10. If our specific medical instructions are not followed in this study, malaria infection can cause

A. Death

B. Serious illness

C. All of the above

11. Which of the following are true regarding pregnancy and participation in this study?

A. Pregnant women may participate in this study.

B. Women should not get pregnant for 12 months after getting malaria.

C. An effective method of birth control is required for women while participating in this study.

12. Which of the following are components of this study?

A. Staying in a hotel each evening for up to 10 nights during the challenge phase

B. Staying overnight in a hospital for up to 10 nights during the challenge phase

C. Coming and going as I please throughout the study

13. Malaria is:

A. A disease transmitted by mosquitoes.

B. A disease transmitted by bad air.

14. We diagnose malaria by which method?

A. Looking at a sample of your blood under a microscope

B. Chest X-ray

C. Having you walk on a treadmill

15. If you develop malaria, we will:

A. Treat you immediately with effective medications.

B. See how sick you can get without treating you.

16. Which one of the following is true regarding medical care for problems resulting from your participation in this study?

A. We will provide all necessary medical care for injury or disease resulting from your participation in this study.

B. You will be charged for any medical care rendered for problems associated with your participation in this study.

_____________________________________________________________

Volunteer signature / date

Score: _________

Reviewer signature / date__________________________________________

*Appendix N: Positive Malaria Blood Smear Report*

The following individual is **POSITIVE FOR MALARIA**…..

ID# / Initials	_______________

Date	_______________

Time	_______________

Microscopist	_______________

Initials	_______________

Appendix O: List of investigators and their roles and responsibilities.

| **Names** | **Study roles and responsibilities** |
| --- | --- |
| Michele D. Spring, M.D. | **Principal investigator.** Physician responsible for the overall conduct of the study |
| D. Gray Heppner, COL, MC  Christian Ockenhouse, COL, MC Kent E. Kester, COL, MC  James F. Cummings, LTC, MC  Douglas S. Walsh, COL, MC  Mark G. Kortepeter, LTC, MC | **Sub-investigators.**  Physicians whose role is to participate in participant recruitment, screening, immunization, follow-up of study participants.  *Note that the physicians directly participating in immunization will not be involved in post immunization assessment of reactogenicity to maintain study blind. However, all physicians will be involved in the challenge follow-up |
| Christian Ockenhouse, LTC, MC | Responsible for the molecular profiling of immune responses after immunization using high-density oligonucleotide microarrays |
| Evelina Angov, Ph.D. | **Sub-Investigator**. Responsible for conducting pLDH GIA assays at WRAIR |
| David Lanar, Ph.D. | Responsible for development of malaria vaccine antigens/recombinant proteins |
| Urszula Krzych, Ph.D. | **Sub-Investigator**. Responsible for cellular immunology assessment at WRAIR |
| Ted Hall, Ph.D. | Responsible for clinical microscopy during the challenge phase |
| Ann Stewart, DVM, Ph.D. | **Sub-Investigator**. Responsible for humoral serology assessment at WRAIR |
| Jackie Williams, LTC, MS | Responsible for malaria culturing and will provide oversight for the malaria challenge |
| J. David Haynes, M.D. | Responsible for conducting exploratory GIA assays at WRAIR |
| Sheetij Dutta, Ph.D. | Responsible for conducting exploratory IFA and PIA assay at WRAIR |
|  |  |
|  |  |
| **Names** | **Study Roles and Responsibilities** |
| Carole Long, Ph.D. | Responsible for conducting exploratory GIA assays at NIH |
| Clara Brando, Ph.D. | Responsible for NK cell assays |
| Ronald Taylor, Ph.D. | Responsible for complement assays at UVA |

***Appendix P: Personnel***

***Principal Investigator***

Michele D. Spring, M.D**.**	Department of Immunology

Division of Communicable Diseases and Immunology

Walter Reed Army Institute of Research

503 Robert Grant Avenue

Silver Spring, MD 20910

Tel: 301-319-9307

Fax: 301-319-7358

Email: michele.spring@amedd.army.mil

**Sub-Investigators:**

D. Gray Heppner, COL, MC	Department of Immunology, WRAIR

Christian Ockenhouse, LTC, MC	Tel: 301-319-9543, Fax: 301-319-7358

James F. Cummings, LTC, MC

Evelina Angov,, PhD

V. Ann Stewart, DVM, PhD

Urszula Krzych, PhD

Kent E. Kester, COL, MC	Department of Clinical Trials, WRAIR

Douglas S. Walsh, COL, MC	Tel: 301-319-9660, Fax: 301-319-9585

Mark Kortepeter, LTC, MC	Infectious Disease Service, Walter Reed Army Medical Center (WRAMC)

Tel: 202-782-1663, Fax: 202-782-3765

**Research Associates:**

David E. Lanar, PhD	Department of Immunology, WRAIR

Ted Hall, PhD	Tel: 301-319-9543, Fax: 301-319-7358

J. David Haynes, MD

Sheetij Dutta, PhD

Clara Brando, PhD

Jackie Williams, LTC, MS	Department of Entomology, WRAIR

Tel: 301-319-9557

Carole Long, PhD	National Institute of Allergy and Infectious

Disease, National Institutes of Health

Malaria Vaccine Development Unit

5640 Fisher Lane

Rockville, MD 20852

Ronald Taylor, PhD	Department of Biochemistry and Molecular Genetics

University Of Virginia (UVA)

P.O. Box 800733

Charlottesville, VA 22908-0733

**Medical Monitor:**

Peter J. Weina, LTC, MC	Walter Reed Army Institute of Research

503 Robert Grant Avenue

Silver Spring, MD 20910

Tel: 301-319-9956

Fax: 301-319-7360

Quest Diagnostics, Inc.	1901 Sulphur Spring Road

Baltimore, MD 21227

(410) 247-9100

**Preclinical Product Development**

Carter Diggs, MD, PhD	United States Agency for International

Lorraine Soisson, PhD	Development

Malaria Vaccine Development Program

1300 Pennsylvania Avenue

Ronald Reagan Building, 3^rd^ Floor

Washington, D.C. 20523

**Funding Sponsor Representative:**

Eveline Tierney	PATH Malaria Vaccine Initiative (MVI)

7500 Old Georgetown Road

Bethesda, MD 20814

Tel: 240-395-2700

Email: etierney@malariavaccine.org

Appendix Q: Amendments/Modifications to the Study Protocol
